# A genome-wide map of circular RNAs in adult zebrafish

**DOI:** 10.1038/s41598-019-39977-7

**Published:** 2019-03-05

**Authors:** Disha Sharma, Paras Sehgal, Samatha Mathew, Shamsudheen Karuthedath Vellarikkal, Angom Ramcharan Singh, Shruti Kapoor, Rijith Jayarajan, Vinod Scaria, Sridhar Sivasubbu

**Affiliations:** 1 GN Ramachandran Knowledge Center for Genome Informatics, CSIR Institute of Genomics and Integrative Biology (CSIR-IGIB), Mathura Road, Delhi, 110025 India; 2 Genomics and Molecular Medicine, CSIR Institute of Genomics and Integrative Biology, Mathura Road, Delhi, 110025 India; 3 Academy of Scientific and Innovative Research, CSIR Institute of Genomics and Integrative Biology South Campus, Mathura Road, Delhi, 110025 India

## Abstract

Circular RNAs (circRNAs) are transcript isoforms generated by back-splicing of exons and circularisation of the transcript. Recent genome-wide maps created for circular RNAs in humans and other model organisms have motivated us to explore the repertoire of circular RNAs in zebrafish, a popular model organism. We generated RNA-seq data for five major zebrafish tissues - Blood, Brain, Heart, Gills and Muscle. The repertoire RNA sequence reads left over after reference mapping to linear transcripts were used to identify unique back-spliced exons utilizing a split-mapping algorithm. Our analysis revealed 3,428 novel circRNAs in zebrafish. Further in-depth analysis suggested that majority of the circRNAs were derived from previously well-annotated protein-coding and long noncoding RNA gene loci. In addition, many of the circular RNAs showed extensive tissue specificity. We independently validated a subset of circRNAs using polymerase chain reaction (PCR) and divergent set of primers. Expression analysis using quantitative real time PCR recapitulate selected tissue specificity in the candidates studied. This study provides a comprehensive genome-wide map of circular RNAs in zebrafish tissues.

## Introduction

The recent years have seen an in-depth annotation of transcriptome using next-generation sequencing technologies, significantly enhancing the repertoire of transcripts and transcript isoforms identified for protein-coding as well as noncoding RNA genes^1^. Circular RNAs encompass one such transcript isoform that has been recently identified in Humans^2^ and model organisms including mouse^2^, worms^2^, fruitfly^3^ and viruses^4^. In contrast to the linear transcript isoforms, circular RNAs are formed by a unique mechanism of back-splicing between the 3′ and 5′ splice sites of a gene, resulting in circularised transcript isoform^5^. Existence of circular RNAs have been previously studied in viruses, but their biogenesis and molecular function in higher eukaryotic systems has remained elusive, though a number of recent evidence suggests their role in regulating important biological functions^6,7^. Though a few candidates have been catalogued in humans, as in the case of *DCC* (Deleted in Colorectal Carcinoma), a candidate tumour suppressor gene^8^, these RNAs were largely considered as arising from sequencing artefacts, sequencing errors or products of tandem duplication or reverse transcription-induced template switching. In addition, these transcripts remained elusive because standard transcriptome profiling approaches largely enriched the poly-adenylated fraction of the transcriptome. The large-scale discovery of circular RNAs have been made possible by deep sequencing approaches and mapping algorithms, which enable the accurate annotation and quantitation of circular RNAs^9^. The methodologies rely on the fact that in contrast to linear isoforms of the transcript that are amenable to exonuclease activity, circular RNAs are resistant to exonuclease activity due to their specific conformation^5^. In addition, the back-splicing forms a new sequence conformation which could be accurately identified by split-read mapping of deep sequencing reads. Recent genome-scale annotation of circular RNAs in humans^2,9,10^ and mouse^11^ have suggested a prevalent expression of circular RNAs in various tissues and cell types. Many of these orthologous loci were shown to encode for circular RNAs. Evidence suggests that significant number of circular RNAs could function as microRNA sponges^12^. The human circular RNA, CIRS7 (previously known as CDR1AS) has been shown to be associated with midbrain development in zebrafish by virtue of being a microRNA sponge for miR-7^2^. Additional evidence on the role of circular RNAs in modulating the pathophysiology of diseases are emerging^13,14^.

*Danio rerio*, zebrafish, has been a popular model system to understand human diseases^15,16^. It has been estimated that over 70% of human genes have an orthologue in zebrafish^17^. Apart from the advantages of being small and embryos being transparent in early development, availability of efficient tools to manipulate the genome and the availability of genome sequences have been well documented^16,18,19^. Recent reports employing RNA sequencing approach has characterized the transcriptome of zebrafish, apart from uncovering a large repertoire of long noncoding RNAs expressed in developmental stages^20^ as well as adult tissues^21^. Nevertheless, the earlier RNA-Seq approaches were limited to identifying poly-adenylated transcripts. The availability of approaches to sequence whole RNA has provided a unique opportunity to identify novel transcript isoforms including circular RNAs in zebrafish.

Recently Shen *et al*. reported the identification of circRNAs in zebrafish^22^ but the expression of these circular RNAs in developmental stages and their tissue specificities remain unknown.

In the present study, we use an RNA-seq approach along with computational algorithms to discover 3,428 novel circular RNAs in adult zebrafish. A significant number of the circular RNAs showed tissue specificity. A subset of newly discovered circular RNAs were validated using experimental approaches, and their expression analysis revealed strong concordance with the tissue specificity observed. Our analysis also a subset of circRNAs are derived from orthologous genes in zebrafish and human indicating an evolutionarily conserved molecular mechanism between two species. This report describes a comprehensive genome-wide map of tissue-wise circular RNAs in zebrafish and uncovers a hitherto unknown repertoire of circular RNAs in the organism.

## Materials and Methods

### Ethics statement

Experiments using Zebrafish were performed as per the recommendations and guidelines prescribed by the CSIR Institute of Genomics and Integrative Biology (CSIR- IGIB), India and approved by the Institutional Animal Ethics Committee (IAEC) of CSIR-IGIB which included proactive measures to minimise animal suffering.

### Zebrafish Husbandry

The adult Assam wild type zebrafish (hereafter abbreviated as ASWT) maintained at the CSIR-Institute of Genomics and Integrative Biology were used in this study^16^.

### RNA isolation and library preparation

Tissues were isolated from adult zebrafish anaesthetized using 0.004% Tricaine (Sigma, USA). Extreme care was taken to avoid contamination to obtain pure homogenous tissue samples. Blood, brain, muscle, gills and heart tissues were extracted for this study. The tissues were repeatedly washed in PBS to remove contaminating debris. The tissue samples were homogenized in Trizol for cell lysis (Invitrogen, USA). RNA was isolated from the homogenized tissue samples using RNeasy kit (Qiagen, USA). Sample preparation for sequencing was carried out using Truseq stranded RNA sample preparation kit (Illumina, USA) as per supplier’s instructions. In order to remove the ribosomal RNA (rRNA), one microgram of total RNA was hybridised with Ribo-zero gold rRNA removal probe. Upon removing rRNA, the samples were processed for fragmentation in the presence of ionic cations at 37 degree Celcius. First stranded complementary DNA (cDNA) was prepared by random hexamers and superscript II reverse transcriptase (Invitrogen, USA) in presence of Actinomycin D to facilitate RNA dependent synthesis for improving strand specificity. The second strand was synthesised with second strand cDNA mix containing dUTP instead of dTTP and subjected to A base addition followed by adapter ligation. Final libraries were prepared by amplifying adapter ligated double strand cDNA. Clusters were generated on Hiseq flow-cell v3 (Illumina) in cBot according to standard protocol (Illumina,USA).

### Sequencing and Alignment

**Figure 1:**
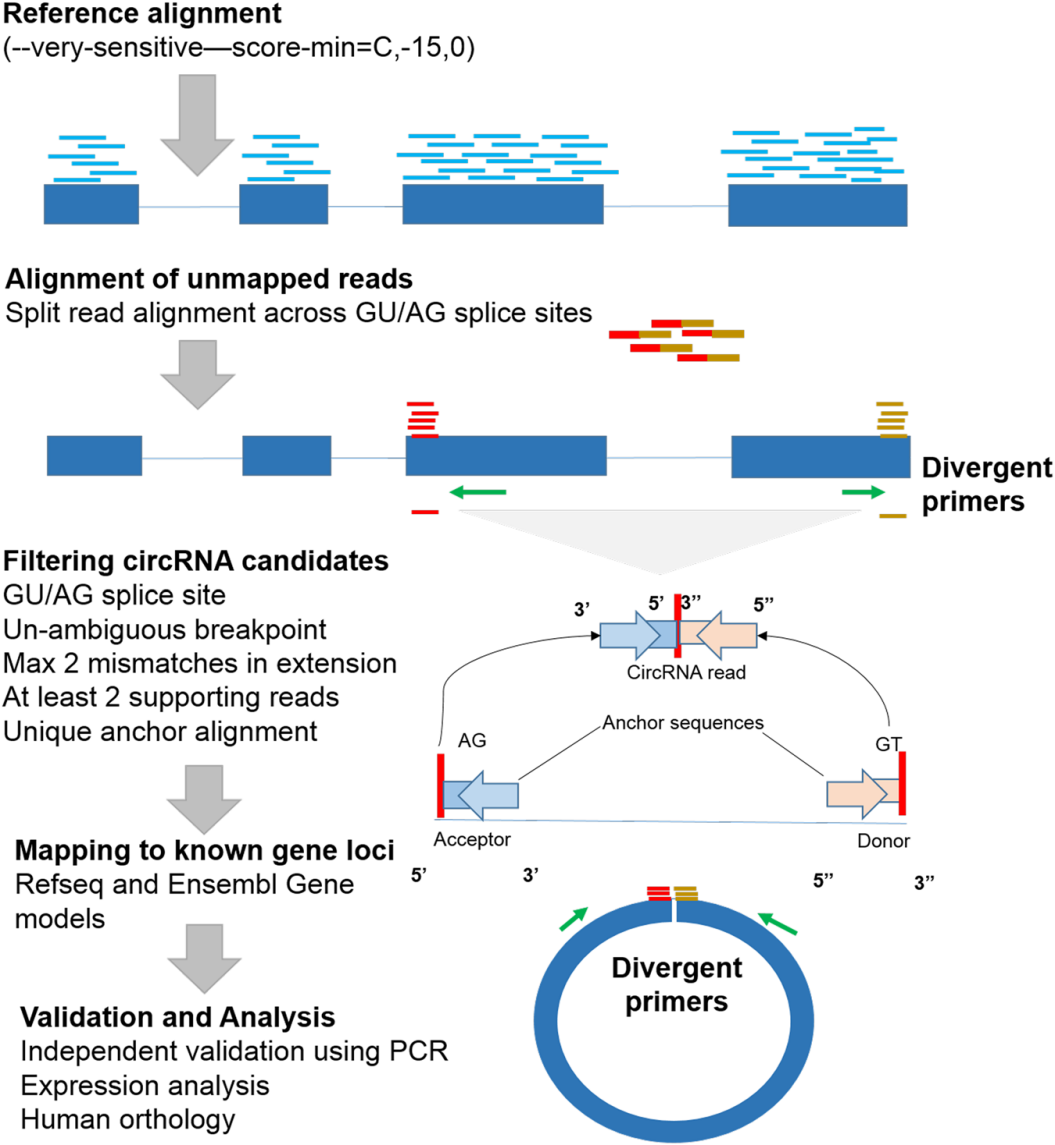
Schematic description of the bioinformatics analysis pipeline used for the discovery and validation of novel circular RNAs in different tissues of zebrafish.

We generated on average 10–12 million reads for each tissue with 101 bp fragment length using Illumina HiSeq. 2500 (Illumina Inc, SA, USA) as per standard protocols provided by the manufacturer. Adapter trimming, quality filters and trimming was implemented using Trimmomatic PE^23^. We used a Phred quality filter cut-off 30. We have submitted sequencing reads for each tissue on SRA archive with submission ID SUB3157917. To identify circular RNAs, we used the circular RNA pipeline findcirc, described by Memczak and co-workers^2^. Findcirc pipeline was designed to identify circular RNA back splice junctions and identifies reads overlapping the back-splice site. The pipelineuses customized scripts to filter out potential false positives based on the read alignment scores. The pipeline is very user friendly and requires modest computational hardware. Anchor sequence of 20 nucleotides from head and tail of the reads are mapped and extended until full reads were mapped. Contiguous reads with maximum of 2 mismatch and a splice site of GU/AG were applied as filters to enhance the specificity of circular RNA identification. We used the Zv9 reference genome for zebrafish, downloaded from UCSC genome bioinformatics^24^. Reads were aligned to the indexed Zv9 zebrafish genome using a read aligner tool Bowtie2^25,26^. We used the options–very-sensitive for end-to-end alignment and minimum score alignment C,-15,0 as used in previous studies^2^. Reads that aligned to the full length genome were discarded. We extracted 20 bps anchor sequences from both the ends of the remaining unmapped reads. These anchors were aligned back to probe for splice site junctions. From the splice site junction, 20 bps were extended in the reverse direction keeping their paired order under consideration. Anchors aligned in reverse direction (5′ head to 3′ tail junction) were considered potential circular RNA sequences as shown in the Fig. 1. The output file in BED format was generated with the coordinates for circular RNAs splice junctions. These candidates were further filtered and selected for further validation on the basis of tissue specificity and functional relevance mentioned in literature.

### Identification of false-positive candidate circRNAs from PolyA RNA datasets

We have used publically available polyA RNA-seq datasets to identify false-positive candidate circular RNA. The unique backsplice junctions is a specific characteristics of circular RNAs. By the premise, any candidates the computational pipeline would detect from RNAseq of samples prepared by the standard polyA based primers would be artefacts and therefore would enable estimate of the false positives. The list of accession number for datasets used for RNA-seq is mentioned in Supplementary Data Table 1.

### Identification of circular RNAs using CIRI

We have used another circRNA identification pipeline, CIRI. CIRI is a popular circular RNA identification as well as annotation tool using RNA-sequencing reads^27^. This tool is not dependent on annotation or circular RNA enrichment, rather uses unique paired chiastic clipping model to identify circular RNAs from the read alignment file. This tool uses BWA as aligner to generate alignment file in SAM format. In the present analysis, BWA-MEM algorithm was used to generate SAM file for each tissue RNA-seq reads and the files were further processed through the CIRI pipeline to identify circRNA junctions.

### Genome wide distribution of circular RNAs

The genomic coordinates corresponding to RefSeq genes and their untranslated regions (UTRs) and coding sequences (CDS) were retrieved from UCSC genome browser using the Table View option. Similarly the coordinates were retrieved from Ensembl using BioMart. The circular RNA splice junctions were mapped separately to the genomic contexts. We mapped the positions specifically to the whole gene, exons, introns, 5′ UTR, 3′ UTR and 1000 bases upstream and downstream of the genes. The co-ordinates of lncRNAs were downloaded from zflncRNApedia, a comprehensive resource for zebrafish lncRNAs maintained by our group^28^. The dataset encompassed a total of 2,267 lncRNAs.

### Experimental validation of the back splice junctions using polymerase chain reaction (PCR)

Divergent and convergent primers were designed to selectively amplify the circular and linear transcripts respectively, using coordinates predicted through the bioinformatics pipeline. cDNA was prepared from 200 ng of total RNA from individual tissues, isolated as described before. First strand cDNA synthesis was performed using Superscript II reverse transcriptase (Invitrogen, USA). The genomic DNA (gDNA) was isolated from ASWT zebrafish using the standard isopropanol precipitation method^29^. In order to validate the origin of the back-spliced transcripts from the transcriptome, and not from the genome, PCR amplification was performed on cDNAs of corresponding tissues and gDNA of ASWT zebrafish, simultaneously, using divergent primers. Convergent primers of beta- actin were used as control for all combinations of reactions.

### Confirmation of the circRNA candidates using RNAase R to deplete non-circular isoforms

RNA isolated from each of the 5 tissues was treated with RNAse R as described in Memczak *et al*. 2013. 5ug of RNA was treated with 15 units of RNAse R(Epicentre, Illumina, San Diego, CA, USA) for 15 minutes to degrade linear RNA and enrich circular RNAs. RNAse R depleted (RNAseR+) and untreated RNA (RNAseR−) was further purified using LiCl method and RNA conc was determined. 1ug of the untreated RNA and same volume of RNAse R treated RNA was converted to cDNA using Superscript II reverse transcriptase (Invitrogen, USA). PCR amplification was performed for selected circular RNA candidates and two linear mRNA as a control in the reaction. These two liner mRNA controls included one tissue specific positive control and second universal control as beta-actin. Primer sequences for PCR are summarised in Supplementary Data Table 2.

### Expression analysis using quantitative real-time PCR

Expression analysis was attempted for selected candidate circular RNAs, which showed tissue-specific expression patterns. RNA was isolated from blood, brain, muscle, heart and gills using RNeasy kit (Qiagen, USA) and cDNA was synthesized, as previously described. Linear and circular transcript levels were assayed by quantitative Real Time Polymerase Chain Reaction (qRT-PCR), carried out using Sybr Green mix (Roche, Germany) and detected by Lightcycler LC 480 (Roche). Respective gene markers for individual tissues were used as positive controls in all experimental sets. The markers *tal1*, *mdka*, *tnnt2c, vmhc* and *cx44.2* were used for the corresponding tissues blood, brain, muscle, heart and gills respectively. Primer sequences for qRT- PCR are summarised in Supplementary Data Table 2.

### Human orthologues for zebrafish genes harbouring circular RNAs

We retrieved the human orthologues for zebrafish genes, which had at least one circular RNA originating from the gene boundaries. The circular RNA for the human orthologues were retrieved from circBase, a comprehensive online resource for circular RNAs^30^. Disease genes were mapped from the Online Mendelian Inheritance in Man (OMIM) and zebrafish mutant information was retrieved from ZFIN using the Ensembl gene ID.

A schematic representation summarising the approach used for identification and validation of circular RNAs in zebrafish is summarised in Fig. 1.

## Results and Discussion

### Summary of the RNA-Seq data generated and analysis

We generated 250 million reads in total for all the samples encompassing different adult tissues. Each dataset had at least 2 million reads. The reads on an average had over 60% alignment with the reference genome. The details of the reads generated and alignment percentage is summarised in Table 1. We further retrieved the unmapped reads, which did not map contiguously. We used an algorithm reported by Memczak and co-workers, previously used to characterize the human circular RNA compendium^2^. The algorithm uses unmapped reads as templates, aligns and extends 20 nucleotides from either end of the reads, called ‘anchor sequences. These anchor sequences were further extended. The reads with flanking splice site GU/AG were included as potential circular RNA reads. We allowed maximum of 2 mismatches and unique alignment with best matching score in the extension of anchor sequences. The algorithm was applied on unmapped reads from each dataset independently. We used an empirical cut-off of at least 2 reads mapping the back-splice junction to prioritise high quality circular RNAs. Our analysis revealed that ~40% of unmapped reads spanned the back-splice junctions in each dataset (Table 1).

**Table 1:** Summary of data generated for adult zebrafish tissue.

Sample	Total no. of reads	Paired/single-end	Alignment Mapped reads	Alignment anchor sequences	No. of circular RNA
Blood	20461146	paired	75.29%	78.39%	1196
Brain	16700545	paired	81.53%	80.26%	327
Muscle	20362229	paired	80.21%	80.27%	686
Heart	25765610	paired	64.89%	78.87%	1014
Gills	22524306	paired	62.82%	71.43%	1228

### Abundant number of circular RNAs in zebrafish

**Figure 2:**
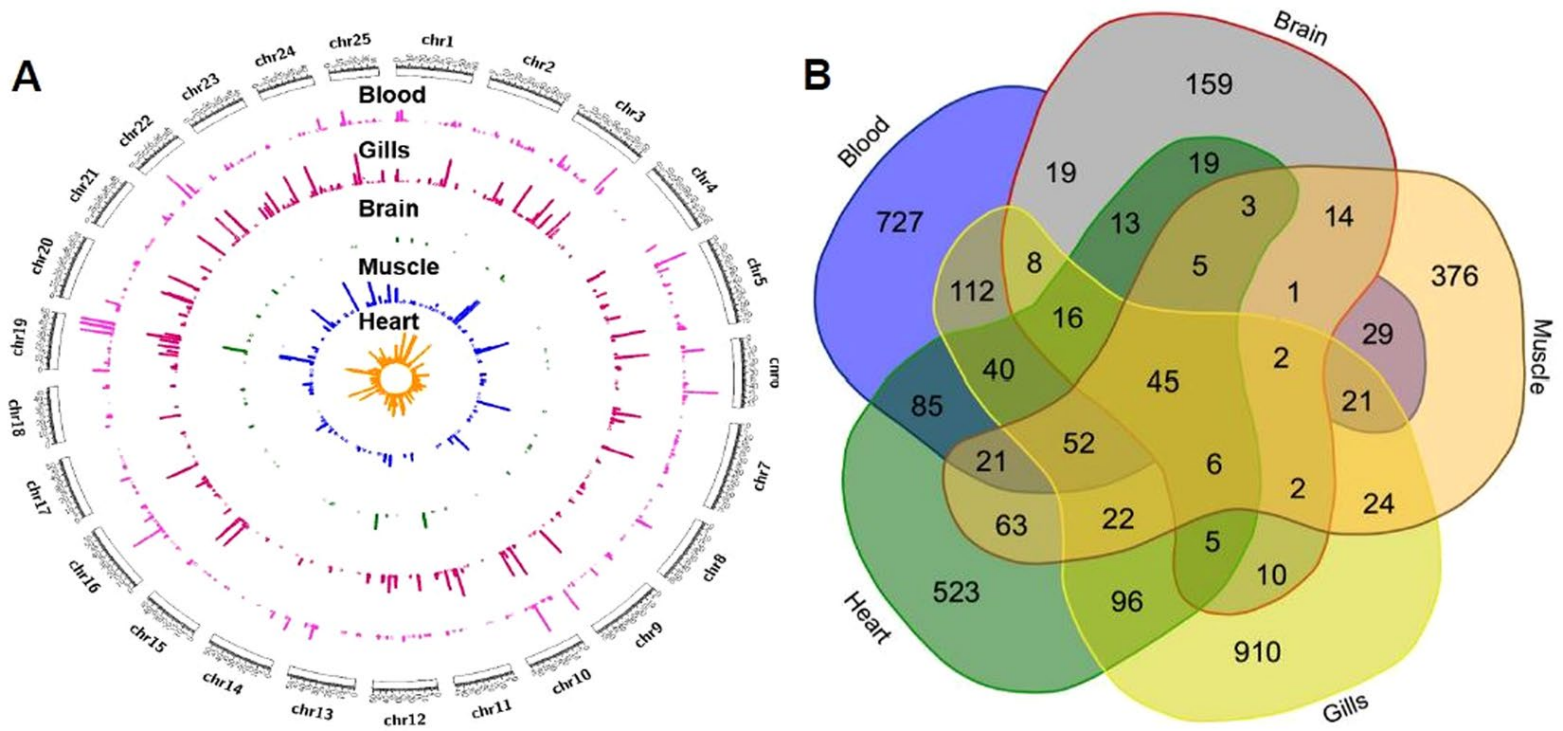
Tissue distribution and repertoire of predicted circular RNAs (**A**). Circos representation for tissue-wise distribution of circular RNAs with respect to read coverage across genomic co-ordinates. (**B**) Illustration of the number of circular RNAs shared between tissues analysed in the present study.

Our analysis revealed a hitherto unknown and abundant repertoire of circular RNAs in zebrafish. We identified a total number of 3,428 circRNAs in zebrafish. The chromosome-wide circos-map of circular RNAs in zebrafish is summarised in Fig. 2A. The circular RNAs and the back-splice junctions are available as a BED file (Supplementary Data Table 3).

We also analysed polyA RNA-seq datasets as described in the Materials and Methods section using the pipeline described by Memczak *et al*., to identify potential false positive circular RNA candidates. The datasets analysed included polyA RNA-seq datasets for tissues including Blood, Brain, Muscle and Heart. Our analysis identified 740 circRNA candidates. Out of these 740 circRNA junctions, 249 were common with circRNA junctions identified from non-polyA datasets. The list of the junction predicted from polyA datasets is shown in Supplementary Table 4. We have shown the overlap between polyA and non-polyA circ-junctions in Supplementary Figure 1.

**Figure 3:**
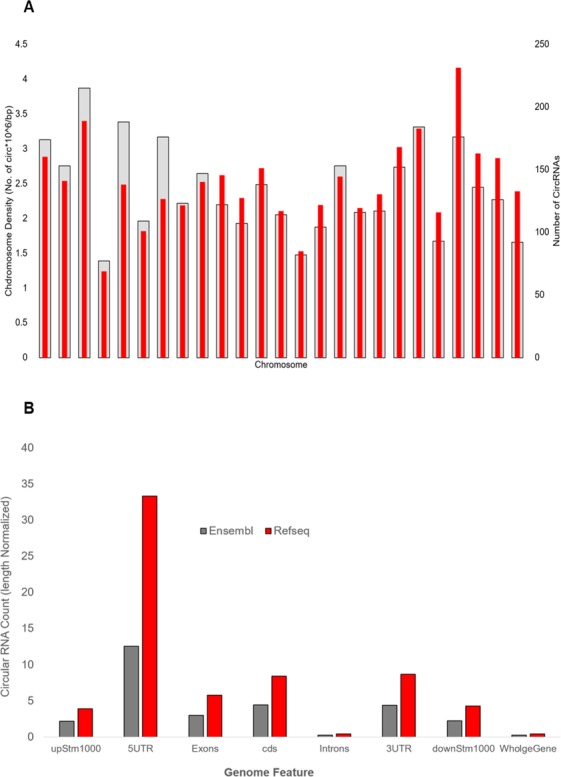
Genomic distribution of the circRNAs (**A**). Density-wise distribution of circular RNA across chromosome length (**B**). Genome-wide distribution of circular RNAs (length normalized for each region) for Ensembl and RefSeq respectively.

The circular RNAs were distributed across all chromosomes in zebrafish, with the maximum number of candidates arising from chromosome 3. Normalising for the length of the chromosomes, the zebrafish chromosome 22 had the largest density (per million bases) of circular RNAs, with approximately 176 circular RNAs arising from the chromosome (Fig. 3A).

### Cross comparison of candidate circular RNAs with an alternate computational pipeline

We also used an additional pipeline, CIRI, to identify circular RNA junctions to be able to compare and cross-validate the candidate circular RNAs identified FindCirc. The number of circRNAs identified from this pipeline are listed in the Supplementary Table 5. The independent pipeline identified a total of 1,919 circRNA junctions out of which 1,222 circRNA candidates were common between both circRNA pipelines (Supplementary Figure 2). The list of circRNA junctions identified from this pipeline is given in Supplementary Table 6 and the list of common junctions between findcirc and ciri is mentioned in Supplementary Table 7. Further analysis was done using the list of circRNAs identified from FindCirc pipeline.

### Genomic context of the circular RNAs

We analysed the genomic context of the circular RNAs vis-a-vis annotated Ensembl and Refseq genes. Our analysis suggests that a significant proportion of the circular RNAs in zebrafish arise from genic loci. Out of all, 1,833 circular RNAs (54.47%) map to RefSeq gene loci, whereas 3,015 (87.95%) map to Ensembl gene loci (Fig. 3B). Summary of the circRNA mapped over Refseq and Ensembl is shown as Table 2. A majority of these loci were protein coding. The circular RNAs mapping to RefSeq and Ensembl protein coding genes were 1,743 (50.85%) and 2,909 (84.86%) respectively. These observations are also in agreement with previous analyses on mammalian circular RNAs^30^. We overlapped the circular RNA splice sites with the ensemble annotation file and found ~27% of novel splice sites FindCirc circRNA and ~22% of novel splice sites from CIRI circRNAs. The venn diagram is shown in Supplementary Figure 3.

**Table 2:** Summary of circular RNAs identified in the tissues considered and the mapping of genome contexts.

Tissue	Total number of circRNAs	No. of circRNAs mapping to gene loci (RefSeq)	Refseq overlapped circ count %	No. of circRNAs mapping to gene loci (Ensembl)	Ensembl overlapped circ count %	No. of circRNAs mapping to lncRNA	zflncRNApedia (lncRNA) overlapped circ count %
Blood	1196	674	56.35	1067	89.21	26	2.17
Brain	327	181	55.35	287	87.77	6	1.83
Muscle	686	420	61.22	636	92.71	19	2.77
Heart	1014	568	56.02	913	90.04	38	3.75
Gills	1371	724	52.81	1180	86.07	59	4.30

For the circular RNAs mapping to protein coding gene loci, we further examined whether they have specificity/preponderance to specific gene features. To this end, we examined the splice junctions with respect to the various gene features: CDS, Introns, 3′ and 5′UTRs and 1000bases upstream and downstream of the genes. Since the genomic regions encompassing the features differed in their overall size, a normalised measure adjusting for the bases in each feature was also computed. Our analysis revealed circular RNAs have a preponderance to the 5′UTR of genes. This was followed by the 3′UTR and CDS. Introns had the least preponderance, as expected by the fact that they do not form part of the messenger RNA. This preponderance suggests a potential regulatory nature of circRNAs. These observations were consistent across the RefSeq and Ensembl gene annotations. Incidentally, the 1000 bp upstream and downstream of the gene loci also had a significant number of splice junction mappings (Supplementary Table 8). This could arise from mis-annotation of gene/transcript boundaries. Many of the junctions were examined manually and had read coverage (data not presented) which supports this.

Out of 3428 circRNAs, 1132 were overlapping with 5′UTR, 1197 with 3′UTR and 2919 were overlapping with intronic region. Out of these, we found circular RNAs exclusively originating from introns as well as UTRs. We observed 16, 34, 175 circRNAs originating from introns, 5UTRs and 3UTRs respectively. These circular RNA might have a role in regulation of transcription as well as in cross-talk with miRNAs. Gene Ontology (GO) analysis of genes from exonic circRNA clearly showed that most of the genes are either part of chromatin coiling and nuclear processing (Supplementary Table 9). Few genes were also involved in oxidative phosphorylation, ATP synthesis and metal transport.

### Circular RNAs originates from long noncoding RNA loci

We also examined whether circular RNAs mapped to previously annotate long noncoding RNAs. We mapped the circular RNA splice junctions to lncRNA co-ordinates in zflncRNApedia, well annotated and comprehensive resource of long noncoding RNAs. Our analysis revealed a total of 105 (3.06%) circular RNAs in zebrafish mapped to long noncoding RNA loci (Supplementary Table 10).

### Circular RNAs show tissue specificity

We also analysed the circular RNAs, which were shared between the five adult tissues/organs considered in the present analysis. A large majority of the circular RNAs amounting to 2695 (78.62%) showed a tissue restricted expression. The largest number of circular RNAs specific for a tissue was in gills, which had a total of 910, followed by blood (727), heart (523), muscle (376) and brain (159). A total of 733 (21.38%) circular RNAs were shared between any two of the five tissues analysed, out of which only 45 (1.31%) of the circular RNAs were shared between all the five tissues. Functional analysis of the protein-coding genes harbouring these circular RNAs revealed most annotated genes are involved in regulating signalling pathways. The overlaps of circular RNAs discovered in the different tissues is summarised in Fig. 2B.

We selected candidate circular RNAs for independent validation using PCR followed by quantitation using qRT-PCR using a set of divergent primers for the circular RNA junction and convergent primers for the linear transcript isoforms.

The genes for validation were randomly selected and include *hbaa, ank, ikzf, map2k5* and *epb4.1* from blood dataset; *dab1, fam107a, clstn1, sox9b* and *taf6* from brain dataset; *rmb41, znf609a, eef1al* and *furina* from heart dataset; *srek1, nexn, tnnt2a, ttnb1* and *ttnb2* from muscle dataset and *zp3, atp1a1a, sd:key241l7.3, sd:key194m7.4* and *sd:key146l10.7* from gill dataset. This list also included a few candidates which were tissue restricted *taf6* (brain), *epb4.1* (blood), *ttnb1* (muscle). The selection includes circular RNAs arising from genes associated with a variety of functions. For example *taf6* (transcription initiation factor subunit 6) is required for transcription activity^31^, while *ttnb1* codes for a muscle protein^32^. *furina*, has a role in functional in processing proteins, their regulation and trafficking^33^ while *epb4.1* is an erythrocyte membrane protein^34^.

### Validation of predicted circular RNAs using PCR

**Figure 4:**
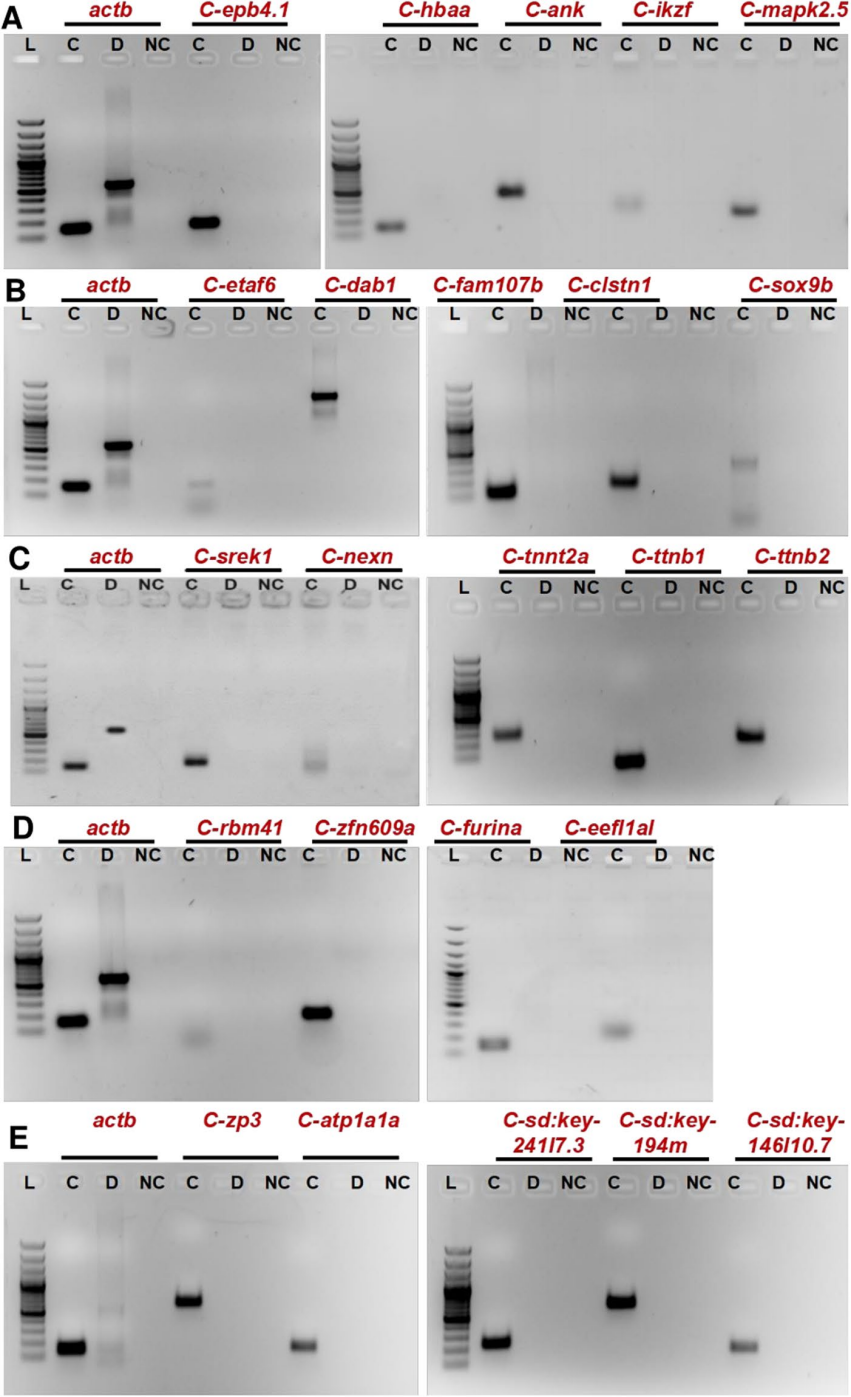
Validation of predicted novel circRNA in different tissues. (**A**–**E**) PCR based confirmation of circRNA junctions in (**A**) Blood, (**B**) Brain, (**C**) Muscle, (**D**) Heart and (**E**) Gills, respectively. Divergent primers facing outwards were used to amplify cDNA (**C**), DNA (**D**) and negative control (NC). Convergent primers for *actb1* gene used in every tissue as control. L- 100 bp Ladder (Fermentas, USA).

Towards validating the predicted circular RNAs, we used a PCR based approach to identify the back-splice junctions. The experiment was designed based on the logic that the convergent (conventional) primers would amplify the linear isoform of RNA, whereas the divergent primers would amplify only the circular RNA isoform. The divergent primers of length ~20 nucleotides were designed facing the back-splice junctions to obtain an amplicon of approximately 200 bp. cDNA prepared from individual tissues and genomic DNA isolated from ASWT zebrafish were used for the validation. We selected four candidates, arising from the exonic and intronic loci in the zebrafish genome. The amplicons spanning the junction of the circular RNAs were amplified from the cDNA, whereas no product was obtained in case of the gDNA as template. For all reactions, beta-actin (*actb1)* was used as the positive control, for which the convergent primers were able to amplify the product in both cDNA and as well as gDNA as the template. We could successfully amplify the circular isoform for all the candidates tested. The splice junctions were further confirmed for a few selected candidates by capillary sequencing (Eurofins, India). Results for the experimental validation is summarised in Fig. 4.

**Figure 5:**
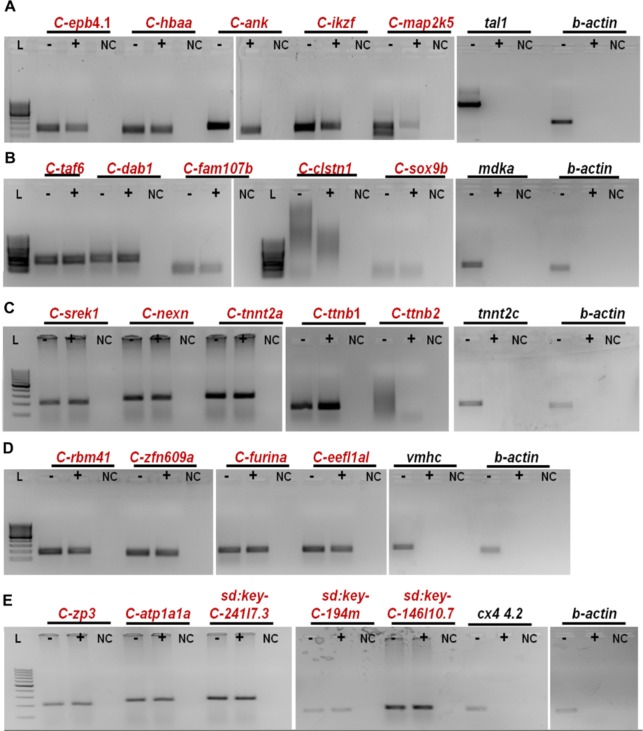
Validation of circRNA with RNAse R treatment (**A**). Blood (**B**) Brain (**C**) Muscle (**D**) Heart (**E**) Gills. L-ladder, +represents RNAse treated and −represents not treated with RNAse. We have used two positive controls for each tissue. One is tissue specific positive control and second is beta-actin.

An additional validation was performed for the candidate circRNAs using RNAse R depletion to gain more confidence on the candidate circRNAs. RNAse R depletes linear transcript isoforms, thereby reducing the possibility of PCR amplification arising from mis-spliced linear isoforms. Presence of product of circular RNA in RNAse depleted (RNAse+) and untreated (RNAse R−) samples in all the 5 tissues confirmed the existence of circular RNAs. Whereas product for linear mRNA (*actb* and linear counterpart of one of circular RNA of a given tissue) was only observed in the RNAse R - sample, which explain the activity of the RNAse R treatment (as shown in Fig. 5). Interestingly, out of selected candidates, 3 candidates were also identified in polyA datasets. These 3 candidates included circular RNA junction for hbaa1, eef1al and ttnb1. Interestingly, these candidates are also validated using RNAse treatment even showing occurrence in polyA datasets, suggesting a small subset of circular RNAs could potentially be identified from polyA RNAseq datasets, and might represent transcript isoforms which could potentially be primed internally by oligonucleotides.

### Expression analysis of predicted tissue-enriched circular RNAs

**Figure 6:**
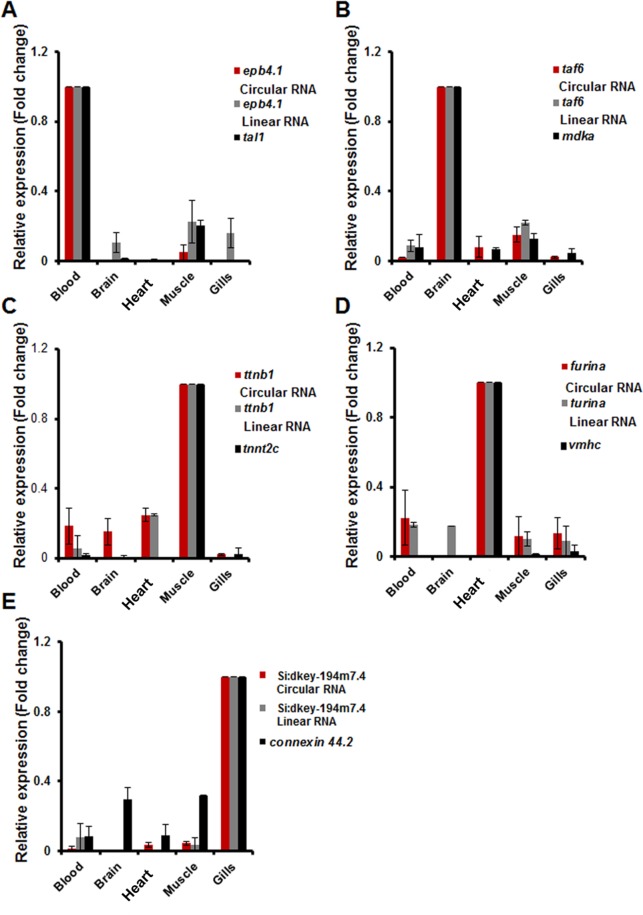
Quantitative validation of putative novel circular RNAs across different tissues. qRT-PCR based relative expression analysis of candidate circRNAs in (**A**). Blood, (**B**) Brain, (**C**) Muscle, (**D**) Heart, (**E**) Gills. The expression levels of linear transcripts of parent gene of each candidate circRNA were also estimated. Tissue specific protein coding gene marker *tal1*(blood), *mdka*(brain), *tnnt2c*(muscle), *vmhc*(heart) and *cx44.2*(gills) were used as controls. Data was normalized with the expression levels of *actb1*. Data was collected from independent experiments and represented as relative mean fold change ± SD across tissues. circRNAs examined in a particular tissue type displayed relatively high expression in the specific tissue when compared to other tissue types.

The experimentally validated circular RNA candidates were also further analysed for their expression in five tissues. The expression of the candidate circular RNAs were compared vis-a-vis selected protein coding genes previously shown to be high expression in the specific tissue under consideration. Expression analysis of the circular RNA candidates from each tissue by RT-PCR suggested their differential and spatially enriched expression. As shown in Fig. 6, circ *epb4.1* and circ *taf6* displayed their expression restricted only to blood and brain tissues, respectively. Apart from high expression in muscle, circ *ttnb1* showed marginal expression in blood, brain and heart. Similarly circ *furina* was highly expressed in heart, and *sd:key194m7.4* was enriched in gills, supporting our analysis.

### Circular RNA forming ability is enriched in human gene orthologues in zebrafish

We further examined circular RNAs in human-zebrafish orthologues. Out of the total gene repertoire in human genes, 19,571 genes share orthologue with 16,144 genes in zebrafish according to Ensembl. A total of 1,869 of the 2,539 genes which had circular RNAs in zebrafish had an orthologue in the human genome. We further examined specific cases of orthologues harbouring circular RNAs and additionally analysed whether their human orthologues also harbour circular RNAs. The 1,869 zebrafish genes which harboured circular RNAs had 1,954 human orthologues. Further analysis of these genes revealed 2,180 of these human genes harbour circular RNAs. The enrichment of zebrafish genes with circRNA forming ability when compared to ones which have human gene orthologues was significant (p < 0.05). As a case example, we focussed on one candidate circular RNA arising out of a gene conserved and harbour circular RNAs in human and zebrafish. The human *clstn1* gene and zebrafish *clstn1* both encode for 5 transcript isoforms. The zebrafish circular RNA was also validated on PCR assays. The five isoforms encompass transcripts of 6281, 6148, 2895, 2495 and 1746 bps in length translating to 954, 971, 964, 317 and 317 amino acids respectively. The human *clstn1* gene has 19 exons whereas zebrafish clstn1 orthologue has 18 exons in total. The human and zebrafish orthologues showed a high overall sequence identity of 73%. The exon-wise identity between human and zebrafish was 71% and protein sequence identity was 74%. Recent evidence from deep sequencing suggest that the gene loci encodes for 18 circular RNA junctions in human *clstn1*. We focused on the conserved regions and splice sites between human and zebrafish. The high degree of conservation between the two species and the overlapping circular RNA made us focus on the orthologous region in exon 12 of *clstn1* gene. In human, clstn1 has a role in mediating axonal anterograde transport of vesicles. Role of *clstn1* gene has also been studied in Alzheimer’s disease^35^.

Conservation and orthologue between both species in case of *clstn1* gene can potentially help us understand the role of the circular RNAs.

## Conclusions

In the present analysis, we provide a genome-scale map of circular RNAs in major tissues in adult zebrafish. Our analysis uncovers a total of 3,428 circular RNAs from 5 tissues which include blood, brain, heart, gills and muscle for zebrafish. Independent validation of the circular RNA junctions were attempted using divergent primer sets and polymerase chain reaction, which suggests high concordance. Further analysis revealed a large majority of the circular RNAs were derived from protein-coding gene loci. A small minority did arise from long noncoding RNA gene loci. Expression analysis revealed many of the circular RNAs showed tissue-specific patterns of expression. The expression levels of a few candidates were independently validated using RT-PCR, which suggested high concordance. Analysis also revealed that a number of zebrafish lncRNAs arise from human-orthologous genes in zebrafish, suggesting the utility and opportunity to study the biological functions and regulatory networks of human circular RNAs in zebrafish. Our analysis, to the best of our knowledge reports the comprehensive map of circular RNAs in zebrafish. We envision this report could serve as the starting point to understand circular RNA biology and its regulatory networks in key biological processes including development and disease in zebrafish.

## Supplementary information

supplementary_info
