# Supplementary material for: A genome-wide map of circular RNAs in adult zebrafish
Source: Sci Rep. 2019 Mar 5;9:3432. doi: 10.1038/s41598-019-39977-7 (PMC6401160; doi:10.1038/s41598-019-39977-7)
Supplement: Supplementary file 1 — supplementary_info [file 41598_2019_39977_MOESM1_ESM.pdf]

A genome-wide map of circular RNAs in adult zebrafish

Disha Sharma[1,3], Paras Sehgal[2,3], Samatha Mathew[2], Shamsudheen Karuthedath Vellarikkal[2,3], Angom Ramcharan Singh[2], Shruti Kapoor[1,3], Rijith Jayarajan[2], Vinod Scaria[1,3] & Sridhar Sivasubbu[2,3]

[1] GN Ramachandran Knowledge Center for Genome Informatics, CSIR Institute of Genomics and Integrative Biology (CSIR-IGIB), Mathura Road, Delhi 110025, India

[2] Genomics and Molecular Medicine, CSIR Institute of Genomics and Integrative Biology, Mathura Road, Delhi 110025, India

[3] Academy of Scientific and Innovative Research, CSIR Institute of Genomics and Integrative Biology South Campus, Mathura Road, Delhi 110025, India

Address for correspondence: Vinod Scaria (vinods@igib.in) and Sridhar Sivasubbu s.sivasubbu@igib.res.in

Protocol for isolation of Blood from Zebrafish

For isolating the blood RNA sample from the adult zebrafish same method was used as mentioned previously with some modifications (Pedroso et al., 2012 and kaushik et al.,2013). In brief, Zebrafish were anesthetized using Tricaine (Ethyl 3-aminobenzoate methanesulfonate salt; SigmaAldrich E10521), 0.04 mg/mL for 3−5 min. After anesthesia, fish were soaked using fresh kimwipes. The caudal fin was amputated with sterile fine scissors and fish was held in a vertical upside down position. Blood was collected from amputated region using p10 pipette in a microfuge tube containing 20µl of 0.5M EDTA solution. About 20-30µl of blood was collected from single or multiple fishes. During the collection microfuge tube was flicked several times for proper mixing. After the collection, it was centrifuged at 50 x g for 5 min at 4^0 to remove any tissue debris. The blood was transferred carefully to a fresh tube, 500µl of Trizol reagent was added and stored at -80^0 till further experiment. The RNA was isolated using RNAeasy mini kit (Qiagen, USA) as mentioned previously.

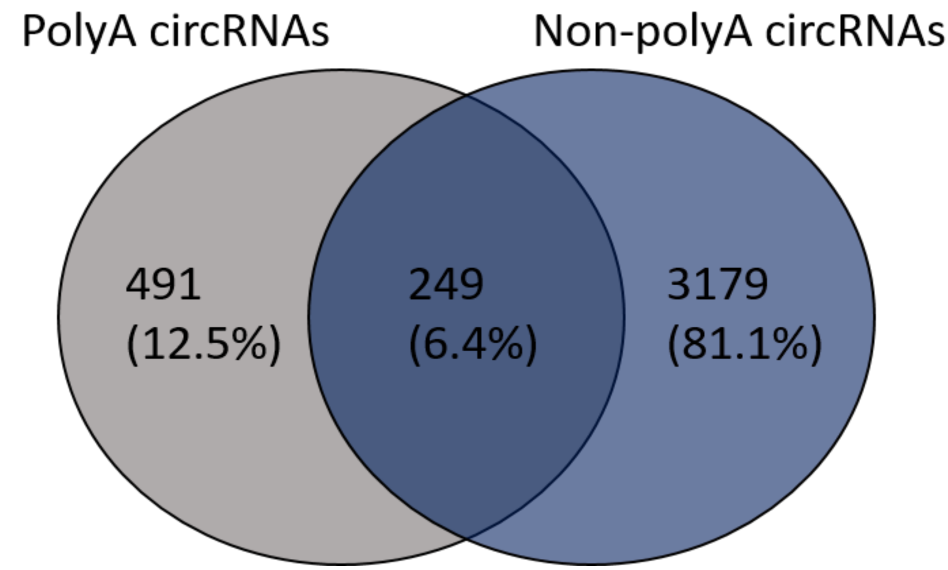

Supplementary Figure 1. Venn diagram showing non-polyA and polyA circular RNAs numbers.

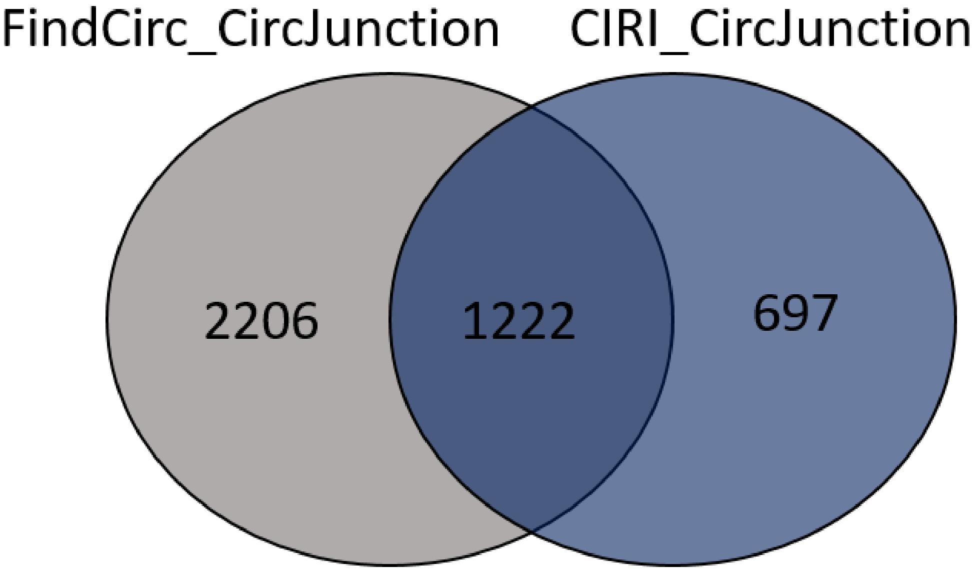

Supplementary Figure 2. Venn diagram showing FindCirc and CIRI circular RNAs numbers.

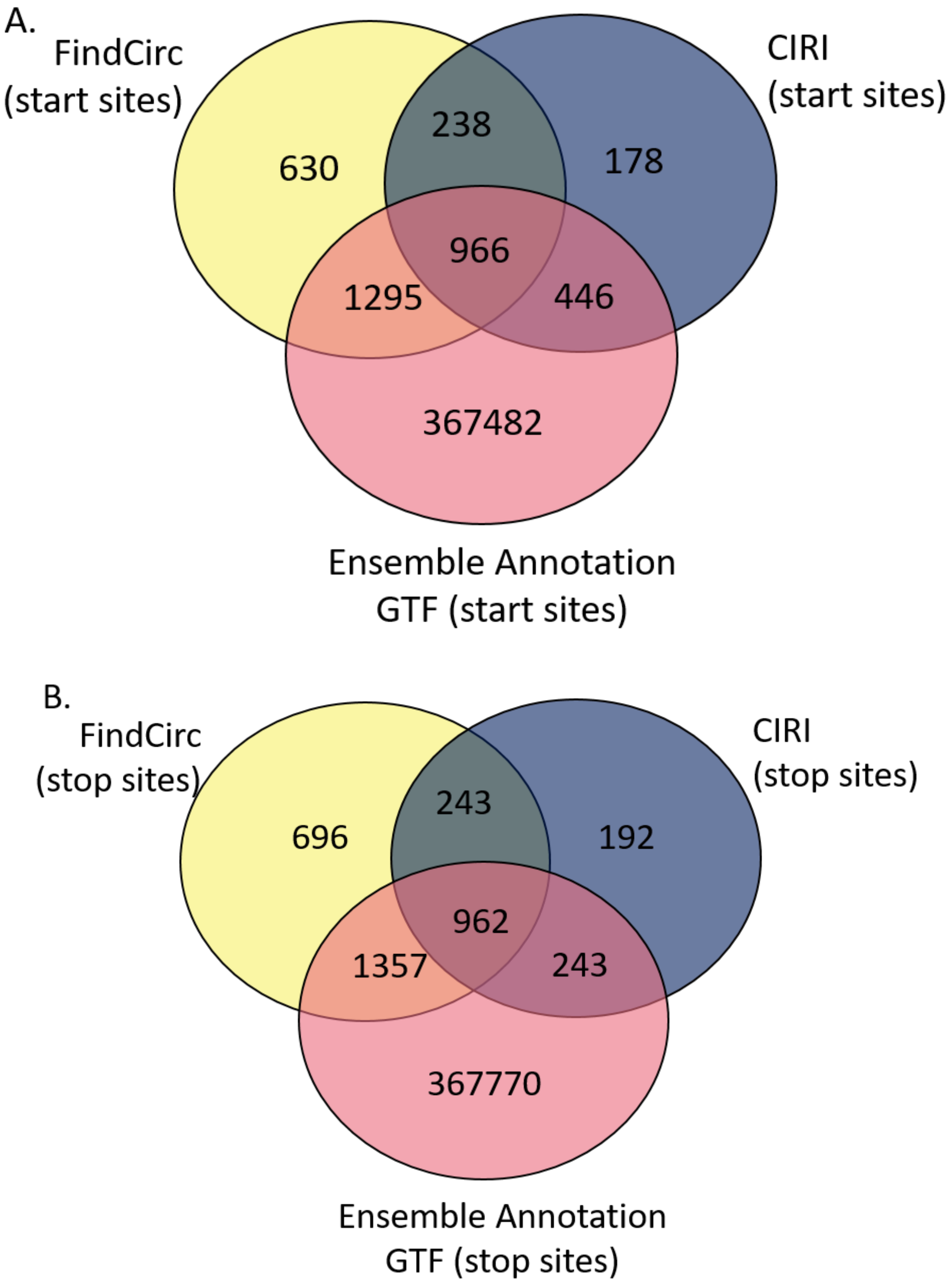

Supplementary Figure 3. Venn diagram showing overlap of annotated splice sites from Ensemble annotation file and identified splice sites from findcirc and CIRI pipelines.

Supplementary table1: List of PolyA RNA-seq datasets used for analysis with sample detail and total number of circular RNA identified.

Dataset	Sample	Total Circular RNAs
SRR891495	Heart	293
SRR891510	Muscle	228
SRR891511	Brain	178
SRR891512	Blood	363

Supplementary table 2: Divergent oligos for circular RNA			
**Tissue**	**Gene**	Primer	Sequence 5'-3'
**Blood**	***epb4.1***	epb4.1_circular_F	GAGCCCAGAGACATTGATGA
		epb4.1_circular_R	TCTGTTCTCTAGTGCTCACC
	hbaa1	hbaa1_circ_F	CAGGAGGGTGTTCCAGCAGA
		hbaa1_circ_R	ACTTCGGGAGVGGTCCTTAC
	ank1	ank1_circ_F	AGACACCACCGCAAAGTAGA
		ank1_circ_R	ATTGGCCCAGAAGGAGGTTT
	ikzf1	ikzf1_circ_F	GCATAGTTGCACAGGTGACA
		ikzf1_circ_R	AAGTGTGCATATTGCGGACG
	map2k5	map2k5_circ_F	AGACCAGCTCTGACAGCAGA
		map2k5_circ_R	AGCTCTCCAGCACTGAGAGT
**Brain**	***taf6***	taf6_circular_F	AAACTCTGATAAGGAGCCAG
		taf6_circular_R	TTCTCTGTACCACTCATGGT
	dab1	dab1_circ_F	GTACCTGATATATGCTCTCC
		dab1_circ_R	AGCCTAAACTGCTTTGACCT
	fam107b	fam107b_circ_F	GGTTTTCTGAGTGCTTCGAC
		fam107b_circ_R	CCTAGCAACCAGCAGTCAAA
	clstn1	clstn1_circ_F	CCACAGTCATAGGCAGTCAC
		clstn1_circ_R	AAAACCGAGCATCCGAGGAC
	sox9b	sox9b_circ_F	GTCTGGGCTGGTATTTGTAG
		sox9b_circ_R	AGAGTCTGAAGATGGAGAGC
**Muscle**	***srek***	srek_circ_F	TGCTGTCTGCCATCTTCACT
		srek_circ_R	TACCTGTTGCCATATGCCCA
	nexn	nexn_circ_F	GCTTCTTCTGCTCTTCCTGA
		nexn_circ_R	ATGGAGAAACAGAGGCAGGA
	tnnt2a	tnnt2a_circ_F	TCGCGAATTTACCCCACTGG
		tnnt2a_circ_R	TTCAGGAGAAGCGTCGTTCG
	ttnb1	ttnb1_circular_F	AGCTTCTGGTGAACTTGTC
		ttnb1_circular_R	AAGAAAGCCTCCAACCCAA
	ttnb2	ttnb2_circ_F	TAGTTCCTCTGTTTCTTCTG
		ttnb2_circ_R	GCAAATTTAAGCGTAGGACC
**Heart**	***rbm41***	rbm41_circ_F	CCCATGTGTTGGTGTAAACC
		rbm41_circ_R	AGCAATCGAGAGACAGAAGA
	zfn609a	zfn609a_circ_F	CATGGGAATGGTGATTCCTG
		zfn609a_circ_R	TCTGACCTTCTCAGACGACT
	furina	furina_circular_F	CATGGGAATGGTGATTCCTG
		furina_circular_R	CCTAATGTCACCAAGTCCGT
	eefl1al	eefl1al_circ_F	CCATGGGAATGATGGATCTC
		eefl1al_circ_R	TTTCACTCCCAGGGTGAAAG
**Gills**	***zp3***	zp3_circ_F	GCTGGCTGGATCAACTCTCC
		zp3_circ_R	GACCAACGCACAGGGAAGTT
	atp1a1a	atp1a1a_circ_F	TCTCCAGCTCTGAAGAAGGC
		atp1a1a_circ_R	TTCCTACCAAGTCAGTGCCC
	si:dkey-241l7.3	si:dkey-241l7.3_circ_F	CAGGACCGAAAACAGCGCAA
		si:dkey-241l7.3_circ_R	TGGTGTGAGTCGGTCTAACC
	si:dkey- 194m7.4	si:dkey- 194m7.4_circular_F	GCGTGACCTCAACAATCGTG
		si:dkey- 194m7.4_circular_R	GCCATCCTTGTGTCCACTCA
	si:ch211-146l10.7	si:ch211-146l10.7_circ_F	CTAGAGGTTCAGGTTGCACC
		si:ch211-146l10.7_circ_F	GCAAGTTTCTCCCTAGCCATG
**Convergent oligos for paternal genes of circular RNA**			
**Blood**	***epb4.1***	epb4.1_linear_F	TATTCTGATTGGTCGGCTGC
		epb4.1_linear_R	CTCCTCCAGCTCTTTACTCT
**Brain**	***taf6***	taf6_linear_F	CCACGTCCGACTTTGACAAT
		taf6_linear_R	CCTGCACTTGTTGCCATAAC
**Muscle**	***ttnb1***	ttnb1_linear_F	GAGGAAAGGCTGTTGAAGGA

**Muscle**	***ttnb1***	ttnb1_linear_R	CTTCTGGCGAAGCTCTGTTT
**Heart**	***furina***	furin_linear_F	CTGACGGACTTGGTGACATT
		furin_linear_R	CCCATGTGTTGGTGTAAACC
**Gills**	***si:dkey- 194m7.4***	si:dkey- 194m7.4_linear_F	GATGACCAGTGGGCAGAGAT
		si:dkey- 194m7.4_linear_R	TAGGCCATCTGCAACCAGGAC
**Oligos for tissue specific protein coding genes**			
**Blood**	***tal1***	tal1_F	CCACGCCACCGAGCTAAAAAA
		tal1_R	TGCACCATTCGAGTGTCGCTC
**Brain**	***mdka***	mdka_F	AGAAGAATAAGGGAGGTAAGG
		mdka_R	CTCCAAATTCTTTCTTCCAGT
**Muscle**	***tnnt2c***	tnnt2c_F	TGCGACACCGAAGAGTTTGTG
		tnnt2c_R	CTTGGGTTTGGGTTTGGTGTC
**Heart**	***vmhc***	vmhc_F	CCTATGGGCATCATGTCCATC
		vmhc_R	GGTCTTTGTTCTTCACCAGCC
**Gills**	***cx44.2***	cx44.2_F	CTCCGTCCTACATTTGCACC
		cx44.2_R	GGTGACAGATGGTGGACGTA
**Oligos for ubiquotous genes**			
**Ubiquotous**	***actb***	actb_F	TTTTGCTGGAGATGATGCCC
		actb_R	GGGTATTTGAGGGTCAGGAT

Supplementary table 3: Total unique circular RNA candidates with Read Coverage.

circChr	circStart	circStop	CircID	Score	circStrand	Blood	Brain	Heart	Muscle	Gills
chr10	11140240	11149265	dre-circ-2904	0	-	3	0	0	0	0
chr10	14901775	14925468	dre-circ-898	0	-	2	0	0	0	0
chr10	14992822	14998437	dre-circ-899	0	+	0	0	0	0	12
chr10	14997886	14998736	dre-circ-900	0	+	0	0	0	0	26
chr10	14997886	15001185	dre-circ-901	0	+	0	0	0	0	12
chr10	14997886	15004979	dre-circ-902	0	+	0	0	0	0	5
chr10	14997886	15018910	dre-circ-903	0	+	0	0	0	0	21
chr10	15000318	15004979	dre-circ-904	0	+	0	0	0	0	7
chr10	15004126	15004979	dre-circ-2905	0	+	0	0	0	0	9
chr10	15004126	15018910	dre-circ-905	0	+	0	0	0	0	6
chr10	15034736	15036255	dre-circ-906	0	+	0	0	0	0	2
chr10	1511576	1547359	dre-circ-2906	0	-	11	0	0	0	9
chr10	15255515	15260799	dre-circ-907	0	+	0	0	2	0	0
chr10	15540461	15553852	dre-circ-2907	0	+	3	0	0	0	0
chr10	16045927	16046265	dre-circ-2908	0	-	2	0	0	0	0
chr10	1694196	1694423	dre-circ-876	0	+	0	2	0	0	0
chr10	17493208	17538094	dre-circ-908	0	-	371	0	17	3	6
chr10	17497461	17540532	dre-circ-909	0	+	2	0	0	0	0
chr10	17499756	17540512	dre-circ-910	0	-	474	0	39	0	6
chr10	17503841	17541436	dre-circ-911	0	-	122	0	0	3	0
chr10	17506357	17544065	dre-circ-2909	0	-	637	0	38	6	10
chr10	17508267	17545725	dre-circ-912	0	-	3	0	0	0	0
chr10	17510902	17545965	dre-circ-913	0	-	712	0	41	0	7
chr10	17511108	17549094	dre-circ-914	0	-	379	0	29	6	5
chr10	17522211	17564141	dre-circ-915	0	-	0	0	2	0	0
chr10	17522444	17564962	dre-circ-916	0	+	0	0	2	0	0
chr10	1800922	1802120	dre-circ-2910	0	-	2	0	3	0	0
chr10	195444	197651	dre-circ-874	0	+	2	0	0	0	0
chr10	19598463	19603253	dre-circ-917	0	+	0	0	0	0	88
chr10	19599366	19619304	dre-circ-918	0	+	0	0	0	0	51
chr10	19599494	19599670	dre-circ-919	0	+	0	0	0	0	19
chr10	19604525	19605601	dre-circ-920	0	-	0	0	0	0	61
chr10	19604534	19605610	dre-circ-921	0	+	0	0	0	0	60
chr10	19615217	19617475	dre-circ-922	0	+	0	0	0	0	15
chr10	19616347	19616862	dre-circ-923	0	-	0	0	0	0	2
chr10	19616347	19617747	dre-circ-924	0	-	0	0	0	0	2
chr10	19618483	19625473	dre-circ-925	0	-	0	0	0	0	3
chr10	19625516	19626229	dre-circ-926	0	-	0	0	0	0	2
chr10	19625516	19626574	dre-circ-927	0	-	0	0	0	0	2
chr10	19625516	19626940	dre-circ-928	0	-	0	0	0	0	2
chr10	20121530	20122876	dre-circ-2911	0	+	6	0	0	0	0
chr10	20168681	20170333	dre-circ-2912	0	+	10	0	2	0	0
chr10	20171698	20178455	dre-circ-2913	0	+	2	0	0	0	0
chr10	20229964	20231323	dre-circ-929	0	+	0	0	0	0	2
chr10	208563	208867	dre-circ-2914	0	+	11	0	0	0	0
chr10	22024642	22030670	dre-circ-2915	0	+	0	2	0	0	0
chr10	22134703	22146664	dre-circ-930	0	-	0	0	2	0	0
chr10	22138016	22150811	dre-circ-931	0	-	0	0	2	0	0
chr10	22291654	22298616	dre-circ-932	0	-	0	0	0	0	17
chr10	22718160	22718553	dre-circ-2916	0	+	5	0	0	0	0
chr10	22943543	22943852	dre-circ-2917	0	+	3	0	0	0	0
chr10	2362134	2362290	dre-circ-877	0	+	0	2	0	0	0
chr10	23773037	23778381	dre-circ-2918	0	+	0	0	0	3	0
chr10	24560654	24578472	dre-circ-933	0	+	0	0	0	0	2
chr10	24899994	24902502	dre-circ-2919	0	+	0	0	0	3	0
chr10	26214104	26219752	dre-circ-934	0	-	2	0	0	0	0
chr10	26429665	26430759	dre-circ-2920	0	-	0	0	2	0	0
chr10	27175774	27226560	dre-circ-935	0	+	6	21	0	0	2
chr10	27532026	27537811	dre-circ-936	0	+	0	0	2	0	0
chr10	2760052	2761751	dre-circ-878	0	+	2	0	0	0	0
chr10	28933515	28937747	dre-circ-937	0	-	2	0	0	0	0
chr10	28933529	28958636	dre-circ-938	0	-	0	2	0	0	0
chr10	29030879	29032793	dre-circ-939	0	-	0	0	2	0	0
chr10	2929765	2934656	dre-circ-879	0	+	2	0	0	0	0
chr10	29350837	29360856	dre-circ-940	0	+	0	0	0	2	0
chr10	30616750	30624192	dre-circ-941	0	-	0	0	2	0	0
chr10	32043776	32044422	dre-circ-2921	0	-	0	0	2	3	0
chr10	33338270	33338435	dre-circ-942	0	-	2	0	0	0	0
chr10	3388335	3389332	dre-circ-2922	0	-	3	0	0	0	0
chr10	34218828	34289021	dre-circ-943	0	-	0	0	2	0	0
chr10	34492837	34494944	dre-circ-2923	0	+	0	0	0	3	0
chr10	34972056	34974535	dre-circ-944	0	+	0	0	2	0	0
chr10	36475109	36475625	dre-circ-945	0	+	0	0	3	0	0
chr10	36529999	36532101	dre-circ-946	0	+	0	0	2	0	0
chr10	37764896	37765039	dre-circ-947	0	-	0	0	0	0	2
chr10	38716170	38725814	dre-circ-2924	0	+	0	0	4	0	0
chr10	39520620	39535347	dre-circ-948	0	-	2	0	0	0	0
chr10	39602143	39602496	dre-circ-2925	0	-	2	0	0	0	0
chr10	4007206	4012121	dre-circ-880	0	+	0	2	0	0	0
chr10	42478785	42482305	dre-circ-2926	0	-	2	0	3	3	0
chr10	43239962	43244654	dre-circ-949	0	-	0	2	0	0	0
chr10	433087	435786	dre-circ-2927	0	+	0	0	2	0	0
chr10	43466158	43466720	dre-circ-950	0	-	2	0	0	0	0
chr10	43469364	43470713	dre-circ-951	0	-	2	0	0	0	0
chr10	43527663	43542341	dre-circ-952	0	+	0	0	0	0	3

chr10	43602108	43602429	dre-circ-953	0	-	2	0	0	0	0
chr10	43631154	43632348	dre-circ-954	0	-	0	0	2	0	0
chr10	44253001	44260384	dre-circ-2928	0	-	0	0	0	0	4
chr10	45620052	45621180	dre-circ-955	0	-	0	0	0	0	13
chr10	45861227	45879431	dre-circ-2929	0	+	2	0	0	0	0
chr10	46039070	46039291	dre-circ-2930	0	-	7	0	0	0	0
chr10	46118471	46118638	dre-circ-956	0	-	2	0	0	0	0
chr10	46154263	46156675	dre-circ-2931	0	-	3	0	0	0	0
chr10	46507852	46508573	dre-circ-2932	0	-	3	0	0	0	0
chr10	5969087	5984626	dre-circ-2933	0	-	0	0	2	0	0
chr10	60099	60631	dre-circ-873	0	-	3	0	0	0	2
chr10	6131874	6133989	dre-circ-881	0	-	0	0	2	0	0
chr10	6249309	6250809	dre-circ-882	0	+	0	0	0	0	2
chr10	66259	114706	dre-circ-2934	0	-	0	0	3	0	6
chr10	6737819	6826701	dre-circ-883	0	+	2	0	0	0	0
chr10	6885588	6886001	dre-circ-2935	0	-	0	2	0	0	0
chr10	7073995	7080621	dre-circ-884	0	+	0	0	0	0	44
chr10	7073995	7087829	dre-circ-885	0	+	0	0	0	0	34
chr10	7073995	7106601	dre-circ-886	0	+	0	0	0	0	23
chr10	7074176	7088013	dre-circ-887	0	-	0	0	0	0	7
chr10	7074599	7081191	dre-circ-888	0	-	0	0	0	0	32
chr10	7075204	7081723	dre-circ-889	0	-	0	0	0	0	119
chr10	7088223	7107301	dre-circ-890	0	-	0	0	0	0	2
chr10	7297129	7312041	dre-circ-2936	0	-	0	0	0	2	0
chr10	7779516	7779722	dre-circ-891	0	-	5	0	0	2	2
chr10	8160818	8165129	dre-circ-892	0	+	0	0	0	0	2
chr10	8372116	8376059	dre-circ-2937	0	+	3	0	0	0	0
chr10	8382330	8385902	dre-circ-893	0	+	2	0	0	0	0
chr10	8900131	8903278	dre-circ-2938	0	+	0	0	0	2	0
chr10	8948531	8953249	dre-circ-2939	0	+	0	0	0	0	3
chr10	8951443	9010597	dre-circ-894	0	+	0	0	0	0	6
chr10	8951671	9010814	dre-circ-895	0	+	0	0	0	0	2
chr10	9042427	9043140	dre-circ-2940	0	-	3	0	0	0	0
chr10	9127856	9139334	dre-circ-2941	0	-	2	0	0	0	0
chr10	940055	944006	dre-circ-875	0	+	0	0	0	0	2
chr10	9451492	9452430	dre-circ-896	0	+	2	0	0	0	0
chr10	9691999	9695291	dre-circ-897	0	+	0	0	0	2	0
chr1	10547888	10556007	dre-circ-28	0	-	0	0	0	0	2
chr1	10844764	10845317	dre-circ-2942	0	+	3	0	0	0	0
chr1	11049377	11049681	dre-circ-2943	0	-	2	0	0	0	0
chr11	10555858	10565369	dre-circ-970	0	-	0	2	0	0	0
chr11	11330969	11331357	dre-circ-2944	0	+	0	0	6	0	0
chr11	11460270	11464743	dre-circ-971	0	-	0	0	0	0	19
chr11	11460270	11491896	dre-circ-972	0	-	0	0	0	0	10
chr11	11460270	11526059	dre-circ-973	0	-	0	0	0	0	9
chr11	11460270	11548343	dre-circ-974	0	-	0	0	0	0	12
chr11	11460270	11559356	dre-circ-975	0	-	0	0	0	0	14
chr11	11954720	12004014	dre-circ-976	0	-	0	0	3	0	14
chr11	12304769	12305366	dre-circ-2945	0	+	0	0	3	0	0
chr11	13398336	13398456	dre-circ-977	0	+	0	0	2	0	0
chr11	135098	138219	dre-circ-2946	0	-	3	0	2	7	2
chr11	13580398	13587011	dre-circ-978	0	+	0	0	2	0	0
chr11	13580398	13598762	dre-circ-979	0	+	2	0	0	0	0
chr11	13665647	13680194	dre-circ-980	0	+	0	0	0	0	2
chr11	13679027	13680194	dre-circ-2947	0	+	0	0	0	2	2
chr11	14941989	14942566	dre-circ-2948	0	+	12	0	5	0	0
chr1	11541954	11543990	dre-circ-2949	0	+	0	0	3	5	0
chr11	15881993	15929985	dre-circ-981	0	+	0	0	0	2	0
chr1	116221	116328	dre-circ-3	0	+	2	0	0	0	0
chr11	1682960	1685953	dre-circ-958	0	+	0	0	2	0	0
chr11	1739229	1740100	dre-circ-2950	0	+	0	2	0	0	2
chr11	18471270	18471637	dre-circ-982	0	-	0	0	2	0	0
chr11	18537933	18553440	dre-circ-983	0	+	0	0	0	0	93
chr11	18652237	18656058	dre-circ-984	0	+	0	0	0	0	4
chr11	18652825	18664835	dre-circ-985	0	-	0	0	0	0	29
chr11	19496680	19505053	dre-circ-2951	0	+	0	0	0	0	2
chr11	20174077	20186393	dre-circ-986	0	-	0	2	0	0	0
chr11	20406497	20438174	dre-circ-987	0	+	0	0	0	0	2
chr11	2073425	2076064	dre-circ-2952	0	-	0	0	0	2	0
chr11	21578157	21578384	dre-circ-988	0	+	0	0	3	0	0
chr11	21578157	21578840	dre-circ-989	0	+	9	0	0	0	25
chr11	21578157	21579068	dre-circ-990	0	+	0	0	0	0	7
chr11	21991125	21993134	dre-circ-2953	0	-	0	0	0	0	2
chr11	22214403	22219741	dre-circ-991	0	-	0	0	0	2	0
chr11	22219301	22219741	dre-circ-992	0	-	0	0	2	0	0
chr11	2264641	2273815	dre-circ-959	0	-	0	2	0	0	0
chr11	23128534	23128859	dre-circ-2954	0	-	0	0	2	0	0
chr11	23328285	23338316	dre-circ-993	0	+	0	0	2	0	0
chr11	2427215	2432085	dre-circ-2955	0	-	0	0	10	0	0
chr11	24299391	24309605	dre-circ-2956	0	-	0	0	0	0	2
chr11	24356088	24356888	dre-circ-2957	0	-	0	0	3	0	0
chr11	24786157	24792780	dre-circ-994	0	+	0	2	0	0	0
chr11	2487474	2491431	dre-circ-2958	0	+	3	0	0	0	0
chr11	25276648	25277017	dre-circ-995	0	+	2	0	0	0	0
chr11	25312865	25314090	dre-circ-2959	0	-	0	3	0	0	0
chr11	26119848	26120513	dre-circ-2960	0	-	0	0	2	0	0
chr11	26220213	26220774	dre-circ-2961	0	+	0	0	0	0	2

chr1	12627949	12656054	dre-circ-29	0	-	0	0	0	2	0
chr11	26350751	26352388	dre-circ-996	0	-	2	0	0	0	0
chr11	26412405	26415592	dre-circ-997	0	+	2	0	0	0	0
chr11	26494429	26494536	dre-circ-998	0	+	0	0	2	0	0
chr11	26494456	26494563	dre-circ-999	0	+	0	0	0	0	2
chr11	2654277	2654429	dre-circ-960	0	+	2	0	0	0	0
chr11	26628964	26633075	dre-circ-2962	0	+	0	2	0	0	0
chr11	269279	282197	dre-circ-957	0	-	3	0	2	0	0
chr11	27457321	27457930	dre-circ-1000	0	+	0	2	0	0	0
chr11	2787671	2788788	dre-circ-2963	0	+	3	0	0	0	0
chr11	28801898	28803811	dre-circ-1001	0	-	0	0	0	2	0
chr11	2902295	2909142	dre-circ-961	0	+	0	0	2	0	0
chr11	30637282	30641506	dre-circ-2964	0	-	0	0	4	0	3
chr11	30667501	30668572	dre-circ-1002	0	+	2	0	0	0	0
chr11	30991933	30994482	dre-circ-1003	0	-	2	0	0	0	0
chr11	31312549	31320425	dre-circ-2965	0	-	0	0	18	0	2
chr11	31420528	31425688	dre-circ-2966	0	-	0	0	6	0	0
chr11	31420528	31438547	dre-circ-2967	0	-	0	0	57	0	0
chr11	31425561	31434260	dre-circ-1004	0	-	0	0	11	0	0
chr11	31425561	31438547	dre-circ-2968	0	-	0	0	47	0	0
chr11	31475039	31476893	dre-circ-2969	0	-	0	0	3	0	0
chr11	31504975	31508283	dre-circ-1005	0	-	6	0	0	0	65
chr11	31505957	31508538	dre-circ-1006	0	-	2	0	0	0	64
chr11	32118744	32125487	dre-circ-1007	0	+	0	0	2	0	0
chr11	3226933	3229072	dre-circ-962	0	+	0	0	0	0	5
chr1	13359476	13361152	dre-circ-2970	0	+	0	0	0	3	0
chr11	3369198	3452850	dre-circ-963	0	+	0	3	2	0	2
chr1	13386164	13412877	dre-circ-30	0	-	0	0	0	0	3
chr11	3388137	3388760	dre-circ-2971	0	+	0	5	0	0	0
chr11	37206138	37208602	dre-circ-2972	0	+	2	0	0	0	4
chr11	37283812	37284235	dre-circ-1008	0	-	0	0	0	0	2
chr11	38124313	38124625	dre-circ-2973	0	+	2	0	0	3	3
chr11	4006677	4008710	dre-circ-964	0	-	0	0	2	0	0
chr11	40324162	40326672	dre-circ-2974	0	-	2	2	2	5	0
chr11	40368695	40372716	dre-circ-2975	0	+	0	0	0	0	2
chr11	40405355	40408325	dre-circ-1009	0	+	0	0	0	2	0
chr11	41513560	41513731	dre-circ-1010	0	-	0	0	2	0	0
chr11	41543463	41548285	dre-circ-2976	0	+	0	0	2	0	0
chr11	41543463	41548290	dre-circ-2977	0	+	0	0	6	0	2
chr11	41629200	41630307	dre-circ-2978	0	+	0	0	3	0	0
chr11	41629203	41637174	dre-circ-2979	0	+	0	0	0	2	0
chr11	41639197	41644435	dre-circ-2980	0	+	0	0	2	0	0
chr11	41733299	41826895	dre-circ-1011	0	-	0	0	0	0	10
chr1	14185460	14186413	dre-circ-2981	0	-	0	0	0	2	2
chr11	41917042	41917358	dre-circ-2982	0	-	0	0	2	0	0
chr11	42055259	42055703	dre-circ-2983	0	+	0	0	2	0	0
chr11	43566460	43575070	dre-circ-1012	0	+	10	0	9	0	0
chr11	43618361	43619144	dre-circ-1013	0	-	0	2	0	0	0
chr11	43669861	43671901	dre-circ-2984	0	+	0	0	0	2	0
chr11	43890125	43896875	dre-circ-1014	0	+	2	0	0	4	5
chr11	44248477	44318739	dre-circ-2985	0	+	9	49	69	25	22
chr11	44248480	44318739	dre-circ-2986	0	+	0	11	15	4	5
chr11	44622744	44634495	dre-circ-2987	0	-	5	0	2	0	0
chr11	44624723	44634495	dre-circ-2988	0	-	2	0	0	0	0
chr11	44629761	44634495	dre-circ-2989	0	-	6	0	0	0	0
chr11	44629761	44655827	dre-circ-1015	0	-	2	0	0	0	0
chr1	1455704	1475317	dre-circ-8	0	+	0	0	0	43	0
chr1	1458097	1480060	dre-circ-9	0	+	2	0	0	47	0
chr1	1458097	1552002	dre-circ-10	0	+	0	0	0	15	0
chr11	46055933	46058686	dre-circ-2990	0	-	3	0	0	0	0
chr1	1475399	1549679	dre-circ-11	0	+	3	0	0	0	0
chr1	1478788	1568269	dre-circ-12	0	+	3	2	0	0	0
chr1	1479892	1551748	dre-circ-2991	0	+	0	0	0	0	12
chr1	1480140	1552002	dre-circ-13	0	+	9	5	19	4	10
chr1	1480140	1568975	dre-circ-14	0	+	29	13	58	5	23
chr1	148498	151782	dre-circ-4	0	+	0	0	0	0	21
chr1	1551052	1568005	dre-circ-15	0	+	4	0	0	0	13
chr1	1551052	1602122	dre-circ-16	0	+	0	0	0	0	9
chr1	1552081	1603092	dre-circ-17	0	+	0	0	0	0	33
chr1	1552313	1569208	dre-circ-18	0	+	0	0	0	0	3
chr11	5528182	5530338	dre-circ-965	0	+	0	2	0	0	0
chr11	5579535	5580813	dre-circ-2992	0	+	2	0	0	0	0
chr1	156278	156469	dre-circ-5	0	+	0	0	0	0	3
chr1	1563544	1595276	dre-circ-19	0	+	15	0	0	3	39
chr1	1568094	1602122	dre-circ-2993	0	+	0	0	0	0	4
chr1	1569054	1603092	dre-circ-2994	0	+	9	0	0	0	18
chr1	1569293	1603322	dre-circ-20	0	+	0	0	0	0	8
chr1	1569499	1603533	dre-circ-21	0	+	0	0	0	0	3
chr1	1569742	1603754	dre-circ-22	0	+	2	0	0	0	5
chr11	5892218	5893901	dre-circ-2995	0	-	2	0	0	0	0
chr1	16501794	16512429	dre-circ-2996	0	-	0	0	3	0	0
chr11	6539836	6539960	dre-circ-966	0	+	0	2	0	0	0
chr1	1654583	1661319	dre-circ-2997	0	+	0	0	0	0	9
chr1	1663485	1678645	dre-circ-23	0	-	0	0	0	0	3
chr11	7053639	7063119	dre-circ-967	0	-	2	0	0	0	0
chr11	7112846	7113415	dre-circ-968	0	-	0	0	3	0	2
chr11	7534717	7539338	dre-circ-2998	0	-	0	0	0	0	2

chr1	17739380	17741015	dre-circ-31	0	+	0	2	0	2	0
chr1	17739380	17751693	dre-circ-32	0	+	0	0	0	2	0
chr1	18346452	18346794	dre-circ-33	0	+	2	0	0	0	0
chr11	8779886	8784789	dre-circ-969	0	-	0	0	0	0	2
chr1	1885755	1886417	dre-circ-24	0	+	0	0	3	0	0
chr11	9049625	9129009	dre-circ-2999	0	-	0	2	2	0	0
chr1	19652203	19655654	dre-circ-3000	0	-	0	0	0	0	2
chr12	11513741	11514051	dre-circ-1035	0	-	2	0	0	0	0
chr12	1246351	1247401	dre-circ-3001	0	-	2	0	0	0	0
chr12	12642717	12644230	dre-circ-1036	0	-	2	0	0	0	0
chr12	14082914	14086632	dre-circ-1037	0	-	0	0	0	0	2
chr12	14697634	14721748	dre-circ-1038	0	-	0	0	0	0	2
chr12	14721387	14721533	dre-circ-1039	0	-	2	0	0	0	0
chr12	14882366	14883154	dre-circ-1040	0	+	0	0	0	2	0
chr12	15050914	15051334	dre-circ-3002	0	+	0	2	2	0	7
chr12	15193434	15197664	dre-circ-3003	0	+	4	0	0	0	0
chr12	16514288	16514406	dre-circ-1041	0	+	3	0	0	0	0
chr12	16514288	16516418	dre-circ-3004	0	+	3	0	0	0	0
chr12	16514288	16516876	dre-circ-3005	0	+	2	0	0	0	0
chr12	16514288	16522808	dre-circ-3006	0	+	3	0	0	0	0
chr12	18240160	18240666	dre-circ-3007	0	-	0	0	2	0	0
chr1	2186341	2190351	dre-circ-3008	0	+	0	0	0	0	3
chr12	18666415	18669137	dre-circ-1042	0	-	2	0	0	0	0
chr12	1867718	1905611	dre-circ-3009	0	+	4	0	3	0	0
chr12	18774038	18778978	dre-circ-3010	0	-	0	0	0	633	0
chr12	20072076	20072427	dre-circ-3011	0	+	3	0	0	0	0
chr12	20244945	20245742	dre-circ-3012	0	-	2	0	0	0	0
chr12	20540365	20543362	dre-circ-3013	0	+	3	0	0	0	0
chr12	206966	210971	dre-circ-3014	0	+	0	0	2	0	0
chr12	22125113	22125495	dre-circ-1043	0	+	0	0	0	2	0
chr1	22309656	22314833	dre-circ-34	0	-	0	2	0	0	0
chr12	23724936	23735040	dre-circ-1044	0	-	0	0	2	0	0
chr12	24219633	24220013	dre-circ-3015	0	+	0	0	2	0	0
chr12	2467022	2467147	dre-circ-1017	0	+	0	0	2	0	0
chr12	24802031	24812216	dre-circ-1045	0	+	0	0	0	0	2
chr12	25315405	25347872	dre-circ-3016	0	+	2	0	0	0	0
chr12	25330970	25331961	dre-circ-1046	0	+	0	0	2	0	0
chr12	25555979	25556367	dre-circ-3017	0	+	18	0	4	0	0
chr12	27388582	27432532	dre-circ-1047	0	+	0	0	29	0	0
chr12	27394094	27434137	dre-circ-1048	0	+	0	6	33	0	0
chr12	27400876	27445532	dre-circ-1049	0	+	0	0	40	22	0
chr12	2779097	2782995	dre-circ-1018	0	-	2	0	0	0	0
chr12	2841796	2848593	dre-circ-1019	0	+	0	0	2	2	0
chr12	285072	285610	dre-circ-1016	0	-	2	0	0	0	0
chr12	28646270	28647111	dre-circ-3018	0	+	2	0	0	0	0
chr12	289587	290255	dre-circ-3019	0	-	5	0	6	0	0
chr12	29600011	29608866	dre-circ-1050	0	+	0	0	2	0	0
chr12	29600011	29613461	dre-circ-3020	0	+	0	0	2	0	0
chr12	29600011	29620128	dre-circ-3021	0	+	0	0	2	0	0
chr12	29632146	29632592	dre-circ-3022	0	+	0	0	2	0	0
chr12	29881440	29888311	dre-circ-3023	0	-	0	0	0	0	2
chr12	30425956	30426595	dre-circ-3024	0	-	0	2	0	0	0
chr12	30629994	30636223	dre-circ-1051	0	+	0	2	0	0	0
chr12	3127570	3127704	dre-circ-1020	0	-	0	0	0	0	2
chr12	31738441	31738827	dre-circ-3025	0	+	0	0	4	0	0
chr12	31866907	31872100	dre-circ-1052	0	-	0	0	0	0	2
chr12	32246573	32246683	dre-circ-1053	0	-	0	0	0	0	2
chr12	32457948	32480561	dre-circ-1054	0	+	0	0	0	2	0
chr12	32461486	32484147	dre-circ-1055	0	+	0	0	0	0	41
chr12	32687032	32691225	dre-circ-1056	0	-	2	0	0	0	0
chr12	33381742	33384134	dre-circ-1057	0	+	0	0	0	0	2
chr12	35729358	35729547	dre-circ-3026	0	-	7	0	2	0	0
chr12	3602124	3604751	dre-circ-1021	0	+	0	0	2	0	0
chr12	36463861	36464982	dre-circ-3027	0	-	5	0	0	0	0
chr12	36509506	36535720	dre-circ-1058	0	-	0	0	2	0	2
chr12	37323575	37326559	dre-circ-3028	0	-	0	0	0	0	2
chr12	37513023	37517972	dre-circ-3029	0	-	2	0	4	2	3
chr12	3798721	3887907	dre-circ-3030	0	-	0	0	2	0	0
chr12	38353300	38353542	dre-circ-3031	0	+	0	0	3	0	2
chr12	40369593	40371334	dre-circ-3032	0	+	9	0	0	0	0
chr12	40441315	40500049	dre-circ-1059	0	+	0	0	0	0	42
chr12	40441315	40507405	dre-circ-3033	0	+	0	0	0	0	18
chr12	40444653	40510843	dre-circ-1060	0	+	0	0	0	0	11
chr12	40476466	40524894	dre-circ-3034	0	+	0	0	0	0	12
chr12	40491882	40538160	dre-circ-1061	0	+	2	0	0	0	23
chr12	40491882	40541232	dre-circ-1062	0	+	2	0	0	0	16
chr12	40492796	40496587	dre-circ-1063	0	+	0	0	0	0	10
chr12	40493295	40551531	dre-circ-1064	0	+	0	0	0	0	16
chr12	40495144	40561182	dre-circ-1065	0	+	0	0	0	0	2
chr12	40496714	40563305	dre-circ-1066	0	+	4	0	0	0	51
chr12	40497554	40564132	dre-circ-1067	0	+	2	0	0	0	22
chr12	40498607	40566722	dre-circ-1068	0	+	3	0	2	0	55
chr12	40500199	40507405	dre-circ-1069	0	+	0	0	0	0	37
chr12	40500890	40508099	dre-circ-1070	0	+	0	0	0	0	15
chr12	40514872	40569657	dre-circ-1071	0	+	6	0	0	0	35
chr12	40518289	40574686	dre-circ-1072	0	+	0	0	0	0	15
chr12	40538652	40541232	dre-circ-1073	0	+	0	0	0	0	4

chr12	4183598	4189439	dre-circ-3035	0	-	0	0	3	0	0
chr12	4205648	4205821	dre-circ-3036	0	-	3	0	0	0	0
chr12	4246352	4247947	dre-circ-3037	0	+	3	0	0	0	0
chr12	43382577	43386696	dre-circ-3038	0	-	4	4	3	2	2
chr12	43389952	43403869	dre-circ-3039	0	-	4	0	0	0	0
chr12	4397958	4490149	dre-circ-1022	0	-	2	0	0	0	6
chr12	45246871	45251774	dre-circ-3040	0	-	0	0	0	2	0
chr12	46617105	46617607	dre-circ-3041	0	-	2	0	0	0	0
chr12	47052629	47145747	dre-circ-1074	0	-	0	0	0	0	2
chr12	47192536	47241558	dre-circ-1075	0	-	0	0	0	0	3
chr12	47192536	47278200	dre-circ-1076	0	-	0	0	0	0	4
chr12	47193344	47258296	dre-circ-1077	0	-	0	0	0	0	14
chr12	47193344	47278983	dre-circ-1078	0	-	0	0	0	0	21
chr12	47193344	47288283	dre-circ-1079	0	-	0	0	0	0	17
chr12	47198646	47262651	dre-circ-1080	0	-	0	0	0	0	6
chr12	47200703	47264072	dre-circ-1081	0	-	0	0	0	0	3
chr12	47200703	47284372	dre-circ-1082	0	-	0	0	0	0	7
chr12	47333580	47339875	dre-circ-1083	0	-	0	0	0	0	6
chr1	24743837	24746774	dre-circ-3042	0	-	0	0	0	5	0
chr12	47934975	47956392	dre-circ-1084	0	+	0	4	0	0	8
chr12	48016566	48021113	dre-circ-3043	0	+	3	0	0	0	0
chr12	48016566	48032291	dre-circ-3044	0	+	5	0	0	0	0
chr12	48016566	48038865	dre-circ-3045	0	+	2	0	0	0	0
chr12	48076742	48140870	dre-circ-1085	0	+	6	0	4	0	0
chr12	48086540	48151709	dre-circ-1086	0	+	3	5	7	20	2
chr12	4813463	4836913	dre-circ-1023	0	+	0	0	7	0	5
chr12	4813742	4837133	dre-circ-3046	0	+	3	0	4	0	5
chr12	4814484	4837666	dre-circ-1024	0	+	2	2	10	0	8
chr12	48165645	48166134	dre-circ-3047	0	+	3	0	0	0	0
chr12	4818167	4842114	dre-circ-3048	0	+	0	0	5	0	0
chr12	4821221	4844186	dre-circ-1025	0	+	0	0	0	4	0
chr12	4822221	4848263	dre-circ-3049	0	+	0	0	0	3	0
chr12	4823912	4848455	dre-circ-1026	0	+	4	0	6	8	5
chr12	48264443	48283741	dre-circ-1087	0	-	0	2	0	0	0
chr12	48857446	48880788	dre-circ-1088	0	-	0	0	0	13	0
chr12	48861335	48884128	dre-circ-1089	0	-	10	2	73	38	25
chr12	48935249	48935829	dre-circ-1090	0	-	0	2	0	0	0
chr12	49028273	49033428	dre-circ-1091	0	-	2	0	3	0	0
chr12	49373268	49379974	dre-circ-3050	0	-	0	0	0	0	2
chr12	4978557	5001480	dre-circ-1027	0	-	0	0	0	0	8
chr12	4979795	4980187	dre-circ-1028	0	-	0	0	0	0	2
chr12	4993342	5007059	dre-circ-3051	0	-	4	0	0	0	22
chr12	4994042	5007247	dre-circ-3052	0	-	3	0	0	0	9
chr12	49947051	49947327	dre-circ-3053	0	+	4	0	0	4	0
chr12	49947051	50044956	dre-circ-1092	0	+	2	0	3	22	0
chr12	5003528	5019764	dre-circ-1029	0	-	0	0	0	0	53
chr12	50189993	50190882	dre-circ-3054	0	-	3	0	0	0	0
chr12	50392443	50480742	dre-circ-3055	0	-	0	0	0	5	0
chr12	5163582	5165639	dre-circ-1030	0	-	0	0	0	0	2
chr12	5266247	5267071	dre-circ-3056	0	-	2	0	0	0	0
chr12	5269282	5269650	dre-circ-1031	0	-	2	0	0	0	0
chr12	5269282	5288998	dre-circ-3057	0	-	2	0	0	0	0
chr1	25380953	25474680	dre-circ-35	0	+	0	0	0	6	0
chr1	25381214	25475176	dre-circ-36	0	+	10	4	0	0	24
chr1	25387544	25478067	dre-circ-37	0	+	0	0	0	3	0
chr1	25626963	25628884	dre-circ-38	0	+	0	0	2	0	0
chr12	6226966	6227335	dre-circ-3058	0	-	0	0	0	0	2
chr1	26794590	26795743	dre-circ-3059	0	+	0	0	2	0	0
chr12	7075009	7094967	dre-circ-1032	0	+	0	0	2	0	0
chr12	7075009	7105110	dre-circ-1033	0	+	0	2	0	0	0
chr1	27205361	27223825	dre-circ-3060	0	+	0	0	0	0	2
chr1	27482155	27488016	dre-circ-39	0	-	0	0	0	0	2
chr1	27482660	27488891	dre-circ-3061	0	-	0	0	2	0	0
chr1	27487902	27488891	dre-circ-3062	0	-	3	2	3	0	0
chr1	27718742	27740044	dre-circ-3063	0	-	0	0	2	0	0
chr1	27737431	27740044	dre-circ-3064	0	-	3	13	19	8	10
chr1	27737431	27741348	dre-circ-3065	0	-	0	0	3	0	0
chr12	8361730	8383177	dre-circ-3066	0	-	0	0	2	0	0
chr12	8372233	8383177	dre-circ-3067	0	-	0	0	2	0	0
chr1	29025837	29076719	dre-circ-40	0	+	0	0	0	0	6
chr12	9339752	9345640	dre-circ-3068	0	-	0	0	0	0	2
chr12	941294	945837	dre-circ-3069	0	+	3	0	0	0	3
chr1	296439	296829	dre-circ-3070	0	+	2	0	0	0	0
chr1	29781822	29782240	dre-circ-41	0	+	0	0	2	0	0
chr12	9852088	9853358	dre-circ-3071	0	-	9	0	0	0	0
chr12	9858642	9861881	dre-circ-1034	0	-	2	0	0	0	0
chr1	30772335	30773090	dre-circ-42	0	+	0	0	2	0	0
chr13	104560	108162	dre-circ-1093	0	-	2	0	0	0	0
chr13	10655931	10656389	dre-circ-3072	0	+	14	0	0	0	2
chr13	1077854	1078275	dre-circ-1095	0	-	0	0	0	0	2
chr13	11322922	11326129	dre-circ-1109	0	-	0	2	0	0	0
chr13	11481730	11490650	dre-circ-3073	0	-	0	0	3	0	0
chr13	1266763	1267536	dre-circ-3074	0	-	0	0	2	0	0
chr13	12793867	12795546	dre-circ-1110	0	+	0	0	2	0	0
chr13	12801388	12815428	dre-circ-3075	0	+	0	0	0	0	3
chr13	1286658	1288659	dre-circ-3076	0	-	0	0	2	2	0
chr13	13611633	13621426	dre-circ-1111	0	+	0	0	0	0	2

chr13	1413823	1415178	dre-circ-3077	0	+	7	49	38	26	29
chr13	15572999	15574407	dre-circ-3078	0	+	0	0	4	0	0
chr13	15608577	15612125	dre-circ-1112	0	+	2	0	0	0	0
chr13	15666877	15679436	dre-circ-3079	0	+	0	0	0	0	2
chr13	15717329	15717780	dre-circ-3080	0	-	3	0	0	0	0
chr13	15729968	15732313	dre-circ-1113	0	-	2	0	0	0	0
chr13	15792126	15796829	dre-circ-3081	0	+	0	0	0	0	2
chr13	15796489	15836927	dre-circ-1114	0	+	0	0	0	0	2
chr13	15919625	15920020	dre-circ-3082	0	-	5	0	0	0	2
chr13	16287756	16304475	dre-circ-1115	0	-	0	0	0	2	0
chr13	16454929	16459319	dre-circ-1116	0	+	0	0	0	2	0
chr13	1760960	1765495	dre-circ-1096	0	+	0	4	0	0	0
chr13	18216516	18216877	dre-circ-3083	0	-	3	0	0	0	0
chr13	18323730	18326125	dre-circ-3084	0	+	3	0	0	3	0
chr13	18335538	18341317	dre-circ-1117	0	+	0	0	0	0	2
chr13	18443681	18446466	dre-circ-3085	0	+	4	0	0	0	0
chr13	19354110	19354294	dre-circ-1118	0	+	3	0	0	0	0
chr13	19686937	19768052	dre-circ-1119	0	-	0	0	0	0	6
chr13	1976046	1978932	dre-circ-3086	0	-	0	0	2	0	0
chr13	1978411	1978572	dre-circ-1097	0	-	0	0	2	0	0
chr13	20246989	20307418	dre-circ-1120	0	+	0	0	0	2	0
chr13	2104006	2108727	dre-circ-3087	0	-	0	0	0	0	5
chr13	2129068	2129356	dre-circ-1098	0	-	0	0	2	0	0
chr13	22346766	22375355	dre-circ-1121	0	+	2	0	0	0	0
chr13	22984482	22990260	dre-circ-1122	0	-	0	0	0	2	0
chr13	23126135	23165178	dre-circ-1123	0	-	0	0	8	0	0
chr13	2325867	2338382	dre-circ-1099	0	+	0	0	0	2	0
chr13	23309356	23380172	dre-circ-1124	0	+	0	0	55	0	0
chr13	23309359	23380172	dre-circ-1125	0	+	0	0	0	4	0
chr13	23313514	23394161	dre-circ-1126	0	+	0	0	14	2	0
chr13	2334605	2339941	dre-circ-3088	0	+	2	0	0	0	2
chr13	23432682	23439871	dre-circ-3089	0	+	5	0	0	0	0
chr13	24164555	24167418	dre-circ-3090	0	-	0	0	0	3	0
chr13	2510189	2516107	dre-circ-1100	0	-	0	2	2	0	0
chr13	25282757	25295896	dre-circ-1127	0	-	2	0	0	0	0
chr13	25462483	25466539	dre-circ-3091	0	-	0	0	0	0	2
chr13	25993794	25995704	dre-circ-1128	0	-	0	0	0	2	0
chr13	26060248	26063400	dre-circ-3092	0	-	0	0	0	2	0
chr13	26777967	26781508	dre-circ-3093	0	-	0	0	0	0	3
chr1	327766	329393	dre-circ-6	0	-	0	0	0	2	0
chr13	28959027	28961909	dre-circ-1129	0	+	2	0	0	0	0
chr13	28975295	28975926	dre-circ-1130	0	+	0	0	0	0	3
chr13	2963936	2964420	dre-circ-3094	0	-	2	0	0	0	2
chr13	29983509	29987193	dre-circ-1131	0	-	0	0	0	2	0
chr13	30262500	30262874	dre-circ-3095	0	+	2	0	0	0	0
chr13	30398863	30404792	dre-circ-3096	0	+	0	3	0	0	0
chr13	31773254	31782790	dre-circ-1132	0	+	2	0	0	0	0
chr13	31998228	32007110	dre-circ-1133	0	+	0	0	2	0	0
chr13	32894343	32903680	dre-circ-1134	0	-	0	2	0	0	0
chr13	33647250	33649273	dre-circ-3097	0	+	2	0	4	2	0
chr1	33427	61370	dre-circ-1	0	+	0	0	7	0	0
chr13	34689974	34752826	dre-circ-3098	0	+	0	3	0	0	0
chr13	34949881	34956878	dre-circ-3099	0	+	0	2	2	0	0
chr13	34949881	34968819	dre-circ-3100	0	+	0	0	2	0	0
chr13	35575976	35581765	dre-circ-1135	0	+	2	0	0	0	0
chr13	35758929	35774372	dre-circ-1136	0	+	0	0	0	0	2
chr13	3580648	3582605	dre-circ-3101	0	-	3	0	0	0	0
chr13	36377370	36385838	dre-circ-1137	0	-	0	0	2	0	4
chr13	37115118	37115374	dre-circ-1138	0	-	2	0	0	0	0
chr13	37566804	37572058	dre-circ-3102	0	-	0	0	2	0	0
chr13	37579381	37591444	dre-circ-1139	0	-	0	0	2	0	0
chr13	37626432	37628618	dre-circ-3103	0	-	0	0	2	0	0
chr13	37824661	37853763	dre-circ-1140	0	-	0	0	2	0	0
chr13	3998173	3998336	dre-circ-1101	0	-	2	0	0	0	0
chr13	4010220	4013415	dre-circ-1102	0	-	2	0	0	0	0
chr13	41861306	41879575	dre-circ-1141	0	-	0	0	2	0	0
chr13	4227583	4247615	dre-circ-1103	0	+	0	0	2	0	0
chr13	4227586	4247615	dre-circ-3104	0	+	0	4	0	0	0
chr13	4255243	4256886	dre-circ-1104	0	+	0	0	2	0	0
chr13	42795046	42809332	dre-circ-1142	0	+	0	0	5	0	0
chr13	43031484	43031686	dre-circ-3105	0	-	2	0	0	0	0
chr13	43179260	43181486	dre-circ-3106	0	-	10	0	5	0	0
chr13	43446474	43514179	dre-circ-3107	0	+	0	0	0	0	2
chr13	4369509	4369640	dre-circ-1105	0	+	7	0	3	0	3
chr13	4369577	4369708	dre-circ-1106	0	+	0	0	0	2	0
chr13	4383628	4383987	dre-circ-3108	0	-	3	0	0	0	0
chr13	44175582	44181698	dre-circ-1143	0	-	2	0	0	0	0
chr13	44468466	44468932	dre-circ-1144	0	+	0	0	2	0	0
chr13	45332086	45335650	dre-circ-3109	0	-	3	0	0	0	0
chr13	45656004	45663674	dre-circ-3110	0	-	3	0	0	0	0
chr13	45771660	45775207	dre-circ-1145	0	-	0	0	0	2	0
chr13	46055030	46055155	dre-circ-1146	0	+	0	0	0	0	2
chr13	46117513	46135257	dre-circ-1147	0	-	2	0	0	0	0
chr13	46124368	46130632	dre-circ-3111	0	-	46	0	5	0	0
chr13	46135107	46135257	dre-circ-3112	0	-	4	0	0	0	0
chr13	46754263	46754740	dre-circ-3113	0	-	0	0	2	0	0
chr13	47282354	47283571	dre-circ-1148	0	-	2	0	0	0	0

chr13	48158057	48166139	dre-circ-3114	0	+	8	7	3	0	17
chr13	48640602	48640736	dre-circ-1149	0	-	0	0	0	0	2
chr13	50083878	50085564	dre-circ-1150	0	-	0	0	2	0	0
chr13	50143520	50145528	dre-circ-3115	0	-	0	0	0	0	2
chr13	50643449	50650536	dre-circ-1151	0	-	0	0	0	2	0
chr13	51052517	51056607	dre-circ-1152	0	-	0	0	2	0	0
chr13	51087679	51096815	dre-circ-1153	0	-	0	0	0	0	2
chr13	51205914	51207794	dre-circ-3116	0	-	4	0	0	0	0
chr13	52015866	52018269	dre-circ-3117	0	+	0	0	3	0	0
chr13	52169253	52174800	dre-circ-3118	0	+	0	0	0	3	0
chr13	52193222	52197559	dre-circ-3119	0	+	4	11	7	6	4
chr1	35290920	35295668	dre-circ-3120	0	+	0	0	5	0	0
chr1	35294662	35295668	dre-circ-43	0	+	0	0	0	2	0
chr13	53649055	53650687	dre-circ-1154	0	+	2	0	0	0	0
chr13	53649055	53655278	dre-circ-3121	0	+	3	0	0	0	0
chr13	53653346	53653778	dre-circ-3122	0	+	12	0	0	0	2
chr1	354482	354695	dre-circ-3123	0	-	6	0	0	0	0
chr13	5570882	5574611	dre-circ-3124	0	-	0	0	0	3	0
chr13	5769483	5777213	dre-circ-1107	0	+	0	0	2	0	0
chr13	6823529	6827026	dre-circ-1108	0	-	2	0	0	0	0
chr1	37095491	37115155	dre-circ-44	0	+	0	0	2	0	0
chr13	7619202	7619479	dre-circ-3125	0	+	0	0	0	0	5
chr13	997802	997998	dre-circ-1094	0	-	0	0	3	0	0
chr1	40455045	40462792	dre-circ-45	0	-	0	0	0	0	2
chr1	40694151	40716854	dre-circ-46	0	+	0	0	0	0	2
chr1	40708359	40708786	dre-circ-3126	0	+	3	0	0	0	18
chr1	40802877	40804460	dre-circ-3127	0	-	3	0	0	2	2
chr14	1012460	1015698	dre-circ-3128	0	+	0	0	3	0	0
chr14	1030714	1031735	dre-circ-3129	0	+	0	0	38	0	0
chr14	10579579	10583276	dre-circ-1166	0	-	2	0	0	0	0
chr1	41073694	41074623	dre-circ-47	0	-	0	0	0	0	2
chr1	41073694	41077745	dre-circ-48	0	-	0	0	0	0	6
chr1	41073694	41078819	dre-circ-49	0	-	3	0	0	0	2
chr1	41073694	41079866	dre-circ-50	0	-	2	0	0	0	3
chr1	41073923	41080074	dre-circ-51	0	-	0	0	0	0	2
chr1	41074166	41076159	dre-circ-52	0	-	0	0	0	0	4
chr1	41074166	41078260	dre-circ-53	0	-	0	0	0	0	3
chr1	41074713	41077745	dre-circ-54	0	-	0	0	0	0	2
chr1	41076397	41078397	dre-circ-55	0	-	3	0	0	0	6
chr1	41076806	41079866	dre-circ-56	0	-	0	0	0	0	4
chr14	1085226	1160870	dre-circ-1156	0	-	0	0	0	2	0
chr1	41119516	41120223	dre-circ-57	0	+	0	0	0	2	0
chr14	1120232	1127996	dre-circ-3130	0	+	3	0	0	0	0
chr14	1154317	1170247	dre-circ-3131	0	-	0	2	0	3	0
chr14	12697770	12704625	dre-circ-1167	0	+	2	0	0	0	0
chr14	13722474	13724167	dre-circ-3132	0	+	3	0	3	0	5
chr14	14115447	14115576	dre-circ-1168	0	-	0	2	0	0	0
chr14	14760292	14836162	dre-circ-1169	0	+	4	5	5	4	8
chr1	41611277	41611918	dre-circ-3133	0	-	0	0	0	0	8
chr14	18591038	18628323	dre-circ-1170	0	-	0	6	9	5	28
chr14	18594044	18630709	dre-circ-1171	0	-	7	3	6	3	17
chr14	18595066	18632758	dre-circ-1172	0	-	13	0	0	0	0
chr14	18595319	18632991	dre-circ-1173	0	-	0	4	0	4	0
chr14	18595770	18633433	dre-circ-1174	0	-	22	0	17	0	28
chr14	18596198	18633868	dre-circ-1175	0	-	0	0	0	0	8
chr14	18605283	18643694	dre-circ-1176	0	-	0	0	7	2	7
chr14	18605283	18643724	dre-circ-1177	0	-	9	0	12	7	25
chr14	18605577	18645668	dre-circ-1178	0	-	2	0	0	0	0
chr14	19091678	19131409	dre-circ-1179	0	+	0	0	0	7	0
chr14	19091678	19157983	dre-circ-1180	0	+	0	0	0	5	0
chr14	19131493	19157983	dre-circ-1181	0	+	0	0	0	5	0
chr14	19231895	19331306	dre-circ-1182	0	+	0	0	0	11	0
chr14	20025440	20026865	dre-circ-3134	0	-	0	0	2	0	0
chr14	20098053	20098577	dre-circ-3135	0	+	2	0	2	2	2
chr14	21177026	21190141	dre-circ-1183	0	+	2	0	0	0	0
chr14	2187416	2205170	dre-circ-1157	0	+	2	0	0	0	0
chr14	21897284	21902792	dre-circ-1184	0	+	0	2	23	3	43
chr14	21956169	21974711	dre-circ-3136	0	+	2	0	0	0	0
chr14	21956169	21979654	dre-circ-3137	0	+	2	0	0	0	0
chr14	24407929	24414693	dre-circ-1185	0	+	0	0	0	0	2
chr14	24561629	24562187	dre-circ-3138	0	+	2	0	0	0	0
chr14	26182740	26184113	dre-circ-1186	0	-	2	2	0	0	3
chr14	27002699	27021655	dre-circ-1187	0	+	0	0	0	0	2
chr14	27004089	27021655	dre-circ-1188	0	+	0	0	0	0	2
chr14	27792322	27793020	dre-circ-1189	0	+	0	0	6	2	0
chr14	27792322	27793719	dre-circ-1190	0	+	0	0	4	4	0
chr14	27792322	27795837	dre-circ-1191	0	+	0	0	0	2	0
chr14	27802054	27804387	dre-circ-1192	0	+	2	0	0	0	0
chr1	42857294	42857497	dre-circ-58	0	+	0	0	0	0	2
chr14	29371840	29386165	dre-circ-3139	0	+	0	0	0	0	3
chr14	30655650	30658797	dre-circ-3140	0	-	0	0	2	0	0
chr14	3069185	3069404	dre-circ-3141	0	-	43	0	0	0	0
chr14	31299687	31302961	dre-circ-3142	0	-	0	2	0	0	0
chr14	31729964	31730135	dre-circ-1193	0	-	0	2	0	0	0
chr14	31732837	31735268	dre-circ-1194	0	-	2	0	0	0	0
chr14	31833588	31833962	dre-circ-1195	0	-	0	0	2	0	0
chr14	33628163	33632852	dre-circ-1196	0	+	0	0	2	0	0

chr14	34229177	34232926	dre-circ-1197	0	-	2	0	0	0	0
chr14	34892801	34897172	dre-circ-3143	0	-	3	0	0	0	0
chr14	37830467	37850202	dre-circ-1198	0	+	0	0	0	0	2
chr14	38870486	38870862	dre-circ-3144	0	+	0	4	0	0	0
chr14	41643224	41648310	dre-circ-3145	0	-	0	5	0	0	0
chr14	4270057	4276095	dre-circ-3146	0	-	2	0	0	0	0
chr14	42831156	42888534	dre-circ-3147	0	+	0	0	0	0	13
chr14	4288240	4291791	dre-circ-1158	0	-	4	0	0	0	0
chr14	4494185	4494487	dre-circ-1159	0	+	0	2	0	0	0
chr14	47005872	47006375	dre-circ-3148	0	-	0	0	0	2	0
chr1	44705091	44707597	dre-circ-3149	0	+	16	0	0	0	0
chr14	47748243	47748537	dre-circ-3150	0	-	5	0	0	0	0
chr14	47943257	47947014	dre-circ-1199	0	+	0	0	0	0	2
chr14	47994943	48009814	dre-circ-3151	0	+	0	0	0	0	2
chr14	48570552	48570732	dre-circ-1200	0	-	0	0	0	0	2
chr14	49160546	49209905	dre-circ-3152	0	+	0	0	0	0	2
chr14	49630071	49646027	dre-circ-1201	0	-	0	2	0	0	0
chr1	45068320	45069015	dre-circ-59	0	-	0	0	0	0	2
chr14	50755654	50757094	dre-circ-1202	0	-	0	0	2	0	0
chr14	50858012	50858179	dre-circ-1203	0	-	0	0	2	0	0
chr14	51442346	51442706	dre-circ-1204	0	+	2	0	0	0	0
chr14	51557354	51566661	dre-circ-1205	0	+	0	0	4	0	0
chr14	51645249	51646434	dre-circ-3153	0	+	6	2	0	2	4
chr1	45331768	45350377	dre-circ-3154	0	+	0	0	2	0	0
chr14	53473753	53474830	dre-circ-3155	0	-	16	0	5	3	0
chr1	456941	461936	dre-circ-7	0	+	0	0	0	2	0
chr1	45705443	45707015	dre-circ-3156	0	+	0	5	0	0	0
chr14	5860940	5861458	dre-circ-3157	0	+	4	0	0	0	0
chr14	593619	593824	dre-circ-1155	0	-	0	0	3	0	0
chr1	45996643	46024770	dre-circ-60	0	-	0	0	0	2	0
chr1	46101877	46111430	dre-circ-3158	0	-	0	0	11	0	0
chr1	46155652	46160692	dre-circ-3159	0	+	2	0	0	0	0
chr14	6257546	6258312	dre-circ-3160	0	-	2	0	4	0	0
chr1	46270174	46276020	dre-circ-61	0	+	0	0	0	2	0
chr14	6315960	6334065	dre-circ-1160	0	+	0	0	0	0	2
chr14	6348145	6348431	dre-circ-1161	0	-	4	0	0	3	0
chr14	6348198	6348484	dre-circ-1162	0	-	0	0	0	2	7
chr14	6348207	6348493	dre-circ-1163	0	-	0	0	0	0	7
chr14	6425997	6428537	dre-circ-3161	0	+	0	0	0	0	3
chr14	6586155	6589767	dre-circ-3162	0	+	5	0	0	0	0
chr1	46836959	46841106	dre-circ-62	0	-	0	0	0	0	2
chr1	47126190	47129997	dre-circ-63	0	-	2	0	0	0	0
chr14	7149411	7154204	dre-circ-1164	0	+	0	0	0	2	0
chr1	4752008	4752191	dre-circ-3163	0	-	4	0	0	0	2
chr1	47617711	47656384	dre-circ-64	0	-	0	0	100	0	0
chr1	47619114	47631867	dre-circ-65	0	-	3	0	0	0	0
chr1	47619114	47642910	dre-circ-66	0	-	0	0	3	0	0
chr1	47619114	47657992	dre-circ-67	0	-	5	0	5	4	0
chr1	47627190	47641600	dre-circ-68	0	+	0	0	7	0	0
chr1	47632247	47642910	dre-circ-69	0	-	0	3	7	0	0
chr1	47632247	47657992	dre-circ-70	0	-	3	0	5	3	2
chr1	47648782	47657992	dre-circ-71	0	-	0	0	0	9	0
chr14	8578606	8579227	dre-circ-3164	0	+	0	0	0	2	0
chr14	8593554	8593930	dre-circ-3165	0	+	3	0	0	0	0
chr1	49102129	49102358	dre-circ-72	0	-	2	0	0	0	0
chr14	9865930	9869090	dre-circ-1165	0	-	0	0	0	2	0
chr1	50033249	50055550	dre-circ-73	0	-	0	0	5	0	2
chr1	50036492	50044915	dre-circ-74	0	-	0	0	4	0	3
chr1	50044356	50061755	dre-circ-75	0	-	0	0	6	0	2
chr1	5010139	5010513	dre-circ-3166	0	+	36	0	2	0	0
chr1	5013514	5018702	dre-circ-3167	0	+	5	0	22	7	2
chr1	50141716	50150718	dre-circ-76	0	-	0	0	2	0	0
chr1	5065577	5067030	dre-circ-25	0	+	2	0	0	0	0
chr1	5066893	5070720	dre-circ-3168	0	+	0	2	0	0	0
chr1	50764058	50768771	dre-circ-77	0	-	0	0	0	2	0
chr1	51158825	51159210	dre-circ-3169	0	+	2	0	0	0	0
chr15	12268715	12269118	dre-circ-1221	0	-	2	0	0	0	0
chr15	13633323	13633829	dre-circ-3170	0	+	4	0	0	0	0
chr15	15229938	15230325	dre-circ-3171	0	-	0	0	2	0	0
chr15	15884480	15885594	dre-circ-1222	0	-	2	0	0	0	0
chr15	16206553	16216985	dre-circ-1223	0	-	0	0	0	2	0
chr15	16206783	16217623	dre-circ-1224	0	-	2	0	0	0	0
chr15	16208997	16231746	dre-circ-1225	0	-	5	0	0	2	4
chr15	16221090	16229746	dre-circ-3172	0	-	0	0	3	0	2
chr15	1656294	1658972	dre-circ-3173	0	+	0	0	2	0	0
chr15	16901121	16903905	dre-circ-3174	0	-	0	0	2	0	0
chr15	16923501	16930225	dre-circ-3175	0	-	0	0	12	0	0
chr15	16926864	16930225	dre-circ-3176	0	-	0	0	4	0	0
chr15	16937597	16939443	dre-circ-1226	0	-	0	0	2	0	0
chr15	17054412	17056011	dre-circ-1227	0	-	2	0	2	0	0
chr15	17201365	17201510	dre-circ-1228	0	+	0	0	0	2	0
chr1	51841979	51849108	dre-circ-3177	0	+	0	4	0	2	0
chr15	1944098	1951631	dre-circ-1212	0	-	0	0	0	0	2
chr15	19868000	19870356	dre-circ-3178	0	-	0	0	2	0	0
chr15	20003626	20041558	dre-circ-1229	0	-	0	0	0	0	4
chr15	20075977	20098853	dre-circ-1230	0	-	0	0	0	2	0
chr15	20094021	20116432	dre-circ-1231	0	-	0	0	0	3	0

chr15	20785305	20791571	dre-circ-3179	0	-	3	0	0	0	0
chr15	22597300	22597736	dre-circ-3180	0	+	3	0	0	0	0
chr15	22972361	22975221	dre-circ-3181	0	+	0	0	2	0	0
chr15	23176756	23177351	dre-circ-1232	0	-	2	0	0	0	0
chr15	23196770	23229665	dre-circ-3182	0	-	2	0	0	0	0
chr15	23370743	23373772	dre-circ-1233	0	+	0	2	0	0	0
chr15	23906924	23908907	dre-circ-1234	0	+	2	0	0	0	0
chr15	2392181	2403233	dre-circ-3183	0	+	0	0	4	0	0
chr15	23927159	23943183	dre-circ-1235	0	+	0	0	2	0	2
chr15	24799151	24800185	dre-circ-3184	0	+	2	0	0	0	0
chr1	52543032	52548311	dre-circ-78	0	+	2	0	0	0	0
chr1	52543032	52550796	dre-circ-3185	0	+	0	0	4	0	0
chr15	26334067	26387270	dre-circ-1236	0	+	2	0	0	0	0
chr15	27354353	27354581	dre-circ-1237	0	-	0	3	0	0	0
chr15	27792181	27805903	dre-circ-1238	0	-	2	0	0	0	0
chr15	2823759	2833389	dre-circ-1213	0	-	2	0	0	0	0
chr15	28458692	28459253	dre-circ-3186	0	-	2	0	0	0	0
chr1	52891896	52892363	dre-circ-79	0	+	0	0	0	0	2
chr15	2894934	2959363	dre-circ-1214	0	-	4	0	0	0	0
chr15	2912411	2912918	dre-circ-3187	0	-	0	0	0	0	2
chr15	29633325	29655374	dre-circ-3188	0	+	3	0	0	0	0
chr15	29637991	29655374	dre-circ-3189	0	+	2	0	0	0	0
chr15	29675650	29677803	dre-circ-3190	0	+	2	0	0	0	0
chr1	52988225	52996094	dre-circ-80	0	-	0	0	0	5	0
chr1	52992244	52998489	dre-circ-81	0	-	0	0	0	2	0
chr15	30333167	30357048	dre-circ-1239	0	-	0	0	0	0	13
chr15	30335505	30357255	dre-circ-1240	0	-	0	0	2	0	0
chr15	30338743	30360228	dre-circ-1241	0	-	0	0	4	0	5
chr1	53086613	53087458	dre-circ-3191	0	+	9	0	6	2	0
chr15	31066906	31074466	dre-circ-1242	0	+	0	0	2	0	0
chr1	53167278	53171249	dre-circ-82	0	+	0	0	2	0	0
chr1	53182808	53183055	dre-circ-3192	0	+	2	0	0	0	0
chr1	53182812	53183196	dre-circ-83	0	+	0	0	2	0	0
chr1	53194434	53195215	dre-circ-84	0	+	4	0	4	0	5
chr15	32071843	32092934	dre-circ-1243	0	+	0	0	2	0	0
chr15	3319625	3323754	dre-circ-1215	0	+	2	0	0	0	0
chr15	333879	336568	dre-circ-1206	0	+	0	0	0	0	2
chr15	33446482	33469039	dre-circ-1244	0	-	0	0	0	2	0
chr15	33643710	33671749	dre-circ-1245	0	-	0	0	0	0	2
chr15	3419752	3420378	dre-circ-1216	0	-	0	0	0	2	0
chr15	34401023	34414602	dre-circ-1246	0	-	0	0	0	0	115
chr15	34404915	34414982	dre-circ-1247	0	-	0	0	0	0	71
chr15	34408894	34418732	dre-circ-1248	0	-	0	0	0	0	22
chr15	36635612	36716773	dre-circ-1249	0	+	0	0	0	0	2
chr15	36636167	36717882	dre-circ-1250	0	-	0	0	0	0	5
chr15	36636491	36718125	dre-circ-1251	0	+	0	0	5	0	14
chr15	36658281	36674830	dre-circ-1252	0	+	0	0	0	2	0
chr15	36672159	36708208	dre-circ-1253	0	+	0	0	0	2	0
chr15	36746081	36780786	dre-circ-1254	0	+	0	0	3	0	0
chr15	3676724	3677152	dre-circ-3193	0	+	3	0	0	0	0
chr15	37013944	37021556	dre-circ-3194	0	+	0	0	0	0	2
chr15	3772678	3773925	dre-circ-1217	0	-	0	0	2	0	0
chr15	38726333	38730489	dre-circ-3195	0	+	2	0	0	0	0
chr15	4011866	4013630	dre-circ-3196	0	-	0	0	0	2	2
chr15	40238830	40266099	dre-circ-1255	0	-	3	0	3	0	0
chr15	40238830	40273876	dre-circ-1256	0	-	2	0	0	0	0
chr15	40239648	40259563	dre-circ-3197	0	-	0	0	3	0	2
chr15	40244307	40278820	dre-circ-1257	0	-	4	0	0	0	0
chr15	40244454	40273339	dre-circ-3198	0	-	2	0	0	0	0
chr15	40245221	40250970	dre-circ-1258	0	-	3	0	0	0	0
chr15	40441077	40469478	dre-circ-1259	0	-	0	0	0	0	3
chr15	40456048	40471086	dre-circ-1260	0	+	0	0	0	0	3
chr15	41241282	41244186	dre-circ-1261	0	-	0	0	3	0	4
chr1	54177551	54179856	dre-circ-3199	0	-	4	2	0	0	0
chr15	41874541	41874859	dre-circ-1262	0	+	0	0	0	0	2
chr15	42192117	42192288	dre-circ-1263	0	+	2	0	0	0	0
chr15	42192117	42201473	dre-circ-1264	0	+	0	0	0	0	3
chr15	42490448	42490697	dre-circ-3200	0	-	0	3	0	0	0
chr15	42659790	42668375	dre-circ-1265	0	+	0	0	0	2	2
chr15	42790764	42800702	dre-circ-1266	0	-	0	0	2	0	0
chr1	54293682	54294120	dre-circ-85	0	-	0	4	0	0	0
chr15	43061461	43080344	dre-circ-3201	0	-	0	0	0	0	2
chr15	43092274	43095644	dre-circ-1267	0	-	2	0	0	0	0
chr15	43250392	43329626	dre-circ-1268	0	-	2	0	0	0	0
chr15	4325819	4331988	dre-circ-3202	0	-	2	0	0	0	0
chr15	4325819	4340622	dre-circ-3203	0	-	5	0	0	0	0
chr15	43856519	43865453	dre-circ-3204	0	+	0	0	2	0	2
chr15	44795590	44801220	dre-circ-3205	0	+	13	3	20	6	34
chr15	455326	457797	dre-circ-1207	0	+	2	0	0	0	0
chr15	45660890	45672972	dre-circ-1269	0	-	17	0	5	4	112
chr15	458071	461842	dre-circ-3206	0	+	4	0	0	0	0
chr15	45987543	45996028	dre-circ-1270	0	-	0	0	0	2	0
chr15	46211724	46213349	dre-circ-1271	0	-	0	0	0	0	5
chr15	46232414	46295234	dre-circ-3207	0	-	0	2	0	0	3
chr15	46365369	46368531	dre-circ-1272	0	-	2	0	0	0	0
chr15	46375614	46381458	dre-circ-3208	0	+	3	0	0	0	0
chr15	46774877	46781743	dre-circ-1273	0	+	18	0	0	0	12

chr1	54677664	54746137	dre-circ-3209	0	-	16	2	6	5	22
chr1	55316661	55380966	dre-circ-86	0	+	0	0	0	0	3
chr1	55426152	55427635	dre-circ-87	0	-	0	0	0	0	3
chr15	5477985	5483212	dre-circ-3210	0	+	0	0	3	0	0
chr15	5517570	5522870	dre-circ-1218	0	+	0	0	0	0	2
chr15	5551356	5551755	dre-circ-1219	0	+	0	2	0	0	0
chr1	55734525	55738556	dre-circ-3211	0	-	2	0	0	0	0
chr15	57997	69140	dre-circ-3212	0	-	0	2	0	4	0
chr1	56219044	56220991	dre-circ-88	0	+	2	0	0	0	0
chr1	56238515	56257407	dre-circ-89	0	+	0	0	0	0	2
chr1	56564783	56659991	dre-circ-90	0	-	0	0	3	0	0
chr1	56582365	56634740	dre-circ-91	0	-	0	0	2	0	0
chr1	56591203	56643892	dre-circ-92	0	-	0	0	0	0	2
chr1	56621264	56621511	dre-circ-93	0	-	0	0	2	0	0
chr15	6747265	6750106	dre-circ-1220	0	-	0	2	0	0	0
chr1	56777740	56821524	dre-circ-3213	0	+	0	0	0	2	0
chr1	56782093	56824136	dre-circ-94	0	+	0	0	6	6	0
chr1	56789737	56833178	dre-circ-95	0	+	0	0	17	0	0
chr1	56790590	56834610	dre-circ-96	0	+	0	0	13	5	0
chr1	56792880	56837841	dre-circ-3214	0	+	0	0	6	0	0
chr1	56794167	56840787	dre-circ-97	0	+	0	0	0	7	0
chr1	56797018	56850176	dre-circ-98	0	+	0	0	7	7	0
chr1	56802277	56853418	dre-circ-99	0	+	0	0	5	5	0
chr1	56803164	56854119	dre-circ-3215	0	+	0	0	0	9	0
chr1	56803307	56854711	dre-circ-100	0	+	0	0	0	10	0
chr1	56807773	56859648	dre-circ-101	0	+	0	0	17	8	0
chr1	56808507	56862657	dre-circ-102	0	+	0	0	0	7	0
chr1	56810667	56864787	dre-circ-3216	0	+	0	0	0	5	0
chr1	56813164	56865219	dre-circ-103	0	+	0	0	16	0	0
chr1	57498386	57501346	dre-circ-104	0	-	0	0	0	0	2
chr15	784502	785176	dre-circ-1208	0	+	0	0	0	0	8
chr15	785846	786436	dre-circ-1209	0	+	2	0	0	0	4
chr15	825590	863822	dre-circ-1210	0	+	0	0	0	2	0
chr1	58482940	58489148	dre-circ-3217	0	+	0	0	3	0	0
chr15	884143	907489	dre-circ-1211	0	-	0	0	2	0	0
chr1	58859293	58953702	dre-circ-105	0	+	0	0	0	0	2
chr1	59146275	59163797	dre-circ-106	0	-	0	0	0	0	4
chr1	59169354	59175479	dre-circ-107	0	-	0	0	0	0	2
chr1	59169354	59180503	dre-circ-108	0	-	0	0	0	0	2
chr1	59209691	59224430	dre-circ-109	0	+	0	0	0	3	0
chr1	59699163	59701702	dre-circ-3218	0	-	13	0	0	0	2
chr1	59699163	59704355	dre-circ-3219	0	-	6	0	0	0	0
chr1	59819777	59863015	dre-circ-110	0	+	0	0	6	0	0
chr1	59825825	59846234	dre-circ-111	0	+	0	0	0	0	7
chr1	59831870	59849867	dre-circ-112	0	-	0	0	0	0	76
chr1	59832292	59850320	dre-circ-113	0	-	0	0	0	0	14
chr1	59832577	59850607	dre-circ-114	0	-	5	0	0	0	88
chr1	59844261	59846662	dre-circ-115	0	+	0	0	9	0	4
chr1	59871842	59875860	dre-circ-116	0	-	0	0	13	0	0
chr1	59871842	59879675	dre-circ-117	0	-	0	0	3	0	0
chr1	59871842	59887299	dre-circ-118	0	-	0	0	5	0	0
chr1	59875490	59878947	dre-circ-119	0	-	0	0	2	0	0
chr1	59875490	59886848	dre-circ-120	0	-	0	0	2	2	0
chr1	59884804	59887025	dre-circ-121	0	-	0	0	2	0	0
chr16	10186590	10195434	dre-circ-1288	0	-	0	2	0	0	0
chr16	10229738	10251842	dre-circ-3220	0	-	0	4	0	0	0
chr16	10393648	10397326	dre-circ-3221	0	+	0	0	0	0	2
chr16	10502040	10513846	dre-circ-3222	0	+	0	0	0	0	2
chr16	11900346	11901400	dre-circ-1289	0	+	3	0	0	0	0
chr16	12050020	12050358	dre-circ-1290	0	+	4	0	0	0	0
chr16	12107297	12107736	dre-circ-1291	0	-	0	0	2	0	0
chr16	1290862	1293650	dre-circ-3223	0	+	3	0	0	0	0
chr16	1330814	1335433	dre-circ-3224	0	+	0	6	11	14	0
chr16	1330814	1343389	dre-circ-1276	0	+	0	0	2	2	0
chr16	13504709	13505332	dre-circ-1292	0	+	0	0	0	0	7
chr16	15739526	15758394	dre-circ-1293	0	+	0	0	0	14	0
chr16	15891196	15899109	dre-circ-3225	0	-	0	0	4	0	0
chr16	15893366	15899109	dre-circ-3226	0	-	0	2	2	0	0
chr16	15918978	15923748	dre-circ-3227	0	-	0	0	2	0	0
chr16	1711515	1758940	dre-circ-1277	0	+	0	0	0	0	6
chr16	18334297	18335134	dre-circ-1294	0	+	0	0	0	0	2
chr16	1870198	1904514	dre-circ-1278	0	+	0	0	0	0	2
chr16	18729897	18733804	dre-circ-1295	0	-	2	0	0	0	0
chr16	1890432	1896506	dre-circ-1279	0	+	0	0	0	0	4
chr16	1893449	1903956	dre-circ-3228	0	+	3	0	0	0	26
chr16	1905208	1934248	dre-circ-1280	0	+	0	0	0	0	3
chr16	19325904	19343460	dre-circ-3229	0	-	2	0	0	0	0
chr16	19332746	19343460	dre-circ-3230	0	-	3	0	0	0	0
chr16	19333129	19343460	dre-circ-3231	0	-	2	0	0	0	0
chr16	19555275	19557475	dre-circ-1296	0	-	2	0	0	0	0
chr16	19702645	19719098	dre-circ-1297	0	-	0	2	0	0	0
chr16	19714960	19719098	dre-circ-3232	0	-	12	2	8	0	7
chr16	20643958	20654576	dre-circ-1298	0	-	2	0	0	0	0
chr16	2074731	2117417	dre-circ-1281	0	+	0	0	0	0	3
chr16	21006291	21037692	dre-circ-1299	0	+	0	0	0	0	2
chr16	21251572	21253329	dre-circ-1300	0	-	0	2	0	0	0
chr16	21517633	21524086	dre-circ-1301	0	-	0	0	0	0	2

chr16	22217674	22239192	dre-circ-1302	0	+	0	0	2	0	0
chr16	22920609	22921365	dre-circ-1303	0	-	2	0	0	0	0
chr1	62317	64894	dre-circ-2	0	+	2	0	0	0	0
chr16	23995114	24077102	dre-circ-1304	0	-	0	0	0	0	3
chr16	24545961	24551656	dre-circ-3233	0	-	0	0	2	0	0
chr16	24629306	24687135	dre-circ-1305	0	-	0	0	0	0	2
chr16	24941150	24945305	dre-circ-1306	0	-	2	0	0	0	0
chr16	24979223	24984740	dre-circ-3234	0	-	7	0	0	5	0
chr16	25055253	25056058	dre-circ-1307	0	+	0	0	0	2	0
chr16	25671295	25729868	dre-circ-1308	0	-	0	0	0	0	3
chr16	26154105	26186796	dre-circ-1309	0	-	0	0	0	4	0
chr16	26154322	26186933	dre-circ-1310	0	+	0	0	0	14	0
chr16	26896635	26901751	dre-circ-1311	0	-	0	0	0	0	3
chr16	26897887	26902058	dre-circ-1312	0	-	0	0	0	0	7
chr16	26898363	26905017	dre-circ-1313	0	-	0	0	0	0	4
chr16	26934795	26934950	dre-circ-1314	0	+	0	0	0	0	2
chr16	27019509	27034979	dre-circ-3235	0	-	3	0	0	0	0
chr16	27505140	27507425	dre-circ-1315	0	+	0	0	0	2	0
chr16	27800051	27802789	dre-circ-1316	0	-	2	0	0	0	0
chr16	28253161	28257880	dre-circ-1317	0	+	0	0	0	0	11
chr16	28253732	28258469	dre-circ-1318	0	+	0	0	11	38	3
chr16	28253954	28258693	dre-circ-1319	0	+	0	0	20	0	0
chr16	28706309	28710390	dre-circ-3236	0	+	2	0	0	0	0
chr16	28719287	28719484	dre-circ-3237	0	+	11	0	0	0	0
chr16	28725766	28726011	dre-circ-1320	0	+	58	0	0	0	0
chr16	28725766	28727527	dre-circ-1321	0	+	24	0	0	0	0
chr16	28725772	28727287	dre-circ-1322	0	+	3	0	0	0	0
chr16	28730014	28730247	dre-circ-1323	0	+	104	0	11	3	0
chr16	28812973	28822282	dre-circ-3238	0	+	0	0	10	0	3
chr16	28825358	28825663	dre-circ-3239	0	+	2	0	0	0	0
chr16	29383957	29385999	dre-circ-3240	0	-	0	0	0	2	0
chr16	30620935	30634695	dre-circ-3241	0	+	0	0	3	0	0
chr16	309553	310076	dre-circ-1275	0	-	0	0	2	0	0
chr16	31335352	31339382	dre-circ-1324	0	-	2	0	0	0	0
chr16	31335352	31349121	dre-circ-3242	0	-	5	0	8	0	0
chr16	31773458	31779846	dre-circ-1325	0	+	0	0	0	0	6
chr16	3178553	3182379	dre-circ-3243	0	+	0	0	2	0	0
chr16	33111878	33112648	dre-circ-3244	0	-	3	0	2	2	0
chr16	33212309	33212462	dre-circ-1326	0	-	2	0	0	0	0
chr16	33570717	33621500	dre-circ-1327	0	-	0	0	0	0	2
chr16	33588864	33589621	dre-circ-3245	0	-	0	2	0	0	0
chr16	33747497	33750239	dre-circ-1328	0	-	5	0	0	0	2
chr16	34125672	34127980	dre-circ-3246	0	-	0	0	2	0	0
chr16	3429512	3429957	dre-circ-3247	0	+	6	0	3	0	0
chr16	34316128	34324265	dre-circ-1329	0	-	0	0	0	4	0
chr16	34502192	34502665	dre-circ-1330	0	+	0	0	0	2	4
chr16	35202645	35205397	dre-circ-1331	0	-	2	0	0	0	0
chr16	35416382	35416787	dre-circ-1332	0	-	2	0	0	0	0
chr16	35416442	35416787	dre-circ-3248	0	-	2	0	0	0	0
chr16	3591113	3596587	dre-circ-1282	0	-	2	0	0	0	0
chr16	36388117	36391193	dre-circ-3249	0	+	2	0	0	0	0
chr16	37590	38056	dre-circ-1274	0	+	2	0	0	0	0
chr16	38164688	38165216	dre-circ-3250	0	+	2	0	0	0	0
chr16	39555399	39555633	dre-circ-3251	0	-	2	0	0	0	0
chr16	39601901	39606269	dre-circ-3252	0	+	6	0	2	2	0
chr16	3976960	3977142	dre-circ-3253	0	+	0	0	3	0	0
chr16	4016991	4021988	dre-circ-3254	0	+	7	0	3	0	0
chr16	40557391	40559328	dre-circ-1333	0	+	0	0	0	0	2
chr16	40653580	40653759	dre-circ-1334	0	-	0	0	4	0	0
chr16	40978432	40985639	dre-circ-3255	0	-	4	2	77	10	8
chr16	42018271	42019369	dre-circ-1335	0	-	2	0	0	0	0
chr16	42332914	42351822	dre-circ-3256	0	-	0	0	2	0	0
chr16	42724988	42740556	dre-circ-1336	0	+	0	0	2	0	0
chr16	43683822	43687578	dre-circ-1337	0	+	2	0	0	0	0
chr16	43693840	43717197	dre-circ-1338	0	+	25	4	4	9	10
chr16	43694139	43717789	dre-circ-1339	0	-	0	0	0	0	2
chr16	43694962	43717843	dre-circ-1340	0	+	30	4	0	0	0
chr16	44470092	44470277	dre-circ-3257	0	-	0	0	0	2	0
chr16	44528984	44532443	dre-circ-3258	0	-	3	0	2	2	4
chr16	44691041	44691510	dre-circ-3259	0	-	2	0	0	0	0
chr16	45056826	45060506	dre-circ-1341	0	-	2	0	0	0	0
chr16	45204815	45204956	dre-circ-1342	0	-	0	2	0	0	0
chr16	45680960	45681167	dre-circ-1343	0	-	0	0	0	0	2
chr16	46144661	46145182	dre-circ-1344	0	-	2	0	0	0	0
chr16	46360562	46365194	dre-circ-3260	0	-	4	0	0	0	0
chr16	47215342	47242417	dre-circ-3261	0	-	0	8	0	0	0
chr16	47391925	47423250	dre-circ-3262	0	-	0	0	0	0	6
chr16	47518273	47518559	dre-circ-3263	0	+	15	0	0	0	0
chr16	47768328	47769224	dre-circ-3264	0	-	0	0	0	0	4
chr16	47774809	47775152	dre-circ-3265	0	-	0	0	0	0	8
chr16	48829858	48839628	dre-circ-3266	0	+	0	0	0	0	2
chr16	49035125	49038162	dre-circ-1345	0	+	0	2	0	0	0
chr16	49131123	49136838	dre-circ-1346	0	+	2	0	0	0	0
chr16	49498110	49524045	dre-circ-1347	0	+	0	2	0	0	0
chr16	49738321	49753709	dre-circ-1348	0	+	5	0	0	0	38
chr16	50614138	50619036	dre-circ-3267	0	-	0	0	2	0	0
chr16	50742403	50765231	dre-circ-1349	0	-	0	0	0	0	2

chr16	52020456	52039146	dre-circ-3268	0	+	0	0	0	0	2
chr16	52897711	52908703	dre-circ-1350	0	-	0	0	0	0	2
chr16	54486077	54502062	dre-circ-1351	0	+	0	0	0	0	4
chr16	54486077	54512226	dre-circ-1352	0	+	0	0	0	0	4
chr16	54488881	54516754	dre-circ-1353	0	+	0	0	0	0	12
chr16	54490097	54519866	dre-circ-1354	0	+	0	0	0	0	3
chr16	54490103	54519866	dre-circ-1355	0	+	0	0	0	0	7
chr16	54849727	54897500	dre-circ-1356	0	+	0	0	0	0	2
chr16	54849951	54897696	dre-circ-1357	0	+	0	0	6	0	0
chr16	54876932	54892688	dre-circ-1358	0	+	0	0	6	2	7
chr16	54877171	54897500	dre-circ-1359	0	+	2	0	8	2	9
chr16	54877373	54897696	dre-circ-1360	0	+	0	0	0	4	0
chr16	54879262	54898243	dre-circ-1361	0	+	0	0	0	0	3
chr16	5560087	5573153	dre-circ-3269	0	-	0	10	0	2	0
chr16	55673657	55674022	dre-circ-1362	0	+	2	0	0	0	0
chr16	55950511	55958260	dre-circ-1363	0	+	0	0	0	0	2
chr16	55964658	55967490	dre-circ-3270	0	+	0	0	0	0	3
chr16	56209460	56218181	dre-circ-3271	0	-	3	0	0	2	0
chr16	56432614	56441840	dre-circ-3272	0	-	0	0	0	2	2
chr16	56436247	56441840	dre-circ-3273	0	-	0	0	0	0	3
chr16	56481013	56483034	dre-circ-1364	0	-	0	0	0	0	2
chr16	56769815	56770421	dre-circ-3274	0	+	0	0	0	0	2
chr16	57127251	57127533	dre-circ-3275	0	+	13	0	2	0	0
chr16	57861874	57862613	dre-circ-1365	0	+	2	0	0	0	0
chr16	57883302	57884355	dre-circ-1366	0	-	14	0	2	0	10
chr16	6887418	6892375	dre-circ-1283	0	+	0	0	0	0	2
chr16	7438840	7444681	dre-circ-3276	0	-	0	0	0	3	0
chr16	7998090	7998537	dre-circ-1284	0	+	2	0	0	0	0
chr16	8006897	8009879	dre-circ-1285	0	+	2	0	0	0	0
chr16	8116905	8120714	dre-circ-3277	0	+	0	0	0	2	2
chr16	8232058	8284671	dre-circ-3278	0	+	2	0	0	0	0
chr16	9025066	9027548	dre-circ-3279	0	-	0	0	3	0	0
chr16	9283283	9285487	dre-circ-1286	0	-	0	0	0	2	0
chr16	9665099	9679551	dre-circ-1287	0	-	0	0	0	2	0
chr17	10253026	10253208	dre-circ-1403	0	+	0	0	0	0	2
chr17	11319288	11329743	dre-circ-1404	0	-	2	0	0	0	0
chr17	1136605	1152121	dre-circ-3280	0	+	4	0	0	0	0
chr17	1145578	1148493	dre-circ-3281	0	+	2	0	0	0	0
chr17	11555728	11555990	dre-circ-3282	0	+	0	0	5	5	0
chr17	12886341	12886851	dre-circ-3283	0	-	4	0	0	0	2
chr17	12932811	12938305	dre-circ-1405	0	-	0	0	0	2	0
chr17	12954893	12955144	dre-circ-3284	0	+	3	2	2	0	0
chr17	14495070	14505713	dre-circ-1406	0	-	0	0	0	0	2
chr17	145524	150594	dre-circ-3285	0	-	2	0	0	0	0
chr17	14882701	14882813	dre-circ-1407	0	+	0	0	2	0	0
chr17	15087665	15088664	dre-circ-1408	0	-	0	0	0	0	2
chr17	15150840	15160516	dre-circ-1409	0	-	2	0	0	0	0
chr17	15299719	15311754	dre-circ-1410	0	-	0	0	0	2	0
chr17	15644356	15651393	dre-circ-3286	0	+	0	0	2	0	0
chr17	16463612	16464168	dre-circ-3287	0	+	0	0	4	0	3
chr17	17884097	17885559	dre-circ-3288	0	+	30	2	9	7	0
chr17	17884097	17892569	dre-circ-1411	0	+	0	0	2	0	0
chr17	17915184	17919738	dre-circ-3289	0	+	3	0	0	0	0
chr17	19890304	19891111	dre-circ-3290	0	-	0	0	363	0	0
chr17	19911129	19912376	dre-circ-3291	0	-	0	0	5	0	0
chr17	20089419	20089912	dre-circ-1412	0	-	0	2	0	0	0
chr17	20546607	20564417	dre-circ-3292	0	-	0	0	2	0	0
chr17	21296562	21322163	dre-circ-1413	0	+	0	0	0	0	2
chr17	22498978	22499659	dre-circ-1414	0	+	0	0	2	0	0
chr17	2270102	2284306	dre-circ-3293	0	-	0	0	2	0	0
chr17	2276447	2284306	dre-circ-3294	0	-	0	5	4	5	0
chr17	2280349	2284306	dre-circ-3295	0	-	0	0	3	0	0
chr17	2347660	2365505	dre-circ-1368	0	-	0	0	0	0	29
chr17	2349428	2354621	dre-circ-1369	0	-	0	0	0	0	196
chr17	2349428	2366980	dre-circ-1370	0	-	0	0	0	0	116
chr17	2350450	2355006	dre-circ-1371	0	-	0	0	0	0	55
chr17	2350450	2360403	dre-circ-1372	0	-	0	0	0	0	242
chr17	2350450	2367376	dre-circ-1373	0	-	0	0	0	0	159
chr17	2350450	2372577	dre-circ-1374	0	-	0	0	0	0	179
chr17	2354888	2360179	dre-circ-1375	0	-	0	0	0	0	144
chr17	2354888	2372281	dre-circ-1376	0	-	0	0	0	0	222
chr17	2355451	2360403	dre-circ-1377	0	-	0	0	0	0	80
chr17	2355451	2367376	dre-circ-1378	0	-	0	0	0	0	52
chr17	2355451	2372577	dre-circ-1379	0	-	0	0	0	0	184
chr17	2355595	2373083	dre-circ-1380	0	-	0	0	0	0	24
chr17	2355855	2368076	dre-circ-1381	0	-	0	0	0	0	38
chr17	2355855	2373343	dre-circ-1382	0	-	0	0	0	0	47
chr17	2358685	2365505	dre-circ-1383	0	-	0	0	0	0	395
chr17	2358685	2370479	dre-circ-1384	0	-	0	0	0	0	410
chr17	2361859	2367376	dre-circ-1385	0	-	0	0	0	0	287
chr17	2362134	2367947	dre-circ-1386	0	+	0	0	0	0	30
chr17	2367258	2372281	dre-circ-1387	0	-	0	0	0	0	127
chr17	2367673	2372577	dre-circ-1388	0	-	0	0	0	0	114
chr17	25105645	25106727	dre-circ-3296	0	-	0	0	2	0	0
chr17	25126298	25131786	dre-circ-1415	0	+	0	0	0	0	2
chr17	25186979	25238729	dre-circ-1416	0	-	0	0	0	3	0
chr17	26434814	26438729	dre-circ-1417	0	-	2	0	0	0	0

chr17	26847283	26849610	dre-circ-1418	0	-	0	0	0	0	2
chr17	28781285	28784511	dre-circ-3297	0	+	5	0	9	0	2
chr17	28781285	28790031	dre-circ-3298	0	+	2	2	5	0	0
chr17	28852069	28853093	dre-circ-3299	0	-	7	12	17	14	6
chr17	30537912	30538804	dre-circ-3300	0	-	4	0	3	0	0
chr17	30562242	30564667	dre-circ-3301	0	-	2	0	0	0	2
chr17	31403167	31411398	dre-circ-1419	0	-	8	0	0	0	0
chr17	31435542	31448396	dre-circ-3302	0	+	0	0	0	0	2
chr17	31630	57588	dre-circ-1367	0	+	4	0	2	0	2
chr17	3193728	3215830	dre-circ-1389	0	+	0	0	0	0	2
chr17	32622068	32652649	dre-circ-1420	0	+	2	0	0	0	0
chr17	33398039	33403634	dre-circ-1421	0	+	2	0	0	0	0
chr17	35375187	35375440	dre-circ-3303	0	+	0	0	5	0	0
chr17	3625046	3628580	dre-circ-1390	0	-	0	0	0	0	2
chr17	37351200	37358291	dre-circ-3304	0	-	2	0	0	0	0
chr17	37497512	37501085	dre-circ-1422	0	+	2	0	0	0	0
chr17	37510463	37511083	dre-circ-1423	0	+	2	0	2	0	0
chr17	3833141	3844364	dre-circ-1391	0	-	0	0	0	2	0
chr17	38706211	38706334	dre-circ-1424	0	+	0	0	2	0	0
chr17	38758282	38758631	dre-circ-1425	0	+	0	0	2	0	0
chr17	40002068	40007738	dre-circ-3305	0	+	15	5	9	4	11
chr17	4098906	4099222	dre-circ-3306	0	+	16	0	0	0	2
chr17	4103664	4108226	dre-circ-1392	0	+	0	0	0	0	2
chr17	41042269	41043332	dre-circ-3307	0	+	3	0	2	0	0
chr17	41057869	41059136	dre-circ-1426	0	+	3	0	0	0	0
chr17	41801318	41803794	dre-circ-3308	0	-	4	0	3	0	0
chr17	4188359	4188517	dre-circ-1393	0	+	0	0	0	0	2
chr17	4242464	4245829	dre-circ-3309	0	+	0	0	0	0	4
chr17	43347171	43350406	dre-circ-1427	0	-	0	2	0	0	0
chr17	44013086	44015823	dre-circ-3310	0	+	2	0	3	0	0
chr17	44914120	44915511	dre-circ-3311	0	+	0	0	4	0	0
chr17	45326693	45329719	dre-circ-1428	0	-	0	0	0	0	2
chr17	45531934	45532806	dre-circ-1429	0	+	0	0	2	0	0
chr17	45549785	45550392	dre-circ-3312	0	-	26	2	10	3	3
chr17	49058995	49059180	dre-circ-1430	0	+	0	2	0	0	0
chr17	49082463	49085000	dre-circ-3313	0	-	0	0	0	8	0
chr17	49162480	49167834	dre-circ-1431	0	+	0	0	2	0	0
chr17	49616531	49698119	dre-circ-1432	0	+	2	0	0	0	10
chr17	49734202	49762996	dre-circ-1433	0	+	2	0	0	0	6
chr17	49743388	49765986	dre-circ-1434	0	+	0	0	0	0	2
chr17	50575631	50593305	dre-circ-1435	0	-	0	0	0	0	2
chr17	50726939	50731619	dre-circ-3314	0	-	0	0	5	0	0
chr17	50996822	50997859	dre-circ-1436	0	-	2	0	0	0	0
chr17	5222713	5227962	dre-circ-3315	0	+	0	0	0	4	0
chr17	52349893	52350619	dre-circ-1437	0	+	0	0	0	0	2
chr17	53726126	53727686	dre-circ-1438	0	+	0	0	0	0	2
chr17	53975109	53977670	dre-circ-1439	0	+	0	2	0	0	0
chr17	6339490	6340874	dre-circ-3316	0	+	2	0	0	0	3
chr17	6339490	6344373	dre-circ-1394	0	+	0	0	0	0	2
chr17	6436166	6474738	dre-circ-1395	0	+	0	0	0	0	2
chr17	6440831	6441792	dre-circ-3317	0	+	3	0	4	0	0
chr17	6467874	6468189	dre-circ-1396	0	+	0	0	0	2	0
chr17	6467874	6474738	dre-circ-1397	0	+	0	0	0	0	2
chr1	7699147	7703431	dre-circ-26	0	+	0	0	0	0	2
chr17	7358670	7368996	dre-circ-1398	0	-	3	0	0	0	0
chr1	7777574	7778068	dre-circ-3318	0	+	6	0	0	0	0
chr17	7937362	7945236	dre-circ-3319	0	-	0	0	0	2	0
chr17	7940685	7945236	dre-circ-1399	0	-	0	0	0	2	0
chr17	8065756	8066468	dre-circ-3320	0	+	3	0	0	0	0
chr17	8194337	8207115	dre-circ-3321	0	+	5	0	0	0	0
chr17	8427197	8427331	dre-circ-1400	0	-	0	0	0	0	2
chr17	8528189	8528756	dre-circ-1401	0	-	2	0	0	0	0
chr17	8594505	8594882	dre-circ-3322	0	-	2	0	0	0	0
chr17	9356379	9356581	dre-circ-1402	0	-	0	0	2	0	0
chr17	957037	961856	dre-circ-3323	0	+	3	0	0	0	0
chr1	7983134	7992924	dre-circ-3324	0	-	0	0	0	0	3
chr18	10281304	10282090	dre-circ-3325	0	+	0	0	0	2	0
chr18	10320182	10325624	dre-circ-3326	0	+	5	0	3	0	0
chr18	10335834	10344500	dre-circ-1454	0	+	0	0	0	0	2
chr18	11768296	11768590	dre-circ-1455	0	+	0	3	0	0	0
chr18	11924320	11944051	dre-circ-1456	0	-	0	0	0	2	0
chr18	127614	129307	dre-circ-1440	0	+	0	0	2	0	0
chr18	14155586	14155912	dre-circ-3327	0	+	0	0	0	2	0
chr18	14284358	14286889	dre-circ-3328	0	+	5	2	3	0	0
chr18	15063572	15153037	dre-circ-1457	0	-	0	0	5	0	0
chr18	15075209	15169264	dre-circ-1458	0	-	0	0	4	0	0
chr18	15088266	15187499	dre-circ-1459	0	-	0	0	0	0	4
chr18	15794940	15797317	dre-circ-3329	0	+	2	0	0	0	0
chr18	16443986	16454938	dre-circ-1460	0	-	0	0	2	0	0
chr18	16707203	16710688	dre-circ-3330	0	-	14	0	0	0	0
chr18	16935872	16937320	dre-circ-1461	0	-	2	0	0	0	0
chr18	17335187	17338070	dre-circ-3331	0	+	0	0	0	3	0
chr18	1807013	1809684	dre-circ-1442	0	+	0	0	2	0	0
chr18	18438234	18477068	dre-circ-1462	0	-	4	0	8	5	3
chr18	18438234	18501540	dre-circ-1463	0	-	0	0	3	4	6
chr18	18438234	18525323	dre-circ-1464	0	-	0	0	0	0	3
chr18	18442210	18481463	dre-circ-1465	0	-	7	4	7	5	5

chr18	18442210	18506586	dre-circ-1466	0	-	5	3	6	6	7
chr18	18442210	18528945	dre-circ-1467	0	-	13	3	5	10	10
chr18	18448064	18482499	dre-circ-1468	0	-	0	0	3	2	6
chr18	18448064	18507767	dre-circ-1469	0	-	2	0	3	0	5
chr18	18448064	18530140	dre-circ-1470	0	-	0	0	2	2	6
chr18	1946132	1950080	dre-circ-1443	0	-	2	0	0	0	0
chr18	20008272	20020136	dre-circ-1471	0	+	2	0	0	0	0
chr18	20472483	20472674	dre-circ-3332	0	+	0	0	0	2	0
chr18	20651844	20652348	dre-circ-3333	0	+	0	0	0	6	0
chr18	20651844	20655924	dre-circ-3334	0	+	0	0	0	4	0
chr18	21892924	21904561	dre-circ-1472	0	+	0	0	0	0	2
chr18	21896426	21904854	dre-circ-1473	0	+	0	0	0	2	0
chr18	22008449	22014062	dre-circ-1474	0	+	0	0	4	0	0
chr18	22832508	22832819	dre-circ-3335	0	+	8	0	0	0	0
chr18	23091228	23094420	dre-circ-1475	0	+	0	0	2	0	0
chr18	25380206	25385792	dre-circ-3336	0	-	2	0	2	0	0
chr18	25591784	25608821	dre-circ-1476	0	-	0	0	0	0	2
chr18	26680090	26682538	dre-circ-3337	0	+	0	0	15	0	0
chr18	27875745	27877008	dre-circ-1477	0	+	0	0	0	5	0
chr18	27931188	27933114	dre-circ-3338	0	+	0	0	3	2	0
chr18	28399633	28402917	dre-circ-1478	0	-	0	2	0	0	0
chr18	29112369	29124392	dre-circ-1479	0	+	0	0	2	0	0
chr18	29116394	29124392	dre-circ-1480	0	+	0	0	3	0	0
chr18	29928443	29928612	dre-circ-1481	0	-	2	0	0	0	0
chr18	300788	355125	dre-circ-3339	0	-	0	0	4	2	61
chr18	31039547	31045181	dre-circ-3340	0	+	3	0	0	0	0
chr18	31040529	31045181	dre-circ-3341	0	+	2	2	3	0	0
chr18	32387519	32394029	dre-circ-1482	0	-	0	0	0	0	2
chr18	33241140	33245276	dre-circ-3342	0	+	2	0	0	0	0
chr18	35649600	35653017	dre-circ-3343	0	-	2	0	0	0	2
chr18	361244	361399	dre-circ-1441	0	-	0	0	2	0	0
chr18	36275898	36285500	dre-circ-3344	0	-	0	3	0	0	0
chr18	36275898	36316518	dre-circ-1483	0	-	0	2	0	0	0
chr18	36285396	36285500	dre-circ-1484	0	-	0	9	0	0	0
chr18	36303289	36316518	dre-circ-3345	0	-	0	3	0	0	0
chr18	36303289	36332175	dre-circ-1485	0	-	0	2	0	0	0
chr18	36357491	36381365	dre-circ-1486	0	-	0	2	0	0	0
chr18	37748807	37753802	dre-circ-1487	0	-	0	0	0	0	3
chr18	37780181	37782190	dre-circ-3346	0	+	2	0	4	0	0
chr18	38872692	38949858	dre-circ-1488	0	+	0	0	0	3	0
chr18	38893387	38950703	dre-circ-1489	0	+	0	0	0	2	0
chr18	38919287	38940955	dre-circ-1490	0	+	0	0	0	24	0
chr18	38919741	38943015	dre-circ-1491	0	+	0	0	0	25	0
chr18	38926878	38949858	dre-circ-1492	0	+	0	0	0	14	0
chr18	38933112	38955541	dre-circ-1493	0	+	0	0	0	25	0
chr18	38934175	38957544	dre-circ-1494	0	+	0	0	0	10	0
chr18	39646077	39646914	dre-circ-3347	0	+	2	0	0	0	0
chr18	39860552	39864703	dre-circ-3348	0	+	0	0	0	0	2
chr18	39860728	39862577	dre-circ-1495	0	+	0	0	2	0	0
chr18	42259543	42264764	dre-circ-1496	0	-	0	0	2	0	0
chr18	42984845	42985335	dre-circ-3349	0	+	6	0	0	0	0
chr18	43226426	43232273	dre-circ-1497	0	+	0	0	0	2	0
chr18	43273366	43274601	dre-circ-1498	0	+	0	0	2	0	0
chr18	43610407	43614369	dre-circ-1499	0	+	0	0	0	2	0
chr18	44022454	44026139	dre-circ-1500	0	-	0	0	0	0	2
chr18	44567991	44568135	dre-circ-1501	0	+	2	0	0	0	0
chr18	44627489	44628760	dre-circ-1502	0	-	0	0	0	2	0
chr18	45269776	45270361	dre-circ-1503	0	-	0	0	0	2	0
chr18	4531600	4618096	dre-circ-3350	0	+	0	2	0	0	0
chr18	47010208	47012743	dre-circ-3351	0	+	0	0	4	0	0
chr18	47010208	47029974	dre-circ-1504	0	+	0	0	2	0	0
chr18	47079699	47091961	dre-circ-1505	0	+	0	0	0	0	4
chr18	47353895	47356844	dre-circ-1506	0	-	6	0	21	0	7
chr18	47353895	47360623	dre-circ-1507	0	-	0	0	26	0	16
chr18	47356845	47360623	dre-circ-1508	0	-	16	4	19	0	0
chr18	47449809	47450219	dre-circ-3352	0	-	2	0	0	0	0
chr18	47463906	47468820	dre-circ-3353	0	-	9	0	0	0	0
chr18	47812718	47823305	dre-circ-1509	0	+	2	0	0	0	0
chr18	47878629	47879031	dre-circ-3354	0	-	0	0	23	2	6
chr18	47899284	47906977	dre-circ-3355	0	-	0	0	2	0	0
chr18	49470012	49514441	dre-circ-3356	0	-	2	0	0	0	14
chr18	49539771	49540310	dre-circ-3357	0	-	3	0	0	0	0
chr18	49589959	49597281	dre-circ-1510	0	+	2	0	0	0	0
chr18	49591919	49593745	dre-circ-1511	0	+	0	0	4	0	0
chr18	49591919	49597486	dre-circ-1512	0	+	6	0	0	0	0
chr18	49591919	49599343	dre-circ-1513	0	+	0	0	5	0	0
chr18	49592266	49593972	dre-circ-1514	0	+	3	0	0	0	0
chr18	49597611	49599343	dre-circ-1515	0	+	7	0	0	0	0
chr18	49599928	49601238	dre-circ-1516	0	+	2	0	0	0	0
chr18	49600214	49603285	dre-circ-1517	0	+	5	0	2	0	0
chr18	49873958	49874255	dre-circ-1518	0	+	2	0	0	0	0
chr18	5361612	5361945	dre-circ-3358	0	+	0	0	2	0	0
chr18	5573808	5574813	dre-circ-1444	0	+	0	0	0	2	0
chr1	8588328	8588815	dre-circ-3359	0	-	4	0	0	0	0
chr18	6048356	6048610	dre-circ-1445	0	-	0	0	0	0	2
chr18	6104258	6110286	dre-circ-1446	0	-	0	0	0	2	0
chr18	6523578	6530370	dre-circ-1447	0	-	0	0	0	0	2

chr18	6773905	6774382	dre-circ-1448	0	+	0	0	0	2	0
chr18	6780203	6782687	dre-circ-3360	0	+	0	0	2	0	0
chr18	7012238	7019214	dre-circ-3361	0	+	0	0	11	6	17
chr18	7012499	7023345	dre-circ-3362	0	+	0	0	0	9	24
chr18	7015387	7023764	dre-circ-1449	0	+	14	4	14	0	60
chr18	7034730	7034838	dre-circ-1450	0	-	21	2	24	7	47
chr18	8436276	8447916	dre-circ-3363	0	-	0	2	0	0	0
chr18	8770103	8777452	dre-circ-1451	0	+	0	0	0	0	2
chr18	8871736	8884131	dre-circ-1452	0	+	0	0	2	0	0
chr18	9092386	9095623	dre-circ-1453	0	-	0	4	0	0	0
chr19	10853355	10861252	dre-circ-1541	0	-	0	2	0	0	0
chr19	1092747	1096051	dre-circ-1522	0	+	0	2	0	0	0
chr19	11187155	11190982	dre-circ-3364	0	+	0	0	0	2	3
chr19	11275419	11276485	dre-circ-1542	0	-	0	0	2	0	0
chr19	11319765	11324995	dre-circ-3365	0	-	0	0	0	2	0
chr19	11335206	11369077	dre-circ-1543	0	-	0	0	0	3	0
chr19	11458597	11463167	dre-circ-1544	0	-	2	0	0	0	0
chr19	11849228	11849649	dre-circ-3366	0	+	0	0	0	0	4
chr19	11985129	11994062	dre-circ-1545	0	+	2	0	0	0	0
chr19	12760534	12760963	dre-circ-1546	0	-	2	2	0	0	0
chr19	1497503	1498222	dre-circ-3367	0	-	0	0	0	2	0
chr19	18130967	18170930	dre-circ-3368	0	-	2	0	3	0	0
chr19	18328345	18330391	dre-circ-1547	0	+	0	2	0	0	0
chr19	19532917	19552577	dre-circ-3369	0	-	0	0	0	2	0
chr19	19692509	19695154	dre-circ-3370	0	-	2	0	3	0	2
chr19	19733451	19741187	dre-circ-3371	0	-	0	0	2	2	0
chr19	19897264	19905483	dre-circ-3372	0	-	0	0	0	0	2
chr19	20505237	20529758	dre-circ-1548	0	+	0	0	2	0	0
chr19	20526987	20527359	dre-circ-3373	0	+	5	0	0	0	0
chr19	2233014	2246371	dre-circ-3374	0	-	0	0	0	0	2
chr19	22650731	22651124	dre-circ-1549	0	+	0	0	0	2	0
chr19	22946830	22959693	dre-circ-3375	0	-	0	2	7	3	0
chr19	23128775	23142736	dre-circ-1550	0	-	0	2	0	0	0
chr19	23274599	23275198	dre-circ-3376	0	-	2	0	2	0	0
chr19	23342283	23424968	dre-circ-1551	0	+	0	0	0	0	9
chr19	23348704	23433291	dre-circ-1552	0	+	0	0	0	0	3
chr19	23349117	23433562	dre-circ-1553	0	-	0	0	0	0	2
chr19	23369178	23450383	dre-circ-1554	0	+	0	0	0	0	12
chr19	23377064	23457415	dre-circ-1555	0	+	0	0	0	0	23
chr19	23379290	23458711	dre-circ-1556	0	+	0	0	0	0	9
chr19	23382924	23460780	dre-circ-1557	0	+	0	0	0	0	3
chr19	23387221	23462333	dre-circ-1558	0	+	2	0	0	0	53
chr19	23392178	23468823	dre-circ-1559	0	+	0	0	0	0	2
chr19	23393723	23470872	dre-circ-1560	0	+	8	0	0	0	55
chr19	23396789	23472549	dre-circ-1561	0	+	0	0	0	0	2
chr19	23397305	23473035	dre-circ-1562	0	+	0	0	0	0	23
chr19	23400203	23476682	dre-circ-1563	0	+	0	0	0	0	9
chr19	23400471	23476926	dre-circ-1564	0	+	0	0	0	0	18
chr19	23402814	23480768	dre-circ-1565	0	+	0	0	0	0	17
chr19	24977575	24977754	dre-circ-1566	0	+	2	0	0	0	0
chr19	26146061	26151409	dre-circ-1567	0	-	2	0	0	0	0
chr19	27802309	27802501	dre-circ-1568	0	-	2	0	0	0	0
chr19	28008431	28008964	dre-circ-3377	0	-	0	0	0	2	3
chr19	28034663	28072856	dre-circ-1569	0	-	0	0	0	0	32
chr19	28048962	28055370	dre-circ-1570	0	-	0	0	0	0	17
chr19	28048962	28064557	dre-circ-1571	0	-	0	0	0	0	3
chr19	28048962	28073864	dre-circ-1572	0	-	0	0	0	0	12
chr19	28063383	28072639	dre-circ-1573	0	-	0	0	0	0	25
chr19	28168715	28173230	dre-circ-3378	0	-	0	0	3	0	0
chr19	28371645	28373115	dre-circ-1574	0	+	0	0	2	0	0
chr19	2970735	2980898	dre-circ-1523	0	+	0	0	0	0	2
chr19	30836573	30841758	dre-circ-3379	0	+	3	0	0	0	0
chr19	30871382	30871490	dre-circ-1575	0	+	0	0	0	0	2
chr19	3104574	3105842	dre-circ-3380	0	+	0	0	3	5	0
chr19	31101859	31102277	dre-circ-1576	0	+	2	0	0	0	0
chr19	31118140	31121859	dre-circ-3381	0	-	0	0	4	0	0
chr19	32667030	32669667	dre-circ-1577	0	+	0	2	0	0	0
chr19	32889607	32900661	dre-circ-3382	0	-	0	0	0	2	0
chr19	33865412	33873236	dre-circ-1578	0	-	0	0	0	0	3
chr19	33865547	33873291	dre-circ-1579	0	-	0	0	0	0	61
chr19	33869151	33875644	dre-circ-1580	0	-	0	0	0	0	2
chr19	33881279	33890516	dre-circ-1581	0	-	2	0	0	0	0
chr19	3521135	3530431	dre-circ-1524	0	-	2	0	0	0	0
chr19	35233183	35284315	dre-circ-1582	0	+	0	0	0	0	2
chr19	35240305	35288809	dre-circ-1583	0	+	2	0	0	0	0
chr19	361079	362942	dre-circ-1519	0	+	46	0	20	5	35
chr19	36708008	36710480	dre-circ-3383	0	-	5	0	0	0	0
chr19	36753642	36770300	dre-circ-3384	0	-	3	0	0	0	0
chr19	39060419	39060828	dre-circ-3385	0	+	6	0	0	0	0
chr19	39065656	39069860	dre-circ-3386	0	+	4	0	0	0	0
chr19	39066297	39066401	dre-circ-1584	0	+	2	0	0	0	0
chr19	39075744	39076334	dre-circ-1585	0	+	27	0	0	0	0
chr19	39075751	39076341	dre-circ-1586	0	-	30	0	0	0	0
chr19	39080443	39081789	dre-circ-1587	0	-	95	0	0	0	0
chr19	39080443	39083820	dre-circ-1588	0	-	3	0	0	0	0
chr19	39080618	39082744	dre-circ-1589	0	+	4	0	0	0	0
chr19	39080830	39088848	dre-circ-1590	0	+	65	0	0	0	0

chr19	39082973	39088084	dre-circ-1591	0	+	3	0	0	0	0
chr19	39083041	39084282	dre-circ-1592	0	-	24	0	0	0	0
chr19	39083146	39084387	dre-circ-1593	0	-	15	0	0	0	0
chr19	39092082	39092208	dre-circ-1594	0	+	4	0	0	0	0
chr19	39094451	39094635	dre-circ-1595	0	-	2	0	0	0	0
chr19	39096833	39096937	dre-circ-1596	0	+	2	0	0	0	0
chr19	39099850	39099954	dre-circ-1597	0	-	10	0	0	0	0
chr19	39101248	39104589	dre-circ-1598	0	-	2	0	0	0	0
chr19	39108282	39108400	dre-circ-1599	0	-	2	0	0	0	0
chr19	39113507	39114698	dre-circ-1600	0	+	3	0	0	0	0
chr19	41049368	41053177	dre-circ-3387	0	+	0	4	3	0	0
chr19	41068792	41120259	dre-circ-1601	0	+	3	2	2	3	16
chr19	41088676	41089826	dre-circ-3388	0	+	2	0	4	4	0
chr19	41088676	41097466	dre-circ-3389	0	+	2	0	2	0	0
chr19	41143189	41143977	dre-circ-3390	0	-	0	0	0	0	6
chr19	41148138	41157000	dre-circ-1602	0	-	0	0	0	9	0
chr19	41151291	41157195	dre-circ-1603	0	-	5	0	82	7	59
chr19	41151506	41158547	dre-circ-1604	0	-	0	0	15	0	17
chr19	41152425	41158820	dre-circ-1605	0	-	0	0	21	3	0
chr19	41863724	41863995	dre-circ-3391	0	-	2	5	0	0	0
chr19	43237476	43242127	dre-circ-1606	0	+	2	0	0	0	0
chr19	43939253	43940947	dre-circ-3392	0	-	0	0	0	3	0
chr19	44158500	44165377	dre-circ-3393	0	-	0	0	2	0	0
chr19	44165285	44173968	dre-circ-3394	0	-	0	0	0	6	0
chr19	44169266	44174695	dre-circ-3395	0	-	0	0	0	2	0
chr19	44657598	44659723	dre-circ-3396	0	+	2	0	0	0	0
chr19	45183326	45186894	dre-circ-3397	0	-	0	0	0	0	2
chr19	45288520	45291824	dre-circ-1607	0	+	119	36	1403	812	79
chr19	45288786	45294179	dre-circ-3398	0	+	448	71	1385	208	834
chr19	45289170	45294550	dre-circ-1608	0	+	73	3	175	68	172
chr19	45289624	45297490	dre-circ-1609	0	+	163	5	561	195	368
chr19	45482167	45489661	dre-circ-1610	0	+	0	4	2	0	0
chr19	45482167	45498223	dre-circ-1611	0	+	2	3	0	0	0
chr19	46099691	46176899	dre-circ-1612	0	-	0	0	0	0	2
chr1	9478433	9478663	dre-circ-27	0	+	2	0	0	0	0
chr19	47910449	47911560	dre-circ-3399	0	+	3	0	0	0	2
chr19	47910449	47913539	dre-circ-1613	0	+	0	0	0	3	0
chr19	49199929	49213528	dre-circ-1614	0	+	3	0	0	0	0
chr19	49200106	49208863	dre-circ-1615	0	+	11	0	14	5	16
chr19	49203875	49213938	dre-circ-1616	0	+	0	0	0	0	5
chr19	49204530	49214333	dre-circ-3400	0	+	6	0	4	0	4
chr19	49852759	49858637	dre-circ-3401	0	-	153	0	51	1049	12
chr19	49853397	49858841	dre-circ-1617	0	-	0	0	0	3	6
chr19	49855247	49859993	dre-circ-3402	0	-	40	3	92	469	47
chr19	49960792	49963577	dre-circ-1618	0	-	0	0	0	2	0
chr19	49992767	49998286	dre-circ-3403	0	+	3	0	0	0	8
chr19	5275533	5277421	dre-circ-3404	0	+	3	0	0	0	0
chr19	5528055	5529127	dre-circ-3405	0	+	0	0	0	2	2
chr19	584650	590052	dre-circ-1520	0	-	2	0	0	0	0
chr19	589624	590052	dre-circ-3406	0	-	6	0	0	0	0
chr19	5975454	5987999	dre-circ-1525	0	-	0	0	0	0	3
chr19	5992232	6003348	dre-circ-3407	0	-	3	0	0	2	14
chr19	676577	679070	dre-circ-1521	0	+	0	0	0	2	0
chr19	7602965	7605482	dre-circ-3408	0	-	4	0	0	0	2
chr19	7663935	7690342	dre-circ-1526	0	-	0	0	0	0	16
chr19	7665718	7692951	dre-circ-3409	0	-	0	0	3	0	5
chr19	7671946	7721733	dre-circ-1527	0	+	2	0	0	0	0
chr19	8143698	8146224	dre-circ-1528	0	-	2	0	0	0	0
chr19	8305928	8318219	dre-circ-1529	0	+	0	0	0	0	4
chr19	8309266	8321515	dre-circ-1530	0	-	0	0	0	0	11
chr19	8423330	8423562	dre-circ-3410	0	+	2	0	0	0	0
chr19	8467742	8519285	dre-circ-1531	0	-	2	0	0	0	0
chr19	8469706	8494092	dre-circ-1532	0	+	0	0	0	0	2
chr19	8471684	8533368	dre-circ-3411	0	+	5	0	0	0	0
chr19	8482883	8506728	dre-circ-1533	0	+	0	0	0	0	3
chr19	8496186	8533368	dre-circ-1534	0	+	13	0	0	0	0
chr19	8500973	8539148	dre-circ-1535	0	+	2	0	0	0	0
chr19	8503723	8541603	dre-circ-1536	0	+	0	0	0	0	5
chr19	8505847	8541869	dre-circ-1537	0	+	4	0	2	0	0
chr19	8508241	8545219	dre-circ-3412	0	+	2	0	0	0	0
chr19	8508573	8545434	dre-circ-3413	0	+	0	0	0	0	6
chr19	8552283	8564056	dre-circ-1538	0	+	0	0	0	0	3
chr19	8553379	8564892	dre-circ-1539	0	+	3	0	0	0	0
chr19	9454298	9459708	dre-circ-1540	0	-	0	0	0	0	2
chr19	9698032	9700100	dre-circ-3414	0	+	2	0	0	0	0
chr19	9742331	9746122	dre-circ-3415	0	+	2	0	0	0	2
chr20	10727175	10733291	dre-circ-1635	0	+	0	0	35	225	0
chr20	10727542	10733666	dre-circ-1636	0	+	0	0	26	144	0
chr20	10727766	10733910	dre-circ-1637	0	+	0	0	35	118	0
chr20	1102298	1105380	dre-circ-1621	0	-	0	0	0	0	2
chr20	12990786	12990992	dre-circ-3416	0	-	0	0	0	0	18
chr20	13015115	13040656	dre-circ-1638	0	-	2	0	0	0	0
chr20	13182517	13186030	dre-circ-3417	0	+	0	0	2	0	0
chr20	14080846	14173538	dre-circ-1639	0	-	0	2	0	0	0
chr20	1424222	1435975	dre-circ-1622	0	+	0	0	0	19	0
chr20	1427332	1439414	dre-circ-1623	0	+	0	0	0	12	0
chr20	1427789	1441131	dre-circ-1624	0	+	0	0	5	0	0

chr20	14803075	14803528	dre-circ-1640	0	-	2	0	0	0	0
chr20	14863956	14926179	dre-circ-1641	0	+	0	0	0	0	6
chr20	14898467	14946766	dre-circ-1642	0	+	3	0	6	6	25
chr20	14898467	14954172	dre-circ-1643	0	+	2	3	2	0	3
chr20	15091590	15100493	dre-circ-3418	0	-	0	0	5	0	10
chr20	15092065	15102687	dre-circ-1644	0	-	0	0	2	0	0
chr20	15095468	15104701	dre-circ-1645	0	-	0	0	5	0	6
chr20	153719	154628	dre-circ-3419	0	-	2	0	0	0	0
chr20	15579888	15580077	dre-circ-1646	0	+	0	2	0	0	0
chr20	16037651	16044558	dre-circ-1647	0	+	0	0	0	2	0
chr20	16116721	16121595	dre-circ-1648	0	-	0	0	0	0	2
chr20	16482068	16484682	dre-circ-1649	0	-	2	0	0	0	0
chr20	18418403	18422256	dre-circ-1650	0	-	0	2	0	2	0
chr20	19504572	19504710	dre-circ-1651	0	+	0	0	0	0	2
chr20	19515100	19541750	dre-circ-1652	0	-	0	0	0	0	2
chr20	19602824	19604714	dre-circ-1653	0	-	2	0	0	0	0
chr20	20833700	20841008	dre-circ-1654	0	+	0	0	0	0	2
chr20	20859769	20859870	dre-circ-1655	0	+	0	0	0	0	2
chr20	21808084	21808965	dre-circ-1656	0	+	0	0	0	0	2
chr20	21900017	21913509	dre-circ-3420	0	-	0	0	4	0	0
chr20	22160537	22202491	dre-circ-1657	0	+	2	0	0	0	0
chr20	23196400	23205589	dre-circ-1658	0	-	0	0	2	0	0
chr20	23320015	23324750	dre-circ-3421	0	+	6	0	0	0	0
chr20	2332276	2340826	dre-circ-3422	0	-	0	0	0	0	10
chr20	2332276	2341615	dre-circ-3423	0	-	2	0	0	0	0
chr20	23441054	23443998	dre-circ-3424	0	-	2	0	0	0	0
chr20	23829848	23830928	dre-circ-1659	0	-	0	0	0	2	0
chr20	24309910	24320982	dre-circ-3425	0	+	5	0	22	6	22
chr20	2459372	2461915	dre-circ-3426	0	+	3	0	0	0	0
chr20	25567113	25568138	dre-circ-1660	0	-	0	0	0	0	2
chr20	25591588	25608990	dre-circ-1661	0	+	0	0	0	2	0
chr20	25620763	25621463	dre-circ-3427	0	+	0	0	2	0	5
chr20	2624400	2625271	dre-circ-3428	0	+	0	0	0	0	2
chr20	26915265	26930171	dre-circ-1662	0	+	0	0	4	0	12
chr20	26915265	26947472	dre-circ-1663	0	+	0	0	3	0	10
chr20	26915468	26930772	dre-circ-1664	0	+	0	0	0	0	2
chr20	26915746	26948009	dre-circ-1665	0	+	0	0	0	0	3
chr20	26915996	26931364	dre-circ-1666	0	+	0	0	0	2	2
chr20	26998812	27000501	dre-circ-3429	0	+	0	0	5	3	0
chr20	26998812	27003399	dre-circ-3430	0	+	0	0	2	0	0
chr20	27179462	27182237	dre-circ-1667	0	-	2	0	0	0	0
chr20	27350082	27351377	dre-circ-3431	0	-	0	0	0	2	0
chr20	27360365	27361919	dre-circ-3432	0	+	0	0	0	0	4
chr20	28308335	28314507	dre-circ-1668	0	-	0	0	0	0	2
chr20	28313652	28314507	dre-circ-3433	0	-	3	0	0	2	0
chr20	29079865	29109507	dre-circ-1669	0	+	0	0	0	0	2
chr20	2911578	2919381	dre-circ-1625	0	-	2	0	0	0	0
chr20	2919303	2924446	dre-circ-3434	0	-	7	0	0	0	0
chr20	2932657	2933459	dre-circ-3435	0	-	4	0	0	0	0
chr20	3009329	3010276	dre-circ-1626	0	+	2	0	2	0	5
chr20	3009495	3010449	dre-circ-1627	0	+	0	0	2	0	2
chr20	3039724	3043220	dre-circ-1628	0	-	0	2	0	0	0
chr20	3061427	3063193	dre-circ-3436	0	-	7	6	14	0	7
chr20	33171146	33171406	dre-circ-3437	0	-	0	0	0	2	0
chr20	33182927	33185866	dre-circ-1670	0	-	0	0	0	2	0
chr20	33513383	33514730	dre-circ-3438	0	-	8	0	6	4	2
chr20	33996875	33998983	dre-circ-1671	0	-	0	0	2	0	0
chr20	33997304	33998642	dre-circ-1672	0	-	0	0	3	0	0
chr20	33997443	33999661	dre-circ-3439	0	-	0	0	12	0	0
chr20	33998792	33999661	dre-circ-1673	0	-	0	0	25	17	0
chr20	33998948	33999933	dre-circ-1674	0	-	0	0	5	0	0
chr20	34012780	34016979	dre-circ-1675	0	+	0	0	0	0	276
chr20	34013761	34017967	dre-circ-1676	0	+	0	0	0	0	55
chr20	34887864	34887998	dre-circ-1677	0	+	0	0	2	0	0
chr20	35614929	35620306	dre-circ-1678	0	-	0	0	0	0	12
chr20	35622910	35623288	dre-circ-1679	0	-	0	0	0	0	10
chr20	35622910	35626520	dre-circ-1680	0	-	0	0	0	0	4
chr20	35623142	35626035	dre-circ-1681	0	-	0	0	0	0	12
chr20	35626521	35637052	dre-circ-1682	0	-	0	0	0	0	12
chr20	35626521	35638212	dre-circ-1683	0	-	0	0	0	0	6
chr20	35627037	35640340	dre-circ-1684	0	-	0	0	0	0	7
chr20	35628975	35631541	dre-circ-1685	0	-	4	0	0	0	46
chr20	35628975	35640340	dre-circ-3440	0	-	0	0	0	0	4
chr20	35634203	35634649	dre-circ-1686	0	-	3	0	0	0	4
chr20	35635357	35637052	dre-circ-1687	0	-	4	0	0	0	7
chr20	35635357	35641043	dre-circ-1688	0	-	2	0	0	0	17
chr20	35635357	35644356	dre-circ-1689	0	-	5	0	0	0	27
chr20	35636565	35637052	dre-circ-1690	0	-	0	0	0	0	6
chr20	35636565	35639699	dre-circ-1691	0	-	0	0	0	0	9
chr20	35636565	35640320	dre-circ-1692	0	-	0	0	0	0	2
chr20	35636906	35642399	dre-circ-1693	0	-	0	0	0	0	7
chr20	35639248	35720283	dre-circ-1694	0	-	0	0	0	0	2
chr20	35639855	35641043	dre-circ-1695	0	-	0	0	0	0	5
chr20	35642253	35644356	dre-circ-3441	0	-	5	0	0	0	13
chr20	35643852	35668082	dre-circ-1696	0	-	0	0	0	0	11
chr20	35643852	35723235	dre-circ-1697	0	-	3	0	0	0	0
chr20	35644210	35673321	dre-circ-1698	0	-	0	0	0	0	2

chr20	35644210	35704129	dre-circ-1699	0	-	4	0	0	0	0
chr20	35644210	35706743	dre-circ-1700	0	-	2	0	0	0	5
chr20	35655599	35662100	dre-circ-1701	0	-	3	0	0	0	0
chr20	35659272	35662100	dre-circ-1702	0	-	0	0	0	0	10
chr20	35659272	35666187	dre-circ-1703	0	-	0	0	0	0	3
chr20	35659272	35671844	dre-circ-1704	0	-	0	0	0	0	12
chr20	35667197	35672436	dre-circ-1705	0	-	0	0	0	0	3
chr20	35667197	35690924	dre-circ-1706	0	-	0	0	0	0	4
chr20	35667197	35708114	dre-circ-1707	0	-	0	0	0	0	9
chr20	35667197	35716858	dre-circ-1708	0	-	0	0	0	0	15
chr20	35667936	35714174	dre-circ-1709	0	-	0	0	0	0	9
chr20	35673175	35676380	dre-circ-1710	0	-	0	0	0	0	4
chr20	35673175	35688704	dre-circ-1711	0	-	0	0	0	0	4
chr20	35673175	35719838	dre-circ-1712	0	-	0	0	0	0	4
chr20	35673500	35689542	dre-circ-1713	0	-	0	0	0	0	13
chr20	35679701	35683541	dre-circ-1714	0	-	0	0	0	0	16
chr20	35679701	35686020	dre-circ-1715	0	-	0	0	0	0	8
chr20	35691962	35722868	dre-circ-1716	0	-	0	0	0	0	7
chr20	35703643	35722839	dre-circ-1717	0	-	0	0	0	0	2
chr20	35706597	35719838	dre-circ-1718	0	-	0	0	0	0	4
chr20	35707411	35712769	dre-circ-1719	0	-	2	0	0	0	6
chr20	35723944	35762594	dre-circ-1720	0	-	0	0	0	0	79
chr20	35724780	35769818	dre-circ-1721	0	-	0	0	0	0	11
chr20	35759032	35760136	dre-circ-1722	0	-	0	0	0	0	10
chr20	36473890	36474251	dre-circ-1723	0	-	0	0	0	2	0
chr20	39100900	39106066	dre-circ-1724	0	-	0	0	2	0	0
chr20	42060089	42080876	dre-circ-1725	0	-	0	0	0	0	2
chr20	42354572	42373010	dre-circ-3442	0	-	0	0	0	0	3
chr20	43228156	43240878	dre-circ-3443	0	-	0	0	5	0	0
chr20	433727	434128	dre-circ-3444	0	-	4	0	0	0	0
chr20	4339467	4341546	dre-circ-3445	0	+	0	0	0	0	3
chr20	44810415	44812707	dre-circ-3446	0	+	2	0	0	0	0
chr20	44812003	44812707	dre-circ-1726	0	+	6	0	0	0	0
chr20	457173	457443	dre-circ-3447	0	-	8	0	2	0	0
chr20	45881617	45888602	dre-circ-3448	0	-	0	0	2	0	0
chr20	46681005	46684197	dre-circ-1727	0	+	2	0	0	0	0
chr20	47101725	47127680	dre-circ-1728	0	-	3	0	0	0	0
chr20	47185706	47186539	dre-circ-3449	0	+	2	0	0	10	0
chr20	47734405	47737550	dre-circ-1729	0	-	0	0	0	0	2
chr20	50334159	50342735	dre-circ-3450	0	+	3	0	6	0	0
chr20	51534613	51534785	dre-circ-1730	0	+	2	0	0	0	0
chr20	51681665	51686502	dre-circ-3451	0	-	2	0	0	0	0
chr20	51711746	51714567	dre-circ-3452	0	-	12	0	4	0	2
chr20	52758353	52760004	dre-circ-1731	0	-	0	2	0	0	0
chr20	5279328	5296702	dre-circ-1629	0	-	6	0	6	0	0
chr20	52813214	52833858	dre-circ-1732	0	+	4	0	0	0	19
chr20	5282978	5299332	dre-circ-1630	0	-	6	0	10	0	0
chr20	5286500	5302010	dre-circ-1631	0	-	0	0	0	0	2
chr20	5299093	5318020	dre-circ-1632	0	-	0	0	0	0	2
chr20	53228507	53261320	dre-circ-3453	0	-	11	0	21	5	3
chr20	53261912	53262015	dre-circ-1733	0	-	0	0	2	0	0
chr20	53289706	53292230	dre-circ-3454	0	-	3	0	0	0	0
chr20	53537695	53537973	dre-circ-1734	0	+	0	0	4	0	0
chr20	53931928	53932089	dre-circ-1735	0	+	0	0	4	0	0
chr20	54325342	54338813	dre-circ-3455	0	-	9	9	15	0	16
chr20	54326115	54340161	dre-circ-1736	0	-	3	3	7	0	6
chr20	54329598	54342889	dre-circ-1737	0	-	6	6	6	0	6
chr20	54373576	54380074	dre-circ-1738	0	+	0	0	0	0	112
chr20	54373576	54383293	dre-circ-1739	0	+	0	0	0	0	106
chr20	54373576	54390027	dre-circ-1740	0	+	0	0	0	0	120
chr20	54380638	54383755	dre-circ-1741	0	+	0	0	0	0	10
chr20	54380638	54390491	dre-circ-1742	0	+	0	0	0	0	16
chr20	54380894	54383962	dre-circ-1743	0	+	0	0	0	0	11
chr20	54550036	54554835	dre-circ-1744	0	+	0	0	0	0	80
chr20	54550036	54559042	dre-circ-1745	0	+	0	0	0	0	75
chr20	54550036	54564426	dre-circ-1746	0	+	0	0	0	0	74
chr20	54550036	54568768	dre-circ-1747	0	+	0	0	0	0	79
chr20	54550036	54572582	dre-circ-1748	0	+	0	0	0	0	58
chr20	54555046	54559197	dre-circ-3456	0	+	0	0	0	0	65
chr20	54555046	54564583	dre-circ-1749	0	+	0	0	0	0	77
chr20	54559268	54564583	dre-circ-1750	0	+	0	0	0	0	140
chr20	54559268	54568913	dre-circ-1751	0	+	0	0	0	0	27
chr20	54564649	54568913	dre-circ-1752	0	+	0	0	0	0	4
chr20	54568838	54572582	dre-circ-1753	0	+	0	0	0	0	73
chr20	5473948	5478053	dre-circ-1633	0	+	2	0	0	0	0
chr20	54767142	54771372	dre-circ-3457	0	-	2	0	0	0	0
chr20	55029269	55067316	dre-circ-3458	0	+	0	2	0	3	0
chr20	598010	600927	dre-circ-1619	0	+	0	0	0	2	0
chr20	6415027	6416002	dre-circ-3459	0	-	6	0	2	0	0
chr20	6707023	6718399	dre-circ-3460	0	+	0	0	2	0	0
chr20	6707023	6725381	dre-circ-3461	0	+	0	0	5	0	0
chr20	6717959	6718399	dre-circ-1634	0	+	0	0	0	2	0
chr20	7050500	7051069	dre-circ-3462	0	-	44	3	13	3	6
chr20	718684	721457	dre-circ-1620	0	+	2	0	0	0	0
chr20	8264914	8269412	dre-circ-3463	0	-	0	0	0	0	3
chr20	8353598	8363989	dre-circ-3464	0	-	0	3	0	0	0
chr2	10335021	10335425	dre-circ-3465	0	-	4	0	0	0	0

chr2	10936473	10937064	dre-circ-144	0	+	0	0	0	0	3
chr21	10321151	10321274	dre-circ-1770	0	+	0	0	2	0	0
chr21	11341102	11342060	dre-circ-3466	0	+	4	2	0	0	0
chr21	11383446	11386031	dre-circ-1771	0	+	0	2	0	0	0
chr21	11454085	11457043	dre-circ-3467	0	+	5	2	6	0	4
chr21	11566742	11568550	dre-circ-3468	0	+	2	0	0	0	0
chr21	11673247	11675981	dre-circ-3469	0	-	0	2	0	0	0
chr21	12192113	12198251	dre-circ-1772	0	-	0	2	0	0	0
chr21	13503582	13521232	dre-circ-1773	0	-	0	0	0	0	11
chr21	13503582	13539650	dre-circ-1774	0	-	3	0	0	0	2
chr21	13504916	13522727	dre-circ-1775	0	+	0	0	0	0	2
chr21	13505383	13522832	dre-circ-1776	0	+	2	0	0	0	17
chr21	13509642	13531442	dre-circ-1777	0	+	0	0	0	0	34
chr21	13526863	13561782	dre-circ-1778	0	+	0	0	0	0	4
chr21	16558171	16577590	dre-circ-1779	0	-	0	2	0	0	0
chr2	116709	117898	dre-circ-3470	0	+	9	0	0	4	17
chr21	1672138	1679276	dre-circ-3471	0	+	0	3	0	2	0
chr21	18117812	18121605	dre-circ-1780	0	-	0	0	0	2	0
chr21	18508045	18510570	dre-circ-1781	0	-	0	0	0	0	2
chr21	19601909	19671560	dre-circ-3472	0	+	0	0	0	3	0
chr21	213163	214281	dre-circ-1754	0	-	2	0	0	0	0
chr21	21437483	21536159	dre-circ-1782	0	-	0	2	0	0	0
chr21	215577	216119	dre-circ-1755	0	-	2	0	0	0	0
chr21	22170826	22175913	dre-circ-3473	0	-	0	0	6	0	0
chr21	23857696	23894665	dre-circ-1783	0	-	0	2	0	0	0
chr21	24660952	24662082	dre-circ-3474	0	-	2	0	0	0	0
chr21	2628512	2629427	dre-circ-3475	0	+	3	0	0	2	2
chr21	26885684	26889091	dre-circ-1784	0	-	0	0	0	2	0
chr21	27753660	27758192	dre-circ-1785	0	-	0	0	2	0	0
chr21	2861453	2864711	dre-circ-1756	0	+	0	0	0	0	2
chr21	29178294	29186147	dre-circ-1786	0	-	0	0	0	2	0
chr2	12934593	12936363	dre-circ-3476	0	+	0	0	3	2	0
chr21	29500122	29500605	dre-circ-3477	0	+	2	0	0	0	0
chr21	2964459	2968677	dre-circ-3478	0	+	8	5	0	3	9
chr21	3197043	3199359	dre-circ-1757	0	-	0	0	0	0	2
chr21	32470971	32472458	dre-circ-3479	0	+	2	3	6	0	0
chr21	32737747	32777120	dre-circ-1787	0	-	0	0	0	0	2
chr21	32989077	32992496	dre-circ-3480	0	-	2	0	0	0	0
chr21	33220271	33220819	dre-circ-3481	0	-	0	0	0	0	3
chr21	33822245	33822657	dre-circ-1788	0	+	0	0	2	0	0
chr21	33858749	33864018	dre-circ-1789	0	-	2	0	0	0	0
chr21	34013657	34014167	dre-circ-1790	0	+	2	0	0	0	0
chr21	34374195	34385293	dre-circ-3482	0	-	0	0	2	0	0
chr21	34383090	34385293	dre-circ-3483	0	-	0	0	3	0	0
chr2	13459937	13461322	dre-circ-3484	0	+	0	0	5	0	0
chr2	13459937	13465243	dre-circ-3485	0	+	0	0	2	0	0
chr2	13542865	13543279	dre-circ-3486	0	+	2	3	0	0	0
chr21	35847101	35867663	dre-circ-3487	0	-	0	0	0	0	3
chr21	36977249	36983955	dre-circ-3488	0	-	3	0	0	0	0
chr21	36983486	36983955	dre-circ-3489	0	-	18	2	11	0	0
chr21	36983486	36989645	dre-circ-3490	0	-	0	0	0	2	0
chr21	37684438	37686479	dre-circ-3491	0	-	4	0	0	0	0
chr21	37684446	37686479	dre-circ-3492	0	-	24	0	0	0	8
chr21	38488451	38488779	dre-circ-3493	0	-	4	2	0	0	0
chr21	38849929	38871649	dre-circ-1791	0	-	0	0	0	3	0
chr21	38851513	38875026	dre-circ-1792	0	-	0	0	0	8	0
chr21	38994853	39002098	dre-circ-3494	0	-	3	0	7	0	2
chr2	13950764	13956256	dre-circ-3495	0	-	0	0	0	2	0
chr21	3966090	3966349	dre-circ-1758	0	-	0	0	0	2	0
chr21	39666202	39666723	dre-circ-1793	0	+	7	0	0	0	0
chr21	39666202	39667224	dre-circ-1794	0	+	3	0	0	0	0
chr21	39724459	39732362	dre-circ-3496	0	-	2	0	0	0	0
chr21	40406512	40414262	dre-circ-1795	0	+	0	0	0	0	2
chr21	40675959	40683003	dre-circ-3497	0	+	0	3	0	0	0
chr21	40675959	40683073	dre-circ-3498	0	+	0	16	0	0	0
chr21	40675959	40683756	dre-circ-3499	0	+	0	12	0	0	0
chr21	40675959	40683855	dre-circ-3500	0	+	0	2	0	0	0
chr21	40921144	40921656	dre-circ-1796	0	+	0	2	0	0	0
chr21	41626015	41628540	dre-circ-3501	0	+	4	0	0	0	0
chr21	41716870	41717709	dre-circ-1797	0	-	0	0	0	0	2
chr21	41717008	41717847	dre-circ-1798	0	+	0	0	0	0	3
chr21	41731060	41732046	dre-circ-1799	0	-	0	0	0	0	2
chr21	42037449	42038765	dre-circ-1800	0	+	0	0	0	0	83
chr21	42055632	42060982	dre-circ-1801	0	-	0	0	0	0	254
chr21	42055632	42071246	dre-circ-1802	0	-	0	0	0	0	11
chr21	42060062	42069858	dre-circ-1803	0	+	0	0	0	0	12
chr21	42060062	42070281	dre-circ-1804	0	+	0	0	0	0	44
chr21	42261456	42268227	dre-circ-1805	0	+	0	0	0	2	0
chr21	42264572	42267441	dre-circ-3502	0	+	0	3	14	23	2
chr21	432173	490598	dre-circ-3503	0	-	2	0	0	2	11
chr21	43627993	43636803	dre-circ-3504	0	-	4	0	2	0	0
chr21	44072980	44074924	dre-circ-3505	0	+	10	0	0	0	0
chr21	44261577	44261953	dre-circ-3506	0	-	4	0	2	0	0
chr21	4484894	4485240	dre-circ-1759	0	+	3	4	0	0	0
chr21	4562846	4563527	dre-circ-1760	0	+	0	0	0	0	7
chr21	4914747	4917819	dre-circ-3507	0	-	0	0	0	2	0
chr21	4943156	4945796	dre-circ-1761	0	-	2	0	0	0	0

chr21	5233041	5234779	dre-circ-1762	0	-	2	0	0	0	0
chr21	5437879	5439194	dre-circ-1763	0	-	2	0	0	0	0
chr21	5437881	5439177	dre-circ-1764	0	-	2	0	0	0	0
chr21	584572	592260	dre-circ-3508	0	+	0	0	0	0	4
chr2	16077678	16094663	dre-circ-3509	0	-	0	0	3	0	0
chr2	16083258	16094663	dre-circ-3510	0	-	2	2	2	0	0
chr2	1679873	1680586	dre-circ-130	0	+	0	0	0	0	2
chr2	17138966	17140726	dre-circ-3511	0	-	2	0	8	0	0
chr2	17138966	17175331	dre-circ-3512	0	-	0	0	3	0	0
chr2	17290648	17290799	dre-circ-145	0	-	2	0	0	0	0
chr21	7612882	7615139	dre-circ-3513	0	-	0	0	2	2	0
chr21	7618724	7622934	dre-circ-1765	0	-	0	0	0	2	0
chr21	7868883	7872140	dre-circ-3514	0	-	0	0	2	0	0
chr21	7986154	7986454	dre-circ-1766	0	+	2	0	0	0	0
chr2	181406	182065	dre-circ-3515	0	-	9	0	0	0	0
chr21	8179279	8179704	dre-circ-1767	0	-	2	0	0	0	0
chr2	1845409	1849440	dre-circ-131	0	-	0	0	0	2	0
chr21	8770540	8770659	dre-circ-1768	0	+	2	0	0	0	0
chr21	8843922	8850333	dre-circ-3516	0	+	0	0	0	0	4
chr2	19190680	19194303	dre-circ-146	0	-	2	0	0	0	0
chr21	9479068	9498534	dre-circ-3517	0	-	0	0	3	0	0
chr21	9854956	9942831	dre-circ-1769	0	-	0	0	0	0	2
chr2	20605809	20629232	dre-circ-147	0	-	12	0	0	0	0
chr2	20613209	20636621	dre-circ-148	0	-	7	0	0	0	7
chr2	20616310	20636872	dre-circ-149	0	-	16	0	0	0	0
chr22	10004353	10010030	dre-circ-3518	0	-	0	0	0	0	12
chr22	10039348	10043323	dre-circ-3519	0	-	0	0	0	0	3
chr22	10677204	10677747	dre-circ-1840	0	-	2	0	0	0	0
chr22	10685088	10685421	dre-circ-1841	0	+	0	2	0	0	0
chr22	11114610	11115158	dre-circ-3520	0	-	24	0	0	0	0
chr22	11266017	11266358	dre-circ-1842	0	-	2	0	0	0	0
chr22	11867266	11885824	dre-circ-1843	0	+	61	0	0	0	157
chr22	11868048	11886838	dre-circ-1844	0	+	32	0	0	0	85
chr22	11868291	11888680	dre-circ-1845	0	+	107	0	28	9	302
chr22	11868580	11888884	dre-circ-1846	0	+	35	0	0	0	99
chr22	11868889	11889256	dre-circ-1847	0	+	20	0	7	0	47
chr22	11978578	11981871	dre-circ-3521	0	+	0	0	0	0	3
chr22	14062261	14084282	dre-circ-1848	0	+	0	0	2	0	0
chr22	14359449	14367775	dre-circ-1849	0	-	2	0	0	0	0
chr22	14395571	14395950	dre-circ-3522	0	-	2	0	3	0	0
chr22	15462335	15483670	dre-circ-1850	0	+	0	0	0	2	0
chr22	15730655	15732968	dre-circ-3523	0	-	0	0	8	0	0
chr22	161167	166147	dre-circ-3524	0	+	2	0	0	0	0
chr22	16244990	16312615	dre-circ-1851	0	+	0	0	2	0	0
chr22	1639732	1640912	dre-circ-3525	0	+	0	0	0	0	2
chr22	1639732	1640916	dre-circ-3526	0	+	0	0	0	0	2
chr22	16447074	16463196	dre-circ-1852	0	-	0	0	0	2	0
chr22	1763071	1768396	dre-circ-1818	0	+	0	0	0	0	5
chr22	17680484	17685131	dre-circ-1853	0	-	40	0	0	0	0
chr22	17680484	17700708	dre-circ-1854	0	-	28	0	6	0	0
chr22	17680484	17709129	dre-circ-1855	0	-	28	0	5	0	0
chr22	17680484	17717742	dre-circ-1856	0	-	2	0	0	0	0
chr22	17691955	17700708	dre-circ-1857	0	-	0	0	2	0	0
chr22	17691955	17717742	dre-circ-1858	0	-	2	0	4	0	4
chr22	17711737	17720739	dre-circ-1859	0	-	3	0	2	0	0
chr22	17795074	17795637	dre-circ-1860	0	-	0	0	0	0	2
chr22	1780853	1785604	dre-circ-1819	0	+	0	0	0	0	3
chr22	1788779	1789030	dre-circ-1820	0	+	0	0	3	3	0
chr22	17979495	17984471	dre-circ-3527	0	-	25	0	0	0	0
chr22	1845110	1854378	dre-circ-1821	0	+	0	0	2	0	2
chr22	18577301	18577559	dre-circ-3528	0	+	3	0	0	0	0
chr22	19271309	19279711	dre-circ-3529	0	-	3	0	7	0	0
chr22	2014549	2040895	dre-circ-3530	0	+	0	0	6	0	0
chr22	20455369	20464823	dre-circ-1861	0	+	0	0	0	0	3
chr22	20456749	20469146	dre-circ-1862	0	-	2	0	0	0	0
chr22	20457456	20469751	dre-circ-1863	0	+	0	0	0	0	3
chr22	20459113	20471331	dre-circ-1864	0	+	0	0	0	0	2
chr22	20460121	20472324	dre-circ-1865	0	+	0	0	4	0	3
chr22	20482591	20483399	dre-circ-3531	0	-	0	2	3	0	0
chr22	21134511	21136099	dre-circ-3532	0	-	2	0	0	0	0
chr22	2122414	2122702	dre-circ-3533	0	+	7	0	2	0	0
chr22	21330956	21352542	dre-circ-3534	0	-	4	0	0	0	0
chr22	21584437	21584573	dre-circ-1866	0	-	0	0	0	0	2
chr22	2195719	2196148	dre-circ-3535	0	-	3	0	0	0	0
chr22	22551304	22552172	dre-circ-1867	0	-	0	0	2	0	0
chr22	2397152	2398514	dre-circ-3536	0	-	3	0	0	0	0
chr22	24292915	24303714	dre-circ-1868	0	+	0	0	0	4	0
chr22	24442931	24452123	dre-circ-1869	0	-	0	0	2	0	0
chr22	24442931	24461197	dre-circ-3537	0	-	0	0	0	0	2
chr22	24709064	24710230	dre-circ-3538	0	-	0	0	0	3	0
chr22	24879654	24904341	dre-circ-1870	0	-	2	0	0	0	0
chr22	25086047	25087129	dre-circ-1871	0	+	2	0	0	0	0
chr22	2510967	2511615	dre-circ-1822	0	+	0	0	2	0	0
chr22	25188979	25191552	dre-circ-3539	0	+	0	5	0	0	0
chr22	25203956	25284104	dre-circ-1872	0	-	0	0	0	0	34
chr22	25204406	25224267	dre-circ-1873	0	-	0	0	22	0	50
chr22	25204406	25257196	dre-circ-1874	0	-	0	0	9	0	53

chr22	25204406	25271501	dre-circ-1875	0	-	0	0	11	0	50
chr22	25204406	25284568	dre-circ-1876	0	-	0	0	0	0	38
chr22	25204694	25224548	dre-circ-1877	0	-	0	0	40	0	50
chr22	25204694	25257476	dre-circ-1878	0	-	0	0	0	0	42
chr22	25204694	25284848	dre-circ-1879	0	-	0	0	68	0	0
chr22	25205739	25225237	dre-circ-1880	0	-	0	0	26	0	54
chr22	25205739	25285550	dre-circ-1881	0	-	0	0	34	0	0
chr22	25206686	25226183	dre-circ-1882	0	-	0	0	0	0	86
chr22	25206982	25226464	dre-circ-1883	0	-	0	0	36	0	52
chr22	25206982	25260189	dre-circ-1884	0	-	0	0	21	0	38
chr22	25206982	25273141	dre-circ-1885	0	-	0	0	32	0	46
chr22	25206982	25286756	dre-circ-1886	0	-	0	0	0	0	61
chr22	25207198	25273375	dre-circ-1887	0	-	0	0	30	0	0
chr22	25207643	25227093	dre-circ-1888	0	-	0	2	42	0	47
chr22	25207643	25260815	dre-circ-1889	0	-	0	0	33	0	33
chr22	25207643	25273773	dre-circ-1890	0	-	0	0	33	0	42
chr22	25207643	25287398	dre-circ-1891	0	-	0	0	37	0	39
chr22	25208160	25274334	dre-circ-1892	0	-	0	0	24	0	26
chr22	25208160	25287915	dre-circ-1893	0	-	0	0	21	0	37
chr22	25208211	25274476	dre-circ-1894	0	-	0	0	3	0	0
chr22	25208938	25228394	dre-circ-1895	0	-	0	0	0	0	103
chr22	25208938	25262091	dre-circ-1896	0	-	0	0	50	0	95
chr22	25208938	25288691	dre-circ-1897	0	-	0	0	24	0	36
chr22	25209676	25262817	dre-circ-1898	0	-	0	0	22	0	47
chr22	25209676	25275883	dre-circ-1899	0	-	0	0	35	0	39
chr22	25210249	25263410	dre-circ-1900	0	-	0	0	7	0	0
chr22	25210499	25229961	dre-circ-1901	0	-	0	0	0	0	4
chr22	25224789	25257653	dre-circ-1902	0	-	0	0	0	0	5
chr22	25226278	25272864	dre-circ-1903	0	-	0	0	8	0	17
chr22	25226278	25286480	dre-circ-1904	0	-	0	0	10	0	11
chr22	25227408	25261008	dre-circ-1905	0	-	0	0	3	2	0
chr22	25227408	25287610	dre-circ-1906	0	-	0	0	0	0	2
chr22	25229249	25262817	dre-circ-1907	0	-	0	0	0	0	9
chr22	25229249	25289401	dre-circ-1908	0	-	0	0	0	0	43
chr22	25229484	25276047	dre-circ-1909	0	-	0	0	0	0	16
chr22	25229484	25289660	dre-circ-1910	0	-	0	0	0	0	15
chr2	22524283	22524616	dre-circ-3540	0	+	2	0	0	0	0
chr22	25257270	25284568	dre-circ-1911	0	-	0	0	0	0	5
chr22	25262911	25275883	dre-circ-1912	0	-	0	0	19	0	0
chr22	25614005	25657881	dre-circ-3541	0	+	0	0	0	0	55
chr22	25614005	25692360	dre-circ-1913	0	+	0	0	0	0	82
chr22	25614005	25710142	dre-circ-1914	0	+	0	0	0	0	40
chr22	25657137	25689994	dre-circ-1915	0	+	0	0	0	0	119
chr22	25657778	25692109	dre-circ-1916	0	+	0	0	0	0	67
chr22	26163245	26256115	dre-circ-1917	0	+	0	0	0	0	10
chr22	26273533	26285088	dre-circ-1918	0	+	0	0	0	0	12
chr22	26273533	26293245	dre-circ-1919	0	+	0	0	0	0	6
chr22	26273533	26304972	dre-circ-1920	0	+	0	0	0	0	11
chr22	26725812	26777180	dre-circ-1921	0	-	0	0	7	0	8
chr22	26737881	26798059	dre-circ-3542	0	-	0	0	4	0	0
chr22	26752997	26812413	dre-circ-1922	0	-	0	0	0	3	0
chr22	26754110	26813056	dre-circ-1923	0	-	0	0	0	2	2
chr22	26754490	26815054	dre-circ-1924	0	-	0	0	0	4	0
chr22	26754756	26815384	dre-circ-3543	0	-	0	0	0	4	8
chr22	26759109	26819042	dre-circ-3544	0	-	0	0	0	6	0
chr22	2707410	2707945	dre-circ-3545	0	+	10	0	6	2	2
chr22	27421862	27422262	dre-circ-3546	0	+	7	0	0	0	0
chr22	276116	307583	dre-circ-1806	0	+	0	0	0	39	0
chr22	283229	302530	dre-circ-1807	0	+	0	0	0	29	0
chr22	283755	329364	dre-circ-1808	0	+	0	0	0	2	0
chr22	284094	303854	dre-circ-1809	0	+	5	0	0	37	0
chr22	292157	308639	dre-circ-1810	0	+	0	0	0	84	0
chr22	295857	310373	dre-circ-1811	0	+	0	0	0	62	0
chr22	295857	339838	dre-circ-1812	0	+	0	0	0	54	0
chr22	296740	311259	dre-circ-1813	0	+	0	0	0	38	0
chr22	296740	340815	dre-circ-1814	0	+	0	0	0	32	0
chr22	296964	311480	dre-circ-1815	0	+	0	0	0	64	0
chr22	31819952	31822673	dre-circ-3547	0	+	0	0	0	2	0
chr2	23191564	23209746	dre-circ-150	0	+	8	3	4	4	8
chr2	23191564	23221260	dre-circ-151	0	+	8	0	3	4	13
chr22	32391789	32393716	dre-circ-3548	0	-	0	0	5	0	0
chr22	32422016	32434213	dre-circ-3549	0	-	0	0	3	0	0
chr22	32710626	32718135	dre-circ-1925	0	+	0	4	0	0	0
chr2	2335116	2335256	dre-circ-3550	0	-	0	3	0	0	0
chr22	33650924	33694152	dre-circ-1926	0	-	0	0	0	0	13
chr22	33656989	33698868	dre-circ-1927	0	-	0	0	3	0	0
chr22	33687494	33701385	dre-circ-1928	0	-	0	0	0	2	0
chr22	3467023	3470297	dre-circ-3551	0	-	0	0	2	0	0
chr2	23576949	23595552	dre-circ-152	0	+	0	0	274	0	0
chr2	23577797	23596711	dre-circ-153	0	+	0	0	5	0	0
chr2	23579345	23598112	dre-circ-154	0	+	0	0	531	0	0
chr2	23579861	23598603	dre-circ-155	0	+	0	0	1608	0	0
chr2	23582807	23599524	dre-circ-3552	0	+	0	0	3703	0	0
chr2	23583584	23600313	dre-circ-156	0	+	0	0	246	0	0
chr2	23583892	23600638	dre-circ-157	0	+	0	0	2	0	0
chr2	23583906	23600638	dre-circ-158	0	+	0	0	1670	0	0
chr2	23584173	23601078	dre-circ-159	0	+	0	0	2	0	0

chr2	23586235	23602793	dre-circ-160	0	+	0	0	3042	0	0
chr2	23587175	23587483	dre-circ-3553	0	+	0	0	3	0	0
chr2	23587175	23603803	dre-circ-161	0	+	0	0	3	0	0
chr2	23588247	23605696	dre-circ-3554	0	+	0	0	2636	2	0
chr22	3728081	3728933	dre-circ-1823	0	+	0	3	5	0	0
chr22	37418670	37419218	dre-circ-3555	0	-	3	2	4	0	0
chr22	38340113	38389790	dre-circ-1929	0	-	0	0	0	0	3
chr22	39625046	39639429	dre-circ-1930	0	-	2	0	0	0	0
chr22	39625215	39640219	dre-circ-1931	0	-	0	0	0	0	3
chr22	39629990	39647103	dre-circ-3556	0	-	6	0	7	0	0
chr22	39631933	39650351	dre-circ-3557	0	-	3	0	2	0	5
chr22	39632680	39652046	dre-circ-3558	0	-	16	0	0	15	0
chr2	2406884	2408358	dre-circ-132	0	+	0	0	2	0	0
chr22	40715168	40718263	dre-circ-3559	0	-	2	0	0	0	3
chr22	40743245	40765549	dre-circ-3560	0	-	0	0	0	0	3
chr22	40858104	40858226	dre-circ-1932	0	-	0	0	0	2	0
chr22	40998416	41000538	dre-circ-1933	0	+	2	0	0	0	0
chr2	24103701	24104096	dre-circ-3561	0	+	0	0	2	0	0
chr22	41268053	41277036	dre-circ-1934	0	+	2	0	0	0	0
chr22	41432343	41487330	dre-circ-1935	0	+	0	4	0	0	0
chr22	41928245	41931043	dre-circ-1936	0	+	0	0	0	2	0
chr22	42032257	42033262	dre-circ-3562	0	+	2	0	0	0	0
chr22	4300830	4303551	dre-circ-3563	0	+	0	3	2	0	0
chr22	4339608	4339712	dre-circ-1824	0	-	0	0	3	0	0
chr2	24492366	24499453	dre-circ-3564	0	+	4	0	0	0	0
chr22	458681	461947	dre-circ-3565	0	-	2	0	0	0	0
chr2	24882539	24888026	dre-circ-162	0	-	0	0	2	0	0
chr2	25393327	25395978	dre-circ-3566	0	+	0	0	6	0	0
chr22	5614673	5614984	dre-circ-3567	0	+	52	0	7	3	0
chr2	25725897	25734115	dre-circ-3568	0	+	3	0	0	0	0
chr22	6224422	6229065	dre-circ-1825	0	-	0	0	0	0	2
chr22	6776374	6789618	dre-circ-3569	0	-	2	0	0	0	0
chr2	26820536	26820725	dre-circ-163	0	+	0	0	0	2	0
chr22	7013876	7046134	dre-circ-3570	0	-	0	0	0	0	3
chr22	728113	764028	dre-circ-1816	0	-	0	0	0	0	2
chr22	7477535	7500945	dre-circ-1826	0	+	0	0	0	2	0
chr22	7477553	7500945	dre-circ-1827	0	+	0	0	9	11	3
chr22	7477553	7510368	dre-circ-1828	0	+	0	0	3	14	4
chr22	7564156	7594595	dre-circ-1829	0	-	0	0	2	0	0
chr2	27790979	27795006	dre-circ-3571	0	-	3	0	0	0	3
chr22	7947096	8028682	dre-circ-1830	0	-	3	0	0	0	0
chr22	805218	808794	dre-circ-1817	0	-	0	0	2	0	0
chr22	8115392	8130936	dre-circ-1831	0	-	0	0	0	0	2
chr22	8115861	8131427	dre-circ-1832	0	-	0	0	2	0	0
chr22	8385860	8423503	dre-circ-1833	0	-	0	0	3	0	0
chr22	8469120	8534144	dre-circ-3572	0	-	0	0	4	0	0
chr22	8485728	8556733	dre-circ-1834	0	-	0	0	2	0	0
chr22	8490322	8559992	dre-circ-1835	0	+	0	2	2	0	0
chr22	8955184	8958350	dre-circ-1836	0	+	0	0	0	0	3
chr22	8956884	8956988	dre-circ-1837	0	+	0	0	0	2	0
chr2	29003877	29012445	dre-circ-3573	0	+	0	0	4	0	0
chr22	9359933	9375599	dre-circ-1838	0	+	2	0	0	0	0
chr22	9367144	9371126	dre-circ-1839	0	+	0	0	2	0	0
chr2	3074400	3078057	dre-circ-3574	0	+	0	0	2	0	0
chr23	10282390	10358168	dre-circ-1953	0	-	0	0	0	0	2
chr23	10283722	10358891	dre-circ-1954	0	-	0	0	0	0	46
chr23	1145819	1185209	dre-circ-3575	0	-	5	2	32	4	43
chr23	1172682	1189492	dre-circ-3576	0	-	7	0	0	2	7
chr23	1185080	1189492	dre-circ-3577	0	-	17	0	22	11	32
chr23	13029839	13030751	dre-circ-3578	0	+	2	0	0	0	0
chr23	13660000	13660381	dre-circ-3579	0	+	2	0	0	0	0
chr23	1384153	1421047	dre-circ-3580	0	-	0	0	0	0	3
chr23	1420419	1468691	dre-circ-3581	0	-	5	0	4	3	10
chr23	15186511	15187691	dre-circ-3582	0	-	0	0	3	3	5
chr2	31631075	31637237	dre-circ-3583	0	-	0	3	0	0	0
chr23	16921024	17002259	dre-circ-1955	0	+	0	0	0	0	2
chr23	16928127	16936026	dre-circ-1956	0	+	2	0	3	0	2
chr23	16928127	17019622	dre-circ-1957	0	+	0	0	0	0	3
chr23	16957248	16978930	dre-circ-1958	0	+	0	0	0	0	2
chr23	16957248	16987978	dre-circ-1959	0	+	0	0	3	0	5
chr23	16958129	16991613	dre-circ-1960	0	+	2	0	3	0	2
chr23	16980630	16987978	dre-circ-1961	0	+	4	0	0	0	0
chr23	17004487	17052256	dre-circ-1962	0	+	0	0	5	0	8
chr23	17053908	17106255	dre-circ-1963	0	+	0	0	0	0	2
chr23	17054125	17075549	dre-circ-1964	0	+	0	0	3	0	0
chr23	17069191	17102807	dre-circ-1965	0	+	0	0	2	0	4
chr23	17078398	17106761	dre-circ-1966	0	+	3	0	2	0	0
chr23	17080987	17108442	dre-circ-3584	0	+	0	0	0	0	5
chr23	17081605	17109056	dre-circ-1967	0	+	2	0	4	0	0
chr23	17082723	17118836	dre-circ-1968	0	+	5	0	4	0	0
chr23	17142239	17159364	dre-circ-1969	0	-	0	0	0	0	10
chr23	17150421	17169595	dre-circ-1970	0	+	0	0	0	0	3
chr23	18022113	18030337	dre-circ-1971	0	-	2	0	0	0	0
chr23	18057672	18059225	dre-circ-3585	0	+	0	0	2	0	0
chr23	18100780	18156767	dre-circ-1972	0	+	0	0	0	0	8
chr23	18101379	18117152	dre-circ-1973	0	+	0	0	0	0	55
chr23	18101648	18117620	dre-circ-1974	0	+	0	0	0	0	8

chr23	18101648	18161195	dre-circ-1975	0	+	0	0	0	0	7
chr23	18103160	18118030	dre-circ-1976	0	+	0	0	0	0	74
chr23	18115824	18153734	dre-circ-1977	0	+	0	0	0	0	4
chr23	18117701	18161195	dre-circ-1978	0	+	0	0	0	3	2
chr23	18185459	18197645	dre-circ-3586	0	-	0	0	0	0	5
chr23	19725900	19726499	dre-circ-3587	0	-	0	0	2	0	2
chr23	20385608	20386726	dre-circ-1979	0	-	0	0	0	2	0
chr23	20502138	20586502	dre-circ-1980	0	-	0	0	16	4	0
chr23	21802569	21804715	dre-circ-3588	0	-	0	0	39	3	0
chr23	22312751	22316761	dre-circ-3589	0	+	3	0	0	0	0
chr23	22801425	22802306	dre-circ-1981	0	-	2	0	0	0	0
chr23	23198732	23200351	dre-circ-1982	0	+	2	0	0	0	0
chr2	32355197	32361181	dre-circ-164	0	-	0	2	0	0	0
chr23	2414512	2423826	dre-circ-1940	0	+	2	0	0	0	0
chr23	24269654	24274538	dre-circ-1983	0	+	0	0	2	0	0
chr23	25267094	25267812	dre-circ-1984	0	+	2	0	0	0	0
chr23	25283183	25283971	dre-circ-1985	0	+	2	0	0	0	0
chr23	26032839	26050080	dre-circ-1986	0	+	2	0	0	0	0
chr23	26234848	26235417	dre-circ-1987	0	-	0	0	0	2	0
chr23	26406720	26415932	dre-circ-1988	0	-	4	0	9	6	11
chr23	26439773	26442636	dre-circ-3590	0	+	2	0	0	2	3
chr23	27545717	27558851	dre-circ-1989	0	-	0	2	0	0	0
chr23	2769645	2781497	dre-circ-3591	0	+	5	0	0	0	0
chr23	2772074	2774743	dre-circ-3592	0	+	6	0	0	0	0
chr23	28044876	28050275	dre-circ-1990	0	-	2	0	0	0	0
chr23	28228849	28242220	dre-circ-1991	0	+	0	0	3	0	0
chr23	2835987	2847282	dre-circ-3593	0	+	0	0	0	2	0
chr23	28948717	28950191	dre-circ-3594	0	+	0	0	2	0	0
chr23	29231346	29237041	dre-circ-3595	0	-	0	0	2	0	0
chr23	296262	298217	dre-circ-1937	0	-	0	0	0	0	2
chr23	29851555	29851668	dre-circ-1992	0	+	0	2	0	0	0
chr23	30176458	30190343	dre-circ-3596	0	+	3	2	2	0	2
chr23	30975114	30975837	dre-circ-3597	0	+	4	0	0	0	2
chr23	31672000	31672413	dre-circ-3598	0	+	7	0	4	2	0
chr23	32269603	32271932	dre-circ-1993	0	-	0	0	0	0	2
chr23	32282975	32291921	dre-circ-3599	0	+	0	0	3	0	0
chr23	32284137	32286291	dre-circ-3600	0	+	0	0	2	0	0
chr23	32284167	32286291	dre-circ-3601	0	+	0	0	28	0	0
chr23	32295387	32295488	dre-circ-1994	0	+	0	0	2	0	0
chr23	32473048	32473701	dre-circ-1995	0	-	0	0	3	0	7
chr23	326791	328995	dre-circ-1938	0	+	2	0	0	0	0
chr23	33676767	33680037	dre-circ-3602	0	-	0	0	5	0	2
chr23	33886470	33890481	dre-circ-1996	0	-	0	0	0	0	2
chr23	34363288	34365107	dre-circ-3603	0	+	5	3	0	0	0
chr23	349638	351998	dre-circ-3604	0	-	2	0	0	0	0
chr23	3516988	3517349	dre-circ-1941	0	+	0	0	0	2	0
chr23	35361164	35362333	dre-circ-3605	0	-	0	0	2	0	0
chr23	35797556	35798375	dre-circ-3606	0	-	0	0	2	0	0
chr2	335857	338026	dre-circ-124	0	+	0	0	0	0	6
chr2	335857	342977	dre-circ-125	0	+	0	0	0	0	6
chr2	335857	348057	dre-circ-126	0	+	0	0	0	0	3
chr23	36614511	36621439	dre-circ-1997	0	-	2	0	0	0	0
chr23	37201807	37206175	dre-circ-1998	0	-	0	0	0	0	2
chr2	33765297	33765837	dre-circ-3607	0	-	4	0	3	0	0
chr23	37887085	37889276	dre-circ-3608	0	+	6	2	6	6	3
chr23	39066245	39072507	dre-circ-1999	0	+	8	10	7	3	19
chr23	39384122	39384297	dre-circ-2000	0	-	0	0	0	0	2
chr23	39579333	39585978	dre-circ-2001	0	+	0	0	2	0	0
chr2	339651	342977	dre-circ-127	0	+	2	0	0	0	0
chr23	39725983	39726563	dre-circ-3609	0	+	0	0	0	0	2
chr23	40106364	40106494	dre-circ-2002	0	+	0	0	2	0	0
chr23	4010659	4012804	dre-circ-1942	0	-	0	0	4	0	2
chr23	401066	405247	dre-circ-3610	0	+	3	0	0	0	0
chr23	40558255	40558496	dre-circ-2003	0	-	0	0	2	0	0
chr23	41038977	41040236	dre-circ-2004	0	+	2	0	0	0	3
chr23	41148039	41154776	dre-circ-3611	0	-	0	0	0	4	0
chr23	41898536	41904065	dre-circ-2005	0	-	0	0	0	0	2
chr2	3429459	3429678	dre-circ-133	0	+	0	0	0	0	3
chr23	42958444	42986438	dre-circ-3612	0	-	0	3	12	0	6
chr23	42999903	43018898	dre-circ-2006	0	-	0	0	4	0	0
chr23	43111953	43112147	dre-circ-2007	0	-	0	0	0	0	4
chr23	4391487	4391829	dre-circ-1943	0	+	2	0	0	0	0
chr2	343957	348057	dre-circ-128	0	+	0	0	4	0	2
chr23	44142832	44145351	dre-circ-3613	0	+	0	0	3	0	0
chr23	44142832	44145356	dre-circ-3614	0	+	2	0	9	0	0
chr23	44244884	44245104	dre-circ-2008	0	-	2	0	2	2	0
chr23	44658586	44659116	dre-circ-2009	0	-	2	0	0	0	0
chr23	44679320	44679427	dre-circ-2010	0	-	0	0	0	0	5
chr23	44683708	44692216	dre-circ-2011	0	+	0	0	0	0	3
chr23	44683708	44692846	dre-circ-2012	0	+	0	0	0	0	10
chr23	44689775	44693571	dre-circ-2013	0	-	0	0	0	0	91
chr23	44689922	44693724	dre-circ-2014	0	-	0	0	0	0	66
chr23	44736928	44737613	dre-circ-2015	0	+	0	0	0	0	2
chr23	44738764	44742631	dre-circ-2016	0	+	9	2	57	101	15
chr23	45088781	45088957	dre-circ-2017	0	+	0	0	5	0	0
chr23	45436407	45442786	dre-circ-3615	0	+	2	0	0	0	0
chr23	46100405	46101455	dre-circ-3616	0	-	7	0	0	0	2

chr23	46100405	46107703	dre-circ-3617	0	-	4	0	2	0	2
chr23	46116458	46116657	dre-circ-3618	0	-	3	0	0	0	0
chr2	34645149	34647071	dre-circ-165	0	+	0	2	0	0	0
chr2	3512879	3513082	dre-circ-3619	0	-	2	0	0	0	0
chr23	5303490	5306269	dre-circ-1944	0	-	0	0	0	2	0
chr23	549189	552121	dre-circ-3620	0	-	6	0	0	0	2
chr23	549189	552124	dre-circ-3621	0	-	4	0	0	0	0
chr23	5557595	5571771	dre-circ-3622	0	+	0	0	2	0	0
chr23	5658798	5661713	dre-circ-3623	0	-	0	0	4	0	0
chr23	5678326	5685308	dre-circ-1945	0	+	4	0	5	0	0
chr23	6188356	6188619	dre-circ-3624	0	+	4	0	0	0	0
chr23	635697	635800	dre-circ-1939	0	+	0	0	6	0	0
chr23	6396590	6402082	dre-circ-3625	0	-	0	0	3	0	3
chr23	6651678	6664169	dre-circ-3626	0	+	2	0	0	0	0
chr23	6747934	6806592	dre-circ-1946	0	-	2	2	0	0	2
chr23	6757284	6816462	dre-circ-1947	0	-	0	0	0	0	20
chr23	6757972	6818901	dre-circ-1948	0	-	0	0	6	0	0
chr23	6777724	6833592	dre-circ-1949	0	-	0	0	0	0	3
chr23	6794211	6855911	dre-circ-1950	0	-	13	0	0	0	34
chr23	6963215	6972914	dre-circ-3627	0	-	0	0	2	0	0
chr2	37026846	37029505	dre-circ-166	0	-	0	0	0	0	2
chr23	7069336	7069794	dre-circ-3628	0	-	2	0	0	0	0
chr2	37095842	37096175	dre-circ-3629	0	-	0	0	4	0	0
chr2	37178925	37179206	dre-circ-3630	0	-	13	0	6	2	10
chr2	37181123	37181377	dre-circ-167	0	-	4	0	0	0	0
chr2	37181207	37181550	dre-circ-168	0	+	0	0	5	0	0
chr2	37284813	37285281	dre-circ-169	0	-	0	0	2	0	0
chr2	37408784	37420369	dre-circ-170	0	-	0	0	0	0	2
chr2	37408796	37420369	dre-circ-3631	0	-	0	0	0	0	2
chr2	37565171	37575053	dre-circ-171	0	+	2	0	0	0	0
chr2	37565204	37575086	dre-circ-172	0	-	0	69	158	0	0
chr2	37565214	37575096	dre-circ-173	0	+	0	68	162	0	0
chr2	37627455	37635057	dre-circ-174	0	-	0	0	0	2	0
chr2	37943701	37945195	dre-circ-3632	0	-	0	0	7	7	0
chr2	38772477	38790770	dre-circ-175	0	-	0	0	0	0	2
chr23	8809034	8813569	dre-circ-1951	0	+	0	4	0	0	0
chr23	8831006	8836984	dre-circ-1952	0	-	0	0	0	0	4
chr2	3934	4292	dre-circ-122	0	+	2	0	0	0	0
chr23	954309	958348	dre-circ-3633	0	-	2	0	0	0	0
chr23	9575770	9593555	dre-circ-3634	0	-	2	0	0	0	0
chr23	961153	972375	dre-circ-3635	0	-	0	0	7	0	5
chr23	9813080	9816264	dre-circ-3636	0	-	0	0	0	3	0
chr2	39900452	39981764	dre-circ-176	0	-	0	0	3	0	0
chr24	11187604	11195825	dre-circ-2041	0	-	0	0	0	0	2
chr24	12867445	12871421	dre-circ-2042	0	-	2	0	0	0	0
chr24	12927045	12933062	dre-circ-2043	0	+	0	3	2	0	0
chr24	14958113	14973864	dre-circ-2044	0	+	0	0	0	0	2
chr24	15160303	15160782	dre-circ-2045	0	-	2	0	0	0	0
chr24	16229888	16244816	dre-circ-2046	0	-	0	0	0	0	2
chr24	16339053	16363996	dre-circ-2047	0	-	0	0	0	0	2
chr24	16637435	16676810	dre-circ-2048	0	-	0	0	0	0	2
chr24	16642390	16680601	dre-circ-2049	0	-	0	0	0	0	11
chr24	17560879	17565366	dre-circ-3637	0	+	8	0	7	0	0
chr24	17571049	17576633	dre-circ-2050	0	-	2	0	0	0	8
chr24	17571377	17576848	dre-circ-2051	0	-	5	6	14	7	22
chr24	19627627	19649714	dre-circ-2052	0	+	0	0	0	2	0
chr24	20621695	20652016	dre-circ-2053	0	-	0	0	0	10	0
chr24	20624835	20655211	dre-circ-2054	0	-	0	0	0	14	0
chr24	21144672	21144804	dre-circ-2055	0	+	2	0	0	0	0
chr24	21521539	21529936	dre-circ-3638	0	+	0	0	0	0	9
chr24	21521720	21526700	dre-circ-2056	0	-	0	0	4	0	10
chr24	21525455	21529203	dre-circ-2057	0	+	0	0	19	7	0
chr24	21946469	21946600	dre-circ-2058	0	-	0	0	0	2	0
chr2	42199390	42200013	dre-circ-177	0	-	0	0	0	0	2
chr24	22019390	22019926	dre-circ-3639	0	-	7	0	0	0	4
chr24	22043522	22046198	dre-circ-3640	0	-	5	0	0	2	0
chr24	22043522	22053508	dre-circ-3641	0	-	4	0	0	0	3
chr24	22043522	22058441	dre-circ-3642	0	-	0	0	3	0	0
chr2	42218842	42226283	dre-circ-3643	0	-	0	0	4	0	0
chr24	22535407	22539887	dre-circ-2059	0	-	0	0	2	0	0
chr24	24763516	24767360	dre-circ-2060	0	-	2	0	0	0	0
chr24	2498598	2499173	dre-circ-3644	0	-	2	0	0	2	3
chr24	2514299	2538114	dre-circ-2019	0	+	0	0	2	0	0
chr2	42528931	42533425	dre-circ-3645	0	-	0	4	5	0	0
chr2	42555860	42560763	dre-circ-3646	0	-	0	0	4	0	4
chr24	26247069	26250004	dre-circ-2061	0	+	0	0	0	2	2
chr24	26247069	26250156	dre-circ-2062	0	+	0	0	0	0	2
chr24	26688776	26692374	dre-circ-3647	0	+	3	0	2	3	2
chr24	26688776	26694808	dre-circ-3648	0	+	2	5	0	0	0
chr24	26731135	26731613	dre-circ-2063	0	+	0	0	2	0	0
chr2	42987343	42989365	dre-circ-178	0	+	2	0	0	0	0
chr24	30510492	30521976	dre-circ-2064	0	-	0	3	6	0	15
chr24	30596701	30597004	dre-circ-3649	0	+	0	0	0	7	0
chr24	30798434	30798885	dre-circ-2065	0	+	2	0	0	0	0
chr24	30820087	30823974	dre-circ-3650	0	-	0	4	0	2	2
chr24	30958382	30960638	dre-circ-3651	0	+	0	0	3	0	0
chr2	43260714	43265158	dre-circ-179	0	-	2	0	0	0	0

chr2	43287990	43292583	dre-circ-3652	0	-	15	0	2	0	0
chr2	43336528	43361782	dre-circ-180	0	+	0	0	0	0	2
chr24	34459948	34462418	dre-circ-3653	0	-	4	0	0	0	0
chr24	34468020	34469908	dre-circ-3654	0	-	2	0	0	0	0
chr24	34506946	34509098	dre-circ-2066	0	-	2	0	0	0	0
chr24	3452388	3456065	dre-circ-3655	0	-	0	0	2	0	2
chr24	34699260	34707639	dre-circ-2067	0	+	2	0	0	0	0
chr24	34738866	34837990	dre-circ-2068	0	-	0	0	0	0	2
chr24	34765841	34840071	dre-circ-2069	0	-	0	0	0	2	0
chr24	35366460	35375334	dre-circ-2070	0	+	0	0	0	0	2
chr24	35575214	35605767	dre-circ-2071	0	-	0	0	0	0	2
chr2	43589256	43594680	dre-circ-181	0	+	0	0	0	2	0
chr2	43635255	43635545	dre-circ-3656	0	+	6	0	0	0	0
chr24	36488141	36491118	dre-circ-3657	0	+	2	0	0	0	0
chr2	43673348	43690838	dre-circ-182	0	+	0	0	2	0	0
chr2	43680997	43700263	dre-circ-183	0	+	0	0	0	2	0
chr24	36887351	36887521	dre-circ-2072	0	+	0	0	0	0	2
chr24	37467482	37467748	dre-circ-2073	0	+	0	0	20	0	0
chr24	37471815	37473770	dre-circ-3658	0	+	0	0	13	7	0
chr24	37763427	37763760	dre-circ-3659	0	-	37	3	11	8	4
chr24	37777102	37777503	dre-circ-3660	0	-	13	0	5	2	4
chr24	37786450	37795988	dre-circ-3661	0	-	5	2	3	0	0
chr24	37870065	37876838	dre-circ-2074	0	+	0	0	3	3	0
chr24	37876688	37880605	dre-circ-3662	0	+	0	0	5	0	0
chr24	37882860	37886654	dre-circ-2075	0	+	0	0	2	0	0
chr24	38074615	38076739	dre-circ-2076	0	+	0	0	0	0	2
chr24	38430814	38438086	dre-circ-2077	0	-	0	0	0	0	11
chr24	389774	390564	dre-circ-2018	0	+	0	0	2	0	0
chr24	3948192	4036213	dre-circ-2020	0	-	0	0	0	0	2
chr24	39585794	39602160	dre-circ-2078	0	-	0	0	0	0	14
chr24	39586086	39602452	dre-circ-2079	0	+	0	0	0	0	5
chr2	43966086	43967197	dre-circ-3663	0	+	0	0	0	0	2
chr24	39680494	39682260	dre-circ-3664	0	+	0	6	0	10	0
chr24	39700018	39714537	dre-circ-2080	0	-	0	0	0	0	3
chr24	39703231	39711987	dre-circ-2081	0	-	0	0	0	0	2
chr2	43976623	44009891	dre-circ-3665	0	+	3	0	8	2	6
chr24	40040105	40056450	dre-circ-2082	0	-	0	0	9	2	0
chr24	40148669	40149114	dre-circ-2083	0	-	2	0	0	0	0
chr24	40187252	40208043	dre-circ-2084	0	+	0	0	0	0	2
chr24	40623445	40624251	dre-circ-2085	0	+	3	0	0	0	0
chr24	41148746	41149523	dre-circ-2086	0	+	2	0	0	0	0
chr24	41190439	41190942	dre-circ-2087	0	+	2	0	0	0	0
chr24	42243960	42268725	dre-circ-2088	0	-	0	0	0	15	0
chr24	42244865	42269639	dre-circ-2089	0	-	0	0	0	4	0
chr24	42244865	42301774	dre-circ-2090	0	-	0	0	0	4	0
chr24	42244865	42319092	dre-circ-2091	0	-	0	0	0	7	0
chr24	42245913	42270634	dre-circ-2092	0	-	0	0	0	76	0
chr24	42246913	42271784	dre-circ-2093	0	-	0	0	0	4	0
chr24	42246913	42303989	dre-circ-2094	0	-	0	0	0	2	0
chr24	42246913	42321240	dre-circ-3666	0	-	0	0	0	7	0
chr24	42247482	42273144	dre-circ-2095	0	-	0	0	0	70	0
chr24	42247976	42273654	dre-circ-2096	0	-	0	0	0	14	0
chr24	42267640	42299805	dre-circ-3667	0	-	2	0	0	48	0
chr24	42267640	42315922	dre-circ-2097	0	-	0	0	0	41	4
chr24	42269718	42301774	dre-circ-2098	0	-	0	0	0	15	0
chr24	42269718	42319092	dre-circ-2099	0	-	0	0	0	17	0
chr24	42269718	42350094	dre-circ-3668	0	-	0	0	0	19	0
chr24	42271259	42320671	dre-circ-2100	0	-	2	0	0	20	0
chr24	42272680	42303989	dre-circ-2101	0	-	0	0	0	23	0
chr24	42272680	42321240	dre-circ-2102	0	-	0	0	0	15	0
chr24	42272680	42352252	dre-circ-2103	0	-	0	0	0	22	0
chr24	42278700	42306549	dre-circ-2104	0	-	0	0	0	38	3
chr24	42278700	42323671	dre-circ-2105	0	-	0	0	0	19	0
chr24	42278700	42353774	dre-circ-2106	0	-	0	0	0	23	0
chr24	42280847	42307507	dre-circ-2107	0	-	0	0	0	6	0
chr24	42280847	42324379	dre-circ-2108	0	-	0	0	0	8	0
chr24	42280847	42354914	dre-circ-2109	0	-	0	0	0	5	0
chr24	42330073	42356411	dre-circ-2110	0	-	0	0	0	0	2
chr24	43506922	43536445	dre-circ-2111	0	+	2	2	0	0	0
chr24	43528323	43542737	dre-circ-3669	0	+	0	0	0	0	2
chr24	43563705	43566072	dre-circ-2112	0	-	0	0	2	0	0
chr24	43834346	43834856	dre-circ-3670	0	-	3	0	0	0	0
chr24	43837299	43838080	dre-circ-2113	0	+	0	0	2	0	0
chr24	43855661	43855897	dre-circ-2114	0	-	0	0	4	0	0
chr24	43889543	43892796	dre-circ-2115	0	+	2	0	0	0	0
chr24	43906844	43908068	dre-circ-2116	0	+	2	0	0	0	0
chr2	44664536	44665722	dre-circ-3671	0	+	5	0	3	0	0
chr2	44673398	44673601	dre-circ-3672	0	+	7	0	0	0	2
chr2	44689495	44690814	dre-circ-184	0	+	2	0	0	0	0
chr2	45030656	45031191	dre-circ-3673	0	-	2	0	0	0	0
chr2	45465493	45467813	dre-circ-185	0	+	0	0	0	2	0
chr2	45548893	45549330	dre-circ-186	0	-	2	0	0	0	0
chr2	4559345	4561050	dre-circ-3674	0	-	4	0	0	0	0
chr24	5889342	5918069	dre-circ-2021	0	+	0	0	0	0	16
chr24	6323756	6341824	dre-circ-2022	0	-	0	0	0	0	2
chr2	4753742	4754529	dre-circ-134	0	-	2	0	0	0	0
chr24	7562323	7565380	dre-circ-2023	0	+	2	0	0	0	0

chr24	7604588	7605013	dre-circ-2024	0	+	2	0	0	0	0
chr2	47877104	47877646	dre-circ-3675	0	+	5	0	0	0	0
chr2	47938439	47943301	dre-circ-3676	0	+	4	0	0	0	0
chr2	47940003	47943301	dre-circ-187	0	+	2	0	0	0	0
chr2	47959383	47970399	dre-circ-188	0	+	0	0	3	0	0
chr2	47983997	48016346	dre-circ-189	0	-	0	0	0	0	3
chr24	8014295	8023870	dre-circ-2025	0	+	2	0	0	0	0
chr2	48097226	48111816	dre-circ-190	0	+	0	0	0	0	2
chr2	48097663	48112253	dre-circ-191	0	-	3	0	0	0	68
chr2	4818654	4819557	dre-circ-3677	0	-	0	0	5	2	0
chr2	48213462	48213760	dre-circ-192	0	+	2	0	0	0	0
chr2	48731	52896	dre-circ-123	0	-	0	0	0	0	3
chr2	49346854	49358313	dre-circ-3678	0	-	0	0	0	3	0
chr2	49458582	49460667	dre-circ-193	0	+	0	2	0	0	0
chr24	9476841	9477118	dre-circ-2026	0	-	0	0	0	0	2
chr24	9778370	9820391	dre-circ-2027	0	-	0	0	0	0	12
chr24	9783255	9788878	dre-circ-2028	0	-	0	0	0	0	25
chr24	9784331	9789954	dre-circ-2029	0	-	0	0	0	0	25
chr24	9789983	9820805	dre-circ-2030	0	-	0	0	0	0	23
chr24	9796125	9812716	dre-circ-2031	0	+	0	0	0	0	57
chr24	9798520	9806772	dre-circ-2032	0	-	0	0	0	0	51
chr24	9798520	9815019	dre-circ-2033	0	-	0	0	0	0	20
chr24	9804443	9819704	dre-circ-2034	0	-	0	0	0	0	40
chr24	9805549	9813802	dre-circ-2035	0	-	0	0	0	0	56
chr24	9806204	9814457	dre-circ-2036	0	-	0	0	0	0	2
chr24	9806849	9815019	dre-circ-2037	0	-	0	0	0	0	116
chr24	9806849	9821885	dre-circ-2038	0	-	0	0	0	0	9
chr24	9813396	9820391	dre-circ-2039	0	-	0	0	0	0	164
chr24	9814070	9820945	dre-circ-2040	0	-	0	0	0	0	21
chr2	50008175	50014279	dre-circ-194	0	+	0	0	0	2	0
chr2	5010315	5013996	dre-circ-3679	0	+	2	0	3	0	0
chr2	5010315	5014640	dre-circ-135	0	+	2	0	0	0	0
chr2	5103192	5113840	dre-circ-136	0	-	0	0	0	4	0
chr25	11542232	11564187	dre-circ-2145	0	+	2	0	0	0	0
chr25	11613393	11618556	dre-circ-3680	0	+	0	0	2	0	0
chr25	11845309	11859208	dre-circ-2146	0	+	2	0	2	0	0
chr25	12597186	12600995	dre-circ-3681	0	+	0	0	4	0	0
chr25	12749231	12762644	dre-circ-2147	0	+	0	0	0	0	18
chr25	12751475	12763383	dre-circ-2148	0	+	0	0	0	0	19
chr25	12752049	12767189	dre-circ-2149	0	+	0	0	4	0	0
chr25	13358935	13361144	dre-circ-3682	0	+	0	0	2	0	0
chr25	14865157	14890001	dre-circ-3683	0	-	0	0	3	0	0
chr2	51638704	51649033	dre-circ-195	0	+	0	0	3	21	15
chr2	51667212	51670594	dre-circ-3684	0	+	2	0	0	16	0
chr25	16974527	16974896	dre-circ-3685	0	-	4	0	0	0	0
chr25	1698452	1699432	dre-circ-3686	0	-	2	0	0	0	0
chr25	1725766	1725886	dre-circ-2118	0	+	2	0	0	0	0
chr2	51760776	51769133	dre-circ-3687	0	-	2	0	0	0	0
chr25	17684518	17685240	dre-circ-3688	0	+	2	2	0	0	0
chr25	17731519	17745517	dre-circ-2150	0	-	0	0	0	0	4
chr25	17731978	17754327	dre-circ-2151	0	-	0	0	0	0	2
chr25	17735052	17748382	dre-circ-2152	0	-	0	0	0	0	5
chr25	18682066	18683013	dre-circ-2153	0	-	4	0	0	0	0
chr25	18759720	18763713	dre-circ-2154	0	+	2	0	0	0	0
chr25	1881	28256	dre-circ-3689	0	+	2	0	2	2	3
chr25	18939167	18950931	dre-circ-2155	0	-	0	0	3	2	0
chr25	18939597	18952701	dre-circ-2156	0	-	0	0	14	8	3
chr25	20391520	20391806	dre-circ-3690	0	+	4	0	0	0	0
chr25	20578997	20586330	dre-circ-3691	0	+	0	0	0	5	0
chr25	21978947	21984104	dre-circ-3692	0	-	0	2	0	0	2
chr25	22850335	22856739	dre-circ-3693	0	+	0	0	0	3	0
chr25	22886407	22887039	dre-circ-2157	0	-	0	0	0	0	2
chr25	24018704	24068559	dre-circ-2158	0	+	0	0	0	0	2
chr25	24284888	24285855	dre-circ-3694	0	-	6	2	0	0	0
chr25	24349065	24351358	dre-circ-3695	0	+	2	0	5	0	0
chr2	52481253	52490381	dre-circ-3696	0	+	0	0	0	0	3
chr25	26726829	26739425	dre-circ-2159	0	+	0	0	2	0	0
chr25	26738783	26739425	dre-circ-3697	0	+	0	3	0	2	0
chr2	52722770	52722941	dre-circ-196	0	-	2	0	0	0	0
chr25	27607207	27610180	dre-circ-3698	0	-	3	0	0	0	0
chr25	28290896	28291494	dre-circ-2160	0	+	2	0	0	0	0
chr25	28986042	28988787	dre-circ-2161	0	+	0	0	0	0	2
chr25	29151716	29154430	dre-circ-3699	0	+	0	0	4	7	3
chr25	29746028	29750831	dre-circ-3700	0	+	11	0	0	2	0
chr2	53038945	53083998	dre-circ-197	0	-	2	0	0	0	0
chr25	31211021	31216509	dre-circ-2162	0	+	2	0	0	0	0
chr25	3122637	3125095	dre-circ-2119	0	-	0	0	0	2	0
chr25	31435816	31439440	dre-circ-2163	0	+	0	0	0	0	2
chr25	31456721	31460189	dre-circ-3701	0	+	9	0	0	0	0
chr25	3183672	3267013	dre-circ-2120	0	-	2	0	0	0	5
chr25	3186319	3269702	dre-circ-2121	0	+	0	0	0	0	3
chr25	3203772	3290554	dre-circ-2122	0	+	46	0	57	23	38
chr25	3204831	3293001	dre-circ-2123	0	+	0	0	0	0	27
chr25	32079506	32117117	dre-circ-2164	0	+	2	0	7	0	5
chr25	32088759	32127832	dre-circ-2165	0	+	0	0	0	58	5
chr25	32118765	32127832	dre-circ-2166	0	+	0	0	0	23	0
chr25	32197516	32200842	dre-circ-3702	0	+	0	0	3	0	0

chr25	32549890	32556405	dre-circ-2167	0	-	0	0	0	2	0
chr25	33750736	33754258	dre-circ-3703	0	+	0	0	0	0	3
chr25	33837613	33837736	dre-circ-2168	0	-	0	2	0	0	0
chr2	5352083	5357962	dre-circ-3704	0	-	0	0	0	2	0
chr2	5354039	5357962	dre-circ-3705	0	-	3	0	0	0	0
chr25	3563619	3566306	dre-circ-3706	0	-	3	0	0	0	0
chr25	36106846	36174944	dre-circ-2169	0	-	0	0	0	0	16
chr25	36163441	36181776	dre-circ-2170	0	+	0	0	0	0	37
chr25	36168469	36208903	dre-circ-2171	0	+	0	0	0	0	2
chr25	36281400	36285667	dre-circ-2172	0	+	0	0	2	0	0
chr25	36708670	36710042	dre-circ-3707	0	-	0	0	0	0	3
chr25	36763508	36763974	dre-circ-3708	0	+	0	0	0	0	3
chr25	3691941	3692326	dre-circ-2124	0	+	2	0	0	0	0
chr25	36993987	36994623	dre-circ-2173	0	+	3	0	0	0	0
chr25	37162243	37165261	dre-circ-2174	0	+	2	0	0	0	0
chr25	37277910	37279790	dre-circ-2175	0	-	2	0	0	0	0
chr25	37364112	37376882	dre-circ-2176	0	+	0	0	0	0	2
chr25	37375104	37382681	dre-circ-2177	0	+	0	0	0	0	3
chr25	38029964	38030160	dre-circ-2178	0	-	0	2	0	0	0
chr2	53830581	53838763	dre-circ-3709	0	-	0	0	2	0	0
chr2	53852142	53852361	dre-circ-198	0	-	0	0	2	0	0
chr25	4035486	4036189	dre-circ-3710	0	-	0	0	0	0	3
chr2	54228300	54228574	dre-circ-3711	0	+	4	0	4	0	0
chr2	54243838	54244171	dre-circ-3712	0	+	10	0	2	3	5
chr25	4443792	4443894	dre-circ-2125	0	+	0	2	0	0	0
chr2	54549739	54550117	dre-circ-199	0	-	0	0	0	0	2
chr2	54846358	54848793	dre-circ-200	0	+	0	0	0	0	2
chr2	54861718	54878541	dre-circ-3713	0	+	0	0	2	0	0
chr25	4926692	4968510	dre-circ-2126	0	+	0	0	2	0	0
chr2	54992668	55002914	dre-circ-201	0	+	2	0	0	0	0
chr2	5514989	5516115	dre-circ-137	0	+	0	0	2	0	0
chr25	5289507	5315199	dre-circ-2127	0	+	0	0	0	0	3
chr25	5290686	5316341	dre-circ-2128	0	+	0	0	3	0	0
chr25	5290686	5335361	dre-circ-2129	0	+	0	0	2	0	3
chr2	55398718	55479330	dre-circ-3714	0	-	7	2	5	5	12
chr2	5558342	5561773	dre-circ-3715	0	+	0	2	0	0	2
chr25	6006308	6008481	dre-circ-3716	0	+	3	0	0	0	0
chr25	6367121	6375033	dre-circ-2130	0	+	0	0	0	0	2
chr25	6640673	6641542	dre-circ-2131	0	-	0	0	0	0	2
chr25	704873	705733	dre-circ-3717	0	+	10	0	3	0	0
chr2	57091142	57091352	dre-circ-3718	0	+	0	3	0	0	0
chr25	7392682	7392974	dre-circ-2132	0	+	0	0	2	0	0
chr2	57410233	57410727	dre-circ-3719	0	-	3	0	0	0	0
chr25	7862245	7873358	dre-circ-2133	0	+	0	0	0	0	4
chr25	8092423	8102796	dre-circ-2134	0	+	0	0	2	0	0
chr2	58106226	58113632	dre-circ-3720	0	+	12	0	4	0	20
chr25	8118850	8118954	dre-circ-2135	0	+	2	0	0	0	3
chr2	58571060	58571281	dre-circ-202	0	-	0	0	0	0	2
chr25	8573258	8624060	dre-circ-3721	0	+	10	0	0	0	83
chr25	8573684	8624344	dre-circ-2136	0	+	2	0	0	0	0
chr25	8573882	8624548	dre-circ-2137	0	+	0	0	0	0	80
chr25	8574885	8624766	dre-circ-3722	0	+	13	0	0	0	107
chr25	8575522	8625468	dre-circ-2138	0	+	3	0	0	0	50
chr25	8577606	8626442	dre-circ-2139	0	-	0	0	0	0	4
chr25	8591968	8592135	dre-circ-2140	0	-	0	0	0	0	22
chr25	8599782	8645800	dre-circ-2141	0	+	0	0	0	0	12
chr2	58715855	58716370	dre-circ-203	0	-	2	0	0	0	0
chr2	58717557	58718075	dre-circ-204	0	-	0	0	0	0	3
chr2	58717557	58735634	dre-circ-205	0	-	0	0	0	0	6
chr25	8969388	8969977	dre-circ-2142	0	+	0	0	2	0	0
chr2	59007410	59018755	dre-circ-3723	0	-	0	0	5	4	0
chr25	9033890	9067809	dre-circ-2143	0	-	0	0	2	0	0
chr25	9060998	9067809	dre-circ-2144	0	-	0	0	0	0	2
chr25	948312	1036714	dre-circ-3724	0	+	0	4	0	0	0
chr2	59619431	59620068	dre-circ-3725	0	+	5	2	4	4	0
chr2	59619431	59620094	dre-circ-3726	0	+	4	0	0	0	0
chr25	963139	963262	dre-circ-2117	0	-	3	0	0	0	2
chr2	6010895	6011238	dre-circ-138	0	-	0	0	2	0	0
chr2	60170332	60195993	dre-circ-206	0	-	0	0	0	0	2
chr2	60170332	60226438	dre-circ-207	0	-	0	0	0	0	2
chr2	6786598	6789319	dre-circ-139	0	+	2	0	0	0	0
chr2	7470755	7473987	dre-circ-3727	0	-	0	0	4	0	0
chr2	7534083	7535330	dre-circ-140	0	-	0	0	0	3	0
chr2	7708664	7708767	dre-circ-141	0	-	0	2	0	0	0
chr2	7865232	7874295	dre-circ-142	0	-	0	0	0	2	0
chr2	795026	795521	dre-circ-129	0	+	2	0	0	0	0
chr2	9324789	9328823	dre-circ-3728	0	+	0	3	0	0	0
chr2	9550947	9551248	dre-circ-143	0	+	2	0	0	0	0
chr2	9817145	9817587	dre-circ-3729	0	+	3	0	0	2	11
chr3	1075522	1089798	dre-circ-210	0	-	0	0	4	0	0
chr3	11505502	11516635	dre-circ-3730	0	-	3	0	0	0	0
chr3	11801309	11801894	dre-circ-267	0	-	0	0	0	0	3
chr3	12507735	12546769	dre-circ-268	0	-	0	0	0	0	2
chr3	13433829	13434071	dre-circ-3731	0	-	8	0	0	0	2
chr3	13468739	13469599	dre-circ-269	0	-	2	0	0	0	0
chr3	13953399	13992019	dre-circ-270	0	+	0	0	3	0	0
chr3	13962364	13992162	dre-circ-271	0	+	0	0	2	0	0

chr3	13979142	14044459	dre-circ-272	0	+	0	0	0	0	3
chr3	14042343	14065158	dre-circ-273	0	+	0	0	0	0	2
chr3	14043373	14065711	dre-circ-3732	0	+	0	0	0	0	3
chr3	14158115	14163305	dre-circ-274	0	+	41	0	0	0	0
chr3	14158208	14165183	dre-circ-275	0	+	2	0	0	0	0
chr3	14679113	14679441	dre-circ-276	0	-	0	0	0	0	2
chr3	14941723	14955802	dre-circ-277	0	-	0	0	0	2	0
chr3	15327149	15327636	dre-circ-3733	0	-	0	0	0	2	0
chr3	15427038	15427366	dre-circ-278	0	-	3	0	0	0	0
chr3	15550030	15556678	dre-circ-3734	0	+	6	0	2	0	0
chr3	15550030	15562572	dre-circ-3735	0	+	0	0	0	2	0
chr3	15700641	15706916	dre-circ-279	0	-	2	0	0	0	0
chr3	15757023	15759166	dre-circ-280	0	-	0	0	0	0	2
chr3	15811119	15811776	dre-circ-281	0	-	0	0	0	2	0
chr3	16265418	16270164	dre-circ-282	0	+	0	2	0	0	0
chr3	16500462	16505879	dre-circ-283	0	+	0	0	0	0	2
chr3	16503081	16503449	dre-circ-3736	0	+	2	0	0	0	3
chr3	16576082	16576963	dre-circ-3737	0	+	2	0	0	0	0
chr3	16929020	16933501	dre-circ-3738	0	+	0	0	3	0	0
chr3	16936126	16936871	dre-circ-3739	0	+	11	0	0	6	0
chr3	17337153	17339667	dre-circ-3740	0	+	4	0	4	0	0
chr3	1781332	1805186	dre-circ-211	0	+	0	0	0	2	0
chr3	18342886	18437486	dre-circ-284	0	+	4	0	0	0	12
chr3	18993897	18994239	dre-circ-3741	0	-	2	0	0	0	0
chr3	21202194	21202553	dre-circ-3742	0	-	3	0	0	0	0
chr3	21371465	21377797	dre-circ-285	0	-	2	0	0	0	0
chr3	22292280	22293519	dre-circ-3743	0	+	2	0	0	0	0
chr3	22700560	22703370	dre-circ-3744	0	+	6	0	0	0	0
chr3	22743447	22821134	dre-circ-286	0	+	17	0	0	0	0
chr3	22751778	22832059	dre-circ-287	0	+	21	0	0	0	0
chr3	23344795	23365293	dre-circ-288	0	+	0	0	0	0	6
chr3	23485672	23504198	dre-circ-3745	0	+	0	0	0	0	2
chr3	23625594	23626332	dre-circ-289	0	-	0	2	0	0	0
chr3	23625625	23626332	dre-circ-3746	0	-	0	0	0	3	0
chr3	23890544	23890854	dre-circ-290	0	-	2	0	0	0	0
chr3	24148581	24152178	dre-circ-3747	0	-	0	2	0	0	4
chr3	24611817	24612445	dre-circ-291	0	-	0	0	0	0	2
chr3	24900077	24900456	dre-circ-292	0	+	0	0	0	0	2
chr3	25226158	25228010	dre-circ-293	0	+	2	0	0	0	0
chr3	26120386	26123254	dre-circ-3748	0	+	0	0	3	0	0
chr3	26466096	26466957	dre-circ-294	0	-	0	2	0	0	0
chr3	26802714	26804812	dre-circ-295	0	-	0	2	0	0	0
chr3	26913309	26919654	dre-circ-296	0	+	0	0	0	0	6
chr3	26977229	26978266	dre-circ-297	0	-	0	0	2	0	0
chr3	27824443	27824844	dre-circ-298	0	+	0	0	0	0	2
chr3	29492619	29493072	dre-circ-3749	0	+	0	2	0	0	0
chr3	30074339	30076909	dre-circ-299	0	-	0	0	0	0	39
chr3	30076546	30079618	dre-circ-300	0	-	0	0	3	0	5
chr3	30079509	30079864	dre-circ-301	0	-	0	0	2	0	5
chr3	30456064	30456446	dre-circ-3750	0	+	0	0	0	2	0
chr3	31895873	31904120	dre-circ-302	0	-	0	0	2	0	0
chr3	31895873	31905931	dre-circ-303	0	-	0	0	2	0	0
chr3	31895873	31913184	dre-circ-3751	0	-	0	0	7	0	0
chr3	32063926	32064474	dre-circ-304	0	+	0	0	0	0	2
chr3	32237117	32262532	dre-circ-305	0	+	13	0	7	3	24
chr3	32237394	32242839	dre-circ-306	0	+	3	0	3	0	26
chr3	32237394	32249811	dre-circ-307	0	+	8	0	5	0	21
chr3	32237394	32256630	dre-circ-308	0	+	8	0	4	0	26
chr3	32237394	32262864	dre-circ-309	0	+	6	0	3	0	16
chr3	32237394	32267404	dre-circ-310	0	+	4	0	6	5	24
chr3	32237394	32272760	dre-circ-311	0	+	5	0	0	3	31
chr3	32237394	32277395	dre-circ-312	0	+	7	2	3	2	19
chr3	32237726	32243380	dre-circ-313	0	-	9	0	12	6	108
chr3	32237726	32250284	dre-circ-314	0	-	9	0	13	7	100
chr3	32237726	32257086	dre-circ-315	0	-	10	0	14	0	87
chr3	32237726	32267875	dre-circ-316	0	-	7	0	10	3	43
chr3	32237726	32273174	dre-circ-317	0	-	9	0	9	0	48
chr3	32237726	32277867	dre-circ-318	0	-	6	0	4	2	28
chr3	32237844	32257204	dre-circ-319	0	+	5	0	7	0	55
chr3	32330048	32333591	dre-circ-3752	0	-	0	0	4	0	0
chr3	32398576	32407042	dre-circ-320	0	-	0	0	0	0	2
chr3	33114549	33143287	dre-circ-321	0	-	3	0	0	0	0
chr3	34791969	34799810	dre-circ-322	0	-	3	0	0	0	0
chr3	34796290	34799810	dre-circ-3753	0	-	2	0	0	0	0
chr3	34810102	34828938	dre-circ-3754	0	-	3	0	0	0	0
chr3	35902315	35923273	dre-circ-323	0	+	0	0	0	0	2
chr3	36121970	36122696	dre-circ-324	0	-	2	0	0	0	0
chr3	36718414	36721334	dre-circ-325	0	+	0	0	0	0	3
chr3	36878376	36882596	dre-circ-326	0	+	2	0	0	0	0
chr3	38668312	38668904	dre-circ-3755	0	-	0	38	0	2	0
chr3	38949754	38958410	dre-circ-327	0	+	0	0	2	0	0
chr3	39133694	39142852	dre-circ-3756	0	-	0	0	0	2	0
chr3	39144281	39147444	dre-circ-328	0	-	0	0	0	2	0
chr3	39146968	39147444	dre-circ-3757	0	-	0	0	3	3	0
chr3	39178862	39187314	dre-circ-329	0	-	0	0	4	0	0
chr3	39184848	39192496	dre-circ-330	0	-	0	0	20	10	5
chr3	3921649	3935692	dre-circ-212	0	-	0	0	0	0	51

chr3	3921649	3949386	dre-circ-213	0	-	0	0	0	0	49
chr3	3921649	3966669	dre-circ-214	0	-	0	0	0	0	28
chr3	3922148	3936211	dre-circ-215	0	-	0	0	0	0	32
chr3	3922148	3949846	dre-circ-216	0	-	0	0	0	0	8
chr3	3923336	3937578	dre-circ-217	0	-	0	0	0	0	7
chr3	3923336	3951150	dre-circ-218	0	-	0	0	0	0	8
chr3	3923479	3937738	dre-circ-219	0	-	0	0	0	0	28
chr3	3923479	3951315	dre-circ-220	0	-	0	0	0	0	21
chr3	3923479	3969158	dre-circ-221	0	-	0	0	0	0	15
chr3	3923703	3937981	dre-circ-222	0	-	0	0	0	0	9
chr3	3923703	3969389	dre-circ-223	0	-	0	0	0	0	8
chr3	3925069	3952736	dre-circ-224	0	-	0	0	0	0	64
chr3	3925465	3939601	dre-circ-225	0	-	0	0	0	0	114
chr3	3925465	3953126	dre-circ-226	0	-	0	0	0	0	114
chr3	3925777	3939897	dre-circ-227	0	-	0	0	0	0	62
chr3	3925777	3953429	dre-circ-228	0	-	0	0	0	0	13
chr3	3927653	3942936	dre-circ-229	0	-	0	0	0	0	7
chr3	3927653	3973295	dre-circ-230	0	-	0	0	0	0	29
chr3	3928238	3943507	dre-circ-231	0	-	0	0	0	0	103
chr3	3928654	3943942	dre-circ-232	0	-	0	0	0	0	51
chr3	3928654	3974310	dre-circ-233	0	-	0	0	0	0	24
chr3	3929091	3944125	dre-circ-234	0	-	0	0	0	0	99
chr3	3929091	3955869	dre-circ-3758	0	-	0	0	0	0	62
chr3	3930568	3960755	dre-circ-235	0	-	0	0	0	0	119
chr3	3931349	3961223	dre-circ-236	0	-	0	0	0	0	224
chr3	3931507	3946792	dre-circ-237	0	+	0	0	0	0	15
chr3	3935977	3949386	dre-circ-238	0	-	0	0	0	0	21
chr3	3935977	3966669	dre-circ-239	0	-	0	0	0	0	17
chr3	3936551	3949846	dre-circ-240	0	-	0	0	0	0	45
chr3	3937158	3950510	dre-circ-241	0	-	0	0	0	0	50
chr3	3937825	3951315	dre-circ-242	0	-	0	0	0	0	39
chr3	3938388	3951706	dre-circ-243	0	-	0	0	0	0	12
chr3	3939145	3952607	dre-circ-244	0	-	0	0	0	0	102
chr3	3939672	3953126	dre-circ-245	0	-	0	0	0	0	274
chr3	3939995	3953429	dre-circ-246	0	-	0	0	0	0	6
chr3	3943587	3955232	dre-circ-247	0	-	0	0	0	0	82
chr3	3944476	3955869	dre-circ-248	0	-	0	0	0	0	79
chr3	3946175	3960755	dre-circ-249	0	-	0	0	0	0	74
chr3	3988674	4005006	dre-circ-250	0	+	0	0	0	0	5
chr3	3991127	4007414	dre-circ-251	0	+	0	0	0	0	12
chr3	3991418	4007719	dre-circ-252	0	+	0	0	0	0	11
chr3	3992118	4008229	dre-circ-253	0	+	0	0	0	0	8
chr3	3993768	4009254	dre-circ-254	0	+	0	0	0	0	5
chr3	3998565	4014524	dre-circ-255	0	+	0	0	0	0	11
chr3	3998954	4014929	dre-circ-256	0	+	0	0	0	0	3
chr3	40213950	40214176	dre-circ-331	0	-	2	0	0	0	0
chr3	40610670	40613234	dre-circ-3759	0	-	6	0	0	0	0
chr3	40809097	40809518	dre-circ-332	0	+	0	0	2	0	0
chr3	40809097	40812595	dre-circ-333	0	+	2	0	0	0	0
chr3	40944124	40964940	dre-circ-334	0	-	0	0	3	4	3
chr3	41122159	41141411	dre-circ-335	0	-	0	0	0	0	2
chr3	41277434	41287055	dre-circ-3760	0	-	0	0	7	0	3
chr3	42087941	42088359	dre-circ-3761	0	+	0	0	0	0	2
chr3	42097650	42098232	dre-circ-336	0	+	0	0	2	0	0
chr3	42552364	42599390	dre-circ-3762	0	+	9	0	0	0	0
chr3	42552614	42599644	dre-circ-3763	0	+	12	0	9	4	59
chr3	42553282	42599882	dre-circ-337	0	+	7	0	0	0	41
chr3	42559919	42602876	dre-circ-3764	0	+	0	0	0	0	4
chr3	42560094	42606683	dre-circ-338	0	+	0	0	0	0	27
chr3	42561245	42607921	dre-circ-339	0	-	3	0	0	0	11
chr3	42561270	42607946	dre-circ-340	0	-	0	0	0	0	10
chr3	42562250	42608737	dre-circ-341	0	+	0	0	0	0	2
chr3	42736617	42740205	dre-circ-342	0	+	2	0	0	0	0
chr3	42737656	42742948	dre-circ-343	0	+	4	0	0	0	42
chr3	42737944	42743369	dre-circ-344	0	+	0	0	0	0	3
chr3	42738607	42743955	dre-circ-345	0	+	0	0	0	0	10
chr3	42739880	42745513	dre-circ-346	0	-	2	0	0	0	0
chr3	42739994	42745546	dre-circ-347	0	+	5	0	0	0	23
chr3	42768679	42769426	dre-circ-348	0	+	2	0	0	0	0
chr3	4410350	4425036	dre-circ-257	0	-	3	0	0	0	3
chr3	4410574	4425348	dre-circ-258	0	+	0	0	0	0	3
chr3	44289220	44291724	dre-circ-349	0	-	0	2	0	0	0
chr3	45843634	45844037	dre-circ-350	0	+	2	0	0	0	0
chr3	46333332	46342685	dre-circ-351	0	+	0	0	0	0	2
chr3	49369628	49375216	dre-circ-3765	0	+	4	0	2	0	0
chr3	50572070	50572527	dre-circ-3766	0	+	5	0	0	68	0
chr3	50572126	50572527	dre-circ-3767	0	+	0	0	0	2	0
chr3	5123672	5187921	dre-circ-259	0	-	0	0	0	0	3
chr3	5125570	5149041	dre-circ-3768	0	-	7	0	0	0	0
chr3	53215963	53216548	dre-circ-352	0	+	2	0	0	0	0
chr3	55306548	55307709	dre-circ-3769	0	-	0	0	4	0	3
chr3	55618936	55619569	dre-circ-3770	0	+	0	8	71	2	13
chr3	55635315	55658450	dre-circ-353	0	+	0	0	0	0	4
chr3	55636480	55662357	dre-circ-354	0	+	0	0	0	0	3
chr3	55643227	55662357	dre-circ-3771	0	+	0	0	3	0	6
chr3	55950204	55955191	dre-circ-355	0	-	10	0	128	0	47
chr3	55950218	55955205	dre-circ-356	0	-	8	0	5	0	0

chr3	55950796	55955683	dre-circ-357	0	-	16659	0	5680	692	230
chr3	55952076	55956952	dre-circ-358	0	+	14556	15	6647	437	376
chr3	55952212	55957235	dre-circ-359	0	-	21	0	0	0	0
chr3	5674311	5674828	dre-circ-260	0	-	2	0	0	0	0
chr3	56801357	56801709	dre-circ-360	0	+	0	2	0	0	0
chr3	58581765	58582876	dre-circ-361	0	-	0	0	0	0	4
chr3	59163069	59163567	dre-circ-362	0	+	0	0	3	0	0
chr3	59458356	59488680	dre-circ-3772	0	-	0	0	2	0	0
chr3	5988214	6003718	dre-circ-261	0	-	0	0	0	0	6
chr3	60442206	60510030	dre-circ-363	0	+	2	0	0	0	0
chr3	60482885	60523737	dre-circ-364	0	+	0	0	2	0	0
chr3	60499570	60500592	dre-circ-365	0	+	2	0	0	0	0
chr3	60499570	60523737	dre-circ-366	0	+	0	0	0	0	2
chr3	60504926	60510030	dre-circ-367	0	+	0	0	0	0	3
chr3	60504926	60523737	dre-circ-368	0	+	0	0	0	0	4
chr3	60612433	60697985	dre-circ-369	0	+	4	0	2	0	4
chr3	60965082	60975293	dre-circ-3773	0	-	29	0	7	19	36
chr3	61787739	61805693	dre-circ-370	0	-	7	0	0	137	0
chr3	61789845	61809134	dre-circ-371	0	-	0	0	0	3	0
chr3	62056567	62059761	dre-circ-372	0	-	0	0	0	0	2
chr3	62720998	62721279	dre-circ-373	0	+	0	0	0	0	2
chr3	62740522	62741958	dre-circ-3774	0	-	11	2	0	2	0
chr3	63181933	63182180	dre-circ-3775	0	-	0	3	0	0	3
chr3	722606	751760	dre-circ-208	0	-	0	0	0	0	12
chr3	7477887	7538980	dre-circ-3776	0	-	0	4	0	0	0
chr3	8056880	8063898	dre-circ-262	0	+	0	0	0	0	3
chr3	8060190	8084537	dre-circ-263	0	-	0	0	0	0	2
chr3	8204542	8212468	dre-circ-264	0	+	2	0	0	0	0
chr3	8250853	8251177	dre-circ-3777	0	+	0	0	2	0	0
chr3	8313735	8319392	dre-circ-265	0	+	0	0	0	2	0
chr3	8823381	8850265	dre-circ-266	0	-	0	0	0	10	0
chr3	988846	989287	dre-circ-209	0	-	0	0	0	2	0
chr3	9997741	9998387	dre-circ-3778	0	+	4	0	0	0	0
chr4	10397941	10400538	dre-circ-395	0	+	0	0	0	2	0
chr4	10676784	10692897	dre-circ-3779	0	+	2	0	0	0	0
chr4	11938923	11947736	dre-circ-3780	0	-	2	0	0	0	0
chr4	1202822	1209620	dre-circ-378	0	+	0	0	2	0	0
chr4	1407775	1468103	dre-circ-379	0	+	0	0	0	5	0
chr4	1421185	1472751	dre-circ-380	0	+	0	0	0	5	0
chr4	15007467	15009264	dre-circ-3781	0	+	0	0	0	0	2
chr4	16716541	16721456	dre-circ-396	0	+	2	0	0	0	0
chr4	170959	171206	dre-circ-3782	0	+	0	0	0	0	2
chr4	18067814	18076353	dre-circ-397	0	-	0	0	0	2	0
chr4	18537845	18586346	dre-circ-398	0	-	0	0	0	0	4
chr4	19063466	19063779	dre-circ-399	0	-	0	0	2	0	0
chr4	19307946	19311859	dre-circ-3783	0	+	0	2	0	0	0
chr4	19834543	19839403	dre-circ-400	0	+	0	0	0	2	0
chr4	20274583	20277712	dre-circ-3784	0	-	0	0	0	0	7
chr4	20274583	20305210	dre-circ-3785	0	-	0	0	0	2	0
chr4	20452666	20455613	dre-circ-401	0	-	0	0	0	0	2
chr4	20475324	20480457	dre-circ-3786	0	-	2	0	0	0	0
chr4	20894814	20898064	dre-circ-402	0	-	0	0	0	2	0
chr4	21242279	21242389	dre-circ-403	0	-	0	0	0	4	0
chr4	21242560	21243785	dre-circ-404	0	-	4	0	4	5	0
chr4	2430491	2434241	dre-circ-381	0	-	2	0	0	0	0
chr4	24696571	24768168	dre-circ-405	0	+	0	0	0	2	0
chr4	24697635	24744742	dre-circ-406	0	+	0	0	0	2	0
chr4	24720239	24768655	dre-circ-407	0	+	0	0	2	0	0
chr4	24734553	24777280	dre-circ-408	0	-	0	0	0	2	0
chr4	24743116	24768168	dre-circ-3787	0	+	0	0	0	11	0
chr4	24743378	24768655	dre-circ-409	0	+	0	0	0	5	0
chr4	24743509	24768944	dre-circ-410	0	+	0	0	0	3	0
chr4	24747707	24774544	dre-circ-411	0	+	0	0	0	4	0
chr4	24832710	24833713	dre-circ-3788	0	+	0	0	3	0	0
chr4	2873453	2876407	dre-circ-382	0	-	0	0	0	2	0
chr4	2929211	2929459	dre-circ-383	0	+	0	3	0	0	0
chr4	29628023	29638365	dre-circ-412	0	+	0	0	40	0	0
chr4	3700678	3701540	dre-circ-384	0	+	0	0	0	2	0
chr4	43973924	44020889	dre-circ-413	0	-	0	0	0	0	4
chr4	4790785	4792456	dre-circ-3789	0	+	0	0	3	0	0
chr4	4856392	4857741	dre-circ-385	0	-	0	0	0	2	0
chr4	4871516	4874686	dre-circ-386	0	-	0	0	0	2	0
chr4	496147	557643	dre-circ-374	0	-	0	0	0	0	2
chr4	5080316	5089543	dre-circ-387	0	+	0	0	0	0	9
chr4	5226354	5227258	dre-circ-388	0	+	0	0	0	0	2
chr4	5365929	5366036	dre-circ-389	0	+	4	0	0	0	2
chr4	5843328	5854268	dre-circ-390	0	-	0	0	2	0	0
chr4	59223506	59225347	dre-circ-3790	0	-	0	0	3	0	0
chr4	59226853	59242606	dre-circ-414	0	-	0	0	2	0	0
chr4	59352601	59397220	dre-circ-3791	0	-	33	14	44	31	60
chr4	59511018	59516239	dre-circ-3792	0	-	0	0	2	0	0
chr4	60269720	60271085	dre-circ-415	0	+	4	0	0	0	0
chr4	60270850	60271542	dre-circ-416	0	-	2	0	0	0	0
chr4	60270877	60271569	dre-circ-417	0	+	0	0	0	0	2
chr4	60484130	60503976	dre-circ-418	0	+	0	0	0	10	0
chr4	60484130	60545021	dre-circ-419	0	+	0	0	0	4	0
chr4	60484414	60505806	dre-circ-420	0	+	0	0	0	4	0

chr4	60484598	60506059	dre-circ-421	0	+	0	0	0	10	0
chr4	60581972	60588454	dre-circ-3793	0	+	0	0	0	3	0
chr4	60588344	60623599	dre-circ-422	0	+	0	0	0	3	0
chr4	60588344	60686899	dre-circ-423	0	+	0	0	0	3	0
chr4	61124296	61138388	dre-circ-424	0	-	0	0	0	0	2
chr4	61124296	61143001	dre-circ-425	0	-	0	0	0	0	2
chr4	61125013	61138739	dre-circ-426	0	-	0	0	0	0	37
chr4	61125454	61134771	dre-circ-427	0	-	0	0	0	0	3
chr4	61125454	61144153	dre-circ-428	0	-	0	0	0	0	5
chr4	61135985	61140304	dre-circ-429	0	-	0	0	0	0	28
chr4	61140581	61145088	dre-circ-430	0	-	0	0	0	0	27
chr4	61140581	61150048	dre-circ-431	0	-	0	0	0	0	20
chr4	61392591	61415983	dre-circ-432	0	+	0	0	0	2	0
chr4	61896120	61900198	dre-circ-433	0	-	0	0	2	0	0
chr4	703035	707806	dre-circ-375	0	-	0	0	0	2	0
chr4	7446747	7448625	dre-circ-391	0	+	2	0	0	0	0
chr4	813543	814010	dre-circ-376	0	+	0	0	0	0	3
chr4	815341	815526	dre-circ-377	0	+	0	0	0	0	2
chr4	8600748	8603436	dre-circ-392	0	-	2	0	0	0	0
chr4	8944190	8944398	dre-circ-393	0	-	2	0	0	0	0
chr4	9003758	9006425	dre-circ-394	0	+	3	0	0	0	0
chr4	9606345	9618122	dre-circ-3794	0	+	0	0	0	0	2
chr4	9837518	9852062	dre-circ-3795	0	-	0	0	3	0	0
chr5	1337914	1357380	dre-circ-436	0	-	0	0	0	2	0
chr5	1354893	1357876	dre-circ-3796	0	-	2	0	0	0	0
chr5	13940821	13941291	dre-circ-3797	0	-	6	3	0	0	0
chr5	14909369	14909512	dre-circ-3798	0	-	3	0	0	0	0
chr5	1600909	1601198	dre-circ-437	0	-	0	0	2	0	0
chr5	1925227	1927714	dre-circ-3799	0	-	2	0	0	0	0
chr5	2022410	2046601	dre-circ-438	0	+	83	0	0	0	0
chr5	2022754	2046940	dre-circ-439	0	+	2	0	0	0	0
chr5	2027390	2046940	dre-circ-440	0	+	103	0	13	0	0
chr5	2032548	2052722	dre-circ-441	0	+	4	0	0	0	0
chr5	21702060	21703881	dre-circ-450	0	-	2	0	0	0	0
chr5	2190764	2268544	dre-circ-442	0	-	2	0	0	0	0
chr5	2194790	2278928	dre-circ-443	0	-	7	0	0	0	0
chr5	21957188	21972479	dre-circ-451	0	-	0	0	0	2	0
chr5	21958489	21972662	dre-circ-452	0	-	0	0	0	3	0
chr5	21958753	21981261	dre-circ-453	0	-	0	0	0	2	0
chr5	2200620	2279450	dre-circ-444	0	-	4	0	4	0	0
chr5	22716409	22716688	dre-circ-454	0	+	0	4	0	0	0
chr5	22947265	22949744	dre-circ-3800	0	+	3	3	8	3	0
chr5	23052029	23066683	dre-circ-455	0	+	0	0	0	0	6
chr5	23124278	23142470	dre-circ-456	0	-	0	0	0	2	0
chr5	23302615	23318309	dre-circ-457	0	-	0	0	0	0	2
chr5	23648997	23651658	dre-circ-458	0	-	3	0	0	0	0
chr5	23823913	23828528	dre-circ-459	0	-	0	0	2	0	0
chr5	23951229	23951690	dre-circ-460	0	+	0	0	0	0	2
chr5	24054479	24066826	dre-circ-3801	0	+	2	0	5	0	0
chr5	24054479	24084624	dre-circ-3802	0	+	0	0	2	0	0
chr5	24066456	24066826	dre-circ-461	0	+	0	0	0	0	2
chr5	24084457	24096308	dre-circ-3803	0	+	0	0	3	0	0
chr5	24108021	24111139	dre-circ-3804	0	+	0	0	4	0	2
chr5	2445185	2448391	dre-circ-445	0	-	2	0	0	0	0
chr5	24770399	24772137	dre-circ-3805	0	+	2	0	0	2	0
chr5	24885317	24885931	dre-circ-3806	0	+	19	0	7	15	22
chr5	24928123	24928482	dre-circ-462	0	-	0	0	0	0	2
chr5	25374624	25391140	dre-circ-463	0	-	0	0	0	0	14
chr5	25708383	25713693	dre-circ-464	0	-	0	0	0	0	2
chr5	25850177	25850689	dre-circ-3807	0	+	0	0	5	3	0
chr5	26543218	26547907	dre-circ-3808	0	-	2	4	4	0	9
chr5	27220772	27223949	dre-circ-3809	0	-	0	0	0	2	0
chr5	27967224	27979135	dre-circ-3810	0	-	0	0	2	0	2
chr5	27967224	28014201	dre-circ-3811	0	-	0	0	13	0	0
chr5	27967224	28032384	dre-circ-465	0	-	0	0	2	0	0
chr5	27974703	28014201	dre-circ-3812	0	-	0	0	5	0	0
chr5	27979018	28014201	dre-circ-466	0	-	0	0	2	0	0
chr5	28000448	28014201	dre-circ-3813	0	-	0	0	2	0	0
chr5	2826818	2828902	dre-circ-3814	0	+	0	0	3	0	0
chr5	28443218	28443803	dre-circ-3815	0	+	4	0	0	0	0
chr5	30436811	30455012	dre-circ-467	0	+	0	0	8	46	0
chr5	30436811	30463020	dre-circ-468	0	+	0	0	7	35	0
chr5	30436811	30496892	dre-circ-469	0	+	0	0	8	21	0
chr5	30438480	30486533	dre-circ-470	0	+	0	0	0	6	0
chr5	30438719	30456522	dre-circ-471	0	+	0	0	0	2	0
chr5	30438719	30470852	dre-circ-472	0	+	0	0	4	15	0
chr5	30470853	30489324	dre-circ-473	0	+	0	0	0	6	0
chr5	30513739	30516617	dre-circ-3816	0	-	0	0	3	0	0
chr5	31208524	31220408	dre-circ-474	0	-	0	0	0	0	3
chr5	3171075	3254327	dre-circ-446	0	-	2	0	0	0	0
chr5	32076223	32095267	dre-circ-475	0	-	0	0	2	0	0
chr5	32091056	32095267	dre-circ-3817	0	-	0	0	3	0	0
chr5	32723500	32724068	dre-circ-476	0	+	0	0	2	0	0
chr5	33307926	33320631	dre-circ-477	0	-	0	0	0	0	2
chr5	33851010	33866897	dre-circ-3818	0	+	0	0	0	678	0
chr5	33851010	33890263	dre-circ-478	0	+	0	0	0	180	0
chr5	33854480	33870577	dre-circ-3819	0	+	0	0	0	395	0

chr5	33860205	33885131	dre-circ-479	0	+	0	0	0	956	0
chr5	33861955	33885370	dre-circ-480	0	+	5	0	0	424	0
chr5	33862759	33886209	dre-circ-481	0	+	0	0	0	472	0
chr5	33863531	33886945	dre-circ-482	0	+	6	0	0	317	0
chr5	33863767	33887187	dre-circ-483	0	+	0	0	0	291	0
chr5	33864867	33888253	dre-circ-484	0	+	12	0	0	815	0
chr5	33866046	33889439	dre-circ-485	0	+	0	0	0	561	0
chr5	33866496	33889765	dre-circ-486	0	+	0	0	0	1141	0
chr5	33866752	33890023	dre-circ-487	0	+	0	0	0	1079	0
chr5	33866794	33890222	dre-circ-488	0	-	0	0	0	44	0
chr5	33868012	33891489	dre-circ-489	0	+	0	0	0	148	0
chr5	33870193	33893662	dre-circ-490	0	+	0	0	0	43	0
chr5	33870914	33894362	dre-circ-491	0	+	0	0	0	542	0
chr5	33871363	33894738	dre-circ-492	0	+	0	0	0	164	0
chr5	33910715	33928500	dre-circ-493	0	-	0	0	0	34	0
chr5	33920259	33936948	dre-circ-494	0	-	0	0	0	503	0
chr5	33921044	33937750	dre-circ-495	0	-	0	0	0	212	0
chr5	34102009	34104766	dre-circ-3820	0	-	0	0	3	3	0
chr5	341834	342394	dre-circ-434	0	-	0	0	0	0	2
chr5	35190903	35192885	dre-circ-3821	0	-	0	0	3	0	0
chr5	38929341	38932037	dre-circ-496	0	-	2	0	0	0	0
chr5	3914663	3915366	dre-circ-3822	0	+	5	2	2	0	3
chr5	39921264	39928095	dre-circ-3823	0	-	0	0	0	0	3
chr5	39947516	39953179	dre-circ-497	0	-	0	0	2	0	0
chr5	40232595	40280840	dre-circ-498	0	+	0	0	2	0	5
chr5	40259325	40281404	dre-circ-499	0	+	0	0	0	0	2
chr5	40403560	40411886	dre-circ-500	0	-	2	0	0	0	0
chr5	40618764	40620228	dre-circ-3824	0	+	0	2	2	0	0
chr5	42943100	43019137	dre-circ-501	0	+	0	0	2	0	0
chr5	43206745	43207406	dre-circ-3825	0	-	2	0	0	0	0
chr5	43451323	43454582	dre-circ-3826	0	-	0	0	0	0	15
chr5	43451577	43454814	dre-circ-502	0	-	0	0	0	0	41
chr5	43451577	43458170	dre-circ-3827	0	-	0	0	2	0	0
chr5	43509901	43510269	dre-circ-3828	0	-	4	0	0	0	0
chr5	43520425	43536300	dre-circ-503	0	-	0	0	2	0	0
chr5	43528886	43553265	dre-circ-3829	0	-	0	0	3	0	2
chr5	43540922	43546037	dre-circ-3830	0	-	3	0	0	0	0
chr5	43540922	43553262	dre-circ-3831	0	-	0	0	4	0	0
chr5	43540922	43553265	dre-circ-3832	0	-	0	0	3	0	0
chr5	43545975	43553262	dre-circ-3833	0	-	0	0	4	0	0
chr5	43545975	43553265	dre-circ-3834	0	-	2	0	4	0	0
chr5	43550730	43553265	dre-circ-3835	0	-	0	0	0	2	0
chr5	43685882	43686109	dre-circ-504	0	+	0	0	0	0	3
chr5	43686833	43714665	dre-circ-505	0	-	0	0	3	0	0
chr5	43748667	43755060	dre-circ-506	0	+	0	0	0	2	0
chr5	47743546	47743933	dre-circ-3836	0	+	0	0	4	5	0
chr5	47889684	47905567	dre-circ-507	0	+	0	0	2	0	0
chr5	47891678	47909000	dre-circ-508	0	+	0	0	0	0	2
chr5	47895789	47913504	dre-circ-509	0	+	0	0	0	0	3
chr5	52503553	52503670	dre-circ-510	0	-	0	0	0	0	3
chr5	54886169	54886809	dre-circ-3837	0	+	0	0	2	0	0
chr5	55638224	55644350	dre-circ-3838	0	-	0	0	0	8	0
chr5	57697120	57706267	dre-circ-511	0	+	0	0	0	2	0
chr5	58344091	58346373	dre-circ-3839	0	-	0	0	3	0	0
chr5	58500893	58501424	dre-circ-3840	0	-	2	0	0	0	0
chr5	58609255	58636358	dre-circ-512	0	-	10	0	9	3	11
chr5	58786699	58790111	dre-circ-3841	0	+	6	0	0	0	0
chr5	59104513	59104984	dre-circ-3842	0	+	2	0	0	0	0
chr5	60712872	60713100	dre-circ-513	0	-	0	0	0	2	0
chr5	60756900	60757010	dre-circ-514	0	+	0	2	0	0	0
chr5	60943911	60946160	dre-circ-3843	0	+	0	0	0	0	2
chr5	6126002	6129825	dre-circ-3844	0	-	0	2	0	0	0
chr5	61277301	61282868	dre-circ-515	0	+	2	0	0	0	0
chr5	62670807	62670932	dre-circ-516	0	+	0	0	2	0	0
chr5	63698808	63699038	dre-circ-3845	0	+	0	4	0	0	0
chr5	63825063	63844727	dre-circ-517	0	-	0	0	2	0	0
chr5	64438503	64442866	dre-circ-3846	0	+	4	0	0	0	0
chr5	64523982	64524506	dre-circ-518	0	+	0	0	0	0	2
chr5	65374229	65374490	dre-circ-3847	0	-	0	0	0	6	0
chr5	65693416	65693677	dre-circ-3848	0	+	0	0	2	0	0
chr5	65693416	65695571	dre-circ-519	0	+	0	0	2	0	0
chr5	66510099	66529496	dre-circ-520	0	+	5	2	3	4	14
chr5	66510525	66531248	dre-circ-521	0	+	0	0	4	0	6
chr5	66510848	66531579	dre-circ-522	0	+	6	0	0	3	7
chr5	66514861	66531946	dre-circ-3849	0	+	3	2	0	0	8
chr5	66567546	66581558	dre-circ-523	0	+	0	0	0	0	9
chr5	66568287	66582268	dre-circ-524	0	+	0	0	0	0	4
chr5	66716866	66719466	dre-circ-3850	0	+	0	3	0	8	0
chr5	66732312	66737099	dre-circ-525	0	+	0	0	2	0	0
chr5	66735550	66737099	dre-circ-3851	0	+	0	0	4	2	0
chr5	67505699	67520725	dre-circ-526	0	-	0	0	0	0	5
chr5	67507374	67521288	dre-circ-527	0	-	0	0	0	0	10
chr5	67507928	67523993	dre-circ-3852	0	-	6	0	0	0	91
chr5	67513662	67526980	dre-circ-528	0	-	0	0	0	0	21
chr5	67513662	67543644	dre-circ-529	0	-	0	0	0	0	8
chr5	67526886	67542921	dre-circ-3853	0	-	8	0	0	0	123
chr5	67645374	67647180	dre-circ-530	0	-	0	0	0	2	0

chr5	68679840	68680186	dre-circ-3854	0	-	0	0	7	3	0
chr5	68707103	68713097	dre-circ-3855	0	-	0	0	7	0	0
chr5	69080849	69083200	dre-circ-531	0	+	0	0	0	0	2
chr5	69227613	69227963	dre-circ-3856	0	-	0	2	0	0	0
chr5	69313958	69323056	dre-circ-532	0	+	0	0	0	0	2
chr5	69327179	69328222	dre-circ-533	0	+	0	0	0	0	2
chr5	70272908	70275222	dre-circ-3857	0	-	2	0	0	0	0
chr5	70336724	70336999	dre-circ-3858	0	-	17	2	5	7	0
chr5	70565197	70565386	dre-circ-3859	0	-	13	0	0	0	0
chr5	70877388	70877903	dre-circ-534	0	+	0	0	0	0	2
chr5	70990849	70990997	dre-circ-535	0	-	2	0	0	0	0
chr5	71129147	71130051	dre-circ-536	0	-	0	0	2	0	0
chr5	71811845	71816733	dre-circ-3860	0	-	2	2	0	0	0
chr5	71879017	71879565	dre-circ-537	0	-	3	0	0	0	0
chr5	71884825	71885036	dre-circ-3861	0	-	5	0	0	0	0
chr5	71898342	71900972	dre-circ-538	0	-	2	0	0	0	0
chr5	71899332	71900972	dre-circ-3862	0	-	2	0	2	0	0
chr5	71911542	71921260	dre-circ-539	0	-	2	0	0	0	0
chr5	71911542	71923841	dre-circ-540	0	-	2	0	0	0	0
chr5	71913199	71914524	dre-circ-3863	0	-	4	0	0	0	0
chr5	72023715	72027118	dre-circ-3864	0	-	0	3	0	0	3
chr5	72304100	72379006	dre-circ-3865	0	-	2	0	0	0	5
chr5	72426330	72427992	dre-circ-3866	0	-	3	0	0	2	2
chr5	72486659	72557196	dre-circ-541	0	-	0	0	5	0	7
chr5	72532221	72551996	dre-circ-542	0	-	0	0	2	0	0
chr5	72607076	72613649	dre-circ-543	0	-	0	0	0	0	7
chr5	72666181	72753484	dre-circ-544	0	+	22	0	0	0	4
chr5	72666583	72754812	dre-circ-545	0	+	11	0	0	0	0
chr5	7363549	7368129	dre-circ-3867	0	+	0	3	0	0	2
chr5	75452412	75455412	dre-circ-3868	0	+	0	0	4	0	0
chr5	75573230	75575687	dre-circ-546	0	+	2	0	0	0	0
chr5	823925	826025	dre-circ-435	0	+	13	3	2	3	7
chr5	8761075	8842843	dre-circ-3869	0	-	2	0	0	0	31
chr5	9329674	9339736	dre-circ-447	0	+	0	0	0	0	2
chr5	9413340	9415655	dre-circ-3870	0	+	2	0	0	2	2
chr5	9643786	9703158	dre-circ-448	0	+	0	0	2	0	0
chr5	9646977	9706514	dre-circ-449	0	+	33	0	0	2	0
chr6	11047371	11049652	dre-circ-561	0	-	0	2	0	0	0
chr6	11708734	11711368	dre-circ-3871	0	-	4	0	0	0	0
chr6	12428288	12441183	dre-circ-562	0	+	2	0	0	0	0
chr6	14031989	14035713	dre-circ-563	0	-	0	2	0	0	0
chr6	15724335	15739944	dre-circ-3872	0	+	0	3	0	0	0
chr6	16354354	16360477	dre-circ-564	0	-	2	0	0	0	0
chr6	16402664	16403034	dre-circ-3873	0	-	2	2	0	0	2
chr6	1646048	1647088	dre-circ-547	0	-	2	0	0	0	0
chr6	1707855	1709708	dre-circ-3874	0	+	2	3	7	0	2
chr6	1711103	1711916	dre-circ-548	0	-	0	0	10	0	0
chr6	1719250	1720559	dre-circ-549	0	+	0	0	0	0	2
chr6	1753946	1756100	dre-circ-550	0	-	2	0	0	0	0
chr6	17663320	17670630	dre-circ-3875	0	+	3	0	8	4	0
chr6	1890211	1895151	dre-circ-3876	0	+	0	0	2	0	0
chr6	19040812	19047056	dre-circ-3877	0	+	0	0	8	0	0
chr6	19046399	19047056	dre-circ-3878	0	+	0	0	0	2	0
chr6	1983930	1984797	dre-circ-3879	0	-	0	0	0	0	3
chr6	19842693	19845894	dre-circ-3880	0	+	0	0	0	0	2
chr6	20246911	20303495	dre-circ-565	0	-	2	0	0	3	12
chr6	2227543	2230541	dre-circ-3881	0	-	9	2	3	0	8
chr6	22468490	22471024	dre-circ-3882	0	-	0	0	3	16	0
chr6	22671183	22671881	dre-circ-3883	0	+	20	0	4	3	0
chr6	22693057	22707594	dre-circ-566	0	+	0	0	0	0	15
chr6	22693057	22747939	dre-circ-567	0	+	0	0	0	0	7
chr6	22800431	22804823	dre-circ-568	0	+	0	0	2	0	0
chr6	22800431	22806443	dre-circ-3884	0	+	0	0	14	3	0
chr6	23030248	23037807	dre-circ-569	0	+	0	0	0	2	0
chr6	2317201	2320815	dre-circ-3885	0	+	3	0	0	0	0
chr6	2317201	2339474	dre-circ-551	0	+	2	0	0	0	0
chr6	26591690	26592008	dre-circ-570	0	-	0	0	0	0	2
chr6	26896691	26898025	dre-circ-571	0	+	0	0	2	0	0
chr6	26896691	26907884	dre-circ-572	0	+	0	2	0	0	0
chr6	27870489	27881158	dre-circ-3886	0	+	4	0	0	0	2
chr6	27891395	27892010	dre-circ-3887	0	+	3	0	4	2	0
chr6	28396562	28400055	dre-circ-3888	0	+	3	0	0	0	0
chr6	29009363	29011363	dre-circ-3889	0	-	0	0	4	3	0
chr6	29051188	29052314	dre-circ-573	0	-	2	0	0	0	0
chr6	29056516	29058303	dre-circ-574	0	-	0	0	0	2	0
chr6	29847686	29853397	dre-circ-3890	0	-	0	2	0	0	0
chr6	30119707	30123022	dre-circ-3891	0	+	0	0	0	0	2
chr6	31006227	31016153	dre-circ-575	0	-	0	0	0	2	0
chr6	3126785	3127781	dre-circ-552	0	-	2	0	0	0	0
chr6	3174029	3182228	dre-circ-3892	0	+	2	0	0	0	0
chr6	3182042	3182228	dre-circ-3893	0	+	8	0	0	0	0
chr6	32028432	32029760	dre-circ-3894	0	+	0	0	3	0	0
chr6	32471261	32471399	dre-circ-576	0	-	0	0	0	0	2
chr6	33246561	33248630	dre-circ-3895	0	+	0	4	0	0	0
chr6	33388510	33395005	dre-circ-577	0	-	2	0	0	0	0
chr6	33511025	33512911	dre-circ-3896	0	-	2	0	0	0	0
chr6	35045381	35057898	dre-circ-578	0	-	0	2	0	0	0

chr6	37344119	37358480	dre-circ-3897	0	-	0	0	0	0	3
chr6	37460619	37465923	dre-circ-3898	0	-	3	0	0	0	0
chr6	37461220	37465923	dre-circ-3899	0	-	0	0	0	0	2
chr6	37594394	37596849	dre-circ-579	0	+	0	0	0	0	2
chr6	37705915	37706241	dre-circ-3900	0	+	0	0	0	2	0
chr6	38399562	38407908	dre-circ-580	0	+	0	2	0	0	0
chr6	39067035	39068837	dre-circ-3901	0	-	12	0	0	0	0
chr6	39070782	39075437	dre-circ-3902	0	-	2	0	0	0	0
chr6	39425582	39437481	dre-circ-581	0	+	0	0	0	0	3
chr6	40361900	40365569	dre-circ-582	0	-	0	0	0	0	2
chr6	40511827	40512257	dre-circ-3903	0	+	7	0	0	0	0
chr6	40680832	40684744	dre-circ-583	0	-	0	0	0	0	2
chr6	40849755	40850529	dre-circ-584	0	-	0	0	2	0	0
chr6	40861882	40869279	dre-circ-3904	0	+	0	0	0	0	2
chr6	41032493	41032605	dre-circ-585	0	+	2	0	0	4	0
chr6	41416897	41424334	dre-circ-586	0	+	0	0	3	0	0
chr6	41422592	41424334	dre-circ-3905	0	+	2	0	13	15	0
chr6	4225179	4225454	dre-circ-3906	0	+	2	0	0	0	0
chr6	43386982	43405568	dre-circ-587	0	+	0	0	0	2	0
chr6	43633739	43723515	dre-circ-588	0	-	2	0	0	0	0
chr6	43913841	43916700	dre-circ-589	0	-	0	0	0	0	2
chr6	43945566	43946013	dre-circ-3907	0	-	5	0	2	0	0
chr6	45262603	45265503	dre-circ-3908	0	+	0	0	2	0	0
chr6	45274464	45363011	dre-circ-3909	0	+	0	0	0	0	3
chr6	45285728	45297930	dre-circ-3910	0	+	0	2	0	0	0
chr6	46508091	46519656	dre-circ-590	0	-	0	0	0	0	2
chr6	46508091	46522867	dre-circ-591	0	-	0	0	0	0	2
chr6	47432861	47433092	dre-circ-3911	0	-	2	0	0	0	0
chr6	47437137	47437310	dre-circ-3912	0	-	127	0	37	8	7
chr6	49691325	49692147	dre-circ-592	0	+	2	0	0	0	0
chr6	5023554	5023697	dre-circ-553	0	-	0	4	0	0	0
chr6	52229323	52236347	dre-circ-593	0	+	2	0	0	0	0
chr6	52262367	52269110	dre-circ-3913	0	+	5	0	19	2	0
chr6	52262367	52277368	dre-circ-594	0	+	0	0	13	0	0
chr6	52267458	52283362	dre-circ-595	0	+	6	0	5	0	0
chr6	52269194	52277368	dre-circ-596	0	+	12	2	20	9	3
chr6	54701358	54703294	dre-circ-597	0	+	0	0	0	0	2
chr6	55212519	55215687	dre-circ-598	0	-	2	0	0	0	0
chr6	55283038	55283626	dre-circ-3914	0	-	0	0	2	0	0
chr6	55283038	55289121	dre-circ-3915	0	-	8	0	0	0	0
chr6	55698845	55699572	dre-circ-599	0	-	0	0	2	6	0
chr6	56048470	56057673	dre-circ-3916	0	+	3	0	0	0	0
chr6	56055846	56057673	dre-circ-3917	0	+	5	0	0	0	0
chr6	59015721	59016021	dre-circ-600	0	+	0	0	0	0	2
chr6	59251674	59255831	dre-circ-601	0	+	0	0	0	2	0
chr6	59402287	59407048	dre-circ-3918	0	+	5	0	0	0	0
chr6	59406882	59407048	dre-circ-3919	0	+	2	0	0	0	0
chr6	59576154	59576276	dre-circ-602	0	+	0	2	0	0	0
chr6	59661848	59662466	dre-circ-603	0	+	4	0	0	0	0
chr6	59674611	59693920	dre-circ-3920	0	+	2	0	0	0	0
chr6	6859391	6864548	dre-circ-3921	0	+	0	0	4	0	0
chr6	6890501	6906850	dre-circ-554	0	+	2	0	0	0	5
chr6	6891327	6907472	dre-circ-555	0	-	64	4	47	29	128
chr6	7436604	7438554	dre-circ-556	0	+	0	0	0	0	2
chr6	7630849	7632082	dre-circ-557	0	-	0	0	4	0	0
chr6	7965796	7966246	dre-circ-3922	0	+	0	8	0	0	0
chr6	8444873	8529659	dre-circ-558	0	+	0	0	0	0	6
chr6	8639592	8649569	dre-circ-559	0	-	0	0	2	0	0
chr6	9873103	9875979	dre-circ-560	0	+	0	0	2	0	0
chr7	1087930	1088156	dre-circ-3923	0	-	3	0	0	0	0
chr7	11608325	11618700	dre-circ-3924	0	+	0	0	0	0	8
chr7	11694185	11694634	dre-circ-621	0	+	0	0	0	3	0
chr7	12110349	12113231	dre-circ-3925	0	+	0	0	2	2	0
chr7	13776783	13780652	dre-circ-622	0	+	0	0	2	0	0
chr7	14440856	14490689	dre-circ-623	0	+	0	0	0	0	5
chr7	14465141	14515259	dre-circ-3926	0	-	0	0	3	4	5
chr7	14553352	14563524	dre-circ-3927	0	-	0	0	2	0	2
chr7	14878384	14878697	dre-circ-3928	0	+	9	0	0	0	3
chr7	14992861	14993151	dre-circ-3929	0	+	4	0	0	0	0
chr7	15253039	15253182	dre-circ-624	0	+	0	2	0	0	0
chr7	16255389	16255514	dre-circ-625	0	+	0	0	0	0	3
chr7	16325541	16325972	dre-circ-626	0	+	0	2	0	0	0
chr7	1644374	1669935	dre-circ-607	0	+	0	0	0	0	18
chr7	16533008	16550235	dre-circ-3930	0	+	0	0	3	0	38
chr7	16955670	16975512	dre-circ-627	0	-	0	0	0	0	3
chr7	1700444	1725435	dre-circ-608	0	+	0	0	0	0	19
chr7	17313922	17314395	dre-circ-3931	0	+	14	0	2	0	0
chr7	17564937	17566279	dre-circ-628	0	+	0	0	2	0	0
chr7	20571251	20571376	dre-circ-629	0	-	2	0	0	0	0
chr7	20653712	20660373	dre-circ-630	0	+	0	0	4	0	0
chr7	20653955	20660620	dre-circ-631	0	+	2	0	25	0	4
chr7	20656650	20661316	dre-circ-632	0	+	0	0	8	0	8
chr7	20657351	20661610	dre-circ-633	0	+	0	0	0	0	2
chr7	20842668	20846848	dre-circ-634	0	-	0	0	4	0	0
chr7	20846209	20850711	dre-circ-635	0	-	0	0	3	0	0
chr7	21227328	21227839	dre-circ-3932	0	-	0	0	2	0	0
chr7	21244899	21248318	dre-circ-636	0	-	0	0	0	0	2

chr7	21617700	21618537	dre-circ-3933	0	-	3	0	0	0	0
chr7	22026089	22028956	dre-circ-3934	0	+	0	0	3	0	0
chr7	22495071	22526595	dre-circ-637	0	+	0	0	0	0	2
chr7	22837017	22863364	dre-circ-3935	0	+	6	0	0	0	0
chr7	22954205	22954703	dre-circ-3936	0	-	0	0	2	0	0
chr7	23045136	23045599	dre-circ-3937	0	-	4	0	6	0	0
chr7	24861148	24866599	dre-circ-3938	0	+	0	0	0	0	2
chr7	24894840	24895621	dre-circ-3939	0	-	0	0	23	0	0
chr7	24941155	24956861	dre-circ-3940	0	-	2	0	0	0	0
chr7	25134532	25139567	dre-circ-3941	0	-	0	4	0	0	0
chr7	25258023	25258203	dre-circ-638	0	+	2	0	0	0	0
chr7	25543289	25545576	dre-circ-3942	0	-	2	0	0	0	0
chr7	25544048	25545576	dre-circ-3943	0	-	0	2	0	3	2
chr7	25613073	25613338	dre-circ-639	0	+	0	0	2	0	0
chr7	25765779	25766314	dre-circ-3944	0	-	3	0	2	0	0
chr7	25989583	25990079	dre-circ-640	0	-	0	0	0	2	0
chr7	26090135	26132101	dre-circ-641	0	+	0	0	0	2	0
chr7	26331329	26338905	dre-circ-3945	0	+	2	0	0	0	0
chr7	26335609	26352715	dre-circ-3946	0	+	2	0	0	0	0
chr7	26378972	26388903	dre-circ-642	0	+	2	0	0	0	0
chr7	26418542	26422343	dre-circ-3947	0	+	9	0	0	0	0
chr7	27455753	27460865	dre-circ-643	0	+	0	0	2	0	0
chr7	27719949	27722669	dre-circ-644	0	-	2	0	0	0	0
chr7	28158829	28162426	dre-circ-3948	0	-	0	2	0	2	0
chr7	28346523	28347519	dre-circ-3949	0	+	0	0	2	0	0
chr7	28446335	28453512	dre-circ-3950	0	+	6	0	0	0	0
chr7	28838425	28841965	dre-circ-3951	0	-	0	0	2	0	0
chr7	28850689	28854079	dre-circ-3952	0	-	5	0	2	0	0
chr7	28939796	28939969	dre-circ-645	0	+	2	0	0	0	0
chr7	29302984	29326158	dre-circ-646	0	-	0	0	0	2	0
chr7	29849261	29857614	dre-circ-647	0	-	0	0	0	0	2
chr7	30016037	30051002	dre-circ-648	0	-	0	0	3	0	4
chr7	31033877	31048090	dre-circ-649	0	-	0	0	0	2	0
chr7	31761777	31762488	dre-circ-3953	0	-	3	0	0	0	0
chr7	31881139	31881296	dre-circ-650	0	-	2	0	0	0	0
chr7	33137231	33137980	dre-circ-3954	0	-	2	0	0	0	0
chr7	33195018	33200137	dre-circ-3955	0	+	0	0	3	0	0
chr7	33196412	33200137	dre-circ-651	0	+	0	0	2	0	0
chr7	33196906	33200137	dre-circ-3956	0	+	0	0	40	0	0
chr7	34247685	34252201	dre-circ-652	0	+	0	0	0	0	2
chr7	34488473	34509529	dre-circ-3957	0	+	0	0	2	0	0
chr7	35311993	35312329	dre-circ-3958	0	+	4	0	0	0	0
chr7	35676424	35680037	dre-circ-653	0	-	0	0	3	0	0
chr7	36199981	36202580	dre-circ-3959	0	+	0	0	2	0	0
chr7	36308606	36309052	dre-circ-3960	0	-	2	0	0	0	0
chr7	36320489	36323253	dre-circ-3961	0	-	4	0	0	0	0
chr7	3732454	3775041	dre-circ-609	0	+	0	0	0	0	6
chr7	37884612	37885018	dre-circ-3962	0	+	3	0	0	0	0
chr7	38353765	38356518	dre-circ-3963	0	+	0	0	3	0	0
chr7	38353765	38356920	dre-circ-3964	0	+	0	2	3	0	0
chr7	39665725	39667704	dre-circ-654	0	+	2	0	0	0	0
chr7	39933081	39949426	dre-circ-3965	0	-	2	0	0	0	0
chr7	40029777	40035873	dre-circ-655	0	-	0	0	6	0	0
chr7	40101393	40101823	dre-circ-3966	0	-	2	0	0	0	0
chr7	40785315	40798560	dre-circ-656	0	+	10	0	0	649	0
chr7	40807182	40819806	dre-circ-3967	0	+	0	0	4	0	0
chr7	40846753	40847337	dre-circ-3968	0	+	0	0	0	6	0
chr7	40847082	40847156	dre-circ-657	0	+	0	0	0	3	0
chr7	4145872	4152942	dre-circ-3969	0	-	5	0	0	0	0
chr7	42102830	42102991	dre-circ-3970	0	+	2	0	0	0	0
chr7	42371412	42377543	dre-circ-3971	0	+	0	2	0	0	0
chr7	43199145	43207961	dre-circ-3972	0	-	3	0	0	0	0
chr7	43314631	43318026	dre-circ-658	0	+	2	0	0	0	0
chr7	43669749	43672283	dre-circ-3973	0	-	2	0	0	0	0
chr7	44142370	44143556	dre-circ-3974	0	-	2	2	0	0	0
chr7	44551705	44562321	dre-circ-659	0	-	0	0	2	0	0
chr7	4520945	4563716	dre-circ-610	0	+	0	0	0	0	2
chr7	4539810	4580539	dre-circ-611	0	+	0	0	0	0	2
chr7	50099090	50105552	dre-circ-3975	0	+	0	0	4	0	0
chr7	50484921	50490304	dre-circ-660	0	+	0	0	0	0	2
chr7	51364171	51364288	dre-circ-661	0	+	2	0	0	0	0
chr7	52061577	52072761	dre-circ-3976	0	-	2	0	0	0	0
chr7	52107465	52108971	dre-circ-3977	0	-	0	0	3	0	0
chr7	53132667	53132772	dre-circ-662	0	-	0	0	0	0	2
chr7	54181299	54186313	dre-circ-663	0	+	2	0	0	0	0
chr7	54365078	54377324	dre-circ-664	0	+	0	0	0	0	2
chr7	54897210	54980703	dre-circ-665	0	+	4	0	0	0	20
chr7	54898538	54898944	dre-circ-3978	0	+	2	0	0	0	0
chr7	56114301	56166738	dre-circ-666	0	-	0	0	0	0	2
chr7	56153059	56170371	dre-circ-667	0	-	0	0	0	0	3
chr7	56226868	56260980	dre-circ-668	0	+	7	0	3	7	94
chr7	56619236	56619727	dre-circ-3979	0	-	3	0	0	0	0
chr7	56620438	56621326	dre-circ-669	0	-	2	0	0	0	0
chr7	56870485	56873483	dre-circ-670	0	-	2	0	0	0	0
chr7	57634197	57641511	dre-circ-3980	0	+	7	0	0	0	0
chr7	58382550	58386793	dre-circ-671	0	+	0	0	2	0	0
chr7	58613818	58617967	dre-circ-672	0	-	0	0	0	3	0

chr7	58801415	58802939	dre-circ-673	0	+	0	0	0	0	2
chr7	59109060	59119234	dre-circ-3981	0	+	0	0	0	3	0
chr7	59197596	59208991	dre-circ-3982	0	-	0	0	5	0	0
chr7	59197596	59208994	dre-circ-3983	0	-	0	0	16	0	0
chr7	59204584	59208991	dre-circ-3984	0	-	0	0	9	0	0
chr7	59204584	59208994	dre-circ-3985	0	-	0	0	30	0	0
chr7	60062175	60062690	dre-circ-3986	0	-	9	3	2	0	0
chr7	60395781	60463969	dre-circ-674	0	+	0	0	0	2	0
chr7	60764089	60764407	dre-circ-3987	0	-	0	0	2	0	3
chr7	60880160	60881184	dre-circ-675	0	+	0	0	0	2	0
chr7	60884194	60885062	dre-circ-676	0	+	2	0	0	0	0
chr7	6520629	6546631	dre-circ-3988	0	+	0	0	0	0	3
chr7	65356230	65357283	dre-circ-677	0	-	0	0	3	3	3
chr7	65638586	65638896	dre-circ-3989	0	-	3	0	0	0	0
chr7	67001463	67006084	dre-circ-678	0	+	0	0	0	0	2
chr7	67220217	67220469	dre-circ-3990	0	-	0	0	0	2	0
chr7	67410842	67476398	dre-circ-679	0	-	2	0	0	0	0
chr7	6754640	6799451	dre-circ-612	0	+	2	0	0	0	83
chr7	68693356	68697099	dre-circ-680	0	+	0	0	0	0	2
chr7	69884023	69889380	dre-circ-681	0	-	2	0	0	0	0
chr7	70242962	70243506	dre-circ-682	0	+	0	0	4	0	0
chr7	71040806	71065717	dre-circ-3991	0	+	0	0	5	0	0
chr7	71121573	71122145	dre-circ-683	0	+	0	0	0	0	2
chr7	71121573	71175570	dre-circ-684	0	+	0	0	0	0	2
chr7	7267426	7289551	dre-circ-613	0	-	0	0	3	0	0
chr7	7268263	7290691	dre-circ-614	0	+	0	0	0	0	6
chr7	7281535	7284148	dre-circ-615	0	-	0	0	0	0	8
chr7	72889012	72896254	dre-circ-3992	0	+	0	0	0	0	17
chr7	730242	730938	dre-circ-3993	0	+	2	0	0	0	0
chr7	73738393	73739592	dre-circ-685	0	+	0	0	2	0	0
chr7	73982175	73983344	dre-circ-3994	0	-	0	0	2	0	0
chr7	74364252	74375145	dre-circ-3995	0	-	0	0	2	0	0
chr7	74386907	74390346	dre-circ-3996	0	-	0	0	13	3	0
chr7	74403327	74403661	dre-circ-686	0	-	0	0	33	11	0
chr7	74560232	74564243	dre-circ-3997	0	+	0	0	6	0	0
chr7	74578101	74580843	dre-circ-687	0	+	0	0	2	0	0
chr7	74737903	74741605	dre-circ-688	0	+	0	2	0	0	0
chr7	74741339	74744628	dre-circ-3998	0	+	2	5	0	0	0
chr7	74789773	74793822	dre-circ-689	0	-	0	0	2	0	2
chr7	75139930	75142405	dre-circ-3999	0	-	0	0	2	0	0
chr7	75170032	75170375	dre-circ-4000	0	-	14	0	2	0	2
chr7	75212330	75213373	dre-circ-4001	0	-	4	0	0	0	0
chr7	75282430	75284919	dre-circ-4002	0	+	2	0	0	0	3
chr7	76137832	76167440	dre-circ-4003	0	+	0	0	0	0	6
chr7	76185714	76218606	dre-circ-4004	0	+	2	0	0	0	3
chr7	76187278	76188753	dre-circ-4005	0	+	2	0	0	0	4
chr7	76712554	76716324	dre-circ-4006	0	-	0	0	0	0	2
chr7	76723975	76725764	dre-circ-4007	0	-	0	0	6	8	0
chr7	7674069	7675380	dre-circ-616	0	-	0	0	2	0	0
chr7	76783197	76784149	dre-circ-4008	0	+	0	2	0	0	0
chr7	76816281	76853612	dre-circ-690	0	-	0	0	0	0	13
chr7	76846668	76855407	dre-circ-691	0	-	0	0	0	0	14
chr7	7986362	7986617	dre-circ-617	0	-	0	0	0	0	2
chr7	8740273	8785433	dre-circ-4009	0	-	0	0	0	0	9
chr7	8762319	8800402	dre-circ-618	0	-	0	0	0	0	5
chr7	8772446	8803314	dre-circ-619	0	-	0	0	0	0	7
chr7	907108	907398	dre-circ-604	0	-	0	0	0	0	3
chr7	9102484	9103338	dre-circ-4010	0	-	3	0	0	0	0
chr7	918280	924511	dre-circ-605	0	-	0	0	0	0	2
chr7	918350	923233	dre-circ-606	0	+	0	0	0	0	3
chr7	9941133	9957383	dre-circ-620	0	+	0	0	0	0	2
chr8	10529696	10530308	dre-circ-704	0	+	0	0	0	0	2
chr8	11353326	11353524	dre-circ-4011	0	-	0	0	3	0	2
chr8	11678714	11687728	dre-circ-4012	0	+	4	2	8	0	2
chr8	12187596	12190762	dre-circ-4013	0	+	3	0	0	0	0
chr8	12191944	12192180	dre-circ-4014	0	+	3	0	0	0	0
chr8	12348719	12350302	dre-circ-4015	0	-	0	7	4	0	0
chr8	13916353	13917173	dre-circ-705	0	+	0	0	0	0	2
chr8	14628944	14667925	dre-circ-706	0	-	0	0	0	2	0
chr8	17038900	17039229	dre-circ-4016	0	-	2	0	2	0	0
chr8	17084835	17102453	dre-circ-707	0	-	0	0	0	0	2
chr8	17471397	17472507	dre-circ-708	0	+	0	0	2	0	0
chr8	18477761	18482203	dre-circ-709	0	-	0	0	2	0	0
chr8	18491223	18494371	dre-circ-4017	0	-	0	0	3	0	0
chr8	18494002	18495103	dre-circ-710	0	-	0	0	2	0	0
chr8	19051381	19052330	dre-circ-4018	0	-	0	0	23	3	0
chr8	1908283	1909186	dre-circ-4019	0	-	0	0	2	2	0
chr8	19783651	19786181	dre-circ-4020	0	-	11	0	3	2	4
chr8	20294232	20341050	dre-circ-711	0	+	0	0	0	0	2
chr8	2031084	2034228	dre-circ-4021	0	+	4	0	0	2	0
chr8	21064595	21084454	dre-circ-4022	0	+	0	3	0	0	0
chr8	21421409	21494500	dre-circ-712	0	+	2	0	0	0	0
chr8	21477687	21535402	dre-circ-713	0	+	0	0	0	0	2
chr8	21513575	21577284	dre-circ-714	0	+	0	0	0	0	2
chr8	21537282	21561745	dre-circ-4023	0	+	0	0	0	0	5
chr8	21592811	21595031	dre-circ-4024	0	-	0	0	3	2	9
chr8	21626376	21636399	dre-circ-715	0	-	0	0	0	0	3

chr8	21628587	21638178	dre-circ-716	0	-	7	0	13	8	9
chr8	21661523	21662058	dre-circ-4025	0	+	2	4	2	0	0
chr8	23012566	23046220	dre-circ-717	0	+	0	0	0	5	0
chr8	23060953	23080537	dre-circ-718	0	+	0	0	0	0	2
chr8	2341563	2360769	dre-circ-693	0	+	0	0	0	0	2
chr8	23659886	23679949	dre-circ-4026	0	-	0	0	0	0	2
chr8	24124163	24149349	dre-circ-719	0	-	0	0	0	0	2
chr8	24527626	24543916	dre-circ-720	0	+	0	0	2	0	0
chr8	24533791	24548157	dre-circ-4027	0	-	0	0	10	7	0
chr8	24534568	24552031	dre-circ-721	0	-	0	0	0	0	7
chr8	24536521	24552273	dre-circ-722	0	-	0	0	0	0	8
chr8	25166148	25209744	dre-circ-723	0	+	0	0	0	0	3
chr8	25168438	25245613	dre-circ-724	0	+	0	0	0	0	2
chr8	25609260	25613088	dre-circ-725	0	+	0	0	0	2	0
chr8	25643079	25650334	dre-circ-726	0	-	2	0	0	2	15
chr8	25934684	25935998	dre-circ-4028	0	+	0	0	2	0	0
chr8	26149996	26153303	dre-circ-727	0	+	51	0	24	0	5
chr8	26944501	26945186	dre-circ-728	0	+	4	0	0	0	0
chr8	27208468	27214520	dre-circ-4029	0	+	2	0	0	0	0
chr8	2760994	2761162	dre-circ-694	0	-	2	0	0	0	0
chr8	2761691	2761798	dre-circ-695	0	-	0	0	0	0	2
chr8	2775588	2786622	dre-circ-4030	0	+	3	0	0	0	0
chr8	28074869	28082363	dre-circ-729	0	-	0	0	0	2	0
chr8	28486157	28513322	dre-circ-730	0	-	0	0	2	0	0
chr8	28497582	28526395	dre-circ-731	0	-	0	0	3	0	0
chr8	32739732	32741861	dre-circ-732	0	+	0	2	0	0	0
chr8	32926851	32929273	dre-circ-4031	0	-	0	0	2	0	0
chr8	33167305	33172804	dre-circ-733	0	-	2	0	2	0	0
chr8	33291006	33296247	dre-circ-734	0	-	0	0	0	0	2
chr8	33291006	33305131	dre-circ-4032	0	-	0	0	0	0	14
chr8	33291535	33295730	dre-circ-735	0	-	0	0	0	0	3
chr8	33293308	33320000	dre-circ-736	0	-	0	0	0	0	10
chr8	33294781	33308899	dre-circ-737	0	-	0	0	0	0	5
chr8	33294781	33309929	dre-circ-4033	0	-	0	0	0	0	6
chr8	33295045	33300236	dre-circ-738	0	-	0	0	0	0	4
chr8	33295045	33305926	dre-circ-739	0	-	0	0	0	0	5
chr8	33295302	33296247	dre-circ-740	0	-	0	0	0	0	16
chr8	33295302	33300497	dre-circ-741	0	-	0	0	0	0	2
chr8	33295819	33300497	dre-circ-742	0	-	3	0	0	0	6
chr8	33295819	33302138	dre-circ-743	0	-	3	0	0	0	3
chr8	33295819	33320503	dre-circ-744	0	-	0	0	0	0	5
chr8	33296083	33307010	dre-circ-745	0	-	0	0	0	0	13
chr8	33304719	33306204	dre-circ-746	0	-	0	0	0	0	19
chr8	33307120	33309177	dre-circ-747	0	-	0	0	0	0	7
chr8	33309022	33319483	dre-circ-748	0	-	2	0	0	0	8
chr8	33309616	33311548	dre-circ-749	0	-	0	0	0	0	3
chr8	33320078	33320503	dre-circ-750	0	-	0	0	0	0	2
chr8	33650738	33725520	dre-circ-751	0	+	0	0	0	0	7
chr8	37469811	37474185	dre-circ-4034	0	+	0	0	0	0	6
chr8	4108421	4115495	dre-circ-696	0	+	0	2	0	0	0
chr8	413015	413949	dre-circ-692	0	-	2	0	0	0	0
chr8	43303053	43303588	dre-circ-752	0	-	2	0	0	0	0
chr8	43360892	43367867	dre-circ-4035	0	+	0	0	3	0	0
chr8	43874710	43874906	dre-circ-4036	0	-	0	0	0	0	2
chr8	44612523	44620876	dre-circ-753	0	-	2	0	0	0	0
chr8	45761100	45774519	dre-circ-754	0	+	0	0	0	0	3
chr8	45898932	45899255	dre-circ-755	0	+	0	0	0	0	2
chr8	45898932	45904256	dre-circ-4037	0	+	0	0	0	0	2
chr8	45932758	46013466	dre-circ-756	0	+	0	0	0	0	2
chr8	46563896	46584721	dre-circ-4038	0	-	0	0	6	0	0
chr8	46807752	46815303	dre-circ-757	0	-	2	0	0	0	0
chr8	46869215	46885616	dre-circ-758	0	-	2	0	0	0	0
chr8	47223751	47224584	dre-circ-4039	0	-	3	0	3	0	0
chr8	4763617	4764678	dre-circ-4040	0	-	0	0	2	0	0
chr8	48119328	48127017	dre-circ-4041	0	-	0	0	2	0	0
chr8	48556230	48560098	dre-circ-4042	0	+	0	2	0	0	0
chr8	48601122	48653364	dre-circ-4043	0	-	0	0	0	0	5
chr8	48603388	48655683	dre-circ-759	0	-	0	0	0	0	16
chr8	48653441	48668192	dre-circ-760	0	-	0	0	0	0	9
chr8	48653441	48713645	dre-circ-761	0	-	0	0	0	0	3
chr8	48653704	48668440	dre-circ-762	0	-	0	0	0	0	5
chr8	48668134	48713586	dre-circ-763	0	-	0	0	0	2	0
chr8	48668266	48678814	dre-circ-764	0	-	0	0	2	0	0
chr8	49137097	49139534	dre-circ-765	0	+	0	0	0	0	2
chr8	49315166	49315474	dre-circ-766	0	-	2	0	0	0	0
chr8	49842383	49842792	dre-circ-767	0	+	0	0	0	0	2
chr8	49931374	49934803	dre-circ-768	0	-	0	0	2	0	0
chr8	50069915	50087225	dre-circ-769	0	+	2	0	0	0	0
chr8	50069915	50091248	dre-circ-770	0	+	2	0	0	0	0
chr8	51183658	51186364	dre-circ-771	0	+	2	0	0	0	0
chr8	51281511	51281940	dre-circ-772	0	+	2	0	0	0	0
chr8	52044741	52047573	dre-circ-773	0	+	2	0	0	0	0
chr8	52167079	52178879	dre-circ-774	0	-	2	0	0	0	0
chr8	52683401	52687391	dre-circ-4044	0	+	0	0	0	2	2
chr8	55313597	55314726	dre-circ-4045	0	+	0	0	0	0	5
chr8	55371422	55377091	dre-circ-4046	0	-	4	0	0	0	0
chr8	6374982	6433723	dre-circ-4047	0	-	0	7	0	0	4

chr8	6432183	6433723	dre-circ-697	0	-	0	2	0	0	0
chr8	7242245	7254565	dre-circ-698	0	-	0	0	0	2	0
chr8	7614690	7615615	dre-circ-4048	0	+	0	0	0	7	0
chr8	8035872	8038832	dre-circ-699	0	-	0	0	0	0	2
chr8	8623282	8629190	dre-circ-700	0	+	0	0	0	0	2
chr8	8852370	8870400	dre-circ-701	0	+	0	0	2	0	0
chr8	9238607	9240682	dre-circ-702	0	-	2	0	0	0	0
chr8	9449655	9459840	dre-circ-4049	0	+	2	0	0	0	0
chr8	9520681	9538103	dre-circ-703	0	+	2	0	0	0	0
chr8	9637117	9638580	dre-circ-4050	0	-	0	3	0	0	0
chr9	10201818	10206681	dre-circ-797	0	-	0	0	0	2	0
chr9	10570242	10576183	dre-circ-798	0	-	0	0	2	0	0
chr9	1069789	1070013	dre-circ-4051	0	-	15	0	0	0	0
chr9	10930282	10934681	dre-circ-4052	0	+	10	0	0	0	0
chr9	1252189	1255543	dre-circ-776	0	+	0	0	0	2	0
chr9	13307444	13310151	dre-circ-799	0	+	0	0	0	0	2
chr9	14263914	14279485	dre-circ-800	0	+	0	0	0	0	9
chr9	14718483	14722726	dre-circ-4053	0	-	3	0	0	3	0
chr9	1477490	1483505	dre-circ-777	0	-	0	0	0	0	2
chr9	14845362	14845796	dre-circ-801	0	-	0	2	0	0	0
chr9	16607972	16650537	dre-circ-4054	0	+	0	0	2	0	2
chr9	1662693	1663038	dre-circ-4055	0	+	5	0	0	0	0
chr9	1667802	1670507	dre-circ-778	0	-	0	0	0	3	0
chr9	17674822	17680044	dre-circ-802	0	-	0	0	0	0	2
chr9	17689537	17694798	dre-circ-4056	0	-	2	0	0	0	0
chr9	17709274	17709732	dre-circ-803	0	-	2	0	0	0	0
chr9	18804996	18805569	dre-circ-4057	0	-	2	0	0	0	0
chr9	19510532	19511246	dre-circ-804	0	+	0	0	0	0	2
chr9	23346221	23346754	dre-circ-805	0	+	0	0	3	0	0
chr9	2379599	2380527	dre-circ-779	0	-	2	0	0	0	0
chr9	23879764	23884024	dre-circ-806	0	-	0	0	0	12	0
chr9	23879764	23885293	dre-circ-807	0	-	0	0	0	41	0
chr9	23879764	23886371	dre-circ-808	0	-	0	0	0	26	0
chr9	23879764	23887589	dre-circ-809	0	-	0	0	0	7	0
chr9	23879764	23889708	dre-circ-810	0	-	0	0	0	9	0
chr9	23881426	23884024	dre-circ-811	0	-	0	0	0	24	0
chr9	23881426	23886371	dre-circ-812	0	-	0	0	0	2	0
chr9	23883713	23887169	dre-circ-4058	0	-	0	0	0	46	0
chr9	23884152	23885293	dre-circ-813	0	-	0	0	0	14	0
chr9	23884152	23886371	dre-circ-814	0	-	0	0	0	14	0
chr9	23884152	23887589	dre-circ-815	0	-	0	0	0	12	0
chr9	23884152	23889708	dre-circ-816	0	-	0	0	0	9	0
chr9	23885387	23886371	dre-circ-817	0	-	0	0	0	21	0
chr9	23885387	23887589	dre-circ-818	0	-	0	0	0	4	0
chr9	23885387	23889708	dre-circ-819	0	-	0	0	0	8	0
chr9	23886060	23887169	dre-circ-820	0	-	0	0	0	15	0
chr9	23886060	23890507	dre-circ-821	0	-	0	0	0	11	0
chr9	23886479	23887589	dre-circ-822	0	-	0	0	0	5	0
chr9	23886479	23889708	dre-circ-823	0	-	2	0	0	24	0
chr9	23887278	23890507	dre-circ-824	0	-	0	0	0	17	0
chr9	23887684	23889708	dre-circ-825	0	-	0	0	0	64	0
chr9	23893726	23894782	dre-circ-826	0	-	0	0	0	59	0
chr9	23939481	23939805	dre-circ-4059	0	-	30	0	2	0	0
chr9	25121407	25128538	dre-circ-827	0	+	0	0	0	0	2
chr9	26187345	26188315	dre-circ-4060	0	+	0	0	3	0	2
chr9	26359710	26359880	dre-circ-4061	0	-	21	0	0	2	3
chr9	27424344	27478379	dre-circ-828	0	+	2	0	0	0	0
chr9	28913340	28913669	dre-circ-4062	0	+	2	0	0	0	0
chr9	29204017	29219459	dre-circ-829	0	+	0	0	2	0	0
chr9	29301724	29304511	dre-circ-4063	0	-	0	0	2	0	0
chr9	29716597	29718479	dre-circ-830	0	+	0	0	0	2	0
chr9	3086397	3088676	dre-circ-4064	0	+	0	0	3	0	14
chr9	31269549	31269775	dre-circ-831	0	-	2	0	0	0	0
chr9	3130354	3130879	dre-circ-780	0	+	0	8	0	0	0
chr9	3130809	3131728	dre-circ-781	0	-	24	3	0	0	2
chr9	3130809	3132637	dre-circ-782	0	-	13	2	0	0	5
chr9	3131219	3131595	dre-circ-783	0	+	0	0	2	0	0
chr9	3133559	3134641	dre-circ-784	0	-	2	0	0	0	0
chr9	3133623	3134641	dre-circ-785	0	-	2	0	0	0	0
chr9	31632729	31639907	dre-circ-832	0	+	0	0	0	2	0
chr9	31632729	31655944	dre-circ-833	0	+	0	0	0	2	0
chr9	31655790	31655944	dre-circ-834	0	+	2	0	0	0	0
chr9	3173709	3182313	dre-circ-786	0	+	0	0	0	0	2
chr9	3173709	3187527	dre-circ-787	0	+	0	0	0	0	2
chr9	31791264	31798224	dre-circ-4065	0	+	0	2	0	0	0
chr9	32075886	32088374	dre-circ-4066	0	+	0	0	2	0	0
chr9	33679215	33679586	dre-circ-835	0	-	2	0	0	0	0
chr9	33691252	33703983	dre-circ-836	0	-	0	0	3	0	0
chr9	33841425	33858402	dre-circ-837	0	-	0	0	4	0	13
chr9	33844548	33870031	dre-circ-838	0	-	0	0	11	0	35
chr9	34250547	34253854	dre-circ-839	0	+	0	0	0	0	338
chr9	34251453	34254855	dre-circ-840	0	-	0	0	0	0	13
chr9	34254363	34270787	dre-circ-841	0	+	0	0	0	0	10
chr9	34403322	34415076	dre-circ-4067	0	-	0	0	4	0	0
chr9	35175650	35176876	dre-circ-4068	0	-	4	0	6	2	0
chr9	35879756	35883253	dre-circ-4069	0	-	0	2	0	0	0
chr9	35989006	35991369	dre-circ-4070	0	+	4	0	3	5	0

chr9	38397453	38397627	dre-circ-4071	0	+	0	0	0	0	2
chr9	38419567	38468976	dre-circ-842	0	+	2	0	0	0	0
chr9	38867741	38872625	dre-circ-843	0	+	0	0	2	0	0
chr9	39465162	39473565	dre-circ-844	0	+	0	0	3	0	3
chr9	39465162	39501408	dre-circ-845	0	+	0	0	0	3	2
chr9	39468327	39482320	dre-circ-846	0	+	0	0	5	3	3
chr9	39485403	39508554	dre-circ-847	0	+	0	0	5	0	0
chr9	3998769	3998910	dre-circ-788	0	-	2	0	0	0	0
chr9	4015268	4019583	dre-circ-4072	0	-	4	0	0	0	0
chr9	4017788	4019583	dre-circ-789	0	-	2	0	0	0	0
chr9	4052962	4055399	dre-circ-4073	0	-	0	0	7	0	0
chr9	40542101	40549620	dre-circ-848	0	+	0	2	0	0	0
chr9	4104875	4108984	dre-circ-4074	0	+	0	0	0	0	3
chr9	41811311	41827299	dre-circ-849	0	-	0	0	0	0	2
chr9	42306014	42331903	dre-circ-850	0	+	0	0	10	0	0
chr9	42309061	42336629	dre-circ-851	0	-	0	0	0	0	3
chr9	42311496	42336667	dre-circ-852	0	+	0	0	0	0	6
chr9	42383409	42396789	dre-circ-4075	0	-	2	0	2	0	0
chr9	42386504	42396789	dre-circ-4076	0	-	2	0	0	0	0
chr9	427756	428077	dre-circ-775	0	+	0	0	0	0	2
chr9	43837099	43837223	dre-circ-853	0	+	0	0	3	0	0
chr9	43904725	43904877	dre-circ-854	0	+	0	0	0	3	0
chr9	43935054	43935173	dre-circ-855	0	-	0	0	0	2	0
chr9	43976636	43976798	dre-circ-856	0	-	0	0	0	2	0
chr9	44037251	44037382	dre-circ-857	0	-	0	0	35	21	0
chr9	44073216	44073326	dre-circ-858	0	-	0	0	2	0	0
chr9	44073216	44074946	dre-circ-859	0	-	0	0	0	6	0
chr9	44073560	44073667	dre-circ-860	0	-	0	0	5	0	0
chr9	44074421	44074531	dre-circ-861	0	-	0	0	0	3	0
chr9	44074421	44074946	dre-circ-862	0	-	0	0	9	0	0
chr9	44074836	44074937	dre-circ-863	0	-	0	0	3	0	0
chr9	44074836	44074946	dre-circ-864	0	-	0	0	10	2	0
chr9	44114911	44140443	dre-circ-865	0	-	0	0	0	0	2
chr9	44180265	44180927	dre-circ-4077	0	-	0	0	2	0	0
chr9	44872872	44874062	dre-circ-4078	0	+	2	0	0	0	0
chr9	453892	454300	dre-circ-4079	0	+	2	0	0	0	0
chr9	45571417	45577735	dre-circ-4080	0	-	0	0	3	0	0
chr9	46586034	46587006	dre-circ-866	0	+	0	0	0	2	0
chr9	46701340	46707866	dre-circ-867	0	+	0	0	0	0	3
chr9	47009256	47016868	dre-circ-868	0	-	2	0	0	0	0
chr9	47064793	47065393	dre-circ-4081	0	-	13	0	4	0	3
chr9	48645068	48645830	dre-circ-4082	0	-	0	0	0	2	2
chr9	49042254	49044824	dre-circ-869	0	-	2	0	0	0	2
chr9	49374789	49378472	dre-circ-870	0	-	0	2	0	0	0
chr9	50853213	50853447	dre-circ-4083	0	-	0	0	0	0	2
chr9	51030756	51033130	dre-circ-871	0	+	2	0	0	0	0
chr9	51056832	51057211	dre-circ-4084	0	+	7	0	0	0	0
chr9	51472899	51476130	dre-circ-4085	0	+	0	0	2	0	0
chr9	52889603	52908445	dre-circ-4086	0	-	0	0	2	0	0
chr9	53949458	53998246	dre-circ-872	0	+	0	0	0	0	3
chr9	54809033	54809376	dre-circ-4087	0	-	4	0	0	0	0
chr9	55311462	55324784	dre-circ-4088	0	-	0	0	0	0	2
chr9	56415912	56416403	dre-circ-4089	0	-	0	0	0	3	0
chr9	56725952	56733379	dre-circ-4090	0	+	2	0	0	0	0
chr9	56739105	56745358	dre-circ-4091	0	+	0	0	0	0	2
chr9	58001623	58001850	dre-circ-4092	0	-	8	0	0	0	0
chr9	58110797	58179977	dre-circ-4093	0	-	82	6	67	49	104
chr9	597804	598360	dre-circ-4094	0	-	0	0	2	0	0
chr9	6142779	6150315	dre-circ-4095	0	+	0	0	2	0	0
chr9	6324141	6336945	dre-circ-4096	0	+	0	0	2	0	0
chr9	6652755	6652905	dre-circ-790	0	+	2	0	0	0	0
chr9	6994284	6994717	dre-circ-791	0	+	0	0	2	0	0
chr9	7025385	7025941	dre-circ-792	0	-	0	0	0	0	2
chr9	786634	790254	dre-circ-4097	0	-	0	0	12	3	6
chr9	8075101	8090990	dre-circ-793	0	+	0	2	0	0	0
chr9	8434214	8452441	dre-circ-794	0	-	0	0	0	0	9
chr9	912347	917309	dre-circ-4098	0	-	0	0	6	0	0
chr9	9431200	9431655	dre-circ-4099	0	-	0	2	0	0	0
chr9	9776461	9783356	dre-circ-795	0	+	0	0	0	2	0
chr9	9969135	9996191	dre-circ-796	0	-	0	0	2	0	0
Zv9_NA1	4162	4733	dre-circ-4100	0	+	15	0	15	7	6
Zv9_NA1	4607	4846	dre-circ-2182	0	-	0	0	2	0	0
Zv9_NA1	732	2873	dre-circ-4101	0	+	0	4	3	3	7
Zv9_NA1	70	2170	dre-circ-2179	0	+	2	0	0	0	0
Zv9_NA1	73	466	dre-circ-4102	0	+	2	0	0	0	0
Zv9_NA2	7599	7712	dre-circ-2183	0	-	0	0	0	0	2
Zv9_NA2	14179	15693	dre-circ-4103	0	-	4	0	0	0	11
Zv9_NA2	15583	15693	dre-circ-2184	0	-	3	0	0	0	25
Zv9_NA3	43701	44543	dre-circ-2185	0	+	0	0	2	0	0
Zv9_NA3	5783	8164	dre-circ-4104	0	+	8	0	2	7	9
Zv9_NA3	49575	49820	dre-circ-4105	0	-	0	0	0	0	2
Zv9_NA3	50398	50538	dre-circ-2186	0	-	2	0	0	0	0
Zv9_NA3	13047	14772	dre-circ-4106	0	-	2	0	0	0	0
Zv9_NA3	4852	13221	dre-circ-4107	0	-	9	0	0	0	0
Zv9_NA3	12488	13669	dre-circ-2187	0	+	0	0	0	0	2
Zv9_NA3	48864	58142	dre-circ-4108	0	+	0	0	0	2	0
Zv9_NA4	15984	16405	dre-circ-4109	0	-	0	0	3	0	2

Zv9_NA4	3657	4045	dre-circ-4110	0	-	0	0	3	0	44
Zv9_NA4	6486	13009	dre-circ-2189	0	-	0	0	0	0	8
Zv9_NA4	6486	9445	dre-circ-2188	0	-	0	0	2	0	2
Zv9_NA4	3153	3303	dre-circ-2190	0	-	2	0	0	0	0
Zv9_NA4	3177	14483	dre-circ-2191	0	-	0	0	0	0	4
Zv9_NA4	5096	5492	dre-circ-2192	0	-	2	0	0	0	0
Zv9_NA5	417	803	dre-circ-4111	0	-	0	0	0	0	4
Zv9_NA6	27220	30104	dre-circ-2193	0	-	0	0	2	0	0
Zv9_NA7	12217	13713	dre-circ-2180	0	+	0	0	0	0	2
Zv9_NA7	364	28440	dre-circ-2194	0	+	0	0	0	0	3
Zv9_NA7	72448	97328	dre-circ-2196	0	-	0	0	0	0	11
Zv9_NA7	88164	97328	dre-circ-2197	0	-	0	0	0	0	2
Zv9_NA7	981	28794	dre-circ-2195	0	+	0	0	2	0	6
Zv9_NA8	37454	57376	dre-circ-4112	0	+	0	0	0	2	0
Zv9_NA8	87503	88115	dre-circ-2198	0	-	0	2	0	0	0
Zv9_NA8	31845	34124	dre-circ-2199	0	-	0	0	2	0	0
Zv9_NA8	20148	38185	dre-circ-2200	0	+	0	0	0	0	4
Zv9_NA9	4370	22871	dre-circ-4113	0	-	4	0	2	0	8
Zv9_NA9	36062	60974	dre-circ-4114	0	-	2	0	0	0	0
Zv9_NA9	39827	41403	dre-circ-2201	0	-	2	0	0	0	0
Zv9_NA9	84969	85224	dre-circ-2202	0	-	3	0	0	0	0
Zv9_NA9	2675	2788	dre-circ-2181	0	-	0	0	0	0	7
Zv9_NA9	157490	160863	dre-circ-4115	0	+	3	0	0	0	2
Zv9_NA9	73558	77246	dre-circ-4116	0	-	4	0	0	0	0
Zv9_NA9	129964	145545	dre-circ-2203	0	+	0	0	0	4	0
Zv9_scaf	151516	155713	dre-circ-4117	0	+	0	0	0	0	5
Zv9_scaf	154895	155713	dre-circ-2204	0	+	0	0	0	0	2
Zv9_scaf	270211	313032	dre-circ-2205	0	+	0	0	0	0	3
Zv9_scaf	61378	71914	dre-circ-4118	0	+	2	0	0	0	0
Zv9_scaf	73336	73641	dre-circ-4119	0	+	0	0	0	0	8
Zv9_scaf	105372	108364	dre-circ-4120	0	+	2	0	0	0	0
Zv9_scaf	21030	57201	dre-circ-4121	0	-	0	0	0	0	3
Zv9_scaf	91725	95344	dre-circ-4122	0	-	0	2	0	0	0
Zv9_scaf	12316	18137	dre-circ-2206	0	+	0	2	0	0	0
Zv9_scaf	165026	166329	dre-circ-4123	0	-	0	0	2	0	0
Zv9_scaf	15110	17650	dre-circ-4124	0	-	0	0	0	3	0
Zv9_scaf	27008	34918	dre-circ-4125	0	+	0	0	3	0	0

Supplementary table 4: List of polyA candidates identified

chr1:1480139-1568975:+
chr1:33426-61370:+
chr1:47619113-47631867:-
chr1:47619113-47642910:-
chr1:47632246-47642910:-
chr1:47632246-47657992:-
chr1:50033248-50055550:-
chr1:54677663-54746137:-
chr1:56564782-56659991:-
chr1:56582364-56634740:-
chr1:56777739-56821524:+
chr1:56790589-56834610:+
chr1:56797017-56850176:+
chr1:56807772-56859648:+
chr1:56810666-56864787:+
chr1:59699162-59701702:-
chr1:59819776-59863015:+
chr1:59832576-59850607:-
chr1:59871841-59875860:-
chr1:59871841-59879675:-
chr1:59871841-59887299:-
chr10:1511575-1547359:-
chr10:17493207-17538094:-
chr10:17499755-17540512:-
chr10:17503840-17541436:-
chr10:17510901-17545965:-
chr10:22291653-22298616:-
chr10:27175773-27226560:+
chr10:34218827-34289021:-
chr10:4007205-4012121:+
chr10:66258-114706:-
chr10:7088222-7107301:-
chr10:7779515-7779722:-
chr11:135097-138219:-
chr11:269278-282197:-
chr11:31505956-31508538:-
chr11:3369197-3452850:+
chr11:44248476-44318739:+
chr11:44248479-44318739:+
chr12:18774037-18778978:-
chr12:27394093-27434137:+
chr12:27400875-27445532:+
chr12:32246572-32246683:-
chr12:3798720-3887907:-
chr12:40514871-40569657:+
chr12:48086539-48151709:+
chr12:4822220-4848263:+
chr12:4823911-4848455:+
chr12:48264442-48283741:-
chr12:48861334-48884128:-
chr12:49947050-50044956:+
chr12:50392442-50480742:-
chr13:23126134-23165178:-
chr13:23313513-23394161:+
chr13:36377369-36385838:-
chr13:42795045-42809332:+
chr13:4369508-4369640:+
chr13:48158056-48166139:+
chr14:1085225-1160870:-
chr14:18594043-18630709:-
chr14:18605282-18643694:-
chr14:19091677-19131409:+
chr14:19091677-19157983:+
chr14:19231894-19331306:+
chr15:30333166-30357048:-
chr15:30338742-30360228:-
chr15:34408893-34418732:-
chr15:42790763-42800702:-
chr15:44795589-44801220:+
chr15:45660889-45672972:-
chr17:40002067-40007738:+
chr17:49734201-49762996:+
chr17:49743387-49765986:+
chr17:53975108-53977670:+
chr18:18438233-18477068:-
chr18:18438233-18525323:-
chr18:18442209-18481463:-
chr18:18442209-18506586:-
chr18:18442209-18528945:-
chr18:21896425-21904854:+
chr18:300787-355125:-
chr18:4531599-4618096:+
chr18:47878628-47879031:-
chr18:49470011-49514441:-
chr18:49600213-49603285:+
chr18:7015386-7023764:+
chr18:7034729-7034838:-
chr19:28034662-28072856:-
chr19:361078-362942:+
chr19:41148137-41157000:-
chr19:41151290-41157195:-
chr19:41151505-41158547:-
chr19:41152424-41158820:-
chr19:45288519-45291824:+
chr19:45288785-45294179:+
chr19:45289169-45294550:+
chr19:45289623-45297490:+
chr19:45482166-45498223:+
chr19:49852758-49858637:-
chr19:49853396-49858841:-
chr19:49855246-49859993:-
chr19:49992766-49998286:+
chr19:7663934-7690342:-
chr19:7665717-7692951:-
chr2:116708-117898:+
chr2:23576948-23595552:+
chr2:23577796-23596711:+
chr2:23579344-23598112:+
chr2:23582806-23599524:+
chr2:23583905-23600638:+
chr2:23586234-23602793:+
chr2:23587174-23603803:+
chr2:23588246-23605696:+
chr2:37178924-37179206:-
chr2:51638703-51649033:+
chr2:58106225-58113632:+
chr20:1424221-1435975:+
chr20:1427331-1439414:+
chr20:14898466-14954172:+
chr20:2332275-2340826:-
chr20:33996874-33998983:-
chr20:52813213-52833858:+
chr20:53228506-53261320:-
chr20:54325341-54338813:-
chr20:54326114-54340161:-
chr20:54329597-54342889:-
chr20:54564648-54568913:+
chr20:55029268-55067316:+
chr21:2964458-2968677:+
chr21:40675958-40683073:+
chr21:40675958-40683756:+
chr21:432172-490598:-
chr22:11867265-11885824:+
chr22:11868047-11886838:+
chr22:11868290-11888680:+
chr22:11868888-11889256:+
chr22:17691954-17700708:-
chr22:25203955-25284104:-
chr22:25204405-25224267:-
chr22:25204405-25257196:-
chr22:25257269-25284568:-
chr22:283754-329364:+
chr22:284093-303854:+
chr22:40743244-40765549:-
chr23:10282389-10358168:-
chr23:1420418-1468691:-
chr23:16980629-16987978:+
chr23:17082722-17118836:+
chr23:18101378-18117152:+
chr23:18117700-18161195:+
chr23:20502137-20586502:-
chr23:26406719-26415932:-
chr23:44738763-44742631:+
chr23:45088780-45088957:+
chr24:17571376-17576848:-
chr24:21521538-21529936:+
chr24:42267639-42299805:-
chr24:42267639-42315922:-
chr24:42272679-42303989:-
chr24:42272679-42352252:-
chr24:42278699-42353774:-
chr24:43528322-43542737:+
chr24:43855660-43855897:-
chr25:12749230-12762644:+
chr25:12751474-12763383:+
chr25:1880-28256:+
chr25:18939166-18950931:-
chr25:32118764-32127832:+
chr25:948311-1036714:+
chr3:32237116-32262532:+
chr3:32237393-32242839:+
chr3:32237393-32249811:+
chr3:32237393-32256630:+
chr3:32237393-32262864:+
chr3:32237393-32267404:+
chr3:32237393-32272760:+
chr3:32237393-32277395:+
chr3:39178861-39187314:-
chr3:55950217-55955205:-
chr3:55952075-55956952:+
chr3:60965081-60975293:-
chr3:722605-751760:-
chr4:1407774-1468103:+
chr4:1421184-1472751:+
chr4:59352600-59397220:-
chr5:2027389-2046940:+
chr5:24928122-24928482:-
chr5:33861954-33885370:+
chr5:33863530-33886945:+
chr5:33864866-33888253:+
chr5:33866045-33889439:+
chr5:33868011-33891489:+
chr5:33870192-33893662:+
chr5:33870913-33894362:+
chr5:43451576-43458170:-
chr5:67505698-67520725:-
chr5:67507927-67523993:-
chr5:67513661-67526980:-
chr5:67645373-67647180:-
chr5:8761074-8842843:-
chr6:47437136-47437310:-
chr6:59661847-59662466:+
chr6:59674610-59693920:+
chr6:6891326-6907472:-
chr7:14465140-14515259:-
chr7:16533007-16550235:+
chr7:40785314-40798560:+
chr7:40847081-40847156:+
chr7:54897209-54980703:+
chr7:56226867-56260980:+

chr15:46211723-46213349:-	chr22:25204405-25271501:-	chr7:65356229-65357283:-
chr15:46232413-46295234:-	chr22:25204405-25284568:-	chr7:74403326-74403661:-
chr16:15739525-15758394:+	chr22:25204693-25284848:-	chr7:76712553-76716324:-
chr16:28253731-28258469:+	chr22:25205738-25225237:-	chr7:8772445-8803314:-
chr16:28725765-28726011:+	chr22:25205738-25285550:-	chr8:19783650-19786181:-
chr16:33747496-33750239:-	chr22:25208159-25274334:-	chr8:21628586-21638178:-
chr16:34502191-34502665:+	chr22:25208159-25287915:-	chr8:23060952-23080537:+
chr16:49738320-49753709:+	chr22:25210248-25263410:-	chr9:39465161-39473565:+
chr16:54490096-54519866:+	chr22:25224788-25257653:-	chr9:42311495-42336667:+
chr16:54876931-54892688:+	chr22:25226277-25272864:-	chr9:58110796-58179977:-
chr16:57883301-57884355:-	chr22:25226277-25286480:-	chr9:786633-790254:-
chr17:2355450-2372577:-	chr22:25229483-25289660:-	Zv9_NA169:731-2873:+
Zv9_NA735:72447-97328:-	Zv9_NA900:4369-22871:-	Zv9_NA327:5782-8164:+

Supplementary table 5: Alignment detail and circRNA identified from CIRI pipeline

Tissue Name	Total reads	Mapped reads using BWA	Number of circular RNA identified
Blood	20461146	98.46%	833
Brain	16700545	99.57%	207
Muscle	20362229	99.58%	329
Gills	22524306	97.16%	530
Heart	25765610	98.06%	677

Supplementary table 6: List of circRNA junctions identified using CIRI

circChr	circStart	circStop	CircID	Score	circStrand	Blood	Brain	Heart	Muscle	Gills
chr10	11140240	11149265	dre-circ-2904	0	-	2	0	0	0	0
chr10	15004126	15004979	dre-circ-2905	0	+	0	0	0	0	3
chr10	1511576	1547359	dre-circ-2906	0	-	11	0	0	0	13
chr10	15540461	15553852	dre-circ-2907	0	+	6	0	0	0	0
chr10	16045927	16046265	dre-circ-2908	0	-	2	0	0	0	0
chr10	17494831	17495163	dre-circ-2471	0	-	3	0	0	0	0
chr10	17506357	17544065	dre-circ-2909	0	-	4	0	0	0	0
chr10	1800922	1802120	dre-circ-2910	0	-	0	0	2	0	0
chr10	20121530	20122876	dre-circ-2911	0	+	7	0	0	0	2
chr10	20168681	20170333	dre-circ-2912	0	+	10	0	0	0	0
chr10	20171698	20178455	dre-circ-2913	0	+	2	0	0	0	0
chr10	20544006	20545856	dre-circ-2472	0	-	0	2	0	0	0
chr10	208563	208867	dre-circ-2914	0	+	14	0	0	0	0
chr10	22024642	22030670	dre-circ-2915	0	+	0	2	0	0	0
chr10	22129671	22143333	dre-circ-2473	0	-	0	2	0	0	0
chr10	22718160	22718553	dre-circ-2916	0	+	4	0	0	0	0
chr10	22943543	22943852	dre-circ-2917	0	+	3	0	0	0	0
chr10	23773037	23778381	dre-circ-2918	0	+	0	0	0	2	0
chr10	24899994	24902502	dre-circ-2919	0	+	0	0	0	6	0
chr10	26429665	26430759	dre-circ-2920	0	-	0	0	2	0	0
chr10	27175545	27175966	dre-circ-2474	0	+	2	0	0	0	0
chr10	27532026	27537085	dre-circ-2475	0	+	0	0	2	0	0
chr10	28771365	28793190	dre-circ-2476	0	-	0	0	0	0	3
chr10	28915385	28943514	dre-circ-2477	0	-	0	3	0	0	0
chr10	28915385	28958636	dre-circ-2478	0	-	2	0	0	0	0
chr10	30168786	30169502	dre-circ-2479	0	+	2	0	0	0	0
chr10	32043776	32044422	dre-circ-2921	0	-	0	0	0	3	0
chr10	33418247	33420270	dre-circ-2480	0	-	2	0	0	0	0
chr10	3388335	3389332	dre-circ-2922	0	-	5	0	0	0	0
chr10	34249073	34319328	dre-circ-2481	0	-	2	0	0	0	5
chr10	34492837	34494944	dre-circ-2923	0	+	0	0	0	3	0
chr10	36468170	36475625	dre-circ-2482	0	+	0	0	2	0	0
chr10	37702591	37703683	dre-circ-2483	0	+	2	0	0	0	0
chr10	38716170	38725814	dre-circ-2924	0	+	0	0	5	0	0
chr10	39602143	39602496	dre-circ-2925	0	-	2	0	0	0	0
chr10	39968089	39977651	dre-circ-2484	0	+	0	0	2	0	0
chr10	40443666	40449241	dre-circ-2485	0	+	0	0	3	0	0
chr10	42279408	42293468	dre-circ-2486	0	-	0	0	0	0	2
chr10	42478785	42482305	dre-circ-2926	0	-	3	0	3	3	0
chr10	433087	435786	dre-circ-2927	0	+	0	0	2	0	0
chr10	44253001	44260384	dre-circ-2928	0	-	0	0	0	0	5
chr10	45861227	45879431	dre-circ-2929	0	+	3	0	0	0	0
chr10	46039070	46039291	dre-circ-2930	0	-	7	0	0	0	0
chr10	46137701	46142882	dre-circ-2487	0	+	5	0	0	0	0
chr10	46154263	46156675	dre-circ-2931	0	-	4	0	0	0	0
chr10	46507852	46508573	dre-circ-2932	0	-	3	0	0	0	0
chr10	5084232	5093886	dre-circ-2469	0	+	0	0	0	0	3
chr10	55389	56737	dre-circ-2468	0	+	2	0	0	0	0
chr10	5969087	5984626	dre-circ-2933	0	-	0	0	2	0	0
chr10	66259	114706	dre-circ-2934	0	-	2	2	4	4	7
chr10	6885588	6886001	dre-circ-2935	0	-	0	3	0	0	0
chr10	7075204	7081728	dre-circ-2470	0	-	0	0	0	0	7
chr10	7297129	7312041	dre-circ-2936	0	-	0	0	0	2	0
chr10	8372116	8376059	dre-circ-2937	0	+	2	0	0	0	0
chr10	8900131	8903278	dre-circ-2938	0	+	0	0	0	2	0
chr10	8948531	8953249	dre-circ-2939	0	+	0	0	0	0	4
chr10	9042427	9043140	dre-circ-2940	0	-	3	0	0	0	0
chr10	9127856	9139334	dre-circ-2941	0	-	2	0	0	0	0
chr1	10844764	10845317	dre-circ-2942	0	+	3	0	0	0	0
chr1	11049377	11049681	dre-circ-2943	0	-	2	0	2	0	0
chr11	11330969	11331357	dre-circ-2944	0	+	0	0	6	0	0
chr11	12304769	12305366	dre-circ-2945	0	+	2	2	0	0	0
chr11	135098	138219	dre-circ-2946	0	-	2	0	2	6	0
chr11	13679027	13680194	dre-circ-2947	0	+	0	0	0	0	2
chr11	14941989	14942566	dre-circ-2948	0	+	25	0	10	0	0
chr1	11541954	11543990	dre-circ-2949	0	+	0	0	3	3	0
chr11	1682960	1683498	dre-circ-2490	0	+	0	0	0	0	2
chr11	1739229	1740100	dre-circ-2950	0	+	0	2	0	0	3
chr11	18554328	18677446	dre-circ-2496	0	+	0	0	0	0	4
chr11	19496680	19505053	dre-circ-2951	0	+	2	0	0	0	3
chr11	19582349	19588480	dre-circ-2497	0	+	0	2	0	0	0
chr11	19929153	19930019	dre-circ-2498	0	+	2	0	0	0	0
chr11	2073425	2076064	dre-circ-2952	0	-	0	0	0	3	0
chr11	212360	214342	dre-circ-2489	0	-	2	0	0	0	0
chr11	21991125	21993134	dre-circ-2953	0	-	2	0	0	0	0
chr11	23128534	23128859	dre-circ-2954	0	-	0	0	2	0	0
chr11	2427215	2432085	dre-circ-2955	0	-	0	0	10	0	0
chr11	24299391	24309605	dre-circ-2956	0	-	0	0	0	0	2
chr11	24356088	24356888	dre-circ-2957	0	-	0	0	3	0	0
chr11	2487474	2491431	dre-circ-2958	0	+	3	0	0	0	0
chr11	25139	25567	dre-circ-2488	0	+	2	0	0	0	0
chr11	25312865	25314090	dre-circ-2959	0	-	0	2	0	0	0
chr11	26119848	26120513	dre-circ-2960	0	-	0	0	2	0	0

chr11	26220213	26220774	dre-circ-2961	0	+	0	0	0	0	2
chr11	26412113	26415339	dre-circ-2499	0	+	2	0	0	0	0
chr11	26628964	26633075	dre-circ-2962	0	+	0	2	0	0	0
chr11	2787671	2788788	dre-circ-2963	0	+	2	0	0	0	0
chr11	3050209	3066374	dre-circ-2491	0	+	0	0	0	0	3
chr11	30637282	30641506	dre-circ-2964	0	-	0	0	3	0	3
chr11	31312549	31320425	dre-circ-2965	0	-	4	0	37	0	2
chr11	31420528	31425688	dre-circ-2966	0	-	0	0	6	0	0
chr11	31420528	31438547	dre-circ-2967	0	-	0	0	72	0	0
chr11	31425561	31438547	dre-circ-2968	0	-	0	0	48	0	0
chr11	31475039	31476893	dre-circ-2969	0	-	0	0	2	0	0
chr11	3229030	3229238	dre-circ-2492	0	+	0	0	0	0	3
chr11	3295186	3297777	dre-circ-2493	0	+	2	0	0	0	0
chr1	13359476	13361152	dre-circ-2970	0	+	0	0	0	2	0
chr11	3388137	3388760	dre-circ-2971	0	+	0	3	0	0	0
chr11	37206138	37208602	dre-circ-2972	0	+	3	0	0	0	5
chr11	37383067	37395830	dre-circ-2500	0	+	0	0	0	0	2
chr11	38024412	38188508	dre-circ-2501	0	+	0	0	0	0	10
chr11	38066971	38230719	dre-circ-2502	0	-	0	2	0	0	0
chr11	38124313	38124625	dre-circ-2973	0	+	5	0	0	3	0
chr11	38421098	38421333	dre-circ-2503	0	-	3	0	0	0	0
chr11	40324162	40326672	dre-circ-2974	0	-	2	2	2	11	0
chr11	40368695	40372716	dre-circ-2975	0	+	0	0	0	0	2
chr11	41543463	41548285	dre-circ-2976	0	+	0	0	3	0	0
chr11	41543463	41548290	dre-circ-2977	0	+	0	0	9	0	3
chr1	14159342	14186413	dre-circ-2214	0	-	0	0	0	0	2
chr11	41629200	41630307	dre-circ-2978	0	+	0	0	2	0	0
chr11	41629203	41637174	dre-circ-2979	0	+	0	0	0	2	0
chr11	41631193	41644435	dre-circ-2504	0	+	2	0	0	0	0
chr11	41639197	41644435	dre-circ-2980	0	+	0	0	5	5	0
chr1	14185460	14186413	dre-circ-2981	0	-	2	0	0	2	0
chr11	41917042	41917358	dre-circ-2982	0	-	0	0	2	0	0
chr11	42055259	42055703	dre-circ-2983	0	+	0	0	3	0	0
chr11	42369286	42499688	dre-circ-2505	0	-	0	5	0	0	0
chr11	43669861	43671901	dre-circ-2984	0	+	0	0	0	3	0
chr11	44248477	44318739	dre-circ-2985	0	+	6	41	54	22	0
chr11	44248480	44318739	dre-circ-2986	0	+	0	8	14	4	3
chr11	44622744	44634495	dre-circ-2987	0	-	6	0	2	0	0
chr11	44624723	44634495	dre-circ-2988	0	-	2	0	0	0	0
chr11	44629761	44634495	dre-circ-2989	0	-	4	0	0	0	0
chr11	45183108	45186531	dre-circ-2506	0	-	0	2	0	0	0
chr1	1456054	1565139	dre-circ-2208	0	+	2	0	0	0	6
chr1	1456449	1550424	dre-circ-2209	0	+	0	0	0	0	5
chr11	45689821	45872299	dre-circ-2507	0	-	6	0	0	0	0
chr11	45756165	45942975	dre-circ-2508	0	-	0	0	0	2	0
chr11	46055933	46058686	dre-circ-2990	0	-	2	0	0	0	0
chr11	46461667	46572998	dre-circ-2509	0	-	16	8	11	7	11
chr1	1478788	1602386	dre-circ-2210	0	+	6	2	3	0	0
chr1	1479892	1551748	dre-circ-2991	0	+	2	0	0	0	0
chr1	1550116	1565139	dre-circ-2211	0	+	0	0	0	0	4
chr11	5579535	5580813	dre-circ-2992	0	+	4	0	0	0	0
chr1	1568094	1602122	dre-circ-2993	0	+	0	0	0	0	3
chr1	1569054	1603092	dre-circ-2994	0	+	5	0	0	0	0
chr11	5892218	5893901	dre-circ-2995	0	-	3	0	0	0	0
chr1	16501794	16512429	dre-circ-2996	0	-	0	0	4	0	0
chr1	1654583	1661319	dre-circ-2997	0	+	0	0	0	0	5
chr11	6697133	6698238	dre-circ-2494	0	+	0	2	0	0	0
chr11	7020394	7022932	dre-circ-2495	0	-	0	3	0	0	0
chr11	7534717	7539338	dre-circ-2998	0	-	0	0	0	0	2
chr11	9049625	9129009	dre-circ-2999	0	-	0	2	2	0	0
chr1	19652203	19655654	dre-circ-3000	0	-	0	0	0	0	2
chr12	1246351	1247401	dre-circ-3001	0	-	2	0	0	0	0
chr12	15050914	15051334	dre-circ-3002	0	+	0	3	2	0	7
chr12	15166306	15166496	dre-circ-2517	0	-	3	0	0	0	0
chr12	15193434	15197664	dre-circ-3003	0	+	3	0	0	0	0
chr12	16514288	16516418	dre-circ-3004	0	+	6	0	0	0	0
chr12	16514288	16516876	dre-circ-3005	0	+	2	0	0	0	0
chr12	16514288	16522808	dre-circ-3006	0	+	6	0	0	0	0
chr12	16520616	16522808	dre-circ-2518	0	+	2	0	0	0	0
chr12	17082412	17083066	dre-circ-2519	0	-	2	0	0	2	5
chr12	18240160	18240666	dre-circ-3007	0	-	0	0	2	0	0
chr1	2186341	2190351	dre-circ-3008	0	+	0	0	0	0	3
chr12	1867718	1905611	dre-circ-3009	0	+	6	0	2	0	0
chr12	18774038	18778978	dre-circ-3010	0	-	0	0	0	12	0
chr12	20072076	20072427	dre-circ-3011	0	+	2	0	0	0	0
chr12	20244945	20245742	dre-circ-3012	0	-	3	0	0	0	0
chr12	20540365	20543362	dre-circ-3013	0	+	3	0	0	0	0
chr12	206966	210971	dre-circ-3014	0	+	0	0	3	0	0
chr12	24219633	24220013	dre-circ-3015	0	+	0	0	2	0	0
chr12	25315405	25347872	dre-circ-3016	0	+	3	0	0	0	0
chr12	25555979	25556367	dre-circ-3017	0	+	23	0	3	0	0
chr12	27602316	27605294	dre-circ-2520	0	-	0	0	2	0	0
chr12	28646270	28647111	dre-circ-3018	0	+	2	0	0	0	0
chr12	289587	290255	dre-circ-3019	0	-	7	0	4	0	0
chr12	29470183	29477587	dre-circ-2521	0	+	0	0	3	0	0
chr12	29600011	29613461	dre-circ-3020	0	+	0	0	2	0	0
chr12	29600011	29620128	dre-circ-3021	0	+	0	0	3	0	0

chr12	29632146	29632592	dre-circ-3022	0	+	0	0	2	0	0
chr12	29881440	29888311	dre-circ-3023	0	-	0	0	0	0	5
chr12	30425956	30426595	dre-circ-3024	0	-	0	2	0	0	0
chr12	31738441	31738827	dre-circ-3025	0	+	0	0	3	0	0
chr12	31762912	31772040	dre-circ-2522	0	+	0	0	7	0	0
chr12	31768207	31768365	dre-circ-2523	0	+	0	2	0	0	0
chr12	33101304	33102004	dre-circ-2524	0	-	2	0	0	0	0
chr12	35729358	35729547	dre-circ-3026	0	-	11	0	2	0	0
chr12	36463861	36464982	dre-circ-3027	0	-	4	0	2	0	0
chr12	37323575	37326559	dre-circ-3028	0	-	0	0	0	0	2
chr12	37513023	37517972	dre-circ-3029	0	-	3	0	4	3	3
chr12	3798721	3887907	dre-circ-3030	0	-	0	0	2	0	0
chr12	38353300	38353542	dre-circ-3031	0	+	0	0	3	0	4
chr12	39704487	39707059	dre-circ-2525	0	-	0	0	0	0	2
chr12	40369593	40371334	dre-circ-3032	0	+	9	0	0	0	0
chr12	40431954	40507405	dre-circ-2526	0	+	0	0	0	0	3
chr12	40441315	40507405	dre-circ-3033	0	+	0	0	0	0	2
chr12	40442006	40500782	dre-circ-2527	0	+	6	0	0	0	0
chr12	40450892	40567596	dre-circ-2528	0	+	0	0	2	0	2
chr12	40455154	40570357	dre-circ-2529	0	+	0	0	0	0	2
chr12	40476466	40524894	dre-circ-3034	0	+	0	0	0	0	79
chr12	4183598	4189439	dre-circ-3035	0	-	0	0	2	0	0
chr12	4205648	4205821	dre-circ-3036	0	-	2	0	0	0	0
chr12	4246352	4247947	dre-circ-3037	0	+	2	0	0	0	0
chr12	43382577	43386696	dre-circ-3038	0	-	6	4	5	0	3
chr12	43389952	43403869	dre-circ-3039	0	-	3	0	0	0	0
chr12	45246871	45251774	dre-circ-3040	0	-	0	0	0	2	0
chr12	46285571	46288558	dre-circ-2530	0	-	0	0	0	0	28
chr12	46617105	46617607	dre-circ-3041	0	-	2	0	0	0	0
chr12	47026363	47083820	dre-circ-2531	0	-	0	0	0	0	2
chr12	47027714	47145747	dre-circ-2532	0	-	0	0	0	0	4
chr12	47147682	47330239	dre-circ-2533	0	-	5	0	0	0	12
chr12	47148883	47155021	dre-circ-2534	0	-	0	0	0	0	3
chr12	47227846	47390553	dre-circ-2535	0	-	0	0	0	0	8
chr1	24743837	24746774	dre-circ-3042	0	-	0	0	2	5	0
chr12	4758866	4766728	dre-circ-2515	0	+	0	0	0	0	2
chr12	48016566	48021113	dre-circ-3043	0	+	5	0	0	0	0
chr12	48016566	48032291	dre-circ-3044	0	+	5	0	0	0	0
chr12	48016566	48038865	dre-circ-3045	0	+	3	0	0	0	0
chr12	48016566	48041392	dre-circ-2536	0	+	3	0	0	0	0
chr12	48031633	48032291	dre-circ-2537	0	+	3	0	0	0	0
chr12	48079388	48145621	dre-circ-2538	0	+	0	0	0	0	2
chr12	4813742	4837133	dre-circ-3046	0	+	0	2	0	0	0
chr12	48165645	48166134	dre-circ-3047	0	+	2	0	0	0	0
chr12	4818167	4842114	dre-circ-3048	0	+	0	0	0	5	0
chr12	4822221	4848263	dre-circ-3049	0	+	2	0	0	0	2
chr12	49373268	49379974	dre-circ-3050	0	-	0	0	0	0	2
chr12	4993342	5007059	dre-circ-3051	0	-	0	0	0	0	4
chr12	4994042	5007247	dre-circ-3052	0	-	2	0	0	0	0
chr12	49947051	49947327	dre-circ-3053	0	+	3	0	0	4	0
chr12	50189993	50190882	dre-circ-3054	0	-	2	0	0	0	0
chr12	50329861	50439268	dre-circ-2539	0	-	2	0	2	0	0
chr12	50392443	50480742	dre-circ-3055	0	-	0	0	0	5	0
chr12	5266247	5267071	dre-circ-3056	0	-	2	0	0	0	0
chr12	5269282	5288998	dre-circ-3057	0	-	2	0	0	0	0
chr1	25356181	25443152	dre-circ-2215	0	+	0	0	3	4	9
chr12	548447	550632	dre-circ-2510	0	+	0	0	3	0	0
chr12	5700699	5701293	dre-circ-2516	0	+	2	0	0	0	0
chr12	595850	741812	dre-circ-2511	0	-	2	0	0	0	0
chr12	600939	747851	dre-circ-2512	0	-	4	0	3	0	5
chr1	26079630	26080266	dre-circ-2216	0	+	0	0	0	0	2
chr12	616787	763120	dre-circ-2513	0	-	0	0	3	4	10
chr12	6226966	6227335	dre-circ-3058	0	-	0	0	0	0	2
chr1	26794590	26795743	dre-circ-3059	0	+	0	0	2	0	0
chr1	27205361	27223825	dre-circ-3060	0	+	0	0	0	0	2
chr1	27482660	27488891	dre-circ-3061	0	-	0	0	2	0	0
chr1	27487902	27488891	dre-circ-3062	0	-	3	3	4	0	0
chr1	27702913	27740044	dre-circ-2217	0	-	0	3	0	0	0
chr1	27718742	27740044	dre-circ-3063	0	-	0	0	2	0	0
chr1	27737431	27740044	dre-circ-3064	0	-	3	10	24	10	6
chr1	27737431	27741348	dre-circ-3065	0	-	0	0	3	0	0
chr1	27815742	27817194	dre-circ-2218	0	-	0	0	2	0	0
chr12	8361730	8383177	dre-circ-3066	0	-	0	0	2	0	0
chr12	8372233	8383177	dre-circ-3067	0	-	0	0	3	0	0
chr12	9339752	9345640	dre-circ-3068	0	-	0	0	0	0	2
chr12	941294	945837	dre-circ-3069	0	+	4	0	0	0	2
chr12	943575	947995	dre-circ-2514	0	+	2	0	0	0	0
chr1	296439	296829	dre-circ-3070	0	+	2	0	0	0	0
chr12	9852088	9853358	dre-circ-3071	0	-	5	0	0	0	0
chr13	10655931	10656389	dre-circ-3072	0	+	13	0	0	0	2
chr13	11481730	11490650	dre-circ-3073	0	-	0	0	2	0	0
chr13	1266763	1267536	dre-circ-3074	0	-	0	0	2	0	0
chr13	12801388	12815428	dre-circ-3075	0	+	0	0	0	0	2
chr13	1286658	1288659	dre-circ-3076	0	-	0	0	4	2	0
chr13	1413823	1415178	dre-circ-3077	0	+	7	55	34	30	0
chr13	15572999	15574407	dre-circ-3078	0	+	0	0	3	0	0
chr13	15666877	15679436	dre-circ-3079	0	+	0	0	2	0	2

chr13	15717329	15717780	dre-circ-3080	0	-	3	0	0	0	0
chr13	15792126	15796829	dre-circ-3081	0	+	0	0	0	0	2
chr13	15919625	15920020	dre-circ-3082	0	-	6	0	0	0	2
chr13	17771664	17772395	dre-circ-2543	0	-	6	0	2	0	0
chr13	18216516	18216877	dre-circ-3083	0	-	2	0	0	0	0
chr13	18323730	18326125	dre-circ-3084	0	+	3	2	0	2	0
chr13	18443681	18446466	dre-circ-3085	0	+	3	0	0	0	0
chr13	19047723	19065984	dre-circ-2544	0	+	0	0	2	0	0
chr13	1959785	1963000	dre-circ-2540	0	-	0	0	3	2	0
chr13	1976046	1978932	dre-circ-3086	0	-	0	0	2	0	0
chr13	2053630	2063005	dre-circ-2541	0	-	0	0	2	0	3
chr13	2104006	2108727	dre-circ-3087	0	-	0	0	0	0	3
chr13	22986378	22997597	dre-circ-2545	0	-	0	0	0	2	0
chr13	2334605	2339941	dre-circ-3088	0	+	3	0	6	0	10
chr13	23432682	23439871	dre-circ-3089	0	+	4	0	0	0	0
chr13	24164555	24167418	dre-circ-3090	0	-	0	0	0	6	0
chr13	25462483	25466539	dre-circ-3091	0	-	0	0	0	0	2
chr13	26060248	26063400	dre-circ-3092	0	-	0	0	0	2	0
chr13	26777967	26781508	dre-circ-3093	0	-	0	0	0	0	3
chr13	28278728	28400315	dre-circ-2546	0	-	4	0	0	0	0
chr13	28959027	28966433	dre-circ-2547	0	+	2	0	0	0	0
chr13	2963936	2964420	dre-circ-3094	0	-	2	0	0	0	0
chr13	30262500	30262874	dre-circ-3095	0	+	3	0	0	0	0
chr13	30398863	30404792	dre-circ-3096	0	+	0	2	0	0	0
chr13	30452672	30456866	dre-circ-2548	0	+	2	0	0	0	0
chr13	33647250	33649273	dre-circ-3097	0	+	2	0	6	2	0
chr13	34689974	34752826	dre-circ-3098	0	+	0	2	0	0	0
chr13	34737093	34752826	dre-circ-2549	0	+	0	0	2	0	0
chr13	34949881	34956878	dre-circ-3099	0	+	2	0	2	0	0
chr13	34949881	34968819	dre-circ-3100	0	+	0	0	2	0	0
chr13	3580648	3582605	dre-circ-3101	0	-	2	0	0	0	0
chr13	37566804	37572058	dre-circ-3102	0	-	0	0	2	0	0
chr13	37626432	37628618	dre-circ-3103	0	-	0	0	4	0	0
chr13	4227586	4247615	dre-circ-3104	0	+	0	2	0	0	0
chr13	43031484	43031686	dre-circ-3105	0	-	2	0	0	0	0
chr13	43179260	43181486	dre-circ-3106	0	-	12	0	6	0	3
chr13	43446474	43514179	dre-circ-3107	0	+	0	0	0	0	2
chr13	43705263	43741929	dre-circ-2550	0	+	0	0	0	2	0
chr13	4383628	4383987	dre-circ-3108	0	-	3	0	0	0	0
chr13	44142465	44149544	dre-circ-2551	0	-	0	5	0	0	0
chr13	45332086	45335650	dre-circ-3109	0	-	12	0	0	0	0
chr13	45462362	45463221	dre-circ-2552	0	-	0	0	6	0	13
chr13	45656004	45663674	dre-circ-3110	0	-	2	0	0	0	0
chr13	46124368	46130632	dre-circ-3111	0	-	43	0	4	0	0
chr13	46135107	46135257	dre-circ-3112	0	-	7	0	0	0	0
chr13	46754263	46754740	dre-circ-3113	0	-	4	0	0	0	0
chr13	48158057	48166139	dre-circ-3114	0	+	14	9	9	0	28
chr13	49836970	49979207	dre-circ-2553	0	+	0	0	0	0	3
chr13	49836970	49991606	dre-circ-2554	0	+	0	0	3	0	0
chr13	50059604	50059844	dre-circ-2555	0	-	0	0	0	0	2
chr13	50143520	50145528	dre-circ-3115	0	-	3	0	0	0	3
chr13	50601275	50603450	dre-circ-2556	0	-	0	0	2	0	0
chr13	51205914	51207794	dre-circ-3116	0	-	7	0	0	0	0
chr13	51509156	51515761	dre-circ-2557	0	-	0	2	0	2	0
chr13	52015866	52018269	dre-circ-3117	0	+	0	0	2	0	0
chr13	52169253	52174800	dre-circ-3118	0	+	0	0	3	4	0
chr13	52183864	52189017	dre-circ-2558	0	+	0	0	3	4	0
chr13	52193222	52197559	dre-circ-3119	0	+	9	24	20	12	8
chr1	35290920	35295668	dre-circ-3120	0	+	0	0	4	0	0
chr13	53649055	53655278	dre-circ-3121	0	+	3	0	0	0	0
chr13	53653346	53653778	dre-circ-3122	0	+	11	0	0	0	2
chr1	354482	354695	dre-circ-3123	0	-	8	0	0	0	0
chr13	5570882	5574611	dre-circ-3124	0	-	0	0	0	2	0
chr13	5636173	5636763	dre-circ-2542	0	-	3	0	0	0	0
chr1	35967384	35970300	dre-circ-2219	0	+	0	0	3	0	0
chr1	37491220	37520018	dre-circ-2220	0	-	0	0	0	0	5
chr13	7619202	7619479	dre-circ-3125	0	+	3	0	0	0	5
chr1	38131018	38289546	dre-circ-2221	0	+	0	0	0	0	3
chr1	40708359	40708786	dre-circ-3126	0	+	5	0	0	0	21
chr1	40802877	40804460	dre-circ-3127	0	-	3	0	0	0	2
chr14	1012460	1015698	dre-circ-3128	0	+	0	0	3	0	0
chr14	1030714	1031735	dre-circ-3129	0	+	0	0	42	0	0
chr14	1120232	1127996	dre-circ-3130	0	+	3	0	0	0	0
chr14	1154317	1170247	dre-circ-3131	0	-	0	2	0	4	0
chr14	13722474	13724167	dre-circ-3132	0	+	3	0	0	3	5
chr1	41611277	41611918	dre-circ-3133	0	-	0	0	0	0	11
chr14	19395444	19497322	dre-circ-2564	0	+	0	0	8	0	0
chr14	20025440	20026865	dre-circ-3134	0	-	0	0	2	0	0
chr14	20098053	20098577	dre-circ-3135	0	+	0	0	0	4	0
chr14	21956169	21974711	dre-circ-3136	0	+	2	0	0	0	0
chr14	21956169	21979654	dre-circ-3137	0	+	4	0	0	0	0
chr14	24561629	24562187	dre-circ-3138	0	+	4	0	0	0	0
chr14	29371840	29386165	dre-circ-3139	0	+	0	0	0	0	2
chr14	30655650	30658797	dre-circ-3140	0	-	0	0	2	0	0
chr14	30690760	30696933	dre-circ-2565	0	-	0	0	5	0	0
chr14	3069185	3069404	dre-circ-3141	0	-	52	0	0	0	0
chr14	31299687	31302961	dre-circ-3142	0	-	0	2	0	0	0

chr14	31758670	31761352	dre-circ-2566	0	-	0	0	0	0	2
chr14	33738602	33739765	dre-circ-2567	0	+	0	0	2	0	0
chr14	34892801	34897172	dre-circ-3143	0	-	3	0	0	0	0
chr14	35633131	35642266	dre-circ-2568	0	-	0	0	2	0	0
chr14	3585077	3723775	dre-circ-2559	0	+	0	6	0	0	0
chr14	38870486	38870862	dre-circ-3144	0	+	0	5	0	0	0
chr14	41643224	41648310	dre-circ-3145	0	-	0	3	0	0	0
chr14	4270057	4276095	dre-circ-3146	0	-	2	0	0	0	0
chr14	42831156	42888534	dre-circ-3147	0	+	0	0	0	0	5
chr14	4283259	4291791	dre-circ-2560	0	-	2	0	0	0	0
chr1	44411666	44411973	dre-circ-2222	0	+	6	0	0	6	5
chr14	47005872	47006375	dre-circ-3148	0	-	0	0	3	2	0
chr1	44705091	44707597	dre-circ-3149	0	+	20	0	0	0	0
chr14	47748243	47748537	dre-circ-3150	0	-	6	0	0	0	0
chr14	47994943	48009814	dre-circ-3151	0	+	0	0	0	0	3
chr14	49160546	49209905	dre-circ-3152	0	+	0	0	0	0	2
chr14	49571966	49584194	dre-circ-2569	0	-	2	0	0	0	0
chr14	51645249	51646434	dre-circ-3153	0	+	10	0	0	3	4
chr14	53234046	53234306	dre-circ-2570	0	+	0	2	0	0	0
chr1	45331768	45350377	dre-circ-3154	0	+	0	0	2	0	0
chr14	53473753	53474830	dre-circ-3155	0	-	15	0	4	3	0
chr14	5625095	5634787	dre-circ-2561	0	-	0	0	2	0	0
chr1	45705443	45707015	dre-circ-3156	0	+	0	10	0	0	0
chr14	5860940	5861458	dre-circ-3157	0	+	2	0	0	0	0
chr1	46092679	46111430	dre-circ-2223	0	-	0	0	2	0	0
chr1	46101877	46111430	dre-circ-3158	0	-	0	0	11	0	0
chr1	46155652	46160692	dre-circ-3159	0	+	3	0	0	0	0
chr14	6257546	6258312	dre-circ-3160	0	-	3	2	4	0	0
chr14	6425997	6428537	dre-circ-3161	0	+	0	0	0	0	2
chr14	6586155	6589767	dre-circ-3162	0	+	5	0	0	0	0
chr14	7147677	7150285	dre-circ-2562	0	-	0	0	2	0	0
chr1	4752008	4752191	dre-circ-3163	0	-	3	0	0	0	3
chr1	48139867	48164676	dre-circ-2224	0	+	0	0	6	0	0
chr14	8578606	8579227	dre-circ-3164	0	+	3	0	0	0	0
chr14	8593554	8593930	dre-circ-3165	0	+	4	0	0	0	0
chr1	48968710	49100620	dre-circ-2225	0	+	0	2	0	0	0
chr14	9133815	9139986	dre-circ-2563	0	-	2	0	0	0	0
chr1	49926983	49927146	dre-circ-2226	0	+	2	0	0	0	0
chr1	49971256	49973637	dre-circ-2227	0	+	2	0	0	0	0
chr1	50036046	50061919	dre-circ-2228	0	-	0	0	3	0	0
chr1	5010139	5010513	dre-circ-3166	0	+	0	0	3	0	0
chr1	5013514	5018702	dre-circ-3167	0	+	4	0	18	5	5
chr1	50589833	50592790	dre-circ-2229	0	+	0	0	0	2	0
chr1	5066893	5070720	dre-circ-3168	0	+	0	2	0	0	0
chr15	1002387	1019200	dre-circ-2575	0	-	4	0	0	0	0
chr1	51158825	51159210	dre-circ-3169	0	+	2	0	0	0	0
chr15	12934748	12946163	dre-circ-2581	0	-	0	0	4	0	0
chr1	51356831	51382324	dre-circ-2230	0	+	0	0	0	0	2
chr15	13633323	13633829	dre-circ-3170	0	+	31	0	0	0	0
chr15	1514987	1517911	dre-circ-2576	0	-	2	0	0	0	0
chr15	15229938	15230325	dre-circ-3171	0	-	0	0	2	0	0
chr15	16206248	16216480	dre-circ-2582	0	-	0	0	0	0	3
chr15	16221090	16229746	dre-circ-3172	0	-	0	0	3	0	0
chr15	1656294	1658972	dre-circ-3173	0	+	0	0	2	0	0
chr15	16901121	16903905	dre-circ-3174	0	-	0	0	2	0	0
chr15	16923501	16930225	dre-circ-3175	0	-	0	0	7	0	0
chr15	16926864	16930225	dre-circ-3176	0	-	0	0	3	0	0
chr15	18211952	18214030	dre-circ-2583	0	+	0	2	2	0	0
chr1	51841979	51849108	dre-circ-3177	0	+	0	4	0	0	0
chr15	19868000	19870356	dre-circ-3178	0	-	0	0	2	0	0
chr15	20266872	20267589	dre-circ-2584	0	-	0	0	0	0	2
chr15	20496977	20507688	dre-circ-2585	0	-	0	0	0	0	4
chr15	20785305	20791571	dre-circ-3179	0	-	3	0	0	0	0
chr15	22597300	22597736	dre-circ-3180	0	+	5	0	0	0	0
chr15	22972361	22975221	dre-circ-3181	0	+	0	0	2	0	0
chr15	23196770	23229665	dre-circ-3182	0	-	2	0	0	0	0
chr15	2392181	2403233	dre-circ-3183	0	+	0	0	4	0	0
chr1	52454487	52455018	dre-circ-2231	0	+	3	0	0	0	0
chr15	24799151	24800185	dre-circ-3184	0	+	2	0	0	0	0
chr15	2525803	2528127	dre-circ-2577	0	+	2	0	0	0	0
chr1	52543032	52550796	dre-circ-3185	0	+	4	0	6	0	0
chr15	28458692	28459253	dre-circ-3186	0	-	3	0	0	0	0
chr15	2912411	2912918	dre-circ-3187	0	-	0	0	0	0	2
chr15	29633325	29655374	dre-circ-3188	0	+	3	0	0	0	0
chr15	29637991	29655374	dre-circ-3189	0	+	4	0	0	0	0
chr15	29675650	29677803	dre-circ-3190	0	+	2	0	0	0	0
chr1	53086613	53087458	dre-circ-3191	0	+	11	0	6	2	0
chr15	31086673	31086999	dre-circ-2586	0	+	0	0	0	0	2
chr15	31522558	31525556	dre-circ-2587	0	+	2	0	0	0	0
chr1	53182808	53183055	dre-circ-3192	0	+	2	0	0	0	0
chr15	3676724	3677152	dre-circ-3193	0	+	3	0	0	0	0
chr15	37013944	37021556	dre-circ-3194	0	+	0	0	0	0	2
chr15	37943996	37976254	dre-circ-2588	0	-	2	0	0	0	0
chr15	38726333	38730489	dre-circ-3195	0	+	2	0	0	0	0
chr15	4011866	4013630	dre-circ-3196	0	-	0	0	0	2	0
chr15	40238392	40284001	dre-circ-2589	0	-	4	0	3	0	0
chr15	40239648	40259563	dre-circ-3197	0	-	0	0	4	0	0

chr15	40244454	40273339	dre-circ-3198	0	-	3	0	3	0	0
chr15	40269594	40279569	dre-circ-2590	0	-	0	0	5	0	0
chr15	41459227	41462605	dre-circ-2591	0	-	0	0	0	0	2
chr1	54177551	54179856	dre-circ-3199	0	-	3	0	0	0	0
chr15	42490448	42490697	dre-circ-3200	0	-	0	2	0	0	0
chr15	42817867	42834507	dre-circ-2592	0	-	0	0	0	0	5
chr15	4286886	4291533	dre-circ-2578	0	-	2	0	0	0	0
chr15	43061461	43080344	dre-circ-3201	0	-	0	0	0	0	3
chr15	4325819	4331988	dre-circ-3202	0	-	9	0	0	0	0
chr15	4325819	4340622	dre-circ-3203	0	-	5	0	0	0	0
chr15	43856519	43865453	dre-circ-3204	0	+	0	0	2	0	0
chr15	44255211	44391064	dre-circ-2593	0	-	32	12	22	7	36
chr15	44370095	44559816	dre-circ-2594	0	-	15	2	5	6	32
chr15	44795590	44801220	dre-circ-3205	0	+	0	0	16	3	22
chr15	45736674	45738663	dre-circ-2595	0	-	0	0	0	0	8
chr15	458071	461842	dre-circ-3206	0	+	6	0	0	0	0
chr15	46232414	46295234	dre-circ-3207	0	-	0	2	0	0	0
chr15	46375614	46381458	dre-circ-3208	0	+	3	0	0	0	0
chr1	54677664	54746137	dre-circ-3209	0	-	22	2	7	4	29
chr15	513637	517125	dre-circ-2571	0	+	0	0	0	0	2
chr1	55319352	55425324	dre-circ-2232	0	+	0	0	7	0	0
chr15	5477985	5483212	dre-circ-3210	0	+	0	0	2	0	0
chr1	55734525	55738556	dre-circ-3211	0	-	2	0	0	0	0
chr1	55762368	55763878	dre-circ-2233	0	-	0	0	2	0	0
chr15	57997	69140	dre-circ-3212	0	-	0	0	0	3	0
chr15	5833285	5843660	dre-circ-2579	0	+	0	0	2	0	0
chr1	56061010	56083708	dre-circ-2234	0	+	0	0	0	0	3
chr1	56206024	56264511	dre-circ-2235	0	+	0	0	0	0	2
chr15	676144	736103	dre-circ-2572	0	-	5	0	2	0	2
chr1	56777740	56821524	dre-circ-3213	0	+	0	0	10	0	0
chr1	56787400	56830694	dre-circ-2236	0	+	0	0	3	0	0
chr1	56792880	56837841	dre-circ-3214	0	+	0	0	3	0	0
chr1	56793921	56838322	dre-circ-2237	0	+	0	0	5	0	0
chr1	56794344	56841016	dre-circ-2238	0	+	0	0	5	0	0
chr1	56803164	56854119	dre-circ-3215	0	+	0	0	0	14	0
chr1	56808336	56861578	dre-circ-2239	0	+	0	0	0	14	0
chr1	56810667	56864787	dre-circ-3216	0	+	0	0	5	0	0
chr15	737580	750033	dre-circ-2573	0	-	0	0	6	0	0
chr15	811983	833732	dre-circ-2574	0	+	0	0	0	0	4
chr1	58482940	58489148	dre-circ-3217	0	+	0	0	3	0	0
chr15	8791891	8800920	dre-circ-2580	0	-	0	0	2	0	0
chr1	58895499	59024268	dre-circ-2240	0	+	0	0	0	0	7
chr1	58908862	59040122	dre-circ-2241	0	+	2	0	0	0	0
chr1	58923167	59052601	dre-circ-2242	0	+	0	0	2	0	0
chr1	59167892	59185182	dre-circ-2243	0	-	0	0	0	0	8
chr1	59174934	59185182	dre-circ-2244	0	-	3	0	0	0	4
chr1	59448335	59452375	dre-circ-2245	0	+	2	0	0	0	0
chr1	59699163	59701702	dre-circ-3218	0	-	0	0	4	3	8
chr1	59699163	59704355	dre-circ-3219	0	-	5	0	0	0	0
chr1	59819984	59863143	dre-circ-2246	0	-	0	0	5	0	0
chr1	59849950	59886530	dre-circ-2247	0	-	0	0	7	0	0
chr1	59857235	59863143	dre-circ-2248	0	-	0	0	3	0	0
chr1	60166225	60169709	dre-circ-2249	0	+	0	0	0	0	27
chr1	60166444	60169863	dre-circ-2250	0	+	0	0	0	0	8
chr1	60295205	60297129	dre-circ-2251	0	+	3	0	0	0	0
chr1	605789	606422	dre-circ-2207	0	+	3	0	0	0	0
chr1	6091791	6280448	dre-circ-2212	0	-	18	2	32	4	5
chr16	10229738	10251842	dre-circ-3220	0	-	0	3	0	0	0
chr16	10393648	10397326	dre-circ-3221	0	+	0	0	0	0	2
chr16	10502040	10513846	dre-circ-3222	0	+	0	0	0	0	2
chr16	11159379	11292082	dre-circ-2605	0	-	0	0	16	0	0
chr16	1237128	1237793	dre-circ-2596	0	-	2	0	0	0	0
chr16	1290862	1293650	dre-circ-3223	0	+	2	0	2	0	0
chr16	1330814	1335433	dre-circ-3224	0	+	2	6	12	15	0
chr16	15891196	15899109	dre-circ-3225	0	-	0	0	8	0	0
chr16	15893366	15899109	dre-circ-3226	0	-	0	3	3	0	0
chr16	15918978	15923748	dre-circ-3227	0	-	0	0	2	0	0
chr16	1698202	1700252	dre-circ-2597	0	+	0	0	0	0	2
chr16	1892189	1897504	dre-circ-2598	0	+	0	0	0	0	86
chr16	1892189	1900927	dre-circ-2599	0	+	0	0	0	0	2
chr16	1893449	1903956	dre-circ-3228	0	+	3	0	0	0	0
chr16	1896599	1899923	dre-circ-2600	0	+	0	0	0	0	23
chr16	19325904	19343460	dre-circ-3229	0	-	2	0	0	0	0
chr16	19326145	19343460	dre-circ-2606	0	-	2	0	0	0	0
chr16	19332746	19343460	dre-circ-3230	0	-	2	0	0	0	0
chr16	19333129	19343460	dre-circ-3231	0	-	2	0	0	0	0
chr16	19714960	19719098	dre-circ-3232	0	-	15	3	10	0	10
chr16	19752255	19752812	dre-circ-2607	0	-	0	0	3	0	2
chr16	19752255	19757593	dre-circ-2608	0	-	0	0	4	0	0
chr16	20498197	20498376	dre-circ-2609	0	+	3	0	0	0	0
chr16	2100904	2257041	dre-circ-2601	0	+	7	0	0	0	44
chr16	2117093	2270976	dre-circ-2602	0	+	0	0	0	0	2
chr16	24545961	24551656	dre-circ-3233	0	-	0	0	3	0	0
chr16	24979223	24984740	dre-circ-3234	0	-	9	0	0	4	0
chr16	27019509	27034979	dre-circ-3235	0	-	3	0	0	0	0
chr16	28706309	28710390	dre-circ-3236	0	+	3	0	0	0	0
chr16	28719287	28719484	dre-circ-3237	0	+	14	0	0	0	0

chr16	28812973	28822282	dre-circ-3238	0	+	0	0	8	0	2
chr16	28825358	28825663	dre-circ-3239	0	+	3	0	0	0	0
chr16	29383957	29385999	dre-circ-3240	0	-	0	0	0	3	0
chr16	30505537	30508332	dre-circ-2610	0	+	0	0	0	2	0
chr16	30620935	30634695	dre-circ-3241	0	+	0	0	3	0	0
chr16	31335352	31349121	dre-circ-3242	0	-	4	0	10	0	0
chr16	3174933	3175168	dre-circ-2603	0	+	3	0	0	0	0
chr16	31770309	31774821	dre-circ-2611	0	+	5	0	4	0	0
chr16	3178553	3182379	dre-circ-3243	0	+	2	0	2	0	0
chr16	33111878	33112648	dre-circ-3244	0	-	3	0	2	3	0
chr16	33116368	33118782	dre-circ-2612	0	-	0	2	0	0	0
chr16	33588864	33589621	dre-circ-3245	0	-	0	2	0	0	0
chr16	34125672	34127980	dre-circ-3246	0	-	0	0	2	0	0
chr16	3429512	3429957	dre-circ-3247	0	+	9	0	5	0	0
chr16	35416442	35416787	dre-circ-3248	0	-	2	0	0	0	0
chr16	36388117	36391193	dre-circ-3249	0	+	2	0	0	0	0
chr16	38164688	38165216	dre-circ-3250	0	+	2	0	0	0	0
chr16	38164688	38165410	dre-circ-2613	0	+	0	2	0	0	0
chr16	39235834	39382663	dre-circ-2614	0	-	4	0	0	0	0
chr16	39237434	39382831	dre-circ-2615	0	-	0	3	0	0	0
chr16	39248458	39394847	dre-circ-2616	0	-	0	3	0	0	0
chr16	39555399	39555633	dre-circ-3251	0	-	2	0	0	0	0
chr16	39601901	39606269	dre-circ-3252	0	+	6	0	0	2	0
chr16	39692788	39693403	dre-circ-2617	0	+	0	0	0	0	2
chr16	3976960	3977142	dre-circ-3253	0	+	0	0	2	0	0
chr16	4016991	4021988	dre-circ-3254	0	+	4	0	3	2	0
chr16	40978432	40985639	dre-circ-3255	0	-	2	3	90	8	8
chr16	42332914	42351822	dre-circ-3256	0	-	0	0	3	0	0
chr16	42767138	42767820	dre-circ-2618	0	+	0	0	2	0	0
chr16	43695116	43719608	dre-circ-2619	0	+	3	0	0	0	0
chr16	44470092	44470277	dre-circ-3257	0	-	0	0	0	2	0
chr16	44528984	44532443	dre-circ-3258	0	-	3	0	4	0	3
chr16	44618537	44626486	dre-circ-2620	0	+	6	0	0	0	0
chr16	44691041	44691510	dre-circ-3259	0	-	2	0	0	0	0
chr16	46360562	46365194	dre-circ-3260	0	-	9	0	0	0	0
chr16	47215342	47242417	dre-circ-3261	0	-	0	5	0	0	0
chr16	47391925	47423250	dre-circ-3262	0	-	0	0	0	0	5
chr16	47518273	47518559	dre-circ-3263	0	+	16	0	3	0	0
chr1	6477442	6490284	dre-circ-2213	0	+	0	0	2	0	0
chr16	47768328	47769224	dre-circ-3264	0	-	0	0	0	0	4
chr16	47774809	47775152	dre-circ-3265	0	-	0	0	0	0	15
chr16	48829858	48839628	dre-circ-3266	0	+	0	0	0	0	2
chr16	50614138	50619036	dre-circ-3267	0	-	5	0	3	0	3
chr16	52020456	52039146	dre-circ-3268	0	+	0	0	0	0	2
chr16	5546851	5548778	dre-circ-2604	0	+	0	0	2	0	0
chr16	5560087	5573153	dre-circ-3269	0	-	0	0	0	3	0
chr16	55964658	55967490	dre-circ-3270	0	+	0	0	0	0	3
chr16	56209460	56218181	dre-circ-3271	0	-	3	0	0	0	0
chr16	56432614	56441840	dre-circ-3272	0	-	0	0	0	3	2
chr16	56436247	56441840	dre-circ-3273	0	-	0	0	0	0	3
chr16	56478234	56479267	dre-circ-2621	0	-	2	0	3	0	0
chr16	56769815	56770421	dre-circ-3274	0	+	0	0	0	0	2
chr16	57127251	57127533	dre-circ-3275	0	+	15	0	3	0	0
chr16	57929668	57930724	dre-circ-2622	0	-	2	0	0	0	0
chr16	7438840	7444681	dre-circ-3276	0	-	0	0	0	5	0
chr16	8116905	8120714	dre-circ-3277	0	+	0	0	0	0	3
chr16	8232058	8284671	dre-circ-3278	0	+	5	0	0	0	0
chr16	9025066	9027548	dre-circ-3279	0	-	0	0	2	0	0
chr17	10190606	10196131	dre-circ-2628	0	+	2	0	0	0	0
chr17	1136605	1152121	dre-circ-3280	0	+	2	0	0	0	0
chr17	1145578	1148493	dre-circ-3281	0	+	2	0	0	0	0
chr17	11555728	11555990	dre-circ-3282	0	+	0	0	6	4	0
chr17	12886341	12886851	dre-circ-3283	0	-	5	0	0	0	3
chr17	12954893	12955144	dre-circ-3284	0	+	12	4	4	6	0
chr17	145524	150594	dre-circ-3285	0	-	2	0	0	0	0
chr17	14735072	14735549	dre-circ-2629	0	+	0	0	0	0	2
chr17	15299719	15301175	dre-circ-2630	0	-	0	0	0	2	0
chr17	15308122	15309288	dre-circ-2631	0	-	0	0	0	22	0
chr17	15308122	15311754	dre-circ-2632	0	-	0	0	0	0	4
chr17	15308350	15309288	dre-circ-2633	0	-	0	0	0	2	0
chr17	15309583	15311754	dre-circ-2634	0	-	0	0	2	0	0
chr17	15309747	15310107	dre-circ-2635	0	-	0	0	0	2	0
chr17	15318655	15318917	dre-circ-2636	0	-	0	0	0	5	0
chr17	15644356	15651393	dre-circ-3286	0	+	0	0	2	0	0
chr17	16044487	16075344	dre-circ-2637	0	+	0	0	0	0	2
chr17	16463612	16464168	dre-circ-3287	0	+	2	0	3	0	2
chr17	17884097	17885559	dre-circ-3288	0	+	30	2	9	6	0
chr17	17915184	17919738	dre-circ-3289	0	+	5	0	0	0	0
chr17	19890304	19891111	dre-circ-3290	0	-	0	0	351	0	0
chr17	19911129	19912376	dre-circ-3291	0	-	0	0	4	0	0
chr17	20534663	20539531	dre-circ-2638	0	-	0	3	0	0	0
chr17	20546607	20564417	dre-circ-3292	0	-	0	0	3	0	0
chr17	2270102	2284306	dre-circ-3293	0	-	0	0	3	0	0
chr17	2276447	2284306	dre-circ-3294	0	-	0	10	8	5	0
chr17	2280349	2284306	dre-circ-3295	0	-	0	0	2	2	0
chr17	2348187	2358785	dre-circ-2623	0	-	0	0	0	0	2
chr17	2353097	2358939	dre-circ-2624	0	-	0	0	0	0	53

chr17	24043238	24051120	dre-circ-2639	0	+	0	0	5	0	0
chr17	25105645	25106727	dre-circ-3296	0	-	0	0	2	0	0
chr17	28108591	28111713	dre-circ-2640	0	+	0	0	2	0	0
chr17	28781285	28784511	dre-circ-3297	0	+	5	0	6	0	2
chr17	28781285	28790031	dre-circ-3298	0	+	4	3	8	3	2
chr17	28852069	28853093	dre-circ-3299	0	-	8	13	19	16	8
chr17	30537912	30538804	dre-circ-3300	0	-	3	0	3	0	0
chr17	30562242	30564667	dre-circ-3301	0	-	2	0	0	0	0
chr17	30843419	30857119	dre-circ-2641	0	+	0	0	2	0	0
chr17	31435542	31448396	dre-circ-3302	0	+	0	0	0	0	2
chr17	32652304	32652649	dre-circ-2642	0	+	9	0	0	0	0
chr17	35375187	35375440	dre-circ-3303	0	+	0	2	8	0	0
chr17	37351200	37358291	dre-circ-3304	0	-	3	0	0	0	0
chr17	40002068	40007738	dre-circ-3305	0	+	17	5	0	0	0
chr17	40013502	40014832	dre-circ-2643	0	+	12	0	0	0	0
chr17	4098906	4099222	dre-circ-3306	0	+	16	0	0	0	0
chr17	41042269	41043332	dre-circ-3307	0	+	3	2	2	0	2
chr17	4175391	4208837	dre-circ-2625	0	+	2	0	0	0	2
chr17	41801318	41803794	dre-circ-3308	0	-	3	0	0	0	0
chr17	4242464	4245829	dre-circ-3309	0	+	0	0	0	0	5
chr17	44013086	44013447	dre-circ-2644	0	+	7	0	0	0	0
chr17	44013086	44015823	dre-circ-3310	0	+	3	0	2	0	0
chr17	44015156	44015823	dre-circ-2645	0	+	3	0	0	0	2
chr17	44914120	44915511	dre-circ-3311	0	+	0	0	5	0	0
chr17	45430159	45432468	dre-circ-2646	0	+	0	0	3	0	0
chr17	45549785	45550392	dre-circ-3312	0	-	23	2	9	2	3
chr17	49082463	49085000	dre-circ-3313	0	-	0	0	2	6	0
chr17	50726939	50731619	dre-circ-3314	0	-	0	0	6	0	0
chr17	50798139	50798300	dre-circ-2647	0	+	6	0	0	0	0
chr17	50966138	51014380	dre-circ-2648	0	-	6	0	0	0	7
chr17	50984902	51002406	dre-circ-2649	0	-	2	0	0	0	0
chr17	51949620	51951087	dre-circ-2650	0	-	0	4	0	0	0
chr17	5222713	5227962	dre-circ-3315	0	+	0	0	0	2	0
chr17	53148665	53153176	dre-circ-2651	0	+	0	0	0	0	4
chr17	53347199	53447368	dre-circ-2652	0	+	0	10	0	5	6
chr17	53347202	53447368	dre-circ-2653	0	+	0	3	0	0	0
chr17	53808095	53809775	dre-circ-2654	0	-	0	0	2	0	0
chr17	6339490	6340874	dre-circ-3316	0	+	4	0	0	0	3
chr17	6440831	6441792	dre-circ-3317	0	+	2	0	4	0	0
chr17	7358670	7359155	dre-circ-2626	0	-	7	0	0	0	0
chr17	7359031	7368996	dre-circ-2627	0	-	7	0	0	0	0
chr1	7777574	7778068	dre-circ-3318	0	+	8	0	0	0	0
chr17	7937362	7945236	dre-circ-3319	0	-	0	0	0	2	0
chr17	8065756	8066468	dre-circ-3320	0	+	3	0	0	0	0
chr17	8194337	8207115	dre-circ-3321	0	+	4	0	0	0	2
chr17	8594505	8594882	dre-circ-3322	0	-	2	0	0	0	0
chr17	957037	961856	dre-circ-3323	0	+	2	0	0	0	0
chr1	7983134	7992924	dre-circ-3324	0	-	0	0	0	0	2
chr18	10281304	10282090	dre-circ-3325	0	+	0	0	0	2	0
chr18	10320182	10325624	dre-circ-3326	0	+	7	0	3	0	0
chr18	14155586	14155912	dre-circ-3327	0	+	0	0	0	4	0
chr18	14284358	14286889	dre-circ-3328	0	+	4	2	4	2	0
chr18	15794940	15797317	dre-circ-3329	0	+	2	0	0	0	0
chr18	16707203	16710688	dre-circ-3330	0	-	22	0	0	0	0
chr18	17105442	17108612	dre-circ-2657	0	+	0	0	3	0	0
chr18	17335187	17338070	dre-circ-3331	0	+	0	0	0	3	0
chr18	18226176	18349260	dre-circ-2658	0	-	4	0	3	0	0
chr18	20472483	20472674	dre-circ-3332	0	+	0	0	0	2	0
chr18	20651844	20652348	dre-circ-3333	0	+	0	0	0	7	0
chr18	20651844	20655924	dre-circ-3334	0	+	0	0	0	3	0
chr18	22349560	22357986	dre-circ-2659	0	+	0	0	2	0	0
chr18	22832508	22832819	dre-circ-3335	0	+	8	0	0	0	0
chr18	23030068	23030283	dre-circ-2660	0	+	4	0	0	0	3
chr18	25380206	25385792	dre-circ-3336	0	-	4	0	3	0	0
chr18	26680090	26682538	dre-circ-3337	0	+	0	0	23	0	0
chr18	27931188	27933114	dre-circ-3338	0	+	0	0	4	0	0
chr18	300788	355125	dre-circ-3339	0	-	0	0	10	7	82
chr18	31039547	31045181	dre-circ-3340	0	+	2	0	0	0	0
chr18	31040529	31045181	dre-circ-3341	0	+	5	0	2	0	0
chr18	31063652	31078170	dre-circ-2661	0	+	0	0	2	0	0
chr18	33241140	33245276	dre-circ-3342	0	+	2	0	0	0	0
chr18	33860803	33864609	dre-circ-2662	0	-	0	0	0	3	0
chr18	35398525	35399045	dre-circ-2663	0	+	0	0	2	0	3
chr18	35649600	35653017	dre-circ-3343	0	-	2	0	2	0	2
chr18	36275898	36285500	dre-circ-3344	0	-	0	2	0	0	0
chr18	36303289	36316518	dre-circ-3345	0	-	0	2	0	0	0
chr18	37219698	37220050	dre-circ-2664	0	+	4	0	0	0	0
chr18	37780181	37782190	dre-circ-3346	0	+	2	0	3	0	0
chr18	38520860	38522993	dre-circ-2665	0	+	0	2	0	0	0
chr18	38852106	38922225	dre-circ-2666	0	+	0	0	0	8	0
chr18	38872195	38922225	dre-circ-2667	0	+	0	0	0	4	0
chr18	38927062	38950498	dre-circ-2668	0	+	0	0	0	2	0
chr18	38932677	38954242	dre-circ-2669	0	+	2	0	0	0	0
chr18	39646077	39646914	dre-circ-3347	0	+	3	0	0	0	0
chr18	39860552	39864703	dre-circ-3348	0	+	0	0	0	0	2
chr18	42984845	42985335	dre-circ-3349	0	+	8	0	0	0	0
chr18	4531600	4618096	dre-circ-3350	0	+	0	4	2	3	3

chr18	47010208	47012743	dre-circ-3351	0	+	0	0	3	0	0
chr18	47072026	47092568	dre-circ-2670	0	-	0	0	0	0	4
chr18	47449809	47450219	dre-circ-3352	0	-	3	0	0	0	0
chr18	47463906	47468820	dre-circ-3353	0	-	16	0	2	0	0
chr18	47878629	47879031	dre-circ-3354	0	-	0	0	2	0	0
chr18	47899284	47906977	dre-circ-3355	0	-	0	0	2	0	0
chr18	49133028	49133768	dre-circ-2671	0	+	0	2	0	0	0
chr18	49470012	49514441	dre-circ-3356	0	-	5	0	0	0	18
chr18	49539771	49540310	dre-circ-3357	0	-	2	0	0	0	0
chr18	49590482	49595796	dre-circ-2672	0	+	5	0	5	0	0
chr18	49592266	49597727	dre-circ-2673	0	+	3	0	0	0	0
chr18	5361612	5361945	dre-circ-3358	0	+	0	0	2	0	0
chr1	8588328	8588815	dre-circ-3359	0	-	5	0	2	0	0
chr18	5913052	5918274	dre-circ-2655	0	-	0	0	2	0	0
chr18	6780203	6782687	dre-circ-3360	0	+	0	0	3	0	0
chr18	7012238	7019214	dre-circ-3361	0	+	0	0	2	3	6
chr18	7012499	7023345	dre-circ-3362	0	+	0	0	3	3	0
chr18	7014469	7023514	dre-circ-2656	0	+	0	0	0	0	3
chr18	8436276	8447916	dre-circ-3363	0	-	0	3	0	0	0
chr19	1041668	1080992	dre-circ-2676	0	+	0	0	2	0	0
chr19	1050079	1050972	dre-circ-2677	0	-	4	0	0	0	2
chr19	10904413	10908764	dre-circ-2689	0	-	4	0	0	0	0
chr19	11187155	11190982	dre-circ-3364	0	+	3	0	0	0	2
chr19	11319765	11324995	dre-circ-3365	0	-	0	0	0	2	0
chr19	11490020	11501042	dre-circ-2690	0	-	0	0	0	0	5
chr19	11849228	11849649	dre-circ-3366	0	+	0	0	0	0	3
chr19	1497503	1498222	dre-circ-3367	0	-	0	0	0	2	0
chr19	18130967	18170930	dre-circ-3368	0	-	0	0	2	0	0
chr19	19532917	19552577	dre-circ-3369	0	-	0	2	4	2	0
chr19	19532917	19603157	dre-circ-2691	0	-	0	0	3	0	0
chr19	19677053	19691547	dre-circ-2692	0	-	0	0	3	0	0
chr19	19692509	19695154	dre-circ-3370	0	-	0	0	2	0	0
chr19	19733451	19741187	dre-circ-3371	0	-	0	0	2	0	0
chr19	19897264	19905483	dre-circ-3372	0	-	0	0	0	0	2
chr19	20526987	20527359	dre-circ-3373	0	+	3	0	0	0	0
chr19	2233014	2246371	dre-circ-3374	0	-	0	0	0	0	4
chr19	22946830	22959693	dre-circ-3375	0	-	0	4	14	4	0
chr19	23274599	23275198	dre-circ-3376	0	-	2	0	2	0	0
chr19	28008431	28008964	dre-circ-3377	0	-	0	0	0	0	5
chr19	28168715	28173230	dre-circ-3378	0	-	0	0	2	0	0
chr19	30836573	30841758	dre-circ-3379	0	+	2	0	0	0	0
chr19	3104574	3105842	dre-circ-3380	0	+	0	0	0	3	0
chr19	31118140	31121859	dre-circ-3381	0	-	0	0	4	0	0
chr19	32889607	32900661	dre-circ-3382	0	-	0	0	3	4	0
chr19	36708008	36710480	dre-circ-3383	0	-	4	0	0	0	0
chr19	36753642	36770300	dre-circ-3384	0	-	7	0	0	0	0
chr19	39060419	39060828	dre-circ-3385	0	+	10	0	2	0	0
chr19	39065656	39069860	dre-circ-3386	0	+	7	0	0	0	0
chr19	41049368	41053177	dre-circ-3387	0	+	0	4	2	0	0
chr19	41088676	41089826	dre-circ-3388	0	+	3	3	7	0	0
chr19	41088676	41097466	dre-circ-3389	0	+	2	0	0	0	0
chr19	41143189	41143977	dre-circ-3390	0	-	0	0	0	0	6
chr19	41863724	41863995	dre-circ-3391	0	-	2	3	0	0	0
chr19	43939253	43940947	dre-circ-3392	0	-	0	0	0	2	0
chr19	44158500	44165377	dre-circ-3393	0	-	0	0	0	0	2
chr19	44163627	44172450	dre-circ-2693	0	-	0	0	2	0	0
chr19	44165285	44173968	dre-circ-3394	0	-	0	0	3	0	0
chr19	44169266	44174695	dre-circ-3395	0	-	0	0	0	2	0
chr19	44657598	44659723	dre-circ-3396	0	+	2	0	0	0	0
chr19	45183326	45186894	dre-circ-3397	0	-	0	0	0	0	2
chr19	45288786	45294179	dre-circ-3398	0	+	0	36	0	0	0
chr19	45289958	45300421	dre-circ-2694	0	+	0	0	0	0	3
chr19	45319193	45320496	dre-circ-2695	0	+	2	0	0	0	0
chr19	47910449	47911560	dre-circ-3399	0	+	2	0	0	0	0
chr19	48077877	48090679	dre-circ-2696	0	-	2	0	0	0	0
chr19	4838400	4850034	dre-circ-2678	0	+	6	0	0	0	13
chr19	485608	487036	dre-circ-2674	0	-	8	0	3	0	0
chr19	4859917	4877487	dre-circ-2679	0	+	0	0	0	0	7
chr19	49199929	49208161	dre-circ-2697	0	+	7	0	0	2	0
chr19	49204530	49214333	dre-circ-3400	0	+	2	0	0	0	0
chr19	49852759	49858637	dre-circ-3401	0	-	51	0	39	339	12
chr19	49855247	49859993	dre-circ-3402	0	-	0	6	0	0	0
chr19	49992767	49998286	dre-circ-3403	0	+	3	0	0	0	15
chr19	5275533	5277421	dre-circ-3404	0	+	2	0	0	0	0
chr19	5528055	5529127	dre-circ-3405	0	+	0	0	0	2	0
chr19	5652318	5652706	dre-circ-2680	0	-	2	0	0	0	0
chr19	589624	590052	dre-circ-3406	0	-	4	0	0	0	0
chr19	5975218	6003825	dre-circ-2681	0	-	0	0	5	0	0
chr19	5981696	6003825	dre-circ-2682	0	-	0	0	0	13	0
chr19	5992232	6003348	dre-circ-3407	0	-	2	0	0	0	5
chr19	612583	615268	dre-circ-2675	0	+	0	0	2	0	0
chr19	7582165	7582823	dre-circ-2683	0	-	3	0	0	0	0
chr19	7602965	7605482	dre-circ-3408	0	-	4	0	2	0	0
chr19	7665718	7692951	dre-circ-3409	0	-	0	0	7	0	0
chr19	8060575	8061000	dre-circ-2684	0	-	0	0	0	0	2
chr19	8423330	8423562	dre-circ-3410	0	+	4	0	0	0	0
chr19	8471684	8533368	dre-circ-3411	0	+	6	0	0	0	7

chr19	8480931	8541869	dre-circ-2685	0	+	2	0	0	0	3
chr19	8483209	8507783	dre-circ-2686	0	+	0	0	0	0	2
chr19	8506599	8543188	dre-circ-2687	0	+	0	0	4	0	3
chr19	8508241	8545219	dre-circ-3412	0	+	7	0	0	0	5
chr19	8508573	8545434	dre-circ-3413	0	+	0	0	0	0	2
chr19	9499414	9500104	dre-circ-2688	0	-	2	0	0	0	0
chr19	9698032	9700100	dre-circ-3414	0	+	4	0	0	0	0
chr19	9742331	9746122	dre-circ-3415	0	+	0	0	0	0	2
chr20	12990786	12990992	dre-circ-3416	0	-	0	0	0	0	13
chr20	13182517	13186030	dre-circ-3417	0	+	0	0	3	0	0
chr20	15091590	15100493	dre-circ-3418	0	-	0	0	2	0	0
chr20	153719	154628	dre-circ-3419	0	-	3	0	0	0	0
chr20	18421623	18433347	dre-circ-2704	0	-	2	0	0	0	0
chr20	1985681	2005058	dre-circ-2699	0	+	0	0	0	3	0
chr20	21900017	21913509	dre-circ-3420	0	-	0	0	2	0	0
chr20	23320015	23324750	dre-circ-3421	0	+	10	0	0	0	0
chr20	2332276	2340826	dre-circ-3422	0	-	0	0	2	0	0
chr20	2332276	2341615	dre-circ-3423	0	-	3	0	0	0	2
chr20	23441054	23443998	dre-circ-3424	0	-	3	0	0	0	0
chr20	23612878	23615702	dre-circ-2705	0	+	0	0	2	0	0
chr20	24309910	24320982	dre-circ-3425	0	+	5	0	24	3	0
chr20	2453601	2461915	dre-circ-2700	0	+	2	0	0	0	0
chr20	2459372	2461915	dre-circ-3426	0	+	3	0	0	0	0
chr20	25602298	25621189	dre-circ-2706	0	+	0	0	0	0	2
chr20	25620763	25621463	dre-circ-3427	0	+	2	0	3	0	4
chr20	26226617	26229327	dre-circ-2707	0	+	0	2	0	0	0
chr20	2624400	2625271	dre-circ-3428	0	+	0	0	0	0	2
chr20	26998812	27000501	dre-circ-3429	0	+	0	0	8	5	0
chr20	26998812	27003399	dre-circ-3430	0	+	0	0	2	0	0
chr20	27350082	27351377	dre-circ-3431	0	-	0	0	0	2	0
chr20	27360365	27361919	dre-circ-3432	0	+	0	0	0	0	3
chr20	28313652	28314507	dre-circ-3433	0	-	4	0	0	0	0
chr20	2919303	2924446	dre-circ-3434	0	-	10	0	0	0	0
chr20	2932657	2933459	dre-circ-3435	0	-	4	0	0	0	0
chr20	3061427	3063193	dre-circ-3436	0	-	8	7	13	0	9
chr20	33171146	33171406	dre-circ-3437	0	-	0	0	5	5	0
chr20	33513383	33514730	dre-circ-3438	0	-	20	0	14	5	0
chr20	33594262	33618004	dre-circ-2708	0	-	0	0	0	0	13
chr20	33594262	33677831	dre-circ-2709	0	-	2	0	0	0	0
chr20	33997443	33999661	dre-circ-3439	0	-	0	0	38	0	0
chr20	35614929	35625500	dre-circ-2710	0	-	2	0	0	0	0
chr20	35615501	35622770	dre-circ-2711	0	-	0	0	0	0	35
chr20	35615501	35638212	dre-circ-2712	0	-	0	0	0	0	6
chr20	35615854	35626667	dre-circ-2713	0	-	2	0	0	0	0
chr20	35615854	35634649	dre-circ-2714	0	-	0	0	0	0	10
chr20	35615854	35636081	dre-circ-2715	0	-	2	0	0	0	0
chr20	35616153	35625216	dre-circ-2716	0	-	0	0	0	0	10
chr20	35616512	35637875	dre-circ-2717	0	-	2	0	0	0	0
chr20	35617169	35623056	dre-circ-2718	0	-	3	0	0	0	0
chr20	35617169	35626035	dre-circ-2719	0	-	0	0	0	0	3
chr20	35617169	35637875	dre-circ-2720	0	-	0	0	0	0	3
chr20	35617826	35626035	dre-circ-2721	0	-	2	0	0	0	2
chr20	35618504	35626035	dre-circ-2722	0	-	0	0	0	0	3
chr20	35622276	35625734	dre-circ-2723	0	-	0	0	0	0	2
chr20	35622624	35623056	dre-circ-2724	0	-	0	0	0	0	4
chr20	35625070	35626035	dre-circ-2725	0	-	2	0	0	0	0
chr20	35626235	35637875	dre-circ-2726	0	-	0	0	0	0	2
chr20	35628975	35640340	dre-circ-3440	0	-	3	0	0	0	0
chr20	35633844	35637301	dre-circ-2727	0	-	0	0	0	0	17
chr20	35642253	35644356	dre-circ-3441	0	-	2	0	0	0	0
chr20	35647200	35718714	dre-circ-2728	0	-	2	0	0	0	0
chr20	35648325	35706285	dre-circ-2729	0	-	2	0	0	0	12
chr20	35649868	35699779	dre-circ-2730	0	-	7	0	0	0	0
chr20	35650608	35711373	dre-circ-2731	0	-	0	0	0	0	4
chr20	35652163	35686020	dre-circ-2732	0	-	0	0	0	0	38
chr20	35652163	35690329	dre-circ-2733	0	-	0	0	0	0	6
chr20	35652163	35699779	dre-circ-2734	0	-	4	0	0	3	40
chr20	35653618	35720283	dre-circ-2735	0	-	3	0	0	0	0
chr20	35655863	35769818	dre-circ-2736	0	-	0	0	0	0	2
chr20	35659272	35669216	dre-circ-2737	0	-	2	0	0	0	0
chr20	35659272	35690329	dre-circ-2738	0	-	0	0	0	0	3
chr20	35660819	35696820	dre-circ-2739	0	-	0	0	0	0	2
chr20	35660819	35700764	dre-circ-2740	0	-	0	0	0	0	7
chr20	35660819	35706285	dre-circ-2741	0	-	0	0	0	0	4
chr20	35660819	35719838	dre-circ-2742	0	-	0	0	0	0	4
chr20	35660819	35722868	dre-circ-2743	0	-	0	0	0	0	5
chr20	35662276	35699779	dre-circ-2744	0	-	0	0	0	2	0
chr20	35667936	35670267	dre-circ-2745	0	-	0	0	0	0	2
chr20	35667936	35693950	dre-circ-2746	0	-	0	0	0	0	4
chr20	35667936	35696820	dre-circ-2747	0	-	0	0	0	0	2
chr20	35670121	35711373	dre-circ-2748	0	-	0	0	0	0	4
chr20	35672739	35711373	dre-circ-2749	0	-	0	0	0	0	2
chr20	35675712	35692964	dre-circ-2750	0	-	0	0	0	0	12
chr20	35675712	35699779	dre-circ-2751	0	-	0	0	2	0	0
chr20	35679701	35699779	dre-circ-2752	0	-	0	0	0	0	3
chr20	35689646	35720283	dre-circ-2753	0	-	0	0	0	0	2
chr20	35690508	35718714	dre-circ-2754	0	-	0	0	0	0	2

chr20	35691962	35700764	dre-circ-2755	0	-	0	0	0	0	5
chr20	35691962	35706285	dre-circ-2756	0	-	0	0	0	0	5
chr20	35691962	35708691	dre-circ-2757	0	-	0	0	0	0	16
chr20	35691962	35711744	dre-circ-2758	0	-	0	0	0	0	5
chr20	35691962	35719838	dre-circ-2759	0	-	0	0	0	0	3
chr20	35694510	35722868	dre-circ-2760	0	-	0	0	0	0	3
chr20	35694830	35723235	dre-circ-2761	0	-	7	0	0	0	0
chr20	35705881	35769818	dre-circ-2762	0	-	0	0	0	0	2
chr20	38506676	38520688	dre-circ-2763	0	+	2	0	0	0	0
chr20	38904695	38908778	dre-circ-2764	0	+	10	0	0	0	0
chr20	40199648	40225320	dre-circ-2765	0	+	0	0	10	0	0
chr20	40233063	40256783	dre-circ-2766	0	+	0	0	153	0	0
chr20	42354572	42373010	dre-circ-3442	0	-	0	0	0	0	2
chr20	43228156	43240878	dre-circ-3443	0	-	0	0	4	0	0
chr20	433727	434128	dre-circ-3444	0	-	9	0	0	0	0
chr20	4339467	4341546	dre-circ-3445	0	+	0	0	0	0	3
chr20	44810415	44810894	dre-circ-2767	0	+	2	0	0	0	0
chr20	44810415	44812707	dre-circ-3446	0	+	2	0	0	0	0
chr20	457173	457443	dre-circ-3447	0	-	26	0	4	0	2
chr20	45881617	45888602	dre-circ-3448	0	-	0	0	5	0	0
chr20	47185706	47186539	dre-circ-3449	0	+	0	0	0	9	0
chr20	47260606	47262602	dre-circ-2768	0	-	0	0	2	0	0
chr20	47274103	47289005	dre-circ-2769	0	-	0	0	3	0	0
chr20	48636907	48639238	dre-circ-2770	0	+	0	0	2	0	0
chr20	50334159	50342735	dre-circ-3450	0	+	3	0	7	0	0
chr20	51681665	51686502	dre-circ-3451	0	-	2	0	0	0	0
chr20	51711746	51714567	dre-circ-3452	0	-	15	3	4	0	0
chr20	52055896	52060562	dre-circ-2771	0	-	0	0	0	0	3
chr20	52195349	52198312	dre-circ-2772	0	+	2	0	0	0	0
chr20	5282704	5299177	dre-circ-2701	0	-	0	0	2	0	0
chr20	52884363	52884989	dre-circ-2773	0	-	0	0	0	0	3
chr20	53088400	53091497	dre-circ-2774	0	+	0	0	0	2	0
chr20	5317910	5318583	dre-circ-2702	0	-	3	0	0	0	0
chr20	53228507	53261320	dre-circ-3453	0	-	18	0	26	5	7
chr20	53289706	53292230	dre-circ-3454	0	-	4	0	0	0	0
chr20	53324896	53327144	dre-circ-2775	0	-	4	0	0	0	0
chr20	54325342	54338813	dre-circ-3455	0	-	0	8	0	0	0
chr20	54555046	54559197	dre-circ-3456	0	+	0	0	0	0	49
chr20	54767142	54771372	dre-circ-3457	0	-	2	0	0	0	0
chr20	55029269	55067316	dre-circ-3458	0	+	0	3	0	3	0
chr20	6415027	6416002	dre-circ-3459	0	-	4	0	0	0	0
chr20	6707023	6718399	dre-circ-3460	0	+	0	0	3	0	0
chr20	6707023	6725381	dre-circ-3461	0	+	0	0	4	0	0
chr20	7050500	7051069	dre-circ-3462	0	-	48	3	16	4	7
chr20	8162404	8170352	dre-circ-2703	0	-	0	0	0	2	0
chr20	8264914	8269412	dre-circ-3463	0	-	0	0	0	0	3
chr20	8353598	8363989	dre-circ-3464	0	-	0	2	0	0	0
chr20	903687	904095	dre-circ-2698	0	-	4	0	0	0	0
chr2	10335021	10335425	dre-circ-3465	0	-	4	0	0	0	0
chr21	11341102	11342060	dre-circ-3466	0	+	6	2	0	0	0
chr21	11454085	11457043	dre-circ-3467	0	+	6	0	6	3	2
chr21	11566742	11568550	dre-circ-3468	0	+	4	0	0	0	0
chr21	11673247	11675981	dre-circ-3469	0	-	0	2	0	0	0
chr21	13059608	13071042	dre-circ-2782	0	-	3	0	0	0	0
chr21	13505841	13527237	dre-circ-2783	0	+	3	0	0	0	0
chr21	13509642	13566863	dre-circ-2784	0	+	0	0	0	0	6
chr21	13531647	13566863	dre-circ-2785	0	+	0	0	0	0	2
chr2	116709	117898	dre-circ-3470	0	+	7	0	0	2	19
chr21	1672138	1679276	dre-circ-3471	0	+	0	2	0	2	0
chr21	19601909	19671560	dre-circ-3472	0	+	0	0	0	3	0
chr21	19648412	19750892	dre-circ-2786	0	-	0	0	5	0	0
chr21	21430134	21527136	dre-circ-2787	0	-	3	0	0	3	0
chr21	22170826	22175913	dre-circ-3473	0	-	0	0	6	0	0
chr21	24660952	24662082	dre-circ-3474	0	-	2	0	3	0	0
chr21	2628512	2629427	dre-circ-3475	0	+	6	0	0	3	2
chr21	2712836	2713709	dre-circ-2777	0	+	0	0	3	0	0
chr21	2768314	2776264	dre-circ-2778	0	+	0	0	0	0	2
chr2	12934593	12936363	dre-circ-3476	0	+	0	0	2	2	0
chr21	29500122	29500605	dre-circ-3477	0	+	3	0	0	0	0
chr21	2964459	2968677	dre-circ-3478	0	+	9	8	0	3	8
chr21	31125024	31129150	dre-circ-2788	0	-	0	0	2	0	0
chr21	32470971	32472458	dre-circ-3479	0	+	4	3	4	0	0
chr21	32989077	32992496	dre-circ-3480	0	-	2	0	0	0	0
chr21	33220271	33220819	dre-circ-3481	0	-	0	0	0	0	3
chr21	34374195	34385293	dre-circ-3482	0	-	0	0	4	0	0
chr21	34383090	34385293	dre-circ-3483	0	-	0	0	3	0	0
chr2	13459937	13461322	dre-circ-3484	0	+	0	0	4	0	0
chr2	13459937	13465243	dre-circ-3485	0	+	0	0	2	0	0
chr2	13542865	13543279	dre-circ-3486	0	+	2	2	0	0	0
chr21	35847101	35867663	dre-circ-3487	0	-	0	0	0	0	4
chr21	368780	485137	dre-circ-2776	0	-	0	0	0	32	0
chr21	36977249	36983955	dre-circ-3488	0	-	3	0	0	0	0
chr21	36983486	36983955	dre-circ-3489	0	-	17	0	10	0	2
chr21	36983486	36989645	dre-circ-3490	0	-	2	0	0	2	0
chr21	37684438	37686479	dre-circ-3491	0	-	6	0	0	0	0
chr21	37684446	37686479	dre-circ-3492	0	-	26	0	0	0	10
chr21	38161348	38162322	dre-circ-2789	0	-	0	0	4	0	0

chr21	38488451	38488779	dre-circ-3493	0	-	2	0	0	0	0		
chr21	38994853	39002098	dre-circ-3494	0	-	4	0	6	0	2		
chr2	13950764	13956256	dre-circ-3495	0	-	0	0	0	2	0		
chr21	39724459	39732362	dre-circ-3496	0	-	2	0	0	0	0		
chr21	40675959	40683003	dre-circ-3497	0	+	0	2	0	0	0		
chr21	40675959	40683073	dre-circ-3498	0	+	0	10	0	0	0	0	0
chr21	40675959	40683756	dre-circ-3499	0	+	0	10	0	0	0	2	0
chr21	40675959	40683855	dre-circ-3500	0	+	0	3	0	0	0	0	8
chr21	41626015	41628540	dre-circ-3501	0	+	3	0	2	0	0		
chr21	42264572	42267441	dre-circ-3502	0	+	0	3	8	27	3		
chr21	42305983	42458072	dre-circ-2790	0	-	2	0	3	0	0		
chr21	432173	490598	dre-circ-3503	0	-	2	0	0	2	0		
chr21	43627993	43636803	dre-circ-3504	0	-	3	0	2	0	0		
chr21	43674220	43677057	dre-circ-2791	0	-	2	0	0	0	0		
chr21	44072980	44074924	dre-circ-3505	0	+	9	0	0	0	0		
chr21	44261577	44261953	dre-circ-3506	0	-	8	0	0	2	7		
chr21	4914747	4917819	dre-circ-3507	0	-	0	0	0	2	0		
chr21	4940347	4945796	dre-circ-2779	0	-	0	0	3	0	0		
chr21	5829353	5833182	dre-circ-2780	0	+	2	0	0	0	0		
chr21	584572	592260	dre-circ-3508	0	+	0	0	0	0	3		
chr2	15919658	15932415	dre-circ-2258	0	-	0	0	0	0	3		
chr2	16077678	16094663	dre-circ-3509	0	-	0	0	3	0	0		
chr2	16083258	16094663	dre-circ-3510	0	-	0	0	2	0	0		
chr2	17138966	17140726	dre-circ-3511	0	-	4	0	11	0	0		
chr2	17138966	17175331	dre-circ-3512	0	-	0	0	7	0	0		
chr21	7612882	7615139	dre-circ-3513	0	-	2	2	3	6	0		
chr21	7868883	7872140	dre-circ-3514	0	-	0	0	2	0	0		
chr2	181406	182065	dre-circ-3515	0	-	10	0	0	0	0		
chr21	8843922	8850333	dre-circ-3516	0	+	0	0	0	0	3		
chr21	9479068	9498534	dre-circ-3517	0	-	0	0	4	0	0		
chr21	9479068	9499050	dre-circ-2781	0	-	0	0	4	0	0		
chr22	10004353	10010030	dre-circ-3518	0	-	0	0	0	0	5		
chr22	10024416	10029564	dre-circ-2812	0	-	0	0	2	0	0		
chr22	10039348	10043323	dre-circ-3519	0	-	3	3	0	0	5		
chr22	11114610	11115158	dre-circ-3520	0	-	20	0	0	0	0		
chr2	21138892	21139754	dre-circ-2259	0	-	0	0	6	0	0		
chr22	11978578	11981871	dre-circ-3521	0	+	0	0	0	0	5		
chr22	13377327	13383585	dre-circ-2813	0	-	9	0	0	0	0		
chr22	14395571	14395950	dre-circ-3522	0	-	0	0	3	0	0		
chr22	15455858	15483670	dre-circ-2814	0	+	0	0	0	2	0		
chr22	15730655	15732968	dre-circ-3523	0	-	0	0	7	0	0		
chr22	15730819	15731149	dre-circ-2815	0	-	0	0	2	0	0		
chr22	161167	166147	dre-circ-3524	0	+	2	0	0	0	0		
chr22	1639732	1640912	dre-circ-3525	0	+	0	0	0	0	3		
chr22	1639732	1640916	dre-circ-3526	0	+	0	0	0	0	2		
chr22	17979495	17984471	dre-circ-3527	0	-	20	0	0	0	0		
chr22	18577301	18577559	dre-circ-3528	0	+	2	0	0	0	0		
chr22	19271309	19279711	dre-circ-3529	0	-	3	0	8	0	0		
chr22	2014549	2040895	dre-circ-3530	0	+	3	3	0	0	0		
chr22	20457709	20470028	dre-circ-2816	0	+	0	0	0	0	2		
chr22	20482591	20483399	dre-circ-3531	0	-	0	2	2	0	0		
chr22	20649743	20655997	dre-circ-2817	0	-	2	0	0	0	0		
chr22	21134511	21136099	dre-circ-3532	0	-	2	0	0	0	0		
chr22	2122414	2122702	dre-circ-3533	0	+	10	0	0	0	0		
chr22	21330956	21352542	dre-circ-3534	0	-	8	0	0	0	0		
chr22	2195719	2196148	dre-circ-3535	0	-	2	0	0	0	0		
chr22	2397152	2398514	dre-circ-3536	0	-	4	0	0	0	0		
chr22	24442931	24461197	dre-circ-3537	0	-	0	0	0	0	2		
chr22	24709064	24710230	dre-circ-3538	0	-	0	0	0	2	0		
chr22	25188979	25191552	dre-circ-3539	0	+	0	4	0	0	0		
chr22	25210249	25229735	dre-circ-2818	0	-	0	0	4	0	0		
chr2	22524283	22524616	dre-circ-3540	0	+	2	0	0	0	0		
chr22	25260691	25287169	dre-circ-2819	0	-	0	0	0	0	2		
chr22	25260911	25327254	dre-circ-2820	0	-	0	0	2	0	0		
chr22	25614005	25657881	dre-circ-3541	0	+	0	0	0	0	489		
chr22	26723327	26773950	dre-circ-2821	0	-	0	0	0	0	2		
chr22	26731832	26789341	dre-circ-2822	0	-	0	0	0	2	0		
chr22	26737881	26798059	dre-circ-3542	0	-	0	0	3	0	0		
chr22	26754756	26815384	dre-circ-3543	0	-	0	0	0	0	4		
chr22	26759109	26819042	dre-circ-3544	0	-	0	0	0	0	9		
chr22	2707410	2707945	dre-circ-3545	0	+	0	0	11	3	0		
chr22	27373131	27373617	dre-circ-2823	0	+	0	0	0	0	2		
chr22	27421862	27422262	dre-circ-3546	0	+	6	0	0	0	0		
chr22	310170	338577	dre-circ-2792	0	+	0	0	0	8	0		
chr22	31819952	31822673	dre-circ-3547	0	+	0	0	0	2	0		
chr22	32391789	32393716	dre-circ-3548	0	-	0	0	5	0	0		
chr22	32422016	32434213	dre-circ-3549	0	-	0	0	4	0	0		
chr2	2335116	2335256	dre-circ-3550	0	-	0	3	0	0	0		
chr22	33676901	33698218	dre-circ-2824	0	-	0	0	0	2	0		
chr22	3467023	3470297	dre-circ-3551	0	-	0	0	2	0	0		
chr2	23582807	23599524	dre-circ-3552	0	+	0	0	16	0	0		
chr2	23587175	23587483	dre-circ-3553	0	+	0	0	4	0	0		
chr2	23588247	23605696	dre-circ-3554	0	+	0	0	5	0	0		
chr22	37418670	37419218	dre-circ-3555	0	-	2	2	3	0	0		
chr22	39071289	39072904	dre-circ-2825	0	-	0	0	2	0	0		
chr22	39629990	39647103	dre-circ-3556	0	-	7	0	4	0	0		
chr22	39631933	39650351	dre-circ-3557	0	-	3	0	0	0	0		

chr22	39632680	39652046	dre-circ-3558	0	-	0	0	0	10	0
chr22	39781730	39858131	dre-circ-2826	0	+	0	0	0	3	3
chr22	40715168	40718263	dre-circ-3559	0	-	2	0	0	0	5
chr22	40743245	40765549	dre-circ-3560	0	-	0	0	0	0	8
chr2	24103701	24104096	dre-circ-3561	0	+	0	0	2	0	0
chr22	42032257	42033262	dre-circ-3562	0	+	2	0	0	0	0
chr22	4300830	4303551	dre-circ-3563	0	+	0	3	0	0	0
chr2	24492366	24499453	dre-circ-3564	0	+	2	0	0	0	0
chr22	458681	461947	dre-circ-3565	0	-	2	0	0	0	0
chr2	25393327	25395978	dre-circ-3566	0	+	0	0	6	0	0
chr22	5614673	5614984	dre-circ-3567	0	+	53	0	6	4	0
chr2	25725897	25734115	dre-circ-3568	0	+	2	0	0	0	0
chr22	6664493	6769092	dre-circ-2793	0	+	0	0	2	0	0
chr22	6776374	6789618	dre-circ-3569	0	-	2	0	0	0	0
chr22	6852043	6859483	dre-circ-2794	0	-	0	0	2	4	0
chr22	7013876	7046134	dre-circ-3570	0	-	0	0	0	0	2
chr2	27489729	27502604	dre-circ-2260	0	+	0	0	2	2	5
chr2	27790979	27795006	dre-circ-3571	0	-	3	0	0	0	0
chr22	7945374	8131671	dre-circ-2795	0	-	0	3	0	4	0
chr22	8469120	8534144	dre-circ-3572	0	-	0	0	10	10	2
chr22	8630277	8746129	dre-circ-2796	0	-	0	0	0	2	0
chr22	8639882	8757640	dre-circ-2797	0	+	0	0	0	2	0
chr22	8982833	8992178	dre-circ-2798	0	+	0	0	0	0	3
chr2	29003877	29012445	dre-circ-3573	0	+	0	0	4	0	0
chr22	9017253	9031006	dre-circ-2799	0	+	0	0	0	0	3
chr22	9030836	9031006	dre-circ-2800	0	+	0	0	0	0	2
chr22	9120038	9123114	dre-circ-2801	0	-	0	0	0	0	2
chr22	9120038	9126946	dre-circ-2802	0	-	0	0	0	0	2
chr22	9155246	9229106	dre-circ-2803	0	+	0	0	0	0	9
chr22	9165423	9351245	dre-circ-2804	0	+	0	0	0	0	5
chr2	29195961	29196761	dre-circ-2261	0	-	2	0	0	0	0
chr22	9316294	9360397	dre-circ-2805	0	+	6	0	4	5	0
chr22	9359933	9558181	dre-circ-2806	0	+	4	0	0	0	0
chr22	9368258	9374731	dre-circ-2807	0	+	2	0	0	0	0
chr22	9369025	9553201	dre-circ-2808	0	+	0	0	0	0	3
chr22	9384825	9566684	dre-circ-2809	0	+	2	0	0	2	0
chr22	9390297	9566684	dre-circ-2810	0	+	0	0	0	4	0
chr22	9700681	9834352	dre-circ-2811	0	-	2	0	0	0	2
chr2	3074400	3078057	dre-circ-3574	0	+	0	0	2	0	0
chr23	10517705	10532548	dre-circ-2832	0	+	0	0	2	0	0
chr23	1145819	1185209	dre-circ-3575	0	-	6	0	0	6	43
chr23	1172682	1189492	dre-circ-3576	0	-	10	2	3	2	0
chr23	1185080	1189492	dre-circ-3577	0	-	23	0	27	15	0
chr23	13029839	13030751	dre-circ-3578	0	+	4	0	0	0	0
chr23	13660000	13660381	dre-circ-3579	0	+	3	0	0	0	0
chr23	1384153	1421047	dre-circ-3580	0	-	0	0	0	0	3
chr23	1420419	1468691	dre-circ-3581	0	-	0	2	0	0	0
chr23	15186511	15187691	dre-circ-3582	0	-	0	0	3	0	6
chr2	31631075	31637237	dre-circ-3583	0	-	0	3	0	0	0
chr23	16991518	17078632	dre-circ-2833	0	+	0	0	2	0	0
chr23	17080987	17108442	dre-circ-3584	0	+	0	0	0	3	0
chr23	17082575	17116159	dre-circ-2834	0	+	0	0	0	2	0
chr23	17088312	17119196	dre-circ-2835	0	+	0	0	2	0	0
chr23	18057672	18059225	dre-circ-3585	0	+	0	0	2	2	0
chr2	31806434	31807281	dre-circ-2262	0	+	0	0	3	0	0
chr23	18185459	18197645	dre-circ-3586	0	-	0	0	0	0	5
chr23	19218369	19230383	dre-circ-2836	0	-	2	0	0	0	0
chr23	19725900	19726499	dre-circ-3587	0	-	0	0	3	0	0
chr23	21802569	21804715	dre-circ-3588	0	-	0	0	34	0	0
chr23	22312751	22316761	dre-circ-3589	0	+	2	0	0	0	0
chr2	32341718	32360797	dre-circ-2263	0	-	0	0	7	22	0
chr23	23955924	24010016	dre-circ-2837	0	-	0	0	0	0	2
chr23	2554913	2555244	dre-circ-2827	0	+	2	0	0	0	0
chr23	26439773	26442636	dre-circ-3590	0	+	0	0	0	0	2
chr23	27010482	27015173	dre-circ-2838	0	-	0	2	0	0	0
chr23	2769645	2781497	dre-circ-3591	0	+	4	0	0	0	0
chr23	2772074	2774743	dre-circ-3592	0	+	8	0	0	0	0
chr23	2835987	2847282	dre-circ-3593	0	+	0	0	0	2	0
chr23	28443753	28456418	dre-circ-2839	0	-	0	0	2	0	0
chr23	28948717	28950191	dre-circ-3594	0	+	0	2	0	0	0
chr23	29231346	29237041	dre-circ-3595	0	-	0	0	2	0	0
chr23	29709055	29712199	dre-circ-2840	0	-	0	0	0	3	0
chr23	30176458	30190343	dre-circ-3596	0	+	3	0	0	0	0
chr23	30975114	30975837	dre-circ-3597	0	+	5	0	0	0	0
chr23	31672000	31672413	dre-circ-3598	0	+	7	0	3	0	0
chr23	32282975	32291921	dre-circ-3599	0	+	0	0	2	0	0
chr23	32284137	32286291	dre-circ-3600	0	+	0	0	2	0	0
chr23	32284167	32286291	dre-circ-3601	0	+	0	0	36	0	0
chr23	33676767	33680037	dre-circ-3602	0	-	0	0	8	0	0
chr23	34363288	34365107	dre-circ-3603	0	+	6	0	0	0	0
chr23	349638	351998	dre-circ-3604	0	-	3	0	0	0	0
chr23	35039715	35055060	dre-circ-2841	0	-	0	0	2	0	0
chr23	35361164	35362333	dre-circ-3605	0	-	0	0	2	0	0
chr23	35797556	35798375	dre-circ-3606	0	-	0	0	3	0	0
chr2	33765297	33765837	dre-circ-3607	0	-	4	0	2	0	0
chr23	37887085	37889276	dre-circ-3608	0	+	4	0	8	11	2
chr23	38889562	38893817	dre-circ-2842	0	-	2	0	0	0	0

chr23	39725983	39726563	dre-circ-3609	0	+	0	0	0	0	2
chr23	401066	405247	dre-circ-3610	0	+	3	0	0	0	0
chr23	41148039	41154776	dre-circ-3611	0	-	0	0	0	3	0
chr2	3413282	3589763	dre-circ-2252	0	+	4	0	0	0	8
chr23	42958444	42986438	dre-circ-3612	0	-	0	0	0	0	4
chr23	44142832	44145351	dre-circ-3613	0	+	0	0	3	0	0
chr23	44142832	44145356	dre-circ-3614	0	+	0	0	8	0	0
chr23	45257115	45261362	dre-circ-2843	0	+	0	0	0	0	2
chr23	45279345	45281810	dre-circ-2844	0	+	0	0	0	0	3
chr23	45436407	45442786	dre-circ-3615	0	+	2	0	0	0	0
chr23	46100405	46101455	dre-circ-3616	0	-	9	0	0	0	2
chr23	46100405	46107703	dre-circ-3617	0	-	4	0	2	2	0
chr23	46105555	46107703	dre-circ-2845	0	-	2	0	0	0	0
chr23	46116458	46116657	dre-circ-3618	0	-	7	0	0	0	0
chr23	4714003	4717417	dre-circ-2828	0	+	2	0	0	0	0
chr2	3508155	3512423	dre-circ-2253	0	-	2	0	0	0	0
chr2	3512879	3513082	dre-circ-3619	0	-	3	0	0	0	0
chr23	549189	552121	dre-circ-3620	0	-	6	0	0	0	0
chr23	549189	552124	dre-circ-3621	0	-	4	0	0	0	4
chr23	5557595	5571771	dre-circ-3622	0	+	0	0	4	0	0
chr23	5658798	5661713	dre-circ-3623	0	-	0	0	2	0	0
chr23	5716529	5716909	dre-circ-2829	0	-	0	0	9	0	0
chr23	5716529	5716966	dre-circ-2830	0	-	0	0	4	0	0
chr23	6187974	6189002	dre-circ-2831	0	+	4	0	0	0	0
chr23	6188356	6188619	dre-circ-3624	0	+	7	0	4	2	0
chr23	6396590	6402082	dre-circ-3625	0	-	0	0	4	0	3
chr23	6651678	6664169	dre-circ-3626	0	+	2	0	0	0	0
chr23	6963215	6972914	dre-circ-3627	0	-	0	0	3	0	0
chr23	7069336	7069794	dre-circ-3628	0	-	2	0	0	0	0
chr2	37095842	37096175	dre-circ-3629	0	-	0	0	3	0	0
chr2	37178925	37179206	dre-circ-3630	0	-	0	0	0	3	0
chr2	37408796	37420369	dre-circ-3631	0	-	0	0	0	0	2
chr2	37943701	37945195	dre-circ-3632	0	-	0	0	13	8	0
chr23	954309	958348	dre-circ-3633	0	-	2	0	0	0	0
chr23	9575770	9593555	dre-circ-3634	0	-	3	0	0	0	0
chr23	961153	972375	dre-circ-3635	0	-	0	0	7	0	4
chr23	9813080	9816264	dre-circ-3636	0	-	0	0	0	2	0
chr24	14527734	14534724	dre-circ-2849	0	-	0	0	2	0	0
chr24	16644883	16738232	dre-circ-2850	0	-	0	0	0	2	4
chr24	17560879	17565366	dre-circ-3637	0	+	12	0	9	0	0
chr24	20618986	20647314	dre-circ-2851	0	-	0	0	0	11	0
chr2	42076344	42076901	dre-circ-2264	0	-	2	0	0	0	0
chr24	21521539	21529936	dre-circ-3638	0	+	0	4	9	4	28
chr24	21820745	21826138	dre-circ-2852	0	-	0	0	2	0	0
chr24	22019390	22019926	dre-circ-3639	0	-	7	0	0	0	4
chr24	22043522	22046198	dre-circ-3640	0	-	6	0	0	0	0
chr24	22043522	22053508	dre-circ-3641	0	-	5	0	0	0	3
chr24	22043522	22058441	dre-circ-3642	0	-	0	0	5	0	2
chr2	42218842	42226283	dre-circ-3643	0	-	0	0	7	0	0
chr24	24805158	24810327	dre-circ-2853	0	-	0	0	2	0	0
chr24	2498598	2499173	dre-circ-3644	0	-	0	0	0	0	3
chr2	42528931	42533425	dre-circ-3645	0	-	0	2	0	0	0
chr2	42555860	42560763	dre-circ-3646	0	-	3	3	15	0	9
chr24	25631373	25632082	dre-circ-2854	0	-	0	0	2	0	0
chr24	26688776	26692374	dre-circ-3647	0	+	4	2	3	6	4
chr24	26688776	26694808	dre-circ-3648	0	+	5	7	3	2	0
chr24	28109032	28109256	dre-circ-2855	0	-	2	0	0	0	0
chr24	30596701	30597004	dre-circ-3649	0	+	0	0	0	6	0
chr24	30820087	30823974	dre-circ-3650	0	-	0	6	0	2	0
chr24	30958382	30960638	dre-circ-3651	0	+	0	0	5	0	0
chr24	3111401	3113342	dre-circ-2846	0	-	0	0	0	3	0
chr24	32611308	32621774	dre-circ-2856	0	+	0	0	0	2	0
chr24	32824876	32841875	dre-circ-2857	0	+	0	2	0	0	0
chr2	43287990	43292583	dre-circ-3652	0	-	13	0	0	0	0
chr24	34459948	34462418	dre-circ-3653	0	-	3	0	0	0	0
chr24	34463297	34469908	dre-circ-2858	0	-	2	0	0	0	0
chr24	34468020	34469908	dre-circ-3654	0	-	4	0	0	0	0
chr24	3452388	3456065	dre-circ-3655	0	-	0	0	0	0	3
chr2	43635255	43635545	dre-circ-3656	0	+	10	0	0	0	0
chr24	36488141	36491118	dre-circ-3657	0	+	3	0	0	0	0
chr24	37471815	37473770	dre-circ-3658	0	+	0	0	18	8	0
chr24	37763427	37763760	dre-circ-3659	0	-	42	3	11	7	4
chr24	37777102	37777503	dre-circ-3660	0	-	17	0	7	3	3
chr24	37786450	37795988	dre-circ-3661	0	-	5	0	6	0	0
chr24	37876688	37880605	dre-circ-3662	0	+	0	0	5	0	0
chr24	39050893	39052971	dre-circ-2859	0	+	0	0	2	0	0
chr2	43940328	44007120	dre-circ-2265	0	+	0	0	3	0	0
chr2	43966086	43967197	dre-circ-3663	0	+	0	0	0	0	3
chr24	39680494	39682260	dre-circ-3664	0	+	0	11	0	17	0
chr24	39701003	39717867	dre-circ-2860	0	+	0	0	2	4	0
chr2	43976623	44009891	dre-circ-3665	0	+	2	0	0	0	3
chr24	39808856	39810102	dre-circ-2861	0	+	0	0	0	3	0
chr24	40037741	40051472	dre-circ-2862	0	+	0	0	6	0	0
chr24	42243670	42348562	dre-circ-2863	0	-	0	0	0	0	4
chr24	42246630	42339088	dre-circ-2864	0	+	0	0	0	2	0
chr24	42246913	42321240	dre-circ-3666	0	-	0	0	0	10	0
chr24	42267640	42299805	dre-circ-3667	0	-	0	0	0	0	3

chr24	42267640	42457295	dre-circ-2865	0	-	0	0	0	8	0
chr24	42268837	42458338	dre-circ-2866	0	-	0	0	0	0	3
chr24	42269718	42350094	dre-circ-3668	0	-	0	0	2	0	0
chr24	42270392	42459941	dre-circ-2867	0	-	4	0	0	0	0
chr24	42273251	42322504	dre-circ-2868	0	-	2	0	0	0	0
chr24	42298214	42471571	dre-circ-2869	0	-	0	0	0	2	0
chr24	42317161	42348562	dre-circ-2870	0	-	0	0	0	6	0
chr24	42322589	42511711	dre-circ-2871	0	-	0	0	0	0	2
chr24	43528323	43542737	dre-circ-3669	0	+	0	0	0	0	2
chr24	43834346	43834856	dre-circ-3670	0	-	5	0	0	0	0
chr2	44664536	44665722	dre-circ-3671	0	+	8	0	5	0	0
chr2	44673398	44673601	dre-circ-3672	0	+	5	0	0	0	0
chr2	45030656	45031191	dre-circ-3673	0	-	2	0	0	0	0
chr2	4559345	4561050	dre-circ-3674	0	-	4	0	0	0	0
chr2	4727048	4727368	dre-circ-2254	0	-	2	0	0	0	0
chr2	47426512	47431795	dre-circ-2266	0	+	0	0	0	0	2
chr2	4747859	4748240	dre-circ-2255	0	-	2	0	0	0	0
chr24	7536706	7537049	dre-circ-2847	0	+	2	0	0	0	0
chr2	47877104	47877646	dre-circ-3675	0	+	5	0	0	0	0
chr2	47938439	47943301	dre-circ-3676	0	+	5	0	0	0	0
chr2	47968053	47990877	dre-circ-2267	0	-	3	0	0	0	0
chr2	4818654	4819557	dre-circ-3677	0	-	0	0	3	0	0
chr2	49055638	49158927	dre-circ-2268	0	+	3	0	0	0	6
chr2	49346854	49358313	dre-circ-3678	0	-	0	0	0	3	0
chr24	9806849	9815033	dre-circ-2848	0	-	0	0	0	0	3
chr2	5010315	5013996	dre-circ-3679	0	+	3	0	3	0	0
chr25	11613393	11618556	dre-circ-3680	0	+	0	0	2	0	0
chr25	12597186	12600995	dre-circ-3681	0	+	0	0	4	0	0
chr25	13358935	13361144	dre-circ-3682	0	+	0	0	2	3	0
chr25	14865157	14890001	dre-circ-3683	0	-	0	0	2	0	0
chr2	51667212	51670594	dre-circ-3684	0	+	2	0	0	15	0
chr25	16974527	16974896	dre-circ-3685	0	-	2	0	0	0	0
chr25	1698452	1699432	dre-circ-3686	0	-	2	0	0	0	0
chr2	51760776	51769133	dre-circ-3687	0	-	2	0	0	0	0
chr25	17684518	17685240	dre-circ-3688	0	+	2	0	0	0	0
chr25	1881	28256	dre-circ-3689	0	+	0	0	0	6	3
chr25	20391520	20391806	dre-circ-3690	0	+	3	0	0	0	0
chr25	20578997	20586330	dre-circ-3691	0	+	3	0	0	6	0
chr25	20685680	20692094	dre-circ-2882	0	+	0	0	0	0	2
chr25	21342375	21345123	dre-circ-2883	0	+	0	0	4	0	0
chr25	21978947	21984104	dre-circ-3692	0	-	0	2	0	0	0
chr25	22850335	22856739	dre-circ-3693	0	+	0	0	0	3	0
chr25	24284888	24285855	dre-circ-3694	0	-	7	3	0	0	2
chr25	24349065	24351358	dre-circ-3695	0	+	3	0	3	0	0
chr2	52481253	52490381	dre-circ-3696	0	+	0	0	0	0	2
chr25	26738783	26739425	dre-circ-3697	0	+	0	2	3	0	0
chr25	27607207	27610180	dre-circ-3698	0	-	3	0	0	0	0
chr25	29151716	29154430	dre-circ-3699	0	+	0	0	9	8	4
chr25	29746028	29750831	dre-circ-3700	0	+	15	0	0	2	0
chr25	31227653	31230615	dre-circ-2884	0	+	4	0	0	0	0
chr25	31456721	31460189	dre-circ-3701	0	+	14	0	0	0	0
chr25	32197516	32200842	dre-circ-3702	0	+	0	0	3	0	0
chr25	33750736	33754258	dre-circ-3703	0	+	0	0	0	0	2
chr25	3474629	3493838	dre-circ-2874	0	+	0	0	2	0	0
chr2	5352083	5357962	dre-circ-3704	0	-	0	0	0	2	0
chr2	5354039	5357962	dre-circ-3705	0	-	3	0	0	0	0
chr25	3563619	3566306	dre-circ-3706	0	-	6	0	0	0	0
chr25	36708670	36710042	dre-circ-3707	0	-	0	0	0	0	4
chr25	36763508	36763974	dre-circ-3708	0	+	0	0	0	0	2
chr2	53830581	53838763	dre-circ-3709	0	-	0	0	2	0	0
chr25	4035486	4036189	dre-circ-3710	0	-	0	0	0	0	3
chr2	54228300	54228574	dre-circ-3711	0	+	3	0	4	0	0
chr2	54243838	54244171	dre-circ-3712	0	+	15	0	0	5	3
chr25	454568	572404	dre-circ-2872	0	-	6	0	3	2	0
chr2	54861718	54878541	dre-circ-3713	0	+	0	0	2	0	0
chr25	4935881	4936160	dre-circ-2875	0	+	0	0	0	2	0
chr25	5185501	5185733	dre-circ-2876	0	+	2	0	0	0	0
chr2	55398718	55479330	dre-circ-3714	0	-	8	4	7	5	18
chr2	5558342	5561773	dre-circ-3715	0	+	0	2	0	0	0
chr25	5956556	5960162	dre-circ-2877	0	+	0	2	0	0	0
chr25	6006308	6008481	dre-circ-3716	0	+	2	0	0	0	0
chr2	56646291	56646680	dre-circ-2269	0	-	0	0	2	0	0
chr25	6719162	6719403	dre-circ-2878	0	-	0	0	5	0	0
chr25	704873	705733	dre-circ-3717	0	+	11	0	2	0	0
chr2	57091142	57091352	dre-circ-3718	0	+	0	3	0	0	0
chr2	57410233	57410727	dre-circ-3719	0	-	2	0	0	0	0
chr25	7848628	7867138	dre-circ-2879	0	+	0	0	0	0	3
chr25	7988245	7991167	dre-circ-2880	0	-	4	0	0	0	0
chr25	8090607	8090845	dre-circ-2881	0	+	2	0	0	0	0
chr2	58106226	58113632	dre-circ-3720	0	+	0	0	6	0	0
chr25	8573258	8624060	dre-circ-3721	0	+	10	0	0	0	0
chr25	8574885	8624766	dre-circ-3722	0	+	0	0	0	0	37
chr2	59007410	59018755	dre-circ-3723	0	-	0	0	4	3	0
chr25	948312	1036714	dre-circ-3724	0	+	0	5	0	0	0
chr2	59619431	59620068	dre-circ-3725	0	+	6	0	5	8	2
chr2	59619431	59620094	dre-circ-3726	0	+	3	0	0	0	0
chr25	963139	967006	dre-circ-2873	0	-	2	0	0	0	0

chr2	7466911	7473987	dre-circ-2256	0	-	0	0	0	2	0
chr2	7470755	7473987	dre-circ-3727	0	-	0	0	4	0	0
chr2	9313761	9457784	dre-circ-2257	0	+	0	0	0	0	54
chr2	9324789	9328823	dre-circ-3728	0	+	0	4	0	0	0
chr2	9817145	9817587	dre-circ-3729	0	+	0	0	0	0	8
chr3	1075902	1079780	dre-circ-2270	0	-	0	0	0	0	7
chr3	11505502	11506771	dre-circ-2280	0	-	3	0	0	0	0
chr3	11505502	11516635	dre-circ-3730	0	-	2	0	0	0	0
chr3	12416171	12426060	dre-circ-2281	0	-	0	0	0	0	2
chr3	13433829	13434071	dre-circ-3731	0	-	5	0	0	0	0
chr3	13978817	14043678	dre-circ-2282	0	+	0	0	0	0	5
chr3	14043373	14065711	dre-circ-3732	0	+	2	0	0	0	2
chr3	15040640	15041271	dre-circ-2283	0	-	2	0	0	0	2
chr3	15327149	15327636	dre-circ-3733	0	-	0	0	0	2	0
chr3	15550030	15556678	dre-circ-3734	0	+	25	7	12	7	0
chr3	15550030	15562572	dre-circ-3735	0	+	0	0	0	2	0
chr3	16503081	16503449	dre-circ-3736	0	+	2	0	0	0	0
chr3	16576082	16576963	dre-circ-3737	0	+	2	0	0	0	0
chr3	16929020	16933501	dre-circ-3738	0	+	0	0	4	0	0
chr3	16936126	16936871	dre-circ-3739	0	+	20	0	0	8	2
chr3	17337153	17339667	dre-circ-3740	0	+	4	0	4	0	0
chr3	17956676	17995564	dre-circ-2284	0	-	0	0	0	0	2
chr3	18342703	18436928	dre-circ-2285	0	+	0	0	5	0	0
chr3	18993897	18994239	dre-circ-3741	0	-	2	0	0	0	0
chr3	19893018	19895858	dre-circ-2286	0	+	0	0	2	0	0
chr3	21202194	21202553	dre-circ-3742	0	-	3	0	0	0	0
chr3	22292280	22293519	dre-circ-3743	0	+	2	0	0	0	0
chr3	22337573	22343483	dre-circ-2287	0	+	3	0	0	0	0
chr3	22700560	22703370	dre-circ-3744	0	+	10	0	0	0	0
chr3	23485672	23504198	dre-circ-3745	0	+	0	0	0	0	3
chr3	23625625	23626332	dre-circ-3746	0	-	0	0	0	4	0
chr3	24148581	24152178	dre-circ-3747	0	-	0	0	0	0	4
chr3	24575249	24578940	dre-circ-2288	0	+	4	0	0	0	0
chr3	24851219	24855166	dre-circ-2289	0	+	2	0	0	0	0
chr3	26120386	26123254	dre-circ-3748	0	+	0	0	2	0	0
chr3	26760114	26764620	dre-circ-2290	0	-	0	0	0	2	0
chr3	29492619	29493072	dre-circ-3749	0	+	0	2	0	0	0
chr3	30076546	30079609	dre-circ-2291	0	-	0	0	3	0	0
chr3	30453923	30455358	dre-circ-2292	0	+	0	0	0	2	0
chr3	30456064	30456446	dre-circ-3750	0	+	0	0	0	2	0
chr3	31825249	31827924	dre-circ-2293	0	+	0	0	0	4	0
chr3	31895873	31913184	dre-circ-3751	0	-	0	0	10	0	0
chr3	32330048	32333591	dre-circ-3752	0	-	0	0	9	0	0
chr3	34017435	34017964	dre-circ-2294	0	-	0	2	0	0	0
chr3	34796290	34799810	dre-circ-3753	0	-	4	0	0	0	0
chr3	34810102	34828938	dre-circ-3754	0	-	4	0	0	0	0
chr3	35590516	35597144	dre-circ-2295	0	+	2	0	0	0	0
chr3	35595336	35597144	dre-circ-2296	0	+	3	0	0	0	0
chr3	38668312	38668904	dre-circ-3755	0	-	0	32	0	0	0
chr3	39133694	39142852	dre-circ-3756	0	-	0	0	0	3	0
chr3	39146968	39147444	dre-circ-3757	0	-	0	0	3	4	0
chr3	3927933	3943216	dre-circ-2271	0	-	0	0	0	0	264
chr3	3929091	3955869	dre-circ-3758	0	-	0	0	0	0	28
chr3	3940475	3953896	dre-circ-2272	0	-	0	0	2	0	0
chr3	40610670	40613234	dre-circ-3759	0	-	5	0	0	0	0
chr3	40887943	40888325	dre-circ-2297	0	-	2	0	0	0	0
chr3	41277434	41287055	dre-circ-3760	0	-	0	0	7	0	2
chr3	42087941	42088359	dre-circ-3761	0	+	0	0	0	0	2
chr3	42552364	42599390	dre-circ-3762	0	+	11	0	0	0	0
chr3	42552614	42599644	dre-circ-3763	0	+	0	0	0	0	12
chr3	42559919	42602876	dre-circ-3764	0	+	0	0	0	0	11
chr3	42636231	42646331	dre-circ-2298	0	-	0	0	2	0	0
chr3	4407759	4421672	dre-circ-2273	0	-	0	0	0	0	4
chr3	48562382	48567166	dre-circ-2299	0	+	0	0	0	0	2
chr3	49369628	49375053	dre-circ-2300	0	+	4	0	0	0	0
chr3	49369628	49375216	dre-circ-3765	0	+	6	0	3	0	0
chr3	50572070	50572527	dre-circ-3766	0	+	7	0	0	104	0
chr3	50572126	50572527	dre-circ-3767	0	+	0	0	0	2	0
chr3	5122729	5174619	dre-circ-2274	0	-	0	0	0	4	0
chr3	5125570	5149041	dre-circ-3768	0	-	8	0	0	0	0
chr3	52034406	52034732	dre-circ-2301	0	-	0	0	0	2	0
chr3	55306548	55307709	dre-circ-3769	0	-	0	0	5	0	0
chr3	55313041	55321017	dre-circ-2302	0	-	0	0	2	0	0
chr3	55600064	55635575	dre-circ-2303	0	+	0	0	0	3	0
chr3	55600064	55661468	dre-circ-2304	0	+	0	0	4	0	0
chr3	55618936	55619569	dre-circ-3770	0	+	0	7	83	0	20
chr3	55636246	55661896	dre-circ-2305	0	+	0	0	0	0	14
chr3	55643227	55662357	dre-circ-3771	0	+	0	0	4	0	0
chr3	55951805	55952113	dre-circ-2306	0	-	2	0	0	0	0
chr3	55952507	55957610	dre-circ-2307	0	-	12	0	5	0	0
chr3	57328345	57484557	dre-circ-2308	0	+	0	0	3	0	0
chr3	58311790	58317123	dre-circ-2309	0	-	2	0	0	0	0
chr3	59065289	59065462	dre-circ-2310	0	-	0	0	4	0	4
chr3	59438055	59480260	dre-circ-2311	0	-	0	0	0	0	2
chr3	59454370	59457950	dre-circ-2312	0	-	0	0	0	0	4
chr3	59454370	59485294	dre-circ-2313	0	-	0	0	2	0	0
chr3	59458356	59488680	dre-circ-3772	0	-	0	0	2	0	3

chr3	60965082	60975293	dre-circ-3773	0	-	35	0	9	27	49
chr3	61639422	61639632	dre-circ-2314	0	-	2	0	0	0	0
chr3	62740522	62741958	dre-circ-3774	0	-	11	3	2	5	0
chr3	62915929	62983034	dre-circ-2315	0	+	0	0	3	0	0
chr3	63181933	63182180	dre-circ-3775	0	-	0	0	0	0	3
chr3	7023800	7049872	dre-circ-2275	0	-	2	0	0	3	2
chr3	7477887	7538980	dre-circ-3776	0	-	3	4	0	0	2
chr3	7490045	7569171	dre-circ-2276	0	-	0	0	0	0	2
chr3	7680298	7717502	dre-circ-2277	0	-	2	0	2	0	0
chr3	7680298	7743276	dre-circ-2278	0	-	0	2	0	0	0
chr3	8056880	8130835	dre-circ-2279	0	+	3	0	2	0	6
chr3	8250853	8251177	dre-circ-3777	0	+	6	0	3	0	0
chr3	9997741	9998387	dre-circ-3778	0	+	3	0	0	0	0
chr4	10676784	10692897	dre-circ-3779	0	+	5	0	0	0	0
chr4	10678005	10686793	dre-circ-2323	0	-	0	0	2	0	0
chr4	11938923	11947736	dre-circ-3780	0	-	2	0	0	0	0
chr4	13551090	13703574	dre-circ-2324	0	-	0	0	2	0	0
chr4	13551090	13711229	dre-circ-2325	0	-	0	0	0	0	2
chr4	15007467	15009264	dre-circ-3781	0	+	0	0	0	0	3
chr4	15836882	15841501	dre-circ-2326	0	-	0	0	0	0	7
chr4	170959	171206	dre-circ-3782	0	+	0	0	0	0	2
chr4	17391945	17404707	dre-circ-2327	0	-	0	2	0	0	0
chr4	19307946	19311859	dre-circ-3783	0	+	0	2	2	0	0
chr4	20274583	20277712	dre-circ-3784	0	-	0	0	0	2	5
chr4	20274583	20287618	dre-circ-2328	0	-	0	0	0	2	2
chr4	20274583	20305210	dre-circ-3785	0	-	0	0	0	2	0
chr4	20475324	20480457	dre-circ-3786	0	-	6	0	0	0	0
chr4	24743116	24768168	dre-circ-3787	0	+	0	0	0	2	0
chr4	24832710	24833713	dre-circ-3788	0	+	0	0	2	0	0
chr4	4790785	4792456	dre-circ-3789	0	+	0	0	2	0	0
chr4	50304747	50305872	dre-circ-2329	0	-	0	0	7	0	0
chr4	5297184	5327705	dre-circ-2316	0	-	0	0	5	0	0
chr4	5325287	5328863	dre-circ-2317	0	+	7	0	0	0	0
chr4	58325572	58356110	dre-circ-2330	0	-	0	0	2	0	0
chr4	58814841	58822598	dre-circ-2331	0	-	0	4	0	0	5
chr4	59223506	59225347	dre-circ-3790	0	-	0	0	2	0	0
chr4	59352601	59397220	dre-circ-3791	0	-	52	22	56	42	67
chr4	59511018	59516239	dre-circ-3792	0	-	0	0	2	0	0
chr4	60489765	60514436	dre-circ-2332	0	+	0	0	0	2	0
chr4	60505674	60545021	dre-circ-2333	0	+	0	0	0	17	0
chr4	60581972	60588454	dre-circ-3793	0	+	0	0	0	3	0
chr4	60630224	60742426	dre-circ-2334	0	+	0	0	0	2	0
chr4	60630224	60792225	dre-circ-2335	0	+	0	0	3	0	0
chr4	60717285	60806495	dre-circ-2336	0	+	0	0	0	0	2
chr4	61176870	61177538	dre-circ-2337	0	+	0	0	0	0	3
chr4	6552562	6558075	dre-circ-2318	0	+	2	0	0	0	0
chr4	6894820	7003341	dre-circ-2319	0	+	0	0	2	0	0
chr4	7446747	7449637	dre-circ-2320	0	+	7	0	0	6	5
chr4	7513474	7520822	dre-circ-2321	0	-	0	0	0	0	2
chr4	9606345	9618122	dre-circ-3794	0	+	0	0	0	0	3
chr4	9837518	9852062	dre-circ-3795	0	-	4	0	2	0	0
chr4	9839103	9848954	dre-circ-2322	0	-	2	0	0	0	0
chr5	10438025	10540104	dre-circ-2342	0	-	0	0	2	2	6
chr5	1354893	1357876	dre-circ-3796	0	-	3	0	0	0	0
chr5	13940821	13941291	dre-circ-3797	0	-	8	4	0	0	0
chr5	14909369	14909512	dre-circ-3798	0	-	2	0	0	0	0
chr5	16954507	17002200	dre-circ-2343	0	-	2	0	0	0	0
chr5	1786995	1787713	dre-circ-2339	0	-	4	0	0	0	0
chr5	1925227	1927714	dre-circ-3799	0	-	3	0	0	0	0
chr5	1995591	1999025	dre-circ-2340	0	+	2	0	0	0	0
chr5	2027555	2028340	dre-circ-2341	0	+	0	0	2	0	0
chr5	21966681	21977889	dre-circ-2344	0	-	0	0	0	2	0
chr5	22747268	22769720	dre-circ-2345	0	-	0	0	3	0	0
chr5	22750104	22769720	dre-circ-2346	0	-	0	0	2	0	0
chr5	22947265	22949744	dre-circ-3800	0	+	4	6	11	2	0
chr5	24054479	24066826	dre-circ-3801	0	+	0	0	6	0	0
chr5	24054479	24084624	dre-circ-3802	0	+	0	0	2	0	0
chr5	24084457	24096308	dre-circ-3803	0	+	0	0	2	0	0
chr5	24108021	24111139	dre-circ-3804	0	+	0	0	4	0	0
chr5	24770399	24772137	dre-circ-3805	0	+	2	0	0	2	0
chr5	24885317	24885931	dre-circ-3806	0	+	23	0	9	17	24
chr5	25338783	25339103	dre-circ-2347	0	-	2	0	0	0	0
chr5	25850177	25850689	dre-circ-3807	0	+	0	0	5	2	0
chr5	26543218	26547907	dre-circ-3808	0	-	4	4	4	0	9
chr5	27220772	27223949	dre-circ-3809	0	-	0	0	0	3	0
chr5	27967224	27979135	dre-circ-3810	0	-	0	0	5	0	0
chr5	27967224	28014201	dre-circ-3811	0	-	0	0	18	0	0
chr5	27974703	28014201	dre-circ-3812	0	-	0	0	5	0	0
chr5	28000448	28014201	dre-circ-3813	0	-	0	0	3	0	0
chr5	2826818	2828902	dre-circ-3814	0	+	0	0	2	0	0
chr5	28443218	28443803	dre-circ-3815	0	+	2	0	0	0	0
chr5	30424678	30425615	dre-circ-2348	0	+	3	0	0	0	2
chr5	30436811	30469318	dre-circ-2349	0	+	0	0	0	4	0
chr5	30487215	30496071	dre-circ-2350	0	+	0	0	2	0	0
chr5	30513739	30516617	dre-circ-3816	0	-	0	0	2	0	0
chr5	30877133	30880870	dre-circ-2351	0	-	0	0	0	0	2
chr5	32091056	32095267	dre-circ-3817	0	-	0	0	2	0	0

chr5	33851010	33866897	dre-circ-3818	0	+	4	0	0	0	0
chr5	33854480	33870577	dre-circ-3819	0	+	0	0	0	13	0
chr5	34102009	34104766	dre-circ-3820	0	-	0	0	6	3	0
chr5	34119	52763	dre-circ-2338	0	-	0	3	0	0	0
chr5	35190903	35192885	dre-circ-3821	0	-	0	0	2	2	0
chr5	3914663	3915366	dre-circ-3822	0	+	6	3	2	0	4
chr5	39164434	39168545	dre-circ-2352	0	+	0	0	2	0	0
chr5	39921264	39928095	dre-circ-3823	0	-	0	0	0	0	3
chr5	40618764	40620228	dre-circ-3824	0	+	0	2	2	2	0
chr5	43206745	43207406	dre-circ-3825	0	-	2	0	0	0	0
chr5	43451323	43454582	dre-circ-3826	0	-	0	0	0	0	3
chr5	43451577	43458170	dre-circ-3827	0	-	0	0	0	0	8
chr5	43509901	43510269	dre-circ-3828	0	-	3	0	0	0	0
chr5	43528886	43553265	dre-circ-3829	0	-	0	0	2	0	0
chr5	43540922	43546037	dre-circ-3830	0	-	3	0	0	0	0
chr5	43540922	43553262	dre-circ-3831	0	-	0	0	6	0	0
chr5	43540922	43553265	dre-circ-3832	0	-	0	0	3	0	0
chr5	43545975	43553262	dre-circ-3833	0	-	2	0	5	0	0
chr5	43545975	43553265	dre-circ-3834	0	-	2	0	6	0	0
chr5	43550730	43553265	dre-circ-3835	0	-	2	0	0	0	0
chr5	47743546	47743933	dre-circ-3836	0	+	0	0	0	9	0
chr5	54886169	54886809	dre-circ-3837	0	+	0	0	3	0	0
chr5	55638224	55644350	dre-circ-3838	0	-	0	0	0	8	0
chr5	58344091	58346373	dre-circ-3839	0	-	0	0	3	0	0
chr5	58500893	58501424	dre-circ-3840	0	-	2	0	0	0	0
chr5	58633163	58667828	dre-circ-2353	0	+	2	0	0	0	0
chr5	58786699	58790111	dre-circ-3841	0	+	8	0	0	0	0
chr5	59104513	59104984	dre-circ-3842	0	+	3	0	0	0	0
chr5	59424363	59427920	dre-circ-2354	0	-	0	2	0	0	0
chr5	60943911	60946160	dre-circ-3843	0	+	0	0	0	0	2
chr5	6126002	6129825	dre-circ-3844	0	-	0	2	0	0	0
chr5	62717234	62721127	dre-circ-2355	0	+	2	0	0	0	0
chr5	63698808	63699038	dre-circ-3845	0	+	0	2	0	0	0
chr5	64438503	64442866	dre-circ-3846	0	+	3	0	0	0	0
chr5	65374229	65374490	dre-circ-3847	0	-	0	0	0	3	0
chr5	65693416	65693677	dre-circ-3848	0	+	0	0	2	0	0
chr5	66490999	66548674	dre-circ-2356	0	+	0	0	0	0	4
chr5	66514861	66531946	dre-circ-3849	0	+	0	0	0	0	3
chr5	66716866	66719466	dre-circ-3850	0	+	0	4	0	12	0
chr5	66735550	66737099	dre-circ-3851	0	+	0	0	3	3	0
chr5	67503879	67505975	dre-circ-2357	0	-	0	0	0	0	2
chr5	67505365	67519552	dre-circ-2358	0	-	4	0	0	0	0
chr5	67507928	67523993	dre-circ-3852	0	-	3	0	0	0	0
chr5	67526886	67542921	dre-circ-3853	0	-	10	0	0	0	0
chr5	68679840	68680186	dre-circ-3854	0	-	0	0	8	3	0
chr5	68698966	68713097	dre-circ-2359	0	-	0	0	2	0	0
chr5	68707103	68713097	dre-circ-3855	0	-	0	0	11	0	0
chr5	69227613	69227963	dre-circ-3856	0	-	3	2	0	0	0
chr5	69239854	69251577	dre-circ-2360	0	+	0	0	2	0	0
chr5	70259855	70262879	dre-circ-2361	0	-	0	0	2	0	0
chr5	70272908	70275222	dre-circ-3857	0	-	7	0	0	0	0
chr5	70336724	70336999	dre-circ-3858	0	-	13	2	4	6	0
chr5	70565197	70565386	dre-circ-3859	0	-	15	0	0	0	0
chr5	71811845	71816733	dre-circ-3860	0	-	0	2	0	0	0
chr5	71884825	71885036	dre-circ-3861	0	-	7	0	0	0	0
chr5	71899332	71900972	dre-circ-3862	0	-	2	0	0	0	0
chr5	71913199	71914524	dre-circ-3863	0	-	7	0	0	0	0
chr5	72023715	72027118	dre-circ-3864	0	-	0	4	0	0	4
chr5	72304100	72379006	dre-circ-3865	0	-	2	0	0	0	2
chr5	72426330	72427992	dre-circ-3866	0	-	7	0	0	0	3
chr5	72451150	72547360	dre-circ-2362	0	-	0	0	2	0	0
chr5	72528616	72547360	dre-circ-2363	0	-	0	0	2	0	0
chr5	7363549	7368129	dre-circ-3867	0	+	0	5	0	0	0
chr5	75452412	75455412	dre-circ-3868	0	+	0	0	4	0	0
chr5	8761075	8842843	dre-circ-3869	0	-	2	0	2	0	0
chr5	9413340	9415655	dre-circ-3870	0	+	2	0	0	2	0
chr6	10896816	10897152	dre-circ-2368	0	-	2	0	0	0	0
chr6	11708734	11711368	dre-circ-3871	0	-	4	0	0	0	0
chr6	12536783	12539294	dre-circ-2369	0	-	0	0	0	2	0
chr6	12732947	12733239	dre-circ-2370	0	-	0	0	0	0	2
chr6	15724335	15739944	dre-circ-3872	0	+	0	3	0	0	0
chr6	16402664	16403034	dre-circ-3873	0	-	0	0	3	0	2
chr6	1707855	1708056	dre-circ-2364	0	+	2	0	0	0	0
chr6	1707855	1709708	dre-circ-3874	0	+	2	3	12	0	4
chr6	17663320	17670630	dre-circ-3875	0	+	4	0	8	0	0
chr6	1890211	1895151	dre-circ-3876	0	+	0	0	2	0	0
chr6	19040812	19047056	dre-circ-3877	0	+	0	0	6	0	0
chr6	19046399	19047056	dre-circ-3878	0	+	2	0	2	0	0
chr6	19516101	19520754	dre-circ-2371	0	+	0	0	2	0	0
chr6	1983930	1984797	dre-circ-3879	0	-	2	0	0	2	3
chr6	19842693	19845894	dre-circ-3880	0	+	0	0	0	0	2
chr6	2227543	2230541	dre-circ-3881	0	-	23	2	7	2	0
chr6	22468490	22471024	dre-circ-3882	0	-	2	0	7	31	0
chr6	22671183	22671881	dre-circ-3883	0	+	0	0	4	2	0
chr6	22800431	22806443	dre-circ-3884	0	+	0	0	15	2	0
chr6	2317201	2320815	dre-circ-3885	0	+	2	0	0	0	0
chr6	27870489	27881158	dre-circ-3886	0	+	2	0	0	0	2

chr6	27891395	27892010	dre-circ-3887	0	+	0	0	8	3	6
chr6	28396562	28400055	dre-circ-3888	0	+	2	0	0	0	0
chr6	29009363	29011363	dre-circ-3889	0	-	0	0	4	3	0
chr6	29847686	29853397	dre-circ-3890	0	-	0	4	0	0	0
chr6	30119707	30123022	dre-circ-3891	0	+	0	0	0	0	2
chr6	30453795	30459707	dre-circ-2372	0	-	0	0	0	0	2
chr6	3162888	3182228	dre-circ-2365	0	+	2	0	2	0	0
chr6	3174029	3182228	dre-circ-3892	0	+	2	0	0	0	0
chr6	3182042	3182228	dre-circ-3893	0	+	6	0	0	0	0
chr6	32028432	32029760	dre-circ-3894	0	+	0	0	3	0	0
chr6	33246561	33248630	dre-circ-3895	0	+	0	3	0	0	0
chr6	33511025	33512911	dre-circ-3896	0	-	2	0	0	0	0
chr6	37344119	37358480	dre-circ-3897	0	-	0	0	0	0	4
chr6	37365468	37368776	dre-circ-2373	0	-	0	0	0	3	0
chr6	37375768	37377643	dre-circ-2374	0	-	2	0	0	0	0
chr6	37460619	37465923	dre-circ-3898	0	-	2	0	0	0	0
chr6	37461220	37465923	dre-circ-3899	0	-	0	0	0	0	2
chr6	37705915	37706241	dre-circ-3900	0	+	0	0	0	2	0
chr6	39067035	39068837	dre-circ-3901	0	-	13	0	2	0	0
chr6	39070782	39075437	dre-circ-3902	0	-	2	0	0	0	0
chr6	40067999	40080340	dre-circ-2375	0	-	0	0	0	2	0
chr6	40080231	40080454	dre-circ-2376	0	-	2	0	0	0	0
chr6	40511827	40512257	dre-circ-3903	0	+	7	0	0	0	0
chr6	40861882	40869279	dre-circ-3904	0	+	0	0	0	0	3
chr6	41030674	41036008	dre-circ-2377	0	+	3	0	0	0	0
chr6	41422592	41424334	dre-circ-3905	0	+	4	0	18	16	0
chr6	4225179	4225454	dre-circ-3906	0	+	4	0	0	0	0
chr6	43945566	43946013	dre-circ-3907	0	-	6	0	2	0	0
chr6	45262603	45265503	dre-circ-3908	0	+	0	0	2	0	0
chr6	45274464	45363011	dre-circ-3909	0	+	0	0	0	0	4
chr6	45285728	45297930	dre-circ-3910	0	+	0	2	0	0	0
chr6	47432861	47433092	dre-circ-3911	0	-	2	0	0	0	0
chr6	47432861	47437310	dre-circ-2378	0	-	2	0	0	0	0
chr6	47437137	47437310	dre-circ-3912	0	-	166	0	38	9	7
chr6	49767953	49770495	dre-circ-2379	0	-	2	0	0	0	0
chr6	52262367	52269110	dre-circ-3913	0	+	0	0	3	0	0
chr6	53349851	53352290	dre-circ-2380	0	+	27	0	6	0	0
chr6	55283038	55283626	dre-circ-3914	0	-	0	0	2	0	0
chr6	55283038	55289121	dre-circ-3915	0	-	17	0	0	0	2
chr6	55584041	55589383	dre-circ-2381	0	-	0	2	0	0	0
chr6	56048470	56057673	dre-circ-3916	0	+	3	0	0	0	0
chr6	56055846	56057673	dre-circ-3917	0	+	4	0	0	0	0
chr6	59402287	59407048	dre-circ-3918	0	+	9	0	0	0	0
chr6	59406882	59407048	dre-circ-3919	0	+	3	0	0	0	0
chr6	59674611	59693920	dre-circ-3920	0	+	3	0	0	0	0
chr6	6859391	6864548	dre-circ-3921	0	+	0	0	4	0	0
chr6	7182597	7183202	dre-circ-2366	0	+	0	0	0	0	2
chr6	7271639	7271888	dre-circ-2367	0	+	2	0	0	0	0
chr6	7965796	7966246	dre-circ-3922	0	+	0	7	0	0	0
chr7	1087930	1088156	dre-circ-3923	0	-	6	0	0	0	0
chr7	11608325	11618700	dre-circ-3924	0	+	0	0	0	0	8
chr7	12110349	12113231	dre-circ-3925	0	+	0	0	3	2	0
chr7	14465141	14515259	dre-circ-3926	0	-	0	0	0	3	0
chr7	14553352	14563524	dre-circ-3927	0	-	0	0	2	0	2
chr7	14878384	14878697	dre-circ-3928	0	+	9	0	0	0	5
chr7	14992861	14993151	dre-circ-3929	0	+	4	0	0	0	0
chr7	16533008	16550235	dre-circ-3930	0	+	0	0	3	0	40
chr7	17313922	17314395	dre-circ-3931	0	+	16	0	4	0	2
chr7	18862676	18872693	dre-circ-2388	0	+	0	0	0	0	3
chr7	20573096	20574543	dre-circ-2389	0	-	4	0	0	0	0
chr7	21227328	21227839	dre-circ-3932	0	-	0	0	4	0	2
chr7	21275529	21282930	dre-circ-2390	0	+	0	0	3	0	0
chr7	21617700	21618537	dre-circ-3933	0	-	3	0	0	0	0
chr7	22026089	22028956	dre-circ-3934	0	+	0	0	3	0	0
chr7	22837017	22863364	dre-circ-3935	0	+	7	0	0	0	4
chr7	22954205	22954703	dre-circ-3936	0	-	0	0	3	0	0
chr7	23045136	23045599	dre-circ-3937	0	-	6	0	4	0	0
chr7	24847795	24850949	dre-circ-2391	0	+	0	0	0	0	2
chr7	24861148	24866599	dre-circ-3938	0	+	0	0	0	0	2
chr7	24894840	24895621	dre-circ-3939	0	-	0	0	21	0	0
chr7	24941155	24956861	dre-circ-3940	0	-	3	0	0	0	0
chr7	25094400	25116837	dre-circ-2392	0	-	0	0	2	0	0
chr7	25134532	25139567	dre-circ-3941	0	-	0	2	0	0	0
chr7	25239246	25240511	dre-circ-2393	0	+	0	0	2	0	0
chr7	25543289	25545576	dre-circ-3942	0	-	0	0	2	0	0
chr7	25544048	25545576	dre-circ-3943	0	-	2	2	2	2	2
chr7	25765779	25766314	dre-circ-3944	0	-	4	0	2	0	0
chr7	26331329	26338905	dre-circ-3945	0	+	2	0	0	0	0
chr7	26335609	26352715	dre-circ-3946	0	+	4	0	0	0	0
chr7	26349400	26352715	dre-circ-2394	0	+	2	0	0	0	0
chr7	26418542	26422343	dre-circ-3947	0	+	10	0	0	0	0
chr7	27717157	27722669	dre-circ-2395	0	-	2	0	0	0	0
chr7	28158829	28162426	dre-circ-3948	0	-	0	2	0	2	0
chr7	28346523	28347519	dre-circ-3949	0	+	0	0	2	0	0
chr7	28446335	28453512	dre-circ-3950	0	+	4	0	0	0	0
chr7	28838425	28841965	dre-circ-3951	0	-	0	0	2	0	0
chr7	28850689	28854079	dre-circ-3952	0	-	8	0	0	0	0

chr7	30632918	30633790	dre-circ-2396	0	+	0	0	0	2	0
chr7	31761777	31762488	dre-circ-3953	0	-	2	0	0	0	0
chr7	33137231	33137980	dre-circ-3954	0	-	3	0	0	0	0
chr7	33189043	33200137	dre-circ-2397	0	+	0	0	2	0	0
chr7	33195018	33200137	dre-circ-3955	0	+	0	0	5	0	0
chr7	33196906	33200137	dre-circ-3956	0	+	0	0	43	0	0
chr7	34488473	34509529	dre-circ-3957	0	+	0	0	0	0	2
chr7	35311993	35312329	dre-circ-3958	0	+	3	0	0	0	0
chr7	36199981	36202580	dre-circ-3959	0	+	0	0	3	0	0
chr7	36308606	36309052	dre-circ-3960	0	-	2	0	0	0	0
chr7	36320489	36323253	dre-circ-3961	0	-	5	0	0	0	0
chr7	37884612	37885018	dre-circ-3962	0	+	4	0	0	0	0
chr7	38353765	38356518	dre-circ-3963	0	+	0	0	3	0	0
chr7	38353765	38356920	dre-circ-3964	0	+	0	0	3	0	0
chr7	39933081	39949426	dre-circ-3965	0	-	2	0	0	0	0
chr7	40101393	40101823	dre-circ-3966	0	-	5	0	0	0	0
chr7	40807182	40819806	dre-circ-3967	0	+	0	0	6	0	0
chr7	40846753	40847337	dre-circ-3968	0	+	0	0	0	5	0
chr7	4145872	4152942	dre-circ-3969	0	-	8	0	0	0	0
chr7	42102830	42102991	dre-circ-3970	0	+	2	0	0	0	0
chr7	42371412	42377543	dre-circ-3971	0	+	0	0	2	0	0
chr7	43199145	43207961	dre-circ-3972	0	-	2	0	0	0	0
chr7	43239224	43256095	dre-circ-2398	0	-	3	0	0	0	0
chr7	43669749	43672283	dre-circ-3973	0	-	2	0	0	0	0
chr7	43967350	43971025	dre-circ-2399	0	+	2	0	0	0	0
chr7	44142370	44143556	dre-circ-3974	0	-	2	2	0	0	0
chr7	4508966	4511237	dre-circ-2383	0	+	0	0	0	0	2
chr7	50099090	50105552	dre-circ-3975	0	+	0	0	10	0	0
chr7	51698839	51699428	dre-circ-2400	0	-	2	0	2	0	0
chr7	52061577	52072761	dre-circ-3976	0	-	3	0	0	0	2
chr7	52107465	52108971	dre-circ-3977	0	-	0	0	3	0	0
chr7	54898538	54898944	dre-circ-3978	0	+	3	0	0	0	0
chr7	5602010	5786975	dre-circ-2384	0	+	0	0	0	0	2
chr7	56062442	56066048	dre-circ-2401	0	-	2	0	0	0	0
chr7	56095833	56114445	dre-circ-2402	0	-	2	0	0	0	2
chr7	56117916	56145496	dre-circ-2403	0	-	0	0	0	0	4
chr7	56619236	56619727	dre-circ-3979	0	-	5	0	0	0	0
chr7	57634197	57641511	dre-circ-3980	0	+	7	0	0	0	0
chr7	59109060	59119234	dre-circ-3981	0	+	0	0	0	2	0
chr7	59197596	59208991	dre-circ-3982	0	-	0	0	10	0	0
chr7	59197596	59208994	dre-circ-3983	0	-	0	0	23	0	0
chr7	59204584	59208991	dre-circ-3984	0	-	0	0	32	0	0
chr7	59204584	59208994	dre-circ-3985	0	-	0	0	60	0	0
chr7	60062175	60062690	dre-circ-3986	0	-	8	3	2	0	0
chr7	60764089	60764407	dre-circ-3987	0	-	0	0	3	0	5
chr7	60891163	60891521	dre-circ-2404	0	+	0	0	0	0	2
chr7	6520629	6546631	dre-circ-3988	0	+	0	0	0	0	4
chr7	65638586	65638896	dre-circ-3989	0	-	3	0	0	0	0
chr7	67220217	67220469	dre-circ-3990	0	-	0	0	0	3	0
chr7	68546859	68554318	dre-circ-2405	0	-	0	0	2	0	0
chr7	6933978	7088000	dre-circ-2385	0	-	0	0	0	0	2
chr7	71040806	71041429	dre-circ-2406	0	+	2	0	0	0	0
chr7	71040806	71065717	dre-circ-3991	0	+	0	0	6	0	0
chr7	72198002	72201641	dre-circ-2407	0	+	2	0	0	0	0
chr7	7284086	7284300	dre-circ-2386	0	-	2	0	0	0	0
chr7	7284086	7284315	dre-circ-2387	0	-	2	0	0	0	0
chr7	72889012	72896254	dre-circ-3992	0	+	0	0	2	0	0
chr7	730242	730938	dre-circ-3993	0	+	3	0	0	0	0
chr7	73982175	73983344	dre-circ-3994	0	-	0	0	2	0	0
chr7	74364252	74375145	dre-circ-3995	0	-	0	0	2	0	0
chr7	74386907	74390346	dre-circ-3996	0	-	0	0	13	3	0
chr7	74560232	74564243	dre-circ-3997	0	+	0	0	8	0	0
chr7	74741339	74744628	dre-circ-3998	0	+	2	4	0	0	0
chr7	74854856	74855489	dre-circ-2408	0	+	2	0	0	0	0
chr7	75139930	75142405	dre-circ-3999	0	-	0	0	2	0	0
chr7	75170032	75170375	dre-circ-4000	0	-	12	0	0	0	0
chr7	75212330	75213373	dre-circ-4001	0	-	4	0	0	0	0
chr7	75282430	75284919	dre-circ-4002	0	+	4	0	0	0	2
chr7	75474848	75483304	dre-circ-2409	0	+	3	0	0	0	0
chr7	76043262	76048454	dre-circ-2410	0	+	0	2	0	0	0
chr7	76137832	76167440	dre-circ-4003	0	+	0	0	0	0	5
chr7	76185714	76218606	dre-circ-4004	0	+	2	0	0	0	5
chr7	76187278	76188753	dre-circ-4005	0	+	2	0	0	0	6
chr7	76712554	76716324	dre-circ-4006	0	-	0	0	0	0	2
chr7	76723975	76725764	dre-circ-4007	0	-	0	0	5	8	0
chr7	76783197	76784149	dre-circ-4008	0	+	0	3	0	0	0
chr7	827273	844065	dre-circ-2382	0	+	0	0	2	0	0
chr7	8740273	8785433	dre-circ-4009	0	-	0	0	0	0	15
chr7	9102484	9103338	dre-circ-4010	0	-	3	0	0	0	0
chr8	10748687	10935376	dre-circ-2413	0	-	3	0	0	0	0
chr8	10748687	10939452	dre-circ-2414	0	-	2	0	0	0	0
chr8	11353326	11353524	dre-circ-4011	0	-	0	0	2	0	2
chr8	11678714	11687728	dre-circ-4012	0	+	0	2	19	0	4
chr8	12187596	12190762	dre-circ-4013	0	+	2	0	0	0	0
chr8	12191944	12192180	dre-circ-4014	0	+	2	0	0	0	0
chr8	12254187	12257801	dre-circ-2415	0	+	0	0	3	0	0
chr8	12348719	12350302	dre-circ-4015	0	-	0	6	3	0	2

chr8	14680561	14687465	dre-circ-2416	0	+	0	0	3	0	0
chr8	17038900	17039229	dre-circ-4016	0	-	4	0	2	0	0
chr8	17930277	17948229	dre-circ-2417	0	-	0	0	2	0	0
chr8	18491223	18494371	dre-circ-4017	0	-	0	0	2	0	0
chr8	19051381	19052330	dre-circ-4018	0	-	0	0	30	0	0
chr8	1908283	1909186	dre-circ-4019	0	-	0	0	0	3	0
chr8	19560068	19576840	dre-circ-2418	0	-	0	0	2	0	0
chr8	19783651	19786181	dre-circ-4020	0	-	14	0	4	4	4
chr8	2031084	2034228	dre-circ-4021	0	+	6	0	2	0	0
chr8	21064595	21084454	dre-circ-4022	0	+	0	2	0	0	0
chr8	21293181	21306365	dre-circ-2419	0	+	2	0	0	0	0
chr8	21511078	21552233	dre-circ-2420	0	+	3	0	0	0	4
chr8	21537282	21561745	dre-circ-4023	0	+	0	0	0	0	2
chr8	21592811	21595031	dre-circ-4024	0	-	0	0	2	0	0
chr8	21661523	21662058	dre-circ-4025	0	+	2	4	2	0	0
chr8	21909187	21917847	dre-circ-2421	0	+	2	0	0	0	0
chr8	23014204	23057808	dre-circ-2422	0	+	0	0	2	0	0
chr8	23290225	23309535	dre-circ-2423	0	+	2	0	0	0	0
chr8	23659886	23679949	dre-circ-4026	0	-	0	0	0	0	2
chr8	24533791	24548157	dre-circ-4027	0	-	0	0	2	0	0
chr8	24593719	24595891	dre-circ-2424	0	+	2	0	0	0	0
chr8	25166148	25287391	dre-circ-2425	0	+	2	0	0	0	0
chr8	25934684	25935998	dre-circ-4028	0	+	0	0	3	2	0
chr8	26918873	26919419	dre-circ-2426	0	+	3	0	0	0	0
chr8	27208468	27210787	dre-circ-2427	0	+	6	0	0	0	0
chr8	27208468	27214520	dre-circ-4029	0	+	3	0	0	0	0
chr8	27208468	27216840	dre-circ-2428	0	+	6	0	0	0	0
chr8	27458091	27462201	dre-circ-2429	0	-	0	0	2	0	0
chr8	2775588	2786622	dre-circ-4030	0	+	3	0	0	0	0
chr8	28404210	28405271	dre-circ-2430	0	-	0	0	2	0	0
chr8	2895090	2895400	dre-circ-2412	0	+	0	0	3	0	0
chr8	32926851	32929273	dre-circ-4031	0	-	0	0	2	0	0
chr8	33291006	33305131	dre-circ-4032	0	-	5	0	0	0	0
chr8	33294110	33308628	dre-circ-2431	0	-	0	0	0	0	7
chr8	33294781	33309929	dre-circ-4033	0	-	0	0	0	0	9
chr8	33294781	33311044	dre-circ-2432	0	-	0	0	0	0	4
chr8	33305774	33311044	dre-circ-2433	0	-	0	0	0	0	3
chr8	33306040	33310210	dre-circ-2434	0	-	0	0	0	0	4
chr8	33310616	33311044	dre-circ-2435	0	-	0	0	0	0	2
chr8	36691760	36811991	dre-circ-2436	0	+	0	3	0	0	0
chr8	37469811	37474185	dre-circ-4034	0	+	0	0	0	0	7
chr8	43360892	43367867	dre-circ-4035	0	+	0	0	2	0	0
chr8	43874710	43874906	dre-circ-4036	0	-	0	0	0	0	2
chr8	44619370	44620876	dre-circ-2437	0	-	3	0	0	0	0
chr8	45898932	45904256	dre-circ-4037	0	+	0	0	0	0	2
chr8	46563896	46584721	dre-circ-4038	0	-	0	0	7	0	0
chr8	47223751	47224584	dre-circ-4039	0	-	3	0	3	2	0
chr8	4763617	4764678	dre-circ-4040	0	-	0	0	2	0	0
chr8	48119328	48127017	dre-circ-4041	0	-	0	0	2	0	0
chr8	48556230	48560098	dre-circ-4042	0	+	0	0	0	0	3
chr8	48601122	48653364	dre-circ-4043	0	-	0	0	0	0	8
chr8	52683401	52687391	dre-circ-4044	0	+	0	0	2	2	0
chr8	54568160	54575477	dre-circ-2438	0	+	0	0	3	0	0
chr8	55313597	55314726	dre-circ-4045	0	+	0	0	0	0	6
chr8	55371422	55377091	dre-circ-4046	0	-	4	0	0	0	2
chr8	6091	171235	dre-circ-2411	0	-	0	0	2	0	0
chr8	6374982	6433723	dre-circ-4047	0	-	0	7	0	0	3
chr8	7614690	7615615	dre-circ-4048	0	+	0	0	0	7	0
chr8	9449655	9459840	dre-circ-4049	0	+	2	0	0	0	0
chr8	9637117	9638580	dre-circ-4050	0	-	0	3	0	0	0
chr9	1069789	1070013	dre-circ-4051	0	-	14	0	0	0	0
chr9	10930282	10934681	dre-circ-4052	0	+	15	0	0	0	0
chr9	14718483	14722726	dre-circ-4053	0	-	4	0	0	3	0
chr9	1525097	1527118	dre-circ-2444	0	+	0	2	0	0	0
chr9	16066	25690	dre-circ-2439	0	+	0	2	0	0	0
chr9	16607972	16650537	dre-circ-4054	0	+	0	0	4	0	3
chr9	1662693	1663038	dre-circ-4055	0	+	5	0	0	0	0
chr9	17689537	17694798	dre-circ-4056	0	-	2	0	0	0	0
chr9	18804996	18805569	dre-circ-4057	0	-	2	0	0	0	0
chr9	2018195	2027229	dre-circ-2445	0	+	0	0	2	0	0
chr9	2026489	2027229	dre-circ-2446	0	+	2	0	0	0	0
chr9	20989	23473	dre-circ-2440	0	+	0	0	0	2	5
chr9	22937927	22940251	dre-circ-2455	0	-	0	0	0	2	0
chr9	23300	23473	dre-circ-2441	0	+	0	0	0	3	0
chr9	23883713	23887169	dre-circ-4058	0	-	2	0	0	0	0
chr9	23939481	23939805	dre-circ-4059	0	-	30	0	3	0	0
chr9	2424572	2578314	dre-circ-2447	0	-	0	9	0	0	24
chr9	2456461	2460421	dre-circ-2448	0	-	2	0	0	0	0
chr9	25138541	25140617	dre-circ-2456	0	+	0	0	0	2	0
chr9	26156693	26161567	dre-circ-2457	0	+	0	0	2	0	0
chr9	26187345	26188315	dre-circ-4060	0	+	0	0	3	0	2
chr9	26359710	26359880	dre-circ-4061	0	-	21	0	0	0	2
chr9	28913340	28913669	dre-circ-4062	0	+	5	0	0	0	0
chr9	29301724	29304511	dre-circ-4063	0	-	0	0	2	0	0
chr9	3086397	3088676	dre-circ-4064	0	+	0	0	2	0	17
chr9	31262325	31263035	dre-circ-2458	0	-	4	0	0	0	0
chr9	31791264	31798224	dre-circ-4065	0	+	0	4	0	2	0

chr9	32075886	32088374	dre-circ-4066	0	+	0	0	2	0	0
chr9	34403322	34415076	dre-circ-4067	0	-	0	0	3	0	0
chr9	34784638	34785197	dre-circ-2459	0	+	2	0	0	0	0
chr9	35175650	35176876	dre-circ-4068	0	-	5	0	5	2	0
chr9	35879756	35883253	dre-circ-4069	0	-	0	2	0	0	0
chr9	35989006	35991369	dre-circ-4070	0	+	3	0	4	5	0
chr9	38397453	38397627	dre-circ-4071	0	+	0	0	0	0	2
chr9	3970521	3999262	dre-circ-2449	0	-	0	0	0	0	2
chr9	4015268	4019583	dre-circ-4072	0	-	3	0	0	0	0
chr9	4052962	4055399	dre-circ-4073	0	-	0	0	8	0	0
chr9	4087286	4218919	dre-circ-2450	0	+	0	0	16	0	5
chr9	4104875	4108984	dre-circ-4074	0	+	0	0	0	0	2
chr9	418220	419619	dre-circ-2442	0	+	0	0	0	0	2
chr9	4208328	4211063	dre-circ-2451	0	+	0	0	2	0	0
chr9	42383409	42396789	dre-circ-4075	0	-	2	0	2	2	0
chr9	42386504	42396789	dre-circ-4076	0	-	0	0	0	2	0
chr9	42784290	42799204	dre-circ-2460	0	+	3	0	0	0	0
chr9	4385737	4407231	dre-circ-2452	0	+	0	0	2	4	0
chr9	43891347	44054387	dre-circ-2461	0	-	0	0	10	0	0
chr9	43891948	44056628	dre-circ-2462	0	-	6	0	0	0	0
chr9	43899949	43900107	dre-circ-2463	0	-	0	0	0	2	0
chr9	43928996	44119375	dre-circ-2464	0	-	4	0	0	0	0
chr9	44180265	44180927	dre-circ-4077	0	-	0	0	5	2	0
chr9	44872872	44874062	dre-circ-4078	0	+	2	0	0	0	0
chr9	453892	454300	dre-circ-4079	0	+	2	0	0	0	0
chr9	45571417	45577735	dre-circ-4080	0	-	0	0	4	0	0
chr9	46701340	46705474	dre-circ-2465	0	+	0	0	0	0	2
chr9	47064793	47065393	dre-circ-4081	0	-	15	0	4	0	2
chr9	4802098	4810037	dre-circ-2453	0	-	0	0	3	0	0
chr9	48645068	48645830	dre-circ-4082	0	-	0	0	0	0	4
chr9	50853213	50853447	dre-circ-4083	0	-	0	0	0	0	2
chr9	51056832	51057211	dre-circ-4084	0	+	12	0	0	0	0
chr9	51472899	51476130	dre-circ-4085	0	+	0	0	3	0	0
chr9	52889603	52908445	dre-circ-4086	0	-	0	0	2	0	0
chr9	531714	532097	dre-circ-2443	0	+	5	0	0	0	0
chr9	53975572	54145590	dre-circ-2466	0	+	0	20	0	0	163
chr9	54809033	54809376	dre-circ-4087	0	-	8	0	0	0	0
chr9	55311462	55324784	dre-circ-4088	0	-	0	0	0	0	2
chr9	56198191	56199048	dre-circ-2467	0	+	2	0	3	3	0
chr9	56415912	56416403	dre-circ-4089	0	-	0	0	0	2	0
chr9	56725952	56733379	dre-circ-4090	0	+	2	0	0	0	0
chr9	56739105	56745358	dre-circ-4091	0	+	2	0	0	0	6
chr9	58001623	58001850	dre-circ-4092	0	-	6	0	0	0	0
chr9	58110797	58179977	dre-circ-4093	0	-	143	13	113	77	0
chr9	5942607	5945765	dre-circ-2454	0	-	0	3	0	0	0
chr9	597804	598360	dre-circ-4094	0	-	0	0	2	0	0
chr9	6142779	6150315	dre-circ-4095	0	+	0	0	2	0	0
chr9	6324141	6336945	dre-circ-4096	0	+	3	0	2	0	0
chr9	786634	790254	dre-circ-4097	0	-	5	0	0	0	0
chr9	912347	917309	dre-circ-4098	0	-	0	0	8	0	0
chr9	9431200	9431655	dre-circ-4099	0	-	0	0	0	2	0
Zv9_NA	4162	4733	dre-circ-4100	0	+	15	2	11	6	6
Zv9_NA	6842	8395	dre-circ-2885	0	+	0	0	0	2	0
Zv9_NA	732	2873	dre-circ-4101	0	+	0	4	5	2	8
Zv9_NA	73	466	dre-circ-4102	0	+	2	0	0	0	0
Zv9_NA	14179	15693	dre-circ-4103	0	-	3	0	0	0	12
Zv9_NA	11388	12044	dre-circ-2886	0	-	2	0	0	0	0
Zv9_NA	5783	8164	dre-circ-4104	0	+	9	0	0	5	7
Zv9_NA	49575	49820	dre-circ-4105	0	-	0	0	0	0	2
Zv9_NA	13047	14772	dre-circ-4106	0	-	5	0	0	0	0
Zv9_NA	4852	13221	dre-circ-4107	0	-	6	0	0	0	0
Zv9_NA	48864	58142	dre-circ-4108	0	+	0	0	0	4	0
Zv9_NA	15984	16405	dre-circ-4109	0	-	0	0	5	0	8
Zv9_NA	3657	4045	dre-circ-4110	0	-	0	0	4	0	0
Zv9_NA	417	803	dre-circ-4111	0	-	0	0	0	0	4
Zv9_NA	15448	20168	dre-circ-2887	0	-	7	0	0	0	0
Zv9_NA	53081	53532	dre-circ-2888	0	+	0	0	3	0	0
Zv9_NA	37454	57376	dre-circ-4112	0	+	0	0	0	8	0
Zv9_NA	29084	45855	dre-circ-2890	0	+	0	0	0	0	2
Zv9_NA	870	40573	dre-circ-2889	0	+	0	0	0	0	2
Zv9_NA	4370	22871	dre-circ-4113	0	-	6	0	5	0	10
Zv9_NA	4238	4654	dre-circ-2891	0	+	0	0	2	0	0
Zv9_NA	655	1833	dre-circ-2892	0	-	0	0	0	0	7
Zv9_NA	36062	60974	dre-circ-4114	0	-	3	0	0	0	0
Zv9_NA	15069	16958	dre-circ-2893	0	+	0	0	2	0	0
Zv9_NA	136339	141568	dre-circ-2895	0	-	0	0	0	0	2
Zv9_NA	157490	160863	dre-circ-4115	0	+	2	0	0	0	0
Zv9_NA	73558	77246	dre-circ-4116	0	-	6	0	2	0	0
Zv9_NA	79950	81805	dre-circ-2894	0	-	2	0	0	0	0
Zv9_NA	139093	139765	dre-circ-2896	0	+	0	0	0	7	0
Zv9_sc	151516	155713	dre-circ-4117	0	+	0	0	0	0	5
Zv9_sc	193048	197883	dre-circ-2897	0	+	0	0	0	0	11
Zv9_sc	45252	68667	dre-circ-2898	0	+	0	0	3	0	0
Zv9_sc	61378	71914	dre-circ-4118	0	+	2	0	0	0	0
Zv9_sc	73336	73641	dre-circ-4119	0	+	0	0	0	0	6
Zv9_sc	105372	108364	dre-circ-4120	0	+	2	0	0	0	0
Zv9_sc	21030	57201	dre-circ-4121	0	-	0	0	0	0	2

Zv9_sc	91725	95344	dre-circ-4122	0	-	0	2	0	0	0
Zv9_sc	71093	72902	dre-circ-2899	0	-	4	0	3	0	2
Zv9_sc	37980	38644	dre-circ-2900	0	+	0	0	0	0	2
Zv9_sc	81432	98819	dre-circ-2901	0	-	0	0	0	5	0
Zv9_sc	165026	166329	dre-circ-4123	0	-	0	0	3	0	0
Zv9_sc	15110	17650	dre-circ-4124	0	-	0	0	0	2	0
Zv9_sc	202226	306487	dre-circ-2902	0	-	0	0	0	0	4
Zv9_sc	27008	34918	dre-circ-4125	0	+	0	0	2	0	0
Zv9_sc	142596	165500	dre-circ-2903	0	-	2	0	0	8	0

Supplementary table 7:1222 common elements in "FindCirc" and "CIRI":

chr1	10844764	10845317	+	chr17	41042269	41043332	+	chr25	29151716	29154430	+
chr1	11049377	11049681	-	chr17	41801318	41803794	-	chr25	29746028	29750831	+
chr1	11541954	11543990	+	chr17	4242464	4245829	+	chr25	31456721	31460189	+
chr1	13359476	13361152	+	chr17	44013086	44015823	+	chr25	32197516	32200842	+
chr1	14185460	14186413	-	chr17	44914120	44915511	+	chr25	33750736	33754258	+
chr1	1479892	1551748	+	chr17	45549785	45550392	-	chr25	3563619	3566306	-
chr1	1568094	1602122	+	chr17	49082463	49085000	-	chr25	36708670	36710042	-
chr1	1569054	1603092	+	chr17	50726939	50731619	-	chr25	36763508	36763974	+
chr1	16501794	16512429	-	chr17	5222713	5227962	+	chr25	4035486	4036189	-
chr1	1654583	1661319	+	chr17	6339490	6340874	+	chr25	6006308	6008481	+
chr1	19652203	19655654	-	chr17	6440831	6441792	+	chr25	704873	705733	+
chr1	2186341	2190351	+	chr17	7937362	7945236	-	chr25	8573258	8624060	+
chr1	24743837	24746774	-	chr17	8065756	8066468	+	chr25	8574885	8624766	+
chr1	26794590	26795743	+	chr17	8194337	8207115	+	chr25	948312	1036714	+
chr1	27205361	27223825	+	chr17	8594505	8594882	-	chr3	11505502	11516635	-
chr1	27482660	27488891	-	chr17	957037	961856	+	chr3	13433829	13434071	-
chr1	27487902	27488891	-	chr18	10281304	10282090	+	chr3	14043373	14065711	+
chr1	27718742	27740044	-	chr18	10320182	10325624	+	chr3	15327149	15327636	-
chr1	27737431	27740044	-	chr18	14155586	14155912	+	chr3	15550030	15556678	+
chr1	27737431	27741348	-	chr18	14284358	14286889	+	chr3	15550030	15562572	+
chr1	296439	296829	+	chr18	15794940	15797317	+	chr3	16503081	16503449	+
chr1	35290920	35295668	+	chr18	16707203	16710688	-	chr3	16576082	16576963	+
chr1	354482	354695	-	chr18	17335187	17338070	+	chr3	16929020	16933501	+
chr1	40708359	40708786	+	chr18	20472483	20472674	+	chr3	16936126	16936871	+
chr1	40802877	40804460	-	chr18	20651844	20652348	+	chr3	17337153	17339667	+
chr1	41611277	41611918	-	chr18	20651844	20655924	+	chr3	18993897	18994239	-
chr1	44705091	44707597	+	chr18	22832508	22832819	+	chr3	21202194	21202553	-
chr1	45331768	45350377	+	chr18	25380206	25385792	-	chr3	22292280	22293519	+
chr1	45705443	45707015	+	chr18	26680090	26682538	+	chr3	22700560	22703370	+
chr1	46101877	46111430	-	chr18	27931188	27933114	+	chr3	23485672	23504198	+
chr1	46155652	46160692	+	chr18	300788	355125	-	chr3	23625625	23626332	-
chr1	4752008	4752191	-	chr18	31039547	31045181	+	chr3	24148581	24152178	-
chr1	5010139	5010513	+	chr18	31040529	31045181	+	chr3	26120386	26123254	+
chr1	5013514	5018702	+	chr18	33241140	33245276	+	chr3	29492619	29493072	+
chr1	5066893	5070720	+	chr18	35649600	35653017	-	chr3	30456064	30456446	+
chr1	51158825	51159210	+	chr18	36275898	36285500	-	chr3	31895873	31913184	-
chr1	51841979	51849108	+	chr18	36303289	36316518	-	chr3	32330048	32333591	-
chr1	52543032	52550796	+	chr18	37780181	37782190	+	chr3	34796290	34799810	-
chr1	53086613	53087458	+	chr18	39646077	39646914	+	chr3	34810102	34828938	-
chr1	53182808	53183055	+	chr18	39860552	39864703	+	chr3	38668312	38668904	-
chr1	54177551	54179856	-	chr18	42984845	42985335	+	chr3	39133694	39142852	-
chr1	54677664	54746137	-	chr18	4531600	4618096	+	chr3	39146968	39147444	-
chr1	55734525	55738556	-	chr18	47010208	47012743	+	chr3	3929091	3955869	-
chr1	56777740	56821524	+	chr18	47449809	47450219	-	chr3	40610670	40613234	-
chr1	56792880	56837841	+	chr18	47463906	47468820	-	chr3	41277434	41287055	-
chr1	56803164	56854119	+	chr18	47878629	47879031	-	chr3	42087941	42088359	+
chr1	56810667	56864787	+	chr18	47899284	47906977	-	chr3	42552364	42599390	+
chr1	58482940	58489148	+	chr18	49470012	49514441	-	chr3	42552614	42599644	+
chr1	59699163	59701702	-	chr18	49539771	49540310	-	chr3	42559919	42602876	+
chr1	59699163	59704355	-	chr18	5361612	5361945	+	chr3	49369628	49375216	+
chr1	7777574	7778068	+	chr18	6780203	6782687	+	chr3	50572070	50572527	+
chr1	7983134	7992924	-	chr18	7012238	7019214	+	chr3	50572126	50572527	+
chr1	8588328	8588815	-	chr18	7012499	7023345	+	chr3	5125570	5149041	-
chr10	11140240	11149265	-	chr18	8436276	8447916	-	chr3	55306548	55307709	-
chr10	15004126	15004979	+	chr19	11187155	11190982	+	chr3	55618936	55619569	+
chr10	1511576	1547359	-	chr19	11319765	11324995	-	chr3	55643227	55662357	+
chr10	15540461	15553852	+	chr19	11849228	11849649	+	chr3	59458356	59488680	-
chr10	16045927	16046265	-	chr19	1497503	1498222	-	chr3	60965082	60975293	-
chr10	17506357	17544065	-	chr19	18130967	18170930	-	chr3	62740522	62741958	-
chr10	1800922	1802120	-	chr19	19532917	19552577	-	chr3	63181933	63182180	-
chr10	20121530	20122876	+	chr19	19692509	19695154	-	chr3	7477887	7538980	-
chr10	20168681	20170333	+	chr19	19733451	19741187	-	chr3	8250853	8251177	+
chr10	20171698	20178455	+	chr19	19897264	19905483	-	chr3	9997741	9998387	+
chr10	208563	208867	+	chr19	20526987	20527359	+	chr4	10676784	10692897	+
chr10	22024642	22030670	+	chr19	2233014	2246371	-	chr4	11938923	11947736	-

chr10	22718160	22718553	+	chr19	22946830	22959693	-	chr4	15007467	15009264	+
chr10	22943543	22943852	+	chr19	23274599	23275198	-	chr4	170959	171206	+
chr10	23773037	23778381	+	chr19	28008431	28008964	-	chr4	19307946	19311859	+
chr10	24899994	24902502	+	chr19	28168715	28173230	-	chr4	20274583	20277712	-
chr10	26429665	26430759	-	chr19	30836573	30841758	+	chr4	20274583	20305210	-
chr10	32043776	32044422	-	chr19	3104574	3105842	+	chr4	20475324	20480457	-
chr10	3388335	3389332	-	chr19	31118140	31121859	-	chr4	24743116	24768168	+
chr10	34492837	34494944	+	chr19	32889607	32900661	-	chr4	24832710	24833713	+
chr10	38716170	38725814	+	chr19	36708008	36710480	-	chr4	4790785	4792456	+
chr10	39602143	39602496	-	chr19	36753642	36770300	-	chr4	59223506	59225347	-
chr10	42478785	42482305	-	chr19	39060419	39060828	+	chr4	59352601	59397220	-
chr10	433087	435786	+	chr19	39065656	39069860	+	chr4	59511018	59516239	-
chr10	44253001	44260384	-	chr19	41049368	41053177	+	chr4	60581972	60588454	+
chr10	45861227	45879431	+	chr19	41088676	41089826	+	chr4	9606345	9618122	+
chr10	46039070	46039291	-	chr19	41088676	41097466	+	chr4	9837518	9852062	-
chr10	46154263	46156675	-	chr19	41143189	41143977	-	chr5	1354893	1357876	-
chr10	46507852	46508573	-	chr19	41863724	41863995	-	chr5	13940821	13941291	-
chr10	5969087	5984626	-	chr19	43939253	43940947	-	chr5	14909369	14909512	-
chr10	66259	114706	-	chr19	44158500	44165377	-	chr5	1925227	1927714	-
chr10	6885588	6886001	-	chr19	44165285	44173968	-	chr5	22947265	22949744	+
chr10	7297129	7312041	-	chr19	44169266	44174695	-	chr5	24054479	24066826	+
chr10	8372116	8376059	+	chr19	44657598	44659723	+	chr5	24054479	24084624	+
chr10	8900131	8903278	+	chr19	45183326	45186894	-	chr5	24084457	24096308	+
chr10	8948531	8953249	+	chr19	45288786	45294179	+	chr5	24108021	24111139	+
chr10	9042427	9043140	-	chr19	47910449	47911560	+	chr5	24770399	24772137	+
chr10	9127856	9139334	-	chr19	49204530	49214333	+	chr5	24885317	24885931	+
chr11	11330969	11331357	+	chr19	49852759	49858637	-	chr5	25850177	25850689	+
chr11	12304769	12305366	+	chr19	49855247	49859993	-	chr5	26543218	26547907	-
chr11	135098	138219	-	chr19	49992767	49998286	+	chr5	27220772	27223949	-
chr11	13679027	13680194	+	chr19	5275533	5277421	+	chr5	27967224	27979135	-
chr11	14941989	14942566	+	chr19	5528055	5529127	+	chr5	27967224	28014201	-
chr11	1739229	1740100	+	chr19	589624	590052	-	chr5	27974703	28014201	-
chr11	19496680	19505053	+	chr19	5992232	6003348	-	chr5	28000448	28014201	-
chr11	2073425	2076064	-	chr19	7602965	7605482	-	chr5	2826818	2828902	+
chr11	21991125	21993134	-	chr19	7665718	7692951	-	chr5	28443218	28443803	+
chr11	23128534	23128859	-	chr19	8423330	8423562	+	chr5	30513739	30516617	-
chr11	2427215	2432085	-	chr19	8471684	8533368	+	chr5	32091056	32095267	-
chr11	24299391	24309605	-	chr19	8508241	8545219	+	chr5	33851010	33866897	+
chr11	24356088	24356888	-	chr19	8508573	8545434	+	chr5	33854480	33870577	+
chr11	2487474	2491431	+	chr19	9698032	9700100	+	chr5	34102009	34104766	-
chr11	25312865	25314090	-	chr19	9742331	9746122	+	chr5	35190903	35192885	-
chr11	26119848	26120513	-	chr2	10335021	10335425	-	chr5	3914663	3915366	+
chr11	26220213	26220774	+	chr2	116709	117898	+	chr5	39921264	39928095	-
chr11	26628964	26633075	+	chr2	12934593	12936363	+	chr5	40618764	40620228	+
chr11	2787671	2788788	+	chr2	13459937	13461322	+	chr5	43206745	43207406	-
chr11	30637282	30641506	-	chr2	13459937	13465243	+	chr5	43451323	43454582	-
chr11	31312549	31320425	-	chr2	13542865	13543279	+	chr5	43451577	43458170	-
chr11	31420528	31425688	-	chr2	13950764	13956256	-	chr5	43509901	43510269	-
chr11	31420528	31438547	-	chr2	16077678	16094663	-	chr5	43528886	43553265	-
chr11	31425561	31438547	-	chr2	16083258	16094663	-	chr5	43540922	43546037	-
chr11	31475039	31476893	-	chr2	17138966	17140726	-	chr5	43540922	43553262	-
chr11	3388137	3388760	+	chr2	17138966	17175331	-	chr5	43540922	43553265	-
chr11	37206138	37208602	+	chr2	181406	182065	-	chr5	43545975	43553262	-
chr11	38124313	38124625	+	chr2	22524283	22524616	+	chr5	43545975	43553265	-
chr11	40324162	40326672	-	chr2	2335116	2335256	-	chr5	43550730	43553265	-
chr11	40368695	40372716	+	chr2	23582807	23599524	+	chr5	47743546	47743933	+
chr11	41543463	41548285	+	chr2	23587175	23587483	+	chr5	54886169	54886809	+
chr11	41543463	41548290	+	chr2	23588247	23605696	+	chr5	55638224	55644350	-
chr11	41629200	41630307	+	chr2	24103701	24104096	+	chr5	58344091	58346373	-
chr11	41629203	41637174	+	chr2	24492366	24499453	+	chr5	58500893	58501424	-
chr11	41639197	41644435	+	chr2	25393327	25395978	+	chr5	58786699	58790111	+
chr11	41917042	41917358	-	chr2	25725897	25734115	+	chr5	59104513	59104984	+
chr11	42055259	42055703	+	chr2	27790979	27795006	-	chr5	60943911	60946160	+
chr11	43669861	43671901	+	chr2	29003877	29012445	+	chr5	6126002	6129825	-
chr11	44248477	44318739	+	chr2	3074400	3078057	+	chr5	63698808	63699038	+
chr11	44248480	44318739	+	chr2	31631075	31637237	-	chr5	64438503	64442866	+

chr11	44622744	44634495	-	chr2	33765297	33765837	-	chr5	65374229	65374490	-
chr11	44624723	44634495	-	chr2	3512879	3513082	-	chr5	65693416	65693677	+
chr11	44629761	44634495	-	chr2	37095842	37096175	-	chr5	66514861	66531946	+
chr11	46055933	46058686	-	chr2	37178925	37179206	-	chr5	66716866	66719466	+
chr11	5579535	5580813	+	chr2	37408796	37420369	-	chr5	66735550	66737099	+
chr11	5892218	5893901	-	chr2	37943701	37945195	-	chr5	67507928	67523993	-
chr11	7534717	7539338	-	chr2	42218842	42226283	-	chr5	67526886	67542921	-
chr11	9049625	9129009	-	chr2	42528931	42533425	-	chr5	68679840	68680186	-
chr12	1246351	1247401	-	chr2	42555860	42560763	-	chr5	68707103	68713097	-
chr12	15050914	15051334	+	chr2	43287990	43292583	-	chr5	69227613	69227963	-
chr12	15193434	15197664	+	chr2	43635255	43635545	+	chr5	70272908	70275222	-
chr12	16514288	16516418	+	chr2	43966086	43967197	+	chr5	70336724	70336999	-
chr12	16514288	16516876	+	chr2	43976623	44009891	+	chr5	70565197	70565386	-
chr12	16514288	16522808	+	chr2	44664536	44665722	+	chr5	71811845	71816733	-
chr12	18240160	18240666	-	chr2	44673398	44673601	+	chr5	71884825	71885036	-
chr12	1867718	1905611	+	chr2	45030656	45031191	-	chr5	71899332	71900972	-
chr12	18774038	18778978	-	chr2	4559345	4561050	-	chr5	71913199	71914524	-
chr12	20072076	20072427	+	chr2	47877104	47877646	+	chr5	72023715	72027118	-
chr12	20244945	20245742	-	chr2	47938439	47943301	+	chr5	72304100	72379006	-
chr12	20540365	20543362	+	chr2	4818654	4819557	-	chr5	72426330	72427992	-
chr12	206966	210971	+	chr2	49346854	49358313	-	chr5	7363549	7368129	+
chr12	24219633	24220013	+	chr2	5010315	5013996	+	chr5	75452412	75455412	+
chr12	25315405	25347872	+	chr2	51667212	51670594	+	chr5	8761075	8842843	-
chr12	25555979	25556367	+	chr2	51760776	51769133	-	chr5	9413340	9415655	+
chr12	28646270	28647111	+	chr2	52481253	52490381	+	chr6	11708734	11711368	-
chr12	289587	290255	-	chr2	5352083	5357962	-	chr6	15724335	15739944	+
chr12	29600011	29613461	+	chr2	5354039	5357962	-	chr6	16402664	16403034	-
chr12	29600011	29620128	+	chr2	53830581	53838763	-	chr6	1707855	1709708	+
chr12	29632146	29632592	+	chr2	54228300	54228574	+	chr6	17663320	17670630	+
chr12	29881440	29888311	-	chr2	54243838	54244171	+	chr6	1890211	1895151	+
chr12	30425956	30426595	-	chr2	54861718	54878541	+	chr6	19040812	19047056	+
chr12	31738441	31738827	+	chr2	55398718	55479330	-	chr6	19046399	19047056	+
chr12	35729358	35729547	-	chr2	5558342	5561773	+	chr6	1983930	1984797	-
chr12	36463861	36464982	-	chr2	57091142	57091352	+	chr6	19842693	19845894	+
chr12	37323575	37326559	-	chr2	57410233	57410727	-	chr6	2227543	2230541	-
chr12	37513023	37517972	-	chr2	58106226	58113632	+	chr6	22468490	22471024	-
chr12	3798721	3887907	-	chr2	59007410	59018755	-	chr6	22671183	22671881	+
chr12	38353300	38353542	+	chr2	59619431	59620068	+	chr6	22800431	22806443	+
chr12	40369593	40371334	+	chr2	59619431	59620094	+	chr6	2317201	2320815	+
chr12	40441315	40507405	+	chr2	7470755	7473987	-	chr6	27870489	27881158	+
chr12	40476466	40524894	+	chr2	9324789	9328823	+	chr6	27891395	27892010	+
chr12	4183598	4189439	-	chr2	9817145	9817587	+	chr6	28396562	28400055	+
chr12	4205648	4205821	-	chr20	12990786	12990992	-	chr6	29009363	29011363	-
chr12	4246352	4247947	+	chr20	13182517	13186030	+	chr6	29847686	29853397	-
chr12	43382577	43386696	-	chr20	15091590	15100493	-	chr6	30119707	30123022	+
chr12	43389952	43403869	-	chr20	153719	154628	-	chr6	3174029	3182228	+
chr12	45246871	45251774	-	chr20	21900017	21913509	-	chr6	3182042	3182228	+
chr12	46617105	46617607	-	chr20	23320015	23324750	+	chr6	32028432	32029760	+
chr12	48016566	48021113	+	chr20	2332276	2340826	-	chr6	33246561	33248630	+
chr12	48016566	48032291	+	chr20	2332276	2341615	-	chr6	33511025	33512911	-
chr12	48016566	48038865	+	chr20	23441054	23443998	-	chr6	37344119	37358480	-
chr12	4813742	4837133	+	chr20	24309910	24320982	+	chr6	37460619	37465923	-
chr12	48165645	48166134	+	chr20	2459372	2461915	+	chr6	37461220	37465923	-
chr12	4818167	4842114	+	chr20	25620763	25621463	+	chr6	37705915	37706241	+
chr12	4822221	4848263	+	chr20	2624400	2625271	+	chr6	39067035	39068837	-
chr12	49373268	49379974	-	chr20	26998812	27000501	+	chr6	39070782	39075437	-
chr12	4993342	5007059	-	chr20	26998812	27003399	+	chr6	40511827	40512257	+
chr12	4994042	5007247	-	chr20	27350082	27351377	-	chr6	40861882	40869279	+
chr12	49947051	49947327	+	chr20	27360365	27361919	+	chr6	41422592	41424334	+
chr12	50189993	50190882	-	chr20	28313652	28314507	-	chr6	4225179	4225454	+
chr12	50392443	50480742	-	chr20	2919303	2924446	-	chr6	43945566	43946013	-
chr12	5266247	5267071	-	chr20	2932657	2933459	-	chr6	45262603	45265503	+
chr12	5269282	5288998	-	chr20	3061427	3063193	-	chr6	45274464	45363011	+
chr12	6226966	6227335	-	chr20	33171146	33171406	-	chr6	45285728	45297930	+
chr12	8361730	8383177	-	chr20	33513383	33514730	-	chr6	47432861	47433092	-
chr12	8372233	8383177	-	chr20	33997443	33999661	-	chr6	47437137	47437310	-

chr12	9339752	9345640	-	chr20	35628975	35640340	-	chr6	52262367	52269110	+
chr12	941294	945837	+	chr20	35642253	35644356	-	chr6	55283038	55283626	-
chr12	9852088	9853358	-	chr20	42354572	42373010	-	chr6	55283038	55289121	-
chr13	10655931	10656389	+	chr20	43228156	43240878	-	chr6	56048470	56057673	+
chr13	11481730	11490650	-	chr20	433727	434128	-	chr6	56055846	56057673	+
chr13	1266763	1267536	-	chr20	4339467	4341546	+	chr6	59402287	59407048	+
chr13	12801388	12815428	+	chr20	44810415	44812707	+	chr6	59406882	59407048	+
chr13	1286658	1288659	-	chr20	457173	457443	-	chr6	59674611	59693920	+
chr13	1413823	1415178	+	chr20	45881617	45888602	-	chr6	6859391	6864548	+
chr13	15572999	15574407	+	chr20	47185706	47186539	+	chr6	7965796	7966246	+
chr13	15666877	15679436	+	chr20	50334159	50342735	+	chr7	1087930	1088156	-
chr13	15717329	15717780	-	chr20	51681665	51686502	-	chr7	11608325	11618700	+
chr13	15792126	15796829	+	chr20	51711746	51714567	-	chr7	12110349	12113231	+
chr13	15919625	15920020	-	chr20	53228507	53261320	-	chr7	14465141	14515259	-
chr13	18216516	18216877	-	chr20	53289706	53292230	-	chr7	14553352	14563524	-
chr13	18323730	18326125	+	chr20	54325342	54338813	-	chr7	14878384	14878697	+
chr13	18443681	18446466	+	chr20	54555046	54559197	+	chr7	14992861	14993151	+
chr13	1976046	1978932	-	chr20	54767142	54771372	-	chr7	16533008	16550235	+
chr13	2104006	2108727	-	chr20	55029269	55067316	+	chr7	17313922	17314395	+
chr13	2334605	2339941	+	chr20	6415027	6416002	-	chr7	21227328	21227839	-
chr13	23432682	23439871	+	chr20	6707023	6718399	+	chr7	21617700	21618537	-
chr13	24164555	24167418	-	chr20	6707023	6725381	+	chr7	22026089	22028956	+
chr13	25462483	25466539	-	chr20	7050500	7051069	-	chr7	22837017	22863364	+
chr13	26060248	26063400	-	chr20	8264914	8269412	-	chr7	22954205	22954703	-
chr13	26777967	26781508	-	chr20	8353598	8363989	-	chr7	23045136	23045599	-
chr13	2963936	2964420	-	chr21	11341102	11342060	+	chr7	24861148	24866599	+
chr13	30262500	30262874	+	chr21	11454085	11457043	+	chr7	24894840	24895621	-
chr13	30398863	30404792	+	chr21	11566742	11568550	+	chr7	24941155	24956861	-
chr13	33647250	33649273	+	chr21	11673247	11675981	-	chr7	25134532	25139567	-
chr13	34689974	34752826	+	chr21	1672138	1679276	+	chr7	25543289	25545576	-
chr13	34949881	34956878	+	chr21	19601909	19671560	+	chr7	25544048	25545576	-
chr13	34949881	34968819	+	chr21	22170826	22175913	-	chr7	25765779	25766314	-
chr13	3580648	3582605	-	chr21	24660952	24662082	-	chr7	26331329	26338905	+
chr13	37566804	37572058	-	chr21	2628512	2629427	+	chr7	26335609	26352715	+
chr13	37626432	37628618	-	chr21	29500122	29500605	+	chr7	26418542	26422343	+
chr13	4227586	4247615	+	chr21	2964459	2968677	+	chr7	28158829	28162426	-
chr13	43031484	43031686	-	chr21	32470971	32472458	+	chr7	28346523	28347519	+
chr13	43179260	43181486	-	chr21	32989077	32992496	-	chr7	28446335	28453512	+
chr13	43446474	43514179	+	chr21	33220271	33220819	-	chr7	28838425	28841965	-
chr13	4383628	4383987	-	chr21	34374195	34385293	-	chr7	28850689	28854079	-
chr13	45332086	45335650	-	chr21	34383090	34385293	-	chr7	31761777	31762488	-
chr13	45656004	45663674	-	chr21	35847101	35867663	-	chr7	33137231	33137980	-
chr13	46124368	46130632	-	chr21	36977249	36983955	-	chr7	33195018	33200137	+
chr13	46135107	46135257	-	chr21	36983486	36983955	-	chr7	33196906	33200137	+
chr13	46754263	46754740	-	chr21	36983486	36989645	-	chr7	34488473	34509529	+
chr13	48158057	48166139	+	chr21	37684438	37686479	-	chr7	35311993	35312329	+
chr13	50143520	50145528	-	chr21	37684446	37686479	-	chr7	36199981	36202580	+
chr13	51205914	51207794	-	chr21	38488451	38488779	-	chr7	36308606	36309052	-
chr13	52015866	52018269	+	chr21	38994853	39002098	-	chr7	36320489	36323253	-
chr13	52169253	52174800	+	chr21	39724459	39732362	-	chr7	37884612	37885018	+
chr13	52193222	52197559	+	chr21	40675959	40683003	+	chr7	38353765	38356518	+
chr13	53649055	53655278	+	chr21	40675959	40683073	+	chr7	38353765	38356920	+
chr13	53653346	53653778	+	chr21	40675959	40683756	+	chr7	39933081	39949426	-
chr13	5570882	5574611	-	chr21	40675959	40683855	+	chr7	40101393	40101823	-
chr13	7619202	7619479	+	chr21	41626015	41628540	+	chr7	40807182	40819806	+
chr14	1012460	1015698	+	chr21	42264572	42267441	+	chr7	40846753	40847337	+
chr14	1030714	1031735	+	chr21	432173	490598	-	chr7	4145872	4152942	-
chr14	1120232	1127996	+	chr21	43627993	43636803	-	chr7	42102830	42102991	+
chr14	1154317	1170247	-	chr21	44072980	44074924	+	chr7	42371412	42377543	+
chr14	13722474	13724167	+	chr21	44261577	44261953	-	chr7	43199145	43207961	-
chr14	20025440	20026865	-	chr21	4914747	4917819	-	chr7	43669749	43672283	-
chr14	20098053	20098577	+	chr21	584572	592260	+	chr7	44142370	44143556	-
chr14	21956169	21974711	+	chr21	7612882	7615139	-	chr7	50099090	50105552	+
chr14	21956169	21979654	+	chr21	7868883	7872140	-	chr7	52061577	52072761	-
chr14	24561629	24562187	+	chr21	8843922	8850333	+	chr7	52107465	52108971	-
chr14	29371840	29386165	+	chr21	9479068	9498534	-	chr7	54898538	54898944	+

chr14	30655650	30658797	-	chr22	10004353	10010030	-	chr7	56619236	56619727	-
chr14	3069185	3069404	-	chr22	10039348	10043323	-	chr7	57634197	57641511	+
chr14	31299687	31302961	-	chr22	11114610	11115158	-	chr7	59109060	59119234	+
chr14	34892801	34897172	-	chr22	11978578	11981871	+	chr7	59197596	59208991	-
chr14	38870486	38870862	+	chr22	14395571	14395950	-	chr7	59197596	59208994	-
chr14	41643224	41648310	-	chr22	15730655	15732968	-	chr7	59204584	59208991	-
chr14	4270057	4276095	-	chr22	161167	166147	+	chr7	59204584	59208994	-
chr14	42831156	42888534	+	chr22	1639732	1640912	+	chr7	60062175	60062690	-
chr14	47005872	47006375	-	chr22	1639732	1640916	+	chr7	60764089	60764407	-
chr14	47748243	47748537	-	chr22	17979495	17984471	-	chr7	6520629	6546631	+
chr14	47994943	48009814	+	chr22	18577301	18577559	+	chr7	65638586	65638896	-
chr14	49160546	49209905	+	chr22	19271309	19279711	-	chr7	67220217	67220469	-
chr14	51645249	51646434	+	chr22	2014549	2040895	+	chr7	71040806	71065717	+
chr14	53473753	53474830	-	chr22	20482591	20483399	-	chr7	72889012	72896254	+
chr14	5860940	5861458	+	chr22	21134511	21136099	-	chr7	730242	730938	+
chr14	6257546	6258312	-	chr22	2122414	2122702	+	chr7	73982175	73983344	-
chr14	6425997	6428537	+	chr22	21330956	21352542	-	chr7	74364252	74375145	-
chr14	6586155	6589767	+	chr22	2195719	2196148	-	chr7	74386907	74390346	-
chr14	8578606	8579227	+	chr22	2397152	2398514	-	chr7	74560232	74564243	+
chr14	8593554	8593930	+	chr22	24442931	24461197	-	chr7	74741339	74744628	+
chr15	13633323	13633829	+	chr22	24709064	24710230	-	chr7	75139930	75142405	-
chr15	15229938	15230325	-	chr22	25188979	25191552	+	chr7	75170032	75170375	-
chr15	16221090	16229746	-	chr22	25614005	25657881	+	chr7	75212330	75213373	-
chr15	1656294	1658972	+	chr22	26737881	26798059	-	chr7	75282430	75284919	+
chr15	16901121	16903905	-	chr22	26754756	26815384	-	chr7	76137832	76167440	+
chr15	16923501	16930225	-	chr22	26759109	26819042	-	chr7	76185714	76218606	+
chr15	16926864	16930225	-	chr22	2707410	2707945	+	chr7	76187278	76188753	+
chr15	19868000	19870356	-	chr22	27421862	27422262	+	chr7	76712554	76716324	-
chr15	20785305	20791571	-	chr22	31819952	31822673	+	chr7	76723975	76725764	-
chr15	22597300	22597736	+	chr22	32391789	32393716	-	chr7	76783197	76784149	+
chr15	22972361	22975221	+	chr22	32422016	32434213	-	chr7	8740273	8785433	-
chr15	23196770	23229665	-	chr22	3467023	3470297	-	chr7	9102484	9103338	-
chr15	2392181	2403233	+	chr22	37418670	37419218	-	chr8	11353326	11353524	-
chr15	24799151	24800185	+	chr22	39629990	39647103	-	chr8	11678714	11687728	+
chr15	28458692	28459253	-	chr22	39631933	39650351	-	chr8	12187596	12190762	+
chr15	2912411	2912918	-	chr22	39632680	39652046	-	chr8	12191944	12192180	+
chr15	29633325	29655374	+	chr22	40715168	40718263	-	chr8	12348719	12350302	-
chr15	29637991	29655374	+	chr22	40743245	40765549	-	chr8	17038900	17039229	-
chr15	29675650	29677803	+	chr22	42032257	42033262	+	chr8	18491223	18494371	-
chr15	3676724	3677152	+	chr22	4300830	4303551	+	chr8	19051381	19052330	-
chr15	37013944	37021556	+	chr22	458681	461947	-	chr8	1908283	1909186	-
chr15	38726333	38730489	+	chr22	5614673	5614984	+	chr8	19783651	19786181	-
chr15	4011866	4013630	-	chr22	6776374	6789618	-	chr8	2031084	2034228	+
chr15	40239648	40259563	-	chr22	7013876	7046134	-	chr8	21064595	21084454	+
chr15	40244454	40273339	-	chr22	8469120	8534144	-	chr8	21537282	21561745	+
chr15	42490448	42490697	-	chr23	1145819	1185209	-	chr8	21592811	21595031	-
chr15	43061461	43080344	-	chr23	1172682	1189492	-	chr8	21661523	21662058	+
chr15	4325819	4331988	-	chr23	1185080	1189492	-	chr8	23659886	23679949	-
chr15	4325819	4340622	-	chr23	13029839	13030751	+	chr8	24533791	24548157	-
chr15	43856519	43865453	+	chr23	13660000	13660381	+	chr8	25934684	25935998	+
chr15	44795590	44801220	+	chr23	1384153	1421047	-	chr8	27208468	27214520	+
chr15	458071	461842	+	chr23	1420419	1468691	-	chr8	2775588	2786622	+
chr15	46232414	46295234	-	chr23	15186511	15187691	-	chr8	32926851	32929273	-
chr15	46375614	46381458	+	chr23	17080987	17108442	+	chr8	33291006	33305131	-
chr15	5477985	5483212	+	chr23	18057672	18059225	+	chr8	33294781	33309929	-
chr15	57997	69140	-	chr23	18185459	18197645	-	chr8	37469811	37474185	+
chr16	10229738	10251842	-	chr23	19725900	19726499	-	chr8	43360892	43367867	+
chr16	10393648	10397326	+	chr23	21802569	21804715	-	chr8	43874710	43874906	-
chr16	10502040	10513846	+	chr23	22312751	22316761	+	chr8	45898932	45904256	+
chr16	1290862	1293650	+	chr23	26439773	26442636	+	chr8	46563896	46584721	-
chr16	1330814	1335433	+	chr23	2769645	2781497	+	chr8	47223751	47224584	-
chr16	15891196	15899109	-	chr23	2772074	2774743	+	chr8	4763617	4764678	-
chr16	15893366	15899109	-	chr23	2835987	2847282	+	chr8	48119328	48127017	-
chr16	15918978	15923748	-	chr23	28948717	28950191	+	chr8	48556230	48560098	+
chr16	1893449	1903956	+	chr23	29231346	29237041	-	chr8	48601122	48653364	-
chr16	19325904	19343460	-	chr23	30176458	30190343	+	chr8	52683401	52687391	+

chr16	19332746	19343460	-	chr23	30975114	30975837	+	chr8	55313597	55314726	+
chr16	19333129	19343460	-	chr23	31672000	31672413	+	chr8	55371422	55377091	-
chr16	19714960	19719098	-	chr23	32282975	32291921	+	chr8	6374982	6433723	-
chr16	24545961	24551656	-	chr23	32284137	32286291	+	chr8	7614690	7615615	+
chr16	24979223	24984740	-	chr23	32284167	32286291	+	chr8	9449655	9459840	+
chr16	27019509	27034979	-	chr23	33676767	33680037	-	chr8	9637117	9638580	-
chr16	28706309	28710390	+	chr23	34363288	34365107	+	chr9	1069789	1070013	-
chr16	28719287	28719484	+	chr23	349638	351998	-	chr9	10930282	10934681	+
chr16	28812973	28822282	+	chr23	35361164	35362333	-	chr9	14718483	14722726	-
chr16	28825358	28825663	+	chr23	35797556	35798375	-	chr9	16607972	16650537	+
chr16	29383957	29385999	-	chr23	37887085	37889276	+	chr9	1662693	1663038	+
chr16	30620935	30634695	+	chr23	39725983	39726563	+	chr9	17689537	17694798	-
chr16	31335352	31349121	-	chr23	401066	405247	+	chr9	18804996	18805569	-
chr16	3178553	3182379	+	chr23	41148039	41154776	-	chr9	23883713	23887169	-
chr16	33111878	33112648	-	chr23	42958444	42986438	-	chr9	23939481	23939805	-
chr16	33588864	33589621	-	chr23	44142832	44145351	+	chr9	26187345	26188315	+
chr16	34125672	34127980	-	chr23	44142832	44145356	+	chr9	26359710	26359880	-
chr16	3429512	3429957	+	chr23	45436407	45442786	+	chr9	28913340	28913669	+
chr16	35416442	35416787	-	chr23	46100405	46101455	-	chr9	29301724	29304511	-
chr16	36388117	36391193	+	chr23	46100405	46107703	-	chr9	3086397	3088676	+
chr16	38164688	38165216	+	chr23	46116458	46116657	-	chr9	31791264	31798224	+
chr16	39555399	39555633	-	chr23	549189	552121	-	chr9	32075886	32088374	+
chr16	39601901	39606269	+	chr23	549189	552124	-	chr9	34403322	34415076	-
chr16	3976960	3977142	+	chr23	5557595	5571771	+	chr9	35175650	35176876	-
chr16	4016991	4021988	+	chr23	5658798	5661713	-	chr9	35879756	35883253	-
chr16	40978432	40985639	-	chr23	6188356	6188619	+	chr9	35989006	35991369	+
chr16	42332914	42351822	-	chr23	6396590	6402082	-	chr9	38397453	38397627	+
chr16	44470092	44470277	-	chr23	6651678	6664169	+	chr9	4015268	4019583	-
chr16	44528984	44532443	-	chr23	6963215	6972914	-	chr9	4052962	4055399	-
chr16	44691041	44691510	-	chr23	7069336	7069794	-	chr9	4104875	4108984	+
chr16	46360562	46365194	-	chr23	954309	958348	-	chr9	42383409	42396789	-
chr16	47215342	47242417	-	chr23	9575770	9593555	-	chr9	42386504	42396789	-
chr16	47391925	47423250	-	chr23	961153	972375	-	chr9	44180265	44180927	-
chr16	47518273	47518559	+	chr23	9813080	9816264	-	chr9	44872872	44874062	+
chr16	47768328	47769224	-	chr24	17560879	17565366	+	chr9	453892	454300	+
chr16	47774809	47775152	-	chr24	21521539	21529936	+	chr9	45571417	45577735	-
chr16	48829858	48839628	+	chr24	22019390	22019926	-	chr9	47064793	47065393	-
chr16	50614138	50619036	-	chr24	22043522	22046198	-	chr9	48645068	48645830	-
chr16	52020456	52039146	+	chr24	22043522	22053508	-	chr9	50853213	50853447	-
chr16	5560087	5573153	-	chr24	22043522	22058441	-	chr9	51056832	51057211	+
chr16	55964658	55967490	+	chr24	2498598	2499173	-	chr9	51472899	51476130	+
chr16	56209460	56218181	-	chr24	26688776	26692374	+	chr9	52889603	52908445	-
chr16	56432614	56441840	-	chr24	26688776	26694808	+	chr9	54809033	54809376	-
chr16	56436247	56441840	-	chr24	30596701	30597004	+	chr9	55311462	55324784	-
chr16	56769815	56770421	+	chr24	30820087	30823974	-	chr9	56415912	56416403	-
chr16	57127251	57127533	+	chr24	30958382	30960638	+	chr9	56725952	56733379	+
chr16	7438840	7444681	-	chr24	34459948	34462418	-	chr9	56739105	56745358	+
chr16	8116905	8120714	+	chr24	34468020	34469908	-	chr9	58001623	58001850	-
chr16	8232058	8284671	+	chr24	3452388	3456065	-	chr9	58110797	58179977	-
chr16	9025066	9027548	-	chr24	36488141	36491118	+	chr9	597804	598360	-
chr17	1136605	1152121	+	chr24	37471815	37473770	+	chr9	6142779	6150315	+
chr17	1145578	1148493	+	chr24	37763427	37763760	-	chr9	6324141	6336945	+
chr17	11555728	11555990	+	chr24	37777102	37777503	-	chr9	786634	790254	-
chr17	12886341	12886851	-	chr24	37786450	37795988	-	chr9	912347	917309	-
chr17	12954893	12955144	+	chr24	37876688	37880605	+	chr9	9431200	9431655	-
chr17	145524	150594	-	chr24	39680494	39682260	+	Zv9_NA118	4162	4733	+
chr17	15644356	15651393	+	chr24	42246913	42321240	-	Zv9_NA169	732	2873	+
chr17	16463612	16464168	+	chr24	42267640	42299805	-	Zv9_NA18	73	466	+
chr17	17884097	17885559	+	chr24	42269718	42350094	-	Zv9_NA250	14179	15693	-
chr17	17915184	17919738	+	chr24	43528323	43542737	+	Zv9_NA327	5783	8164	+
chr17	19890304	19891111	-	chr24	43834346	43834856	-	Zv9_NA331	49575	49820	-
chr17	19911129	19912376	-	chr25	11613393	11618556	+	Zv9_NA360	13047	14772	-
chr17	20546607	20564417	-	chr25	12597186	12600995	+	Zv9_NA360	4852	13221	-
chr17	2270102	2284306	-	chr25	13358935	13361144	+	Zv9_NA384	48864	58142	+
chr17	2276447	2284306	-	chr25	14865157	14890001	-	Zv9_NA401	15984	16405	-
chr17	2280349	2284306	-	chr25	16974527	16974896	-	Zv9_NA401	3657	4045	-

chr17	25105645	25106727	-	chr25	1698452	1699432	-	Zv9_NA500	417	803	-
chr17	28781285	28784511	+	chr25	17684518	17685240	+	Zv9_NA807	37454	57376	+
chr17	28781285	28790031	+	chr25	1881	28256	+	Zv9_NA900	4370	22871	-
chr17	28852069	28853093	-	chr25	20391520	20391806	+	Zv9_NA915	36062	60974	-
chr17	30537912	30538804	-	chr25	20578997	20586330	+	Zv9_NA947	157490	160863	+
chr17	30562242	30564667	-	chr25	21978947	21984104	-	Zv9_NA947	73558	77246	-
chr17	31435542	31448396	+	chr25	22850335	22856739	+	Zv9_scaffol	151516	155713	+
chr17	35375187	35375440	+	chr25	24284888	24285855	-	Zv9_scaffol	61378	71914	+
chr17	37351200	37358291	-	chr25	24349065	24351358	+	Zv9_scaffol	73336	73641	+
chr17	40002068	40007738	+	chr25	26738783	26739425	+	Zv9_scaffol	105372	108364	+
chr17	4098906	4099222	+	chr25	27607207	27610180	-	Zv9_scaffol	21030	57201	-
								Zv9_scaffol	91725	95344	-
								Zv9_scaffol	165026	166329	-
								Zv9_scaffol	15110	17650	-
								Zv9_scaffol	27008	34918	+

Supplementary Table 8: Genome-wide distribution of total uniq circRNAs

	**Ensembl**			**Refseq**		
	CircRNAs	Length	CircRNA*10^5/Length	CircRNAs	Length	CircRNA*10^5/Length
3UTR	1103	25163692	4.383299557	708	8125258	8.713569465
5UTR	1041	8305389	12.53403062	677	2029505	33.35788776
Exons	2979	98560362	3.022513249	1785	30753486	5.804220048
Introns	2782	1128883593	0.246438164	1682	407983536	0.412271538
WholgeGene	3015	1226670151	0.245787345	1833	438486998	0.418028359
cds	2909	65140982	4.465698721	1743	20625234	8.450813213
downStm1000	1287	56742849	2.268127214	689	16083662	4.283850282
upStm1000	1262	56770469	2.222986743	640	16281787	3.930772464

Supplementary Table 9: GP analysis for genes encoding Exonic circular RNA using DAVID

Category Term Count % PValue Genes List Total Pop Hits Pop Total Fold Enrichment Bonferroni Benjamini FDR

UP_KEYWORDS Coiled coil 50 30.120481927710845 1.1995642487930577E-7 CREBBPB, ENAH, JPH2, PPP1R13BB, MYBPC3, EIF3S10, LUZP2, CANX, SPICE1, CTTN, SETB, VPS13A, SI:CH211-250G4.3, CEP85, FMNL3, NBAS, CTNNA2, SLTM, USP28, MED4, SMARCC1A, KRT15, FILIP1B, AMOTL2A, ALPK3A, EPB41B, MIPOL1, SI:DKEYP-68B7.7, CALR, NMT1B, TFDP1B, STX16, VMHCL, TBC1D4, FBXO41, SLC4A1AP, MAML3, SETA, GOLIM4A, HCLS1, TNNT3B, CENPE, SI:CH211-220F16.2, HDAC5, EPS15, RABEP1, SYF2, SYNM, SETD2, SCG2A 162 3222 22487 2.1540757600141003 1.4394668247708076E-5 1.4394668247708076E-5 1.377017617709697E-4

GOTERM_BP_DIRECT GO:0045214~sarcomere organization 4 2.4096385542168677 0.0016734753265341812 MYL7, TTNB, TNNT3B, SLC8A1A 115 31 14912 16.73155680224404 0.39293134619988623 0.39293134619988623 2.2110488141640627

GOTERM_CC_DIRECT GO:0005750~mitochondrial respiratory chain complex III 3 1.8072289156626504 0.0019711386273206727 UQCRC2B, UQCRC1, UQCRB 116 9 15208 43.701149425287355 0.17092359960080883 0.17092359960080883 2.14547341054816

KEGG_PATHWAY dre00190:Oxidative phosphorylation 7 4.216867469879518 0.002227154793737302 UQCRC2B, ATP5J2, UQCRC1, ATP5B, ATP5G1, ATP5A1, UQCRB 54 140 5434 5.031481481481481 0.11540920774122088 0.11540920774122088 2.1691631011209744

GOTERM_BP_DIRECT GO:0006122~mitochondrial electron transport, ubiquinol to cytochrome c 3 1.8072289156626504 0.00427682452374953 UQCRC2B, UQCRC1, UQCRB 115 13 14912 29.92374581939799 0.7211906033478431 0.47197595068770126 5.560940985527885

GOTERM_BP_DIRECT GO:0030041~actin filament polymerization 3 1.8072289156626504 0.00427682452374953 CTTN, FMNL3, HCLS1 115 13 14912 29.92374581939799 0.7211906033478431 0.47197595068770126 5.560940985527885

GOTERM_MF_DIRECT GO:0046933~proton-transporting ATP synthase activity, rotational mechanism 3 1.8072289156626504 0.006992617387258904 ATP5B, ATP5G1, ATP5A1 119 17 15797 23.42609985170539 0.7542489044194159 0.7542489044194159 8.419616553213082

GOTERM_BP_DIRECT GO:0009060~aerobic respiration 3 1.8072289156626504 0.008182754248407623 UQCRC2B, UQCRC1, UQCRB 115 18 14912 21.611594202898548 0.9135762009569495 0.5578756329523873 10.388287480134107

GOTERM_BP_DIRECT GO:0000184~nuclear-transcribed mRNA catabolic process, nonsense-mediated decay 3 1.8072289156626504 0.009100060984149479 NBAS, PNRC2, DHX34 115 19 14912 20.474141876430206 0.9344034646703155 0.49391877551568353 11.488383408095826

GOTERM_BP_DIRECT GO:0006457~protein folding 5 3.0120481927710845 0.012760790372234713 HSP90AB1, PPIH, FKBP5, CALR, CANX 115 118 14912 5.494473102431835 0.9782292240917779 0.5348695395517348 15.755339893613352

GOTERM_BP_DIRECT GO:2000623~negative regulation of nuclear-transcribed mRNA catabolic process, nonsense-mediated decay 2 1.2048192771084338 0.015231764621992737 NBAS, DHX34 115 2 14912 129.6695652173913 0.9896832855771346 0.5334227402177466 18.5270605677628

GOTERM_BP_DIRECT GO:0015986~ATP synthesis coupled proton transport 3 1.8072289156626504 0.016705082869322326 ATP5B, ATP5G1, ATP5A1 115 26 14912 14.961872909698995 0.9933965533626029 0.5118664395898749 20.139299948380085

INTERPRO IPR003134:Hs1/Cortactin 2 1.2048192771084338 0.021680590860355734 CTTN, HCLS1 156 3 21293 90.9957264957265 0.9998829108686558 0.9998829108686558 26.446057999337846

GOTERM_BP_DIRECT GO:0015992~proton transport 3 1.8072289156626504 0.023317597125403003 ATP5B, ATP5G1, ATP5A1 115 31 14912 12.548667601683029 0.9991158984054527 0.5847470066627882 27.01835589178998

GOTERM_CC_DIRECT GO:0005634~nucleus 32 19.27710843373494 0.027645471825684727 HSP90AB1, CREBBPB, ZNF536, PPP1R13BB, ARNT2, NUFIP1, SOX9B, MEF2AA, CHD9, TFDP1B, ERCC5, INTS7, NIPBLB, DHX34, SETB, SETA, INO80DA, GTF2H4, GADD45BA, FOXN3, CTNNA2, SLTM, HDAC5, AHR2, USP28, MED4, SMARCC1A, PNRC2, SYF2, SETD2, SCMH1, HDAC8 116 2933 15208 1.430381979143398 0.9302826459279926 0.7359595597791743 26.520216264259254

KEGG_PATHWAY dre04530:Tight junction 6 3.614457831325301 0.028159409987657022 MYL7, CTTN, HCLS1, VMHCL, MYL9B, CTNNA2 54 177 5434 3.411173885750157 0.7921610859285673 0.5441064662978508 24.493067964668313

UP_KEYWORDS CF(1) 2 1.2048192771084338 0.02833453714493977 ATP5B, ATP5A1 162 4 22487 69.40432098765432 0.9682303845090562 0.8217596692918692 28.104627996006304

GOTERM_BP_DIRECT GO:0031016~pancreas development 3 1.8072289156626504 0.03242403544405767 CFTR, RPL23A, SOX9B 115 37 14912 10.513748531139836 0.9999457810221201 0.6642493871889259 35.59724700858774

INTERPRO IPR018124:Calreticulin/calnexin, conserved site 2 1.2048192771084338 0.03587420709382523 CALR, CANX 156 5 21293 54.59743589743589 0.9999997199608599 0.9994708127551429 40.06698757883651

INTERPRO IPR009033:Calreticulin/calnexin, P domain 2 1.2048192771084338 0.03587420709382523 CALR, CANX 156 5 21293 54.59743589743589 0.9999997199608599 0.9994708127551429 40.06698757883651

INTERPRO IPR000793:ATPase, F1/V1/A1 complex, alpha/beta subunit, C-terminal 2 1.2048192771084338 0.03587420709382523 ATP5B, ATP5A1 156 5 21293 54.59743589743589 0.9999997199608599 0.9994708127551429 40.06698757883651

GOTERM_CC_DIRECT GO:0045261~proton-transporting ATP synthase complex, catalytic core F(1) 2 1.2048192771084338 0.037246370322488606 ATP5B, ATP5A1 116 5 15208 52.44137931034483 0.9728393199269317 0.6994060652558501 34.113278443684635

GOTERM_BP_DIRECT GO:0016485~protein processing 3 1.8072289156626504 0.04090100518335074 UQCRC2B, UQCRC1, FURINA 115 42 14912 9.262111801242234 0.9999960617446 0.7119085378807375 42.73517668572096

GOTERM_BP_DIRECT GO:0015991~ATP hydrolysis coupled proton transport 3 1.8072289156626504 0.042686462112180114 ATP5B, ATP5G1, ATP5A1 115 43 14912 9.046713852376136 0.9999977397745344 0.6932807246836359 44.14203270283335

INTERPRO IPR002164:Nucleosome assembly protein (NAP) 2 1.2048192771084338 0.042894100830309354 SETA, SETB 156 6 21293 45.49786324786325 0.9999999863076774 0.9976076445437121 45.90052638090114

INTERPRO IPR001580:Calreticulin/calnexin 2 1.2048192771084338 0.042894100830309354 CALR, CANX 156 6 21293 45.49786324786325 0.9999999863076774 0.9976076445437121 45.90052638090114

GOTERM_MF_DIRECT GO:0032041~NAD-dependent histone deacetylase activity (H3-K14 specific) 2 1.2048192771084338 0.04399679669728569 HDAC5, HDAC8 119 6 15797 44.24929971988795 0.9998764423909055 0.9888843529610487 43.104470046141195

GOTERM_BP_DIRECT GO:0000956~nuclear-transcribed mRNA catabolic process 2 1.2048192771084338 0.04500872985956283 NBAS, DHX34 115 6 14912 43.2231884057971 0.9999989039719401 0.6813466616203003 45.924044208438886

UP_KEYWORDS ATP synthesis 2 1.2048192771084338 0.04906052005757633 ATP5B, ATP5A1 162 7 22487 39.65961199294533 0.9976102889399776 0.8663050105860248 43.86808681589797

INTERPRO IPR000194:ATPase, F1/V1/A1 complex, alpha/beta subunit, nucleotide-binding domain 2 1.2048192771084338 0.049863209413237755 ATP5B, ATP5A1 156 7 21293 38.998168498168496 0.9999999993306184 0.9949135072792868 51.166497452093516

INTERPRO IPR004100:ATPase, alpha/beta subunit, N-terminal 2 1.2048192771084338 0.049863209413237755 ATP5B, ATP5A1 156 7 21293 38.998168498168496 0.9999999993306184 0.9949135072792868 51.166497452093516

INTERPRO IPR020003:ATPase, alpha/beta subunit, nucleotide-binding domain, active site 2 1.2048192771084338 0.049863209413237755 ATP5B, ATP5A1 156 7 21293 38.998168498168496 0.9999999993306184 0.9949135072792868 51.166497452093516

GOTERM_CC_DIRECT GO:0033178~proton-transporting two-sector ATPase complex, catalytic domain 2 1.2048192771084338 0.05175686621600954 ATP5B, ATP5A1 116 7 15208 37.45812807881774 0.9935824075570642 0.7169631166933346 44.24288330658568

GOTERM_BP_DIRECT GO:0007519~skeletal muscle tissue development 3 1.8072289156626504 0.05203426432985749 KLHL31, TTNB, LDB3A 115 48 14912 8.104347826086956 0.9999998786033453 0.7062242785367834 51.00002120508176

GOTERM_BP_DIRECT GO:0046847~filopodium assembly 2 1.2048192771084338 0.05231243338886841 FMNL3, AMOTL2A 115 7 14912 37.048447204968944 0.9999998887693469 0.6813589319643432 51.19161735307254

GOTERM_BP_DIRECT GO:0006754~ATP biosynthetic process 2 1.2048192771084338 0.05956076499586976 ATP5B, ATP5A1 115 8 14912 32.417391304347824 0.9999999887134715 0.7047630621729513 55.946376939208605

UP_KEYWORDS Chaperone 3 1.8072289156626504 0.061021177785774754 HSP90AB1, CALR, CANX 162 56 22487 7.436177248677249 0.9994767663424351 0.8487574753993532 51.45918326975786

INTERPRO IPR011765:Peptidase M16, N-terminal 2 1.2048192771084338 0.06365052863618936 UQCRC2B, UQCRC1 156 9 21293 30.33190883190883 0.9999999999984008 0.9956270320762938 60.211162173502245
INTERPRO IPR007863:Peptidase M16, C-terminal domain 2 1.2048192771084338 0.06365052863618936 UQCRC2B, UQCRC1 156 9 21293 30.33190883190883 0.9999999999984008 0.9956270320762938 60.211162173502245
INTERPRO IPR011249:Metalloenzyme, LuxS/M16 peptidase-like 2 1.2048192771084338 0.06365052863618936 UQCRC2B, UQCRC1 156 9 21293 30.33190883190883 0.9999999999984008 0.9956270320762938 60.211162173502245
INTERPRO IPR011237:Peptidase M16 domain 2 1.2048192771084338 0.06365052863618936 UQCRC2B, UQCRC1 156 9 21293 30.33190883190883 0.9999999999984008 0.9956270320762938 60.211162173502245

GOTERM_MF_DIRECT GO:0016820~hydrolase activity, acting on acid anhydrides, catalyzing transmembrane movement of substances 2 1.2048192771084338 0.06526990343218587 ATP5B, ATP5A1 119 9 15797 29.49953314659197 0.9999986283437787 0.9888891207999204 57.08766068621558

KEGG_PATHWAY dre04260:Cardiac muscle contraction 4 2.4096385542168677 0.06642742039187699 UQCRC2B, UQCRC1, VMHCL, UQCRB 54 96 5434 4.192901234567901 0.9771889747714474 0.7163943132215146 49.13938921860021
GOTERM_BP_DIRECT GO:0006306~DNA methylation 2 1.2048192771084338 0.06675414078692979 GATAD2AB, DNMT3AB 115 9 14912 28.815458937198066 0.9999999988549371 0.7238297872687876 60.238217250216806
KEGG_PATHWAY dre00270:Cysteine and methionine metabolism 3 1.8072289156626504 0.06808597310071965 DNMT3AB, APIP, AMD1 54 44 5434 6.861111111111111 0.979314230734404 0.6207566981792741 50.02118436068621
UP_KEYWORDS Zymogen 2 1.2048192771084338 0.06934709771403516 CASP7, AMD1 162 10 22487 27.761728395061727 0.9998203081722088 0.821800834773687 56.176772713017165
INTERPRO IPR023801:Histone deacetylase domain 2 1.2048192771084338 0.07046946154703582 HDAC5, HDAC8 156 10 21293 27.298717948717947 0.9999999999999218 0.9934614401766759 64.08467524229668
GOTERM_BP_DIRECT GO:0043009~chordate embryonic development 4 2.4096385542168677 0.07217761761486549 INSRB, NBAS, DHX34, PAPPAB 115 126 14912 4.116494133885438 0.9999999997983845 0.7310460840904967 63.21459719426744
KEGG_PATHWAY dre04520:Adherens junction 4 2.4096385542168677 0.07319245476283873 INSRB, CREBBPB, SNAI2, CTNNA2 54 100 5434 4.025185185185185 0.98470935370402 0.566603730807367 52.65056173541227
COG_ONTOLOGY Chromatin structure and dynamics / Secondary metabolites biosynthesis, transport, and catabolism 2 1.2048192771084338 0.07327697579315233 HDAC5, HDAC8 21 8 2116 25.19047619047619 0.6281657502649716 0.6281657502649716 39.57896078328621
INTERPRO IPR000286:Histone deacetylase superfamily 2 1.2048192771084338 0.07723905387150269 HDAC5, HDAC8 156 11 21293 24.817016317016318 0.9999999999999962 0.9912850854367988 67.58125161105626
INTERPRO IPR003128:Villin headpiece 2 1.2048192771084338 0.07723905387150269 SI:DKEYP-57F11.2, ABLIM1B 156 11 21293 24.817016317016318 0.9999999999999962 0.9912850854367988 67.58125161105626
GOTERM_MF_DIRECT GO:0051082~unfolded protein binding 3 1.8072289156626504 0.07833284765658721 HSP90AB1, CALR, CANX 119 62 15797 6.423285443209543 0.9999999178094371 0.983068108036485 64.02729178215087
GOTERM_BP_DIRECT GO:0007010~cytoskeleton organization 3 1.8072289156626504 0.07924486721503396 SI:DKEYP-57F11.2, FMNL3, ABLIM1B 115 61 14912 6.37719173200285 0.9999999999793483 0.7450902430323197 66.78408335835728
GOTERM_BP_DIRECT GO:0048589~developmental growth 2 1.2048192771084338 0.08097767387629394 INSRB, NIPBLB 115 11 14912 23.576284584980236 0.9999999999882194 0.7340510774135776 67.60893069859941
GOTERM_BP_DIRECT GO:0030866~cortical actin cytoskeleton organization 2 1.2048192771084338 0.08097767387629394 EPB41B, AMOTL2A 115 11 14912 23.576284584980236 0.9999999999882194 0.7340510774135776 67.60893069859941
GOTERM_BP_DIRECT GO:0046034~ATP metabolic process 2 1.2048192771084338 0.08097767387629394 ATP5B, ATP5A1 115 11 14912 23.576284584980236 0.9999999999882194 0.7340510774135776 67.60893069859941
SMART SM00153:VHP 2 1.2048192771084338 0.08187311900341913 SI:DKEYP-57F11.2, ABLIM1B 95 11 12157 23.266985645933016 0.9999458235140224 0.9999458235140224 62.205239317033275
GOTERM_MF_DIRECT GO:0003713~transcription coactivator activity 3 1.8072289156626504 0.09172531864462327 CREBBPB, TFDP1B, MAML3 119 68 15797 5.856524962926348 0.9999999956001119 0.9786848532700023 70.05695566387827
GOTERM_BP_DIRECT GO:0006412~translation 6 3.614457831325301 0.09300955039095779 SLC25A5, NIPBLB, EIF3S10, GARS, RPL23A, SLC25A43 115 314 14912 2.477762392689006 0.999999999999768 0.766503465308086 72.83422621930671
UP_KEYWORDS Phosphoprotein 5 3.0120481927710845 0.09460786937331545 HSP90AB1, INSRB, EPHB3A, AMOTL2A, CFTR 162 240 22487 2.89184670781893 0.9999933868884028 0.8629956651387727 68.04677203877642
UP_SEQ_FEATURE compositionally biased region:Poly-Gln 2 1.2048192771084338 0.09623970223517525 INTS7, ARNT2 17 17 2704 18.71280276816609 0.9989728790185358 0.9989728790185358 64.69079026009747

Supplementary Table 10: Circular RNA junctions overlapping with lo

CircENd	CircID	CircStrand	Circ_Blood	Circ_'Brain	Circ_Gills	Circ_Heart	Circ_Muscle	chr	start	stop	strand	gid	gene	locus	length	coverage
1661319	Circ28	+	0	0	9	0	0	chr1	1661098	1668111	+	NM_178099	atp1a1a.5	1661097-1668493	7014	-
1661319	Circ28	+	0	0	9	0	0	chr1	1661103	1668493	+	NM_178099	atp1a1a.5	1661097-1668493	7391	-
1678645	Circ29	-	0	0	3	0	0	chr1	1661098	1668111	+	NM_178099	atp1a1a.5	1661097-1668493	7014	-
1678645	Circ29	-	0	0	3	0	0	chr1	1661103	1668493	+	NM_178099	atp1a1a.5	1661097-1668493	7391	-
54746137	Circ130	-	16	2	22	6	5	chr1	54730250	54732305	+	NM_130954	dla	54677659-54749049	2056	-
54746137	Circ130	-	16	2	22	6	5	chr1	54677660	54749049	+	NM_130954	dla	54677659-54749049	71390	-
38790770	Circ268	-	0	0	2	0	0	chr2	38763830	38774500	+	NM_212895	rbp1b	38763829-38774500	10671	-
55479330	Circ315	-	7	2	12	5	5	chr2	55465659	55466883	+	NM_131797	twsg1a	55465658-55466883	1225	-
57410727	Circ317	-	3	0	0	0	0	chr2	57409868	57411715	+	NM_001030070	gpx4b	57409867-57411715	1848	-
13992019	Circ397	+	0	0	0	3	0	chr3	13949156	13981088	+	NM_001002218	nfil3-6	13949155-13981088	31933	-
13992162	Circ398	+	0	0	0	2	0	chr3	13949156	13981088	+	NM_001002218	nfil3-6	13949155-13981088	31933	-
14044459	Circ399	+	0	0	3	0	0	chr3	13949156	13981088	+	NM_001002218	nfil3-6	13949155-13981088	31933	-
55658450	Circ517	+	0	0	4	0	0	chr3	55651523	55651776	+	NM_001020671	ubald1b	55651522-55651776	254	-
55662357	Circ518	+	0	0	3	0	0	chr3	55651523	55651776	+	NM_001020671	ubald1b	55651522-55651776	254	-
55662357	Circ519	+	0	0	6	3	0	chr3	55651523	55651776	+	NM_001020671	ubald1b	55651522-55651776	254	-
58582876	Circ526	-	0	0	4	0	0	chr3	58580511	58582554	+	NM_001080585	syngr2a	58580510-58582554	2044	-
60697985	Circ535	+	4	0	4	2	0	chr3	60638698	60656489	-	NM_001130778	zgc:194562	60638697-60656489	17792	-
557643	Circ544	-	0	0	2	0	0	chr4	486164	569225	+	NM_001002170	mapre3b	486163-569225	83062	-
9852062	Circ567	-	0	0	0	3	0	chr4	9836620	9850020	+	NM_001045067	si:dkey-207j16.2	9836619-9850020	13401	-
61143001	Circ611	-	0	0	2	0	0	chr4	61139037	61145088	+	NM_200610	dopey2	61139036-61145088	6052	-
61144153	Circ614	-	0	0	5	0	0	chr4	61139037	61145088	+	NM_200610	dopey2	61139036-61145088	6052	-
61140304	Circ615	-	0	0	28	0	0	chr4	61139037	61145088	+	NM_200610	dopey2	61139036-61145088	6052	-
61145088	Circ616	-	0	0	27	0	0	chr4	61139037	61145088	+	NM_200610	dopey2	61139036-61145088	6052	-
61150048	Circ617	-	0	0	20	0	0	chr4	61139037	61145088	+	NM_200610	dopey2	61139036-61145088	6052	-
61150048	Circ617	-	0	0	20	0	0	chr4	61147339	61149974	+	NM_200610	dopey2	61147338-61149974	2636	-
61415983	Circ618	+	0	0	0	0	2	chr4	61318900	61414548	+	NM_001044876	LOC558818	61318899-61414548	95649	-
2268544	Circ630	-	2	0	0	0	0	chr5	2159784	2244065	+	NM_001020688	vkorc1l1	2159783-2244065	84282	-

2278928	Circ631	-	7	0	0	0	0	chr5	2159784	2244065	+	NM_001020688	vkorc1l1	2159783-2244065	84282	-
2279450	Circ632	-	4	0	0	4	0	chr5	2159784	2244065	+	NM_001020688	vkorc1l1	2159783-2244065	84282	-
24928482	Circ665	-	0	0	2	0	0	chr5	24925077	24935130	+	NM_001005586	plp1b	24925076-24935130	10054	-
24928482	Circ665	-	0	0	2	0	0	chr5	24925077	24935032	+	NM_001005586	plp1b	24925076-24935130	9956	-
66581558	Circ766	+	0	0	9	0	0	chr5	66559384	66573660	+	NM_001089344	gsnb	66559383-66586964	14277	-
66581558	Circ766	+	0	0	9	0	0	chr5	66559421	66586964	+	NM_001089344	gsnb	66559383-66586964	27544	-
66582268	Circ767	+	0	0	4	0	0	chr5	66559384	66573660	+	NM_001089344	gsnb	66559383-66586964	14277	-
66582268	Circ767	+	0	0	4	0	0	chr5	66559421	66586964	+	NM_001089344	gsnb	66559383-66586964	27544	-
1711916	Circ810	-	0	0	0	10	0	chr6	1710546	1712429	+	NM_213212	myl9b	1710545-1712429	1884	-
1756100	Circ812	-	2	0	0	0	0	chr6	1737793	1781413	+	NM_213212	myl9b	1737792-1781413	43621	-
8529659	Circ829	+	0	0	6	0	0	chr6	8460758	8464224	+	NM_001111249	LOC100006122	8460757-8464777	3467	-
8529659	Circ829	+	0	0	6	0	0	chr6	8463418	8464777	+	NM_001111249	LOC100006122	8460757-8464777	1360	-
32471399	Circ864	-	0	0	2	0	0	chr6	32415144	32531676	+	NM_001082940	mafa	32415143-32531676	116533	-
730938	Circ917	+	2	0	0	0	0	chr7	730749	731278	+	NM_205541	drap1	730748-731278	530	-
4563716	Circ926	+	0	0	2	0	0	chr7	4511174	4529094	+	NM_001045001	slc12a10.2	4511173-4529094	17921	-
22954703	Circ969	-	0	0	0	2	0	chr7	22954071	23006917	+	NM_001077144	tmem88b	22954070-23006917	52847	-
22954703	Circ969	-	0	0	0	2	0	chr7	22954695	23006917	+	NM_001077144	tmem88b	22954070-23006917	52223	-
65357283	Circ1059	-	0	0	3	3	3	chr7	65167386	65410082	+	NM_001003435	mrpl15	65167385-65410082	242697	-
76725764	Circ1089	-	0	0	0	6	8	chr7	76721459	76727712	+	NM_001076724	casq1b	76721458-76727712	6254	-
20341050	Circ1129	+	0	0	2	0	0	chr8	20277144	20341397	+	NM_001270477	LOC566028	20277143-20341397	64254	-
49315474	Circ1204	-	2	0	0	0	0	chr8	49310800	49315172	+	NM_001009902	ppih	49310799-49315172	4373	-
2380527	Circ1226	-	2	0	0	0	0	chr9	2378699	2386167	+	NM_213000	chn1	2378698-2386167	7469	-
38872625	Circ1315	+	0	0	0	2	0	chr9	38869574	38872521	+	NM_001113576	pdia5	38869573-38872521	2948	-
114706	Circ1364	-	0	0	6	3	0	chr10	66084	72595	+	NM_001102623	zgc:171929	66083-72595	6512	-
22030670	Circ1442	+	0	2	0	0	0	chr10	22026522	22055954	+	NM_001012656	pcdh1gc5	22026521-22055954	29433	-
34289021	Circ1462	-	0	0	0	2	0	chr10	34222496	34295864	+	NM_213165	bcl7ba	34222495-34295864	73369	-
32125487	Circ1565	+	0	0	0	2	0	chr11	32110058	32145614	+	NM_001014332	man2b1	32110057-32145614	35557	-
38124625	Circ1568	+	2	0	3	0	3	chr11	37985110	38154470	+	NM_203484	cacna1da	37985109-38154470	169361	-
7094967	Circ1625	+	0	0	0	2	0	chr12	7057148	7176832	+	NM_131003	dkk1b	7057147-7176855	119685	-

7094967	Circ1625	+	0	0	0	2	0	chr12	7069263	7176855	+	NM_131003	dkk1b	7057147-7176855	107593	-
7094967	Circ1625	+	0	0	0	2	0	chr12	7074104	7176855	+	NM_131003	dkk1b	7057147-7176855	102752	-
7105110	Circ1626	+	0	2	0	0	0	chr12	7057148	7176832	+	NM_131003	dkk1b	7057147-7176855	119685	-
7105110	Circ1626	+	0	2	0	0	0	chr12	7069263	7176855	+	NM_131003	dkk1b	7057147-7176855	107593	-
7105110	Circ1626	+	0	2	0	0	0	chr12	7074104	7176855	+	NM_131003	dkk1b	7057147-7176855	102752	-
48140870	Circ1717	+	6	0	0	4	0	chr12	48061639	48151709	+	NM_001098781	lhpp	48061638-48151709	90071	-
48151709	Circ1718	+	3	5	2	7	20	chr12	48061639	48151709	+	NM_001098781	lhpp	48061638-48151709	90071	-
2129356	Circ1740	-	0	0	0	2	0	chr13	2128510	2129324	+	NM_001003727	klhl31	2128509-2129324	815	-
26781508	Circ1793	-	0	0	3	0	0	chr13	26776690	26927690	+	NM_212982	fancl	26776689-26927690	151001	-
1160870	Circ1847	-	0	0	0	0	2	chr14	1087007	1088035	+	NM_212882	cyyr1	1087006-1088035	1029	-
6428537	Circ1861	+	0	0	3	0	0	chr14	6428301	6434768	+	NM_001114586	abca1b	6428300-6434768	6468	-
48570732	Circ1917	-	0	0	2	0	0	chr14	48565579	48573723	+	NM_001135980	zgc:194285		0	
48570732	Circ1917	-	0	0	2	0	0	chr14	48565579	48573723	+	NM_001135980	zgc:194285	48565578-48574855	8145	-
48570732	Circ1917	-	0	0	2	0	0	chr14	48566284	48574855	-	NM_001135980	zgc:194285	48565578-48574855	8572	-
40266099	Circ2003	-	3	0	0	3	0	chr15	40246947	40270021	-	NM_001007387	ftr72	40246946-40270745	23075	-
40266099	Circ2003	-	3	0	0	3	0	chr15	40246947	40270745	-	NM_001007387	ftr72	40246946-40270745	23799	-
40273876	Circ2004	-	2	0	0	0	0	chr15	40246947	40270021	-	NM_001007387	ftr72	40246946-40270745	23075	-
40273876	Circ2004	-	2	0	0	0	0	chr15	40246947	40270745	-	NM_001007387	ftr72	40246946-40270745	23799	-
40259563	Circ2005	-	0	0	2	3	0	chr15	40246947	40270021	-	NM_001007387	ftr72	40246946-40270745	23075	-
40259563	Circ2005	-	0	0	2	3	0	chr15	40246947	40270745	-	NM_001007387	ftr72	40246946-40270745	23799	-
40278820	Circ2006	-	4	0	0	0	0	chr15	40246947	40270021	-	NM_001007387	ftr72	40246946-40270745	23075	-
40278820	Circ2006	-	4	0	0	0	0	chr15	40246947	40270745	-	NM_001007387	ftr72	40246946-40270745	23799	-
40273339	Circ2007	-	2	0	0	0	0	chr15	40246947	40270021	-	NM_001007387	ftr72	40246946-40270745	23075	-
40273339	Circ2007	-	2	0	0	0	0	chr15	40246947	40270745	-	NM_001007387	ftr72	40246946-40270745	23799	-
40250970	Circ2008	-	3	0	0	0	0	chr15	40246947	40270021	-	NM_001007387	ftr72	40246946-40270745	23075	-
40250970	Circ2008	-	3	0	0	0	0	chr15	40246947	40270745	-	NM_001007387	ftr72	40246946-40270745	23799	-
46781743	Circ2029	+	18	0	12	0	0	chr15	46779860	46780418	-	NM_213169	mpzl3	46779859-46780418	559	-
54502062	Circ2161	+	0	0	4	0	0	chr16	54498983	54501006	+	NM_001017775	zgc:110372	54483265-54538333	2024	-
54502062	Circ2161	+	0	0	4	0	0	chr16	54483266	54526085	+	NM_001017775	zgc:110372	54483265-54538333	42820	-

54502062	Circ2161	+	0	0	4	0	0	chr16	54483266	54538333	+	NM_001017775	zgc:110372	54483265-54538333	55068	-
54512226	Circ2162	+	0	0	4	0	0	chr16	54498983	54501006	+	NM_001017775	zgc:110372	54483265-54538333	2024	-
54512226	Circ2162	+	0	0	4	0	0	chr16	54483266	54526085	+	NM_001017775	zgc:110372	54483265-54538333	42820	-
54512226	Circ2162	+	0	0	4	0	0	chr16	54483266	54538333	+	NM_001017775	zgc:110372	54483265-54538333	55068	-
54516754	Circ2163	+	0	0	12	0	0	chr16	54498983	54501006	+	NM_001017775	zgc:110372	54483265-54538333	2024	-
54516754	Circ2163	+	0	0	12	0	0	chr16	54483266	54526085	+	NM_001017775	zgc:110372	54483265-54538333	42820	-
54516754	Circ2163	+	0	0	12	0	0	chr16	54483266	54538333	+	NM_001017775	zgc:110372	54483265-54538333	55068	-
54519866	Circ2164	+	0	0	3	0	0	chr16	54498983	54501006	+	NM_001017775	zgc:110372	54483265-54538333	2024	-
54519866	Circ2164	+	0	0	3	0	0	chr16	54483266	54526085	+	NM_001017775	zgc:110372	54483265-54538333	42820	-
54519866	Circ2164	+	0	0	3	0	0	chr16	54483266	54538333	+	NM_001017775	zgc:110372	54483265-54538333	55068	-
54519866	Circ2165	+	0	0	7	0	0	chr16	54498983	54501006	+	NM_001017775	zgc:110372	54483265-54538333	2024	-
54519866	Circ2165	+	0	0	7	0	0	chr16	54483266	54526085	+	NM_001017775	zgc:110372	54483265-54538333	42820	-
54519866	Circ2165	+	0	0	7	0	0	chr16	54483266	54538333	+	NM_001017775	zgc:110372	54483265-54538333	55068	-
8594882	Circ2233	-	2	0	0	0	0	chr17	8594133	8599903	-	NM_001111148	zgc:171416	8594132-8599903	5771	-
20564417	Circ2254	-	0	0	0	2	0	chr17	20530257	20620014	+	NM_199985	ccdc6a	20530256-20620014	89758	-
41157000	Circ2538	-	0	0	0	0	9	chr19	41146497	41151841	+	NM_001020802	grn1	41146496-41151841	5345	-
41157195	Circ2539	-	5	0	59	82	7	chr19	41146497	41151841	+	NM_001020802	grn1	41146496-41151841	5345	-
41158547	Circ2540	-	0	0	17	15	0	chr19	41146497	41151841	+	NM_001020802	grn1	41146496-41151841	5345	-
44165377	Circ2545	-	0	0	0	2	0	chr19	44163750	44206084	+	NM_001003520	fkbp9	44163749-44206084	42335	-
44173968	Circ2546	-	0	0	0	0	6	chr19	44163750	44206084	+	NM_001003520	fkbp9	44163749-44206084	42335	-
44174695	Circ2547	-	0	0	0	0	2	chr19	44163750	44206084	+	NM_001003520	fkbp9	44163749-44206084	42335	-
1435975	Circ2574	+	0	0	0	0	19	chr20	1433951	1435671	-	NM_001100056	LOC796447	1433950-1435671	1721	-
1439414	Circ2575	+	0	0	0	0	12	chr20	1433951	1435671	-	NM_001100056	LOC796447	1433950-1435671	1721	-
1441131	Circ2576	+	0	0	0	5	0	chr20	1433951	1435671	-	NM_001100056	LOC796447	1433950-1435671	1721	-
3010276	Circ2584	+	2	0	5	2	0	chr20	3009002	3012382	+	NM_001161601	amd1	3009001-3012382	3381	-
3010449	Circ2585	+	0	0	2	2	0	chr20	3009002	3012382	+	NM_001161601	amd1	3009001-3012382	3381	-
35762594	Circ2704	-	0	0	79	0	0	chr20	35737465	35851813	+	NM_001042688	tnfrsf21	35737464-35851813	114349	-
35769818	Circ2705	-	0	0	11	0	0	chr20	35737465	35851813	+	NM_001042688	tnfrsf21	35737464-35851813	114349	-
35760136	Circ2706	-	0	0	10	0	0	chr20	35737465	35851813	+	NM_001042688	tnfrsf21	35737464-35851813	114349	-

43240878	Circ2711	-	0	0	0	5	0	chr20	43154350	43272081	+	NM_001039620	si:dkeyp-95d10.1	43154349-43272081	117732	-
9942831	Circ2777	-	0	0	2	0	0	chr21	9927365	9929893	+	NM_001076714	grin1a	9927364-9929893	2529	-
18510570	Circ2793	-	0	0	2	0	0	chr21	18460130	18513711	+	NM_182870	fgf10a	18460129-18513711	53582	-
18510570	Circ2793	-	0	0	2	0	0	chr21	18460130	18513711	+	NM_182870	fgf10a		0	
43636803	Circ2842	-	4	0	0	2	0	chr21	43626795	43648489	-	NM_001012389	tcf7	43626794-43648489	21695	-
1854378	Circ2864	+	0	0	2	2	0	chr22	1845320	1864245	+	NM_001013285	zgc:110821	1845319-1864245	18926	-
7046134	Circ2878	-	0	0	3	0	0	chr22	6980206	7014459	+	NM_214791	asic1b	6980205-7014460	34254	-
7046134	Circ2878	-	0	0	3	0	0	chr22	7004998	7014460	+	NM_214791	asic1b	6980205-7014460	9463	-
7500945	Circ2879	+	0	0	0	0	2	chr22	7477205	7480339	+	NM_001003737	zgc:92041	7477204-7480339	3135	-
7500945	Circ2879	+	0	0	0	0	2	chr22	7477205	7480339	+	NM_001003737	zgc:92041		0	
7500945	Circ2880	+	0	0	3	9	11	chr22	7477205	7480339	+	NM_001003737	zgc:92041	7477204-7480339	3135	-
7500945	Circ2880	+	0	0	3	9	11	chr22	7477205	7480339	+	NM_001003737	zgc:92041		0	
7510368	Circ2881	+	0	0	4	3	14	chr22	7477205	7480339	+	NM_001003737	zgc:92041	7477204-7480339	3135	-
7510368	Circ2881	+	0	0	4	3	14	chr22	7477205	7480339	+	NM_001003737	zgc:92041		0	
8130936	Circ2884	-	0	0	2	0	0	chr22	8115144	8290526	+	NM_001122619	sc:d217	8115143-8290526	175383	-
8131427	Circ2885	-	0	0	0	2	0	chr22	8115144	8290526	+	NM_001122619	sc:d217	8115143-8290526	175383	-
8556733	Circ2888	-	0	0	0	2	0	chr22	8555677	8584424	+	NM_001122619	sc:d217	8555676-8584424	28748	-
8559992	Circ2889	+	0	2	0	2	0	chr22	8555677	8584424	+	NM_001122619	sc:d217	8555676-8584424	28748	-
38076739	Circ3236	+	0	0	2	0	0	chr24	38076526	38076730	+	NM_001033748	zgc:114120	38076525-38076730	205	-
39602160	Circ3238	-	0	0	14	0	0	chr24	39588330	39595602	+	NM_001114901	zgc:172225	39588329-39595602	7273	-
39602452	Circ3239	+	0	0	5	0	0	chr24	39588330	39595602	+	NM_001114901	zgc:172225	39588329-39595602	7273	-
40056450	Circ3243	-	0	0	0	9	2	chr24	40044817	40045037	+	NM_001077331	zgc:154125	40044816-40045037	221	-
20586330	Circ3339	+	0	0	0	0	5	chr25	20585896	20586483	+	NM_001002101	tnni1al	20585895-20586483	588	-
13713	Circ3377	+	0	0	2	0	0	Zv9_NA73	10596	13706	+	0	0	10595-13706	3111	-
15693	Circ3383	-	4	0	11	0	0	Zv9_NA250	13861	16234	+	0	0	13860-16287	2374	-
15693	Circ3383	-	4	0	11	0	0	Zv9_NA250	14182	16287	+	0	0	13860-16287	2106	-
15693	Circ3384	-	3	0	25	0	0	Zv9_NA250	13861	16234	+	0	0	13860-16287	2374	-
15693	Circ3384	-	3	0	25	0	0	Zv9_NA250	14182	16287	+	0	0	13860-16287	2106	-
13009	Circ3395	-	0	0	8	0	0	Zv9_NA401	9723	13023	+	0	0	9722-13023	3301	-

ong non-coding RNA from ZFlncRNApedia.

2-4cell	1-kcell	dome	shield	bud	1dpf	2dpf	3dpf	5dpf	adult	heart	blood	brain	kidney	liver	name	zf_lnc_id
0	-1.03	0	0	0	0	-1.24	-1.1	-0.79	0	0	-1.54	-3.79	-2.93	0	lnc1_atp1a1a.5	ZF_LNC000004
0	-3.1	0	0	0	0	-3.29	-0.32	-1.06	1.5	1.33	2.14	0.31	1.21	0	lnc2_atp1a1a.5	ZF_LNC000005
0	-1.03	0	0	0	0	-1.24	-1.1	-0.79	0	0	-1.54	-3.79	-2.93	0	lnc1_atp1a1a.5	ZF_LNC000004
0	-3.1	0	0	0	0	-3.29	-0.32	-1.06	1.5	1.33	2.14	0.31	1.21	0	lnc2_atp1a1a.5	ZF_LNC000005
0	0	0	0	0	0	0	0	1.02	0	0	0	1.16	0	0	mstx10	ZF_LNC000068
2.46	2.26	2.09	2.13	1.64	1.44	1.41	1.45	1.46	1.75	1.55	1.93	1.68	1.79	1.98	lnc_dla	ZF_LNC000067
1.24	1.26	0.68	0.66	0.86	1.37	1.56	1.13	1.03	0.99	1.26	0.96	1.3	0.83	0.37	lnc_rbp1b	ZF_LNC000971
3.95	3.43	3.12	2.77	2.31	2.32	2.6	2.4	2.13	2.2	1.9	1.71	2.17	2.08	1.65	twsg1apa	ZF_LNC000992
2.41	2.47	2.21	1.93	0.71	1.31	1.65	0	0.68	1.16	1.19	1.64	0.24	2.2	1.16	lnc_gpx4b	ZF_LNC001000
1.53	1.53	1.35	1.32	1.2	0.85	1.27	0.63	0.99	0.35	1	1.05	0.52	0.93	0.59	lnc_nfil3-6	ZF_LNC001449
1.53	1.53	1.35	1.32	1.2	0.85	1.27	0.63	0.99	0.35	1	1.05	0.52	0.93	0.59	lnc_nfil3-6	ZF_LNC001449
1.53	1.53	1.35	1.32	1.2	0.85	1.27	0.63	0.99	0.35	1	1.05	0.52	0.93	0.59	lnc_nfil3-6	ZF_LNC001449
0	0	0	0	0	2.17	2.7	2.18	2.88	2.45	2.19	1.89	2.36	2.1	0	lnc_ubald1b	ZF_LNC001538
0	0	0	0	0	2.17	2.7	2.18	2.88	2.45	2.19	1.89	2.36	2.1	0	lnc_ubald1b	ZF_LNC001538
0	0	0	0	0	2.17	2.7	2.18	2.88	2.45	2.19	1.89	2.36	2.1	0	lnc_ubald1b	ZF_LNC001538
2.91	2.8	2.62	2.41	2.54	2.05	2.22	2.33	2.42	2.26	1.57	2.1	1.34	2.24	1.89	syngr2aos	ZF_LNC001546
0.58	0.83	0.53	0.39	-0.01	-0.93	0.1	0.52	1.19	0.85	2.21	1.11	2.61	1.27	1.33	mstx130	ZF_LNC001550
1.29	1.41	1.22	1.5	2.03	0.73	1.19	1.41	1.61	1.49	1.46	0.97	1.69	0.79	0.91	mapre3bos	ZF_LNC001558
-0.25	-0.82	0.24	0.17	0.42	0.72	0.61	0.36	1.2	0.43	0.86	0.29	0.81	0.28	1.45	lnc2_si:dkey-207j16.2	ZF_LNC001594
3.07	2.78	2.27	2.1	1.97	0.59	0.28	0	-0.51	2.19	0	1.4	-0.3	1	2.6	dopey2os1	ZF_LNC001679
3.07	2.78	2.27	2.1	1.97	0.59	0.28	0	-0.51	2.19	0	1.4	-0.3	1	2.6	dopey2os1	ZF_LNC001679
3.07	2.78	2.27	2.1	1.97	0.59	0.28	0	-0.51	2.19	0	1.4	-0.3	1	2.6	dopey2os1	ZF_LNC001679
3.07	2.78	2.27	2.1	1.97	0.59	0.28	0	-0.51	2.19	0	1.4	-0.3	1	2.6	dopey2os1	ZF_LNC001679
3.07	2.78	2.27	2.1	1.97	0.59	0.28	0	-0.51	2.19	0	1.4	-0.3	1	2.6	dopey2os1	ZF_LNC001679
3.04	2.42	1.88	1.76	1.79	0.15	0.43	-0.46	-1.28	3.63	0	1.01	0	0.53	2.04	dopey2os2	ZF_LNC001680
0.62	0.61	0.21	0.13	-0.39	-0.86	-0.35	-0.49	-0.37	0.77	0.26	2.38	-0.91	0.72	0	LOC558818os2	ZF_LNC001684
1.39	1.21	0.96	0.73	0.76	0.58	0.75	0.94	0.58	0.08	1.25	1.31	0.84	1.2	1.45	lnc_vkorc1l1	ZF_LNC001701

1.39	1.21	0.96	0.73	0.76	0.58	0.75	0.94	0.58	0.08	1.25	1.31	0.84	1.2	1.45	lnc_vkorc1l1	ZF_LNC001701
1.39	1.21	0.96	0.73	0.76	0.58	0.75	0.94	0.58	0.08	1.25	1.31	0.84	1.2	1.45	lnc_vkorc1l1	ZF_LNC001701
-1.48	0.61	-1.48	-1.48	0.79	1.06	1.47	1.38	0.9	1.06	-1.48	-1.48	-1.47	-1.47	-1.47	lnc_plp1b	ZF_LNC001728
1.31	0.51	0.83	0.47	-0.1	0.94	-0.61	1.14	1.11	0	0.58	2.04	0.65	0.63	0.7	rakt40	ZF_LNC001729
2.34	2.05	1.92	0.81	1.08	0.85	0.72	0.54	0.38	0	0.4	0	0.58	0.53	1.22	edal88	ZF_LNC001807
2.03	2.49	2.13	1.35	1.14	0.78	0.7	0.48	0.75	1.7	1.12	1.51	0.76	1.11	1.18	edal89	ZF_LNC001808
2.34	2.05	1.92	0.81	1.08	0.85	0.72	0.54	0.38	0	0.4	0	0.58	0.53	1.22	edal88	ZF_LNC001807
2.03	2.49	2.13	1.35	1.14	0.78	0.7	0.48	0.75	1.7	1.12	1.51	0.76	1.11	1.18	edal89	ZF_LNC001808
2.11	1.67	1.68	1.83	2.7	3.32	3.36	2.8	2.97	3.03	3.82	2.09	2.49	2.8	2.67	myl9boa	ZF_LNC001823
1.05	1.1	0.86	0.87	0.83	1.04	1.31	1.02	1.39	0.22	0.79	0.59	1.67	0.97	0.36	lnc_myl9b	ZF_LNC001824
0	-0.25	-0.08	0.23	0.2	-0.24	0.69	-0.22	-0.08	0	0	0	0	0	0	lnc_LOC100006122	ZF_LNC001832
0	1.03	0.76	0.55	1.03	1.14	1.32	0.86	1	0	0	1.01	0	0	1.3	ykrt13	ZF_LNC001833
1.09	1.44	1.13	0.89	0.43	0.25	0.4	0.16	0.42	0.56	0.57	0.73	0.53	0.49	0.67	lnc2_mafa	ZF_LNC001873
3.6	3.12	2.83	2.84	2.78	2.34	2.53	2.28	2.2	2.58	2.5	3.11	2.66	2.8	2.89	drap1oa	ZF_LNC001921
-0.55	-0.19	-0.22	0.15	0.12	-0.93	-0.42	-0.43	0.43	-0.47	0	0.34	-0.18	-0.08	0	slc12a10.2oa	ZF_LNC001927
1.8	1.14	1.1	1	0.58	0.06	-0.37	0.47	0.41	0.7	-2.36	-2.36	-4.63	0.31	0	tmem88boa1	ZF_LNC001952
1.28	-0.55	-1.12	0.25	0.75	1.07	0.95	0.46	-0.02	-2.27	2.16	0.17	-3.82	-2.01	-3.79	tmem88boa2	ZF_LNC001953
0.64	0.67	0.83	1.4	1.06	0.95	0.91	0.99	1.47	1.47	0.95	0.84	1.24	0.96	0.95	mrpl15oa	ZF_LNC002019
-0.28	0.04	-0.09	-0.06	0.25	0.75	1.75	1.94	1.55	1.32	2.01	0.8	0.68	0.58	1.09	casq1bos	ZF_LNC002039
0.41	0.11	-0.22	-0.33	-0.69	0.41	0.54	0.63	0.4	0.27	0.35	-0.7	-0.14	0.34	0	LOC566028os	ZF_LNC002078
0.68	-0.31	1.64	1.34	0.67	0.73	0.04	0.49	0.3	0.46	0	0	0	-0.3	0	ppihos	ZF_LNC002118
2.37	2.02	1.23	0.93	1.29	1.18	1.69	1.45	1.8	0.52	1.29	1.15	2.1	1.34	0.53	chn1pa	ZF_LNC002139
0	0	-0.41	-0.64	-0.29	-0.47	0.29	0.12	0.42	0.34	0	0	0.63	-0.12	0	pdia5oa	ZF_LNC002190
0.83	0.6	0.54	0.35	0.44	0.14	0.4	0.3	0.89	1.06	1.41	1.23	1.08	1.13	1.77	zgc:171929oa	ZF_LNC000080
0.28	-0.03	-1.13	-1.16	0.55	1.6	2.37	2.34	2.29	1.23	1.55	0.34	2.55	0.89	1.04	pcdh1gc5oa	ZF_LNC000119
1.33	1.46	1.33	1.52	1.37	0.83	1.02	0.58	0.81	0.31	0.79	0.99	0.63	0.86	1.17	bcl7baoa	ZF_LNC000140
-0.34	-0.05	-0.45	-0.89	-0.3	0.5	0.23	0.2	0.58	0.13	1.5	1.07	0.6	0.3	-0.15	lnc_man2b1	ZF_LNC000221
-0.82	-0.76	-0.65	-0.39	-0.01	0.44	0.68	0.42	1.08	0.51	0.73	0.59	0.55	0.78	2.19	lnc_cacna1da	ZF_LNC000225
-0.1	-0.08	-0.18	-0.44	-0.47	-0.37	-0.51	-1.29	0.32	0	-0.4	0	-0.31	0.05	-4.66	dkk1boa1	ZF_LNC000264

-4.41	-0.68	-0.24	-0.62	-3.66	-3.04	-2.17	-4.7	-1.53	-3.86	-3.86	-3.85	0.11	-2.53	0.06	dkk1boa2	ZF_LNC000265
-4.97	-4.02	0.29	0.76	0.7	0.32	1.09	1.1	1.02	0.57	1.07	-0.25	-0.1	0.17	0	dkk1boa3	ZF_LNC000266
-0.1	-0.08	-0.18	-0.44	-0.47	-0.37	-0.51	-1.29	0.32	0	-0.4	0	-0.31	0.05	-4.66	dkk1boa1	ZF_LNC000264
-4.41	-0.68	-0.24	-0.62	-3.66	-3.04	-2.17	-4.7	-1.53	-3.86	-3.86	-3.85	0.11	-2.53	0.06	dkk1boa2	ZF_LNC000265
-4.97	-4.02	0.29	0.76	0.7	0.32	1.09	1.1	1.02	0.57	1.07	-0.25	-0.1	0.17	0	dkk1boa3	ZF_LNC000266
0.36	0.28	-0.28	-0.64	-0.25	0.3	0.59	0.68	1.15	1.7	1.19	0.93	1.59	2.08	1.94	lhpppa	ZF_LNC000326
0.36	0.28	-0.28	-0.64	-0.25	0.3	0.59	0.68	1.15	1.7	1.19	0.93	1.59	2.08	1.94	lhpppa	ZF_LNC000326
1.68	1.28	0.68	0.5	1.58	3.21	3.36	2.52	3.4	1.69	3.4	1.43	0	1.33	0	klhl31oa	ZF_LNC000338
0.29	0.18	-0.51	-1.13	-1.13	-1.11	-0.72	-0.85	0.25	0.01	-1.17	0	-0.36	-0.5	-0.64	fanclpa1	ZF_LNC000365
0	0	-0.35	0.22	0.8	0.94	0.99	0.57	0.55	0	1.17	0	0.53	0	0	cyyr1ia9	ZF_LNC000407
-0.17	-0.69	0.58	0.13	0.75	0.36	0.43	-0.83	0.47	-0.02	-0.27	0	0.45	0.22	1.5	abca1boa	ZF_LNC000415
0	0	0	0	0	0	0	0	0	0	0	0	0	0	0	lnc1_zgc:194285	ZF_LNC000478
3.25	2.78	3.63	4.11	3.63	3.85	4.3	4.18	4.61	3.67	4.07	3.85	4.06	3.96	4.22	lnc2_zgc:194285	ZF_LNC000479
0.66	0.22	0.87	1.67	0.25	2.26	0	1.82	2.94	1.18	0	0	1.62	1.77	2.77	ykrt33	ZF_LNC000480
0.22	-0.7	1.15	1.51	-1.33	-0.04	0.05	-0.03	0.09	-3.38	0.67	-2.89	0.46	-2.75	0	lnc1_ftr72	ZF_LNC000556
-2.85	-2.84	0.47	0.68	0.2	-0.04	-0.74	-1.05	-0.01	0.95	1.27	1.92	0.9	1.19	1.06	lnc2_ftr72	ZF_LNC000557
0.22	-0.7	1.15	1.51	-1.33	-0.04	0.05	-0.03	0.09	-3.38	0.67	-2.89	0.46	-2.75	0	lnc1_ftr72	ZF_LNC000556
-2.85	-2.84	0.47	0.68	0.2	-0.04	-0.74	-1.05	-0.01	0.95	1.27	1.92	0.9	1.19	1.06	lnc2_ftr72	ZF_LNC000557
0.22	-0.7	1.15	1.51	-1.33	-0.04	0.05	-0.03	0.09	-3.38	0.67	-2.89	0.46	-2.75	0	lnc1_ftr72	ZF_LNC000556
-2.85	-2.84	0.47	0.68	0.2	-0.04	-0.74	-1.05	-0.01	0.95	1.27	1.92	0.9	1.19	1.06	lnc2_ftr72	ZF_LNC000557
0.22	-0.7	1.15	1.51	-1.33	-0.04	0.05	-0.03	0.09	-3.38	0.67	-2.89	0.46	-2.75	0	lnc1_ftr72	ZF_LNC000556
-2.85	-2.84	0.47	0.68	0.2	-0.04	-0.74	-1.05	-0.01	0.95	1.27	1.92	0.9	1.19	1.06	lnc2_ftr72	ZF_LNC000557
0.22	-0.7	1.15	1.51	-1.33	-0.04	0.05	-0.03	0.09	-3.38	0.67	-2.89	0.46	-2.75	0	lnc1_ftr72	ZF_LNC000556
-2.85	-2.84	0.47	0.68	0.2	-0.04	-0.74	-1.05	-0.01	0.95	1.27	1.92	0.9	1.19	1.06	lnc2_ftr72	ZF_LNC000557
0.22	-0.7	1.15	1.51	-1.33	-0.04	0.05	-0.03	0.09	-3.38	0.67	-2.89	0.46	-2.75	0	lnc1_ftr72	ZF_LNC000556
-2.85	-2.84	0.47	0.68	0.2	-0.04	-0.74	-1.05	-0.01	0.95	1.27	1.92	0.9	1.19	1.06	lnc2_ftr72	ZF_LNC000557
1.78	1.58	1.41	2.11	1.32	2.09	1.64	1.95	2.27	2.48	2.08	2.55	1.72	2.07	2.23	mpzl3ia	ZF_LNC000572
0.49	0	1.94	0.93	0.65	1.73	1.33	1.21	1.57	0	2.41	2.27	1.63	1.73	2.18	lnc4_zgc:110372	ZF_LNC000697
-0.16	-0.25	-0.4	-4.41	-4.86	-0.44	-3.09	-4.62	-0.46	0	0	0.87	0	0	0	lnc2_zgc:110372	ZF_LNC000695

-0.76	-0.96	-0.22	-0.3	-0.53	0.36	0.47	0.5	0.43	1.13	1.37	1.08	0.27	0.85	0.67	lnc3_zgc:110372	ZF_LNC000696
0.49	0	1.94	0.93	0.65	1.73	1.33	1.21	1.57	0	2.41	2.27	1.63	1.73	2.18	lnc4_zgc:110372	ZF_LNC000697
-0.16	-0.25	-0.4	-4.41	-4.86	-0.44	-3.09	-4.62	-0.46	0	0	0.87	0	0	0	lnc2_zgc:110372	ZF_LNC000695
-0.76	-0.96	-0.22	-0.3	-0.53	0.36	0.47	0.5	0.43	1.13	1.37	1.08	0.27	0.85	0.67	lnc3_zgc:110372	ZF_LNC000696
0.49	0	1.94	0.93	0.65	1.73	1.33	1.21	1.57	0	2.41	2.27	1.63	1.73	2.18	lnc4_zgc:110372	ZF_LNC000697
-0.16	-0.25	-0.4	-4.41	-4.86	-0.44	-3.09	-4.62	-0.46	0	0	0.87	0	0	0	lnc2_zgc:110372	ZF_LNC000695
-0.76	-0.96	-0.22	-0.3	-0.53	0.36	0.47	0.5	0.43	1.13	1.37	1.08	0.27	0.85	0.67	lnc3_zgc:110372	ZF_LNC000696
0.49	0	1.94	0.93	0.65	1.73	1.33	1.21	1.57	0	2.41	2.27	1.63	1.73	2.18	lnc4_zgc:110372	ZF_LNC000697
-0.16	-0.25	-0.4	-4.41	-4.86	-0.44	-3.09	-4.62	-0.46	0	0	0.87	0	0	0	lnc2_zgc:110372	ZF_LNC000695
-0.76	-0.96	-0.22	-0.3	-0.53	0.36	0.47	0.5	0.43	1.13	1.37	1.08	0.27	0.85	0.67	lnc3_zgc:110372	ZF_LNC000696
0.49	0	1.94	0.93	0.65	1.73	1.33	1.21	1.57	0	2.41	2.27	1.63	1.73	2.18	lnc4_zgc:110372	ZF_LNC000697
-0.16	-0.25	-0.4	-4.41	-4.86	-0.44	-3.09	-4.62	-0.46	0	0	0.87	0	0	0	lnc2_zgc:110372	ZF_LNC000695
-0.76	-0.96	-0.22	-0.3	-0.53	0.36	0.47	0.5	0.43	1.13	1.37	1.08	0.27	0.85	0.67	lnc3_zgc:110372	ZF_LNC000696
0.95	1.49	1.26	1.16	0.94	0.01	0.07	-0.04	0.51	1.66	0.82	1.06	0.62	1.17	0.95	lnc_zgc:171416	ZF_LNC000714
0.36	0.24	-0.28	-0.63	-0.08	0.99	1.48	1.43	1.7	0.52	1.85	0.75	2.03	1.09	0.29	ccdc6apa	ZF_LNC000726
0.87	0.71	0.39	0.25	0.28	-0.3	0.11	0.41	1.55	3.02	3.05	3.02	1.28	3.86	2.35	grn1os	ZF_LNC000902
0.87	0.71	0.39	0.25	0.28	-0.3	0.11	0.41	1.55	3.02	3.05	3.02	1.28	3.86	2.35	grn1os	ZF_LNC000902
0.87	0.71	0.39	0.25	0.28	-0.3	0.11	0.41	1.55	3.02	3.05	3.02	1.28	3.86	2.35	grn1os	ZF_LNC000902
1.27	1.04	0.5	0.55	1.37	1.93	1.71	1.78	0.98	1.08	1.38	1.55	0.81	0.96	1.26	fkbp9os	ZF_LNC000906
1.27	1.04	0.5	0.55	1.37	1.93	1.71	1.78	0.98	1.08	1.38	1.55	0.81	0.96	1.26	fkbp9os	ZF_LNC000906
1.27	1.04	0.5	0.55	1.37	1.93	1.71	1.78	0.98	1.08	1.38	1.55	0.81	0.96	1.26	fkbp9os	ZF_LNC000906
0	0	0	0	-0.65	-0.81	0.08	0.24	-0.57	0	0	0	0	0.42	1.19	LOC796447oa	ZF_LNC001011
0	0	0	0	-0.65	-0.81	0.08	0.24	-0.57	0	0	0	0	0.42	1.19	LOC796447oa	ZF_LNC001011
0	0	0	0	-0.65	-0.81	0.08	0.24	-0.57	0	0	0	0	0.42	1.19	LOC796447oa	ZF_LNC001011
2.52	2.67	2.62	2.74	3.25	3.1	3.24	2.93	3.03	2.63	2.98	2.8	3.29	3.23	2.57	amd1oa	ZF_LNC001016
2.52	2.67	2.62	2.74	3.25	3.1	3.24	2.93	3.03	2.63	2.98	2.8	3.29	3.23	2.57	amd1oa	ZF_LNC001016
-0.28	-0.3	0.43	0.02	0.22	0.12	0.63	0.57	0.75	0.78	0.3	1.43	1.02	0.8	-0.06	tnfrsf21os	ZF_LNC001050
-0.28	-0.3	0.43	0.02	0.22	0.12	0.63	0.57	0.75	0.78	0.3	1.43	1.02	0.8	-0.06	tnfrsf21os	ZF_LNC001050
-0.28	-0.3	0.43	0.02	0.22	0.12	0.63	0.57	0.75	0.78	0.3	1.43	1.02	0.8	-0.06	tnfrsf21os	ZF_LNC001050

1.73	1.46	1	0.17	0.67	0.52	0.55	0.15	1	0.23	1.26	1.04	0.82	0.76	1.33	lnc_si:dkeyp-95d10.1	ZF_LNC001061
0.55	0.66	-0.52	0.04	1.66	2.67	2.88	2.31	1.96	2.26	2.15	1.94	1.21	1.81	0	ledal7	ZF_LNC001097
1.04	0.75	-0.16	-0.47	-0.47	0.54	0.76	0.5	0.62	0	-0.59	-0.22	-0.31	-0.45	-0.33	fgf10aoa1	ZF_LNC001107
0	0	0	0	0	0	0	0	0	0	0	0	0	0	0	fgf10aoa2	ZF_LNC001108
0.77	0.67	0.41	0.39	0.96	0.84	1.04	0.82	0.91	1.1	1.29	1.93	1.24	1.62	1.29	tcf7ia	ZF_LNC001144
0.76	1.21	1.31	1.26	0.88	0.81	0.87	0.61	0.77	1.02	1.11	1.39	0.98	1.19	1.12	zgc:110821ia	ZF_LNC001149
0.77	0.8	0.6	0.39	-0.15	0.17	0.58	0.97	1.16	1.14	0.46	0	1.5	1.11	1.01	asic1bos1	ZF_LNC001161
-3.8	0	-2.78	-0.46	0	-0.78	0	0	0	0	0	0	0	0	0	asic1bos2	ZF_LNC001162
2.67	2.26	1.71	1.15	-0.13	-0.17	-0.29	1.89	2.76	3.79	1.63	3.65	0	0	0.92	zgc:92041oa1	ZF_LNC001163
0	0	0	0	0	0	0	0	0	0	0	0	0	0	0	zgc:92041oa2	ZF_LNC001164
2.67	2.26	1.71	1.15	-0.13	-0.17	-0.29	1.89	2.76	3.79	1.63	3.65	0	0	0.92	zgc:92041oa1	ZF_LNC001163
0	0	0	0	0	0	0	0	0	0	0	0	0	0	0	zgc:92041oa2	ZF_LNC001164
2.67	2.26	1.71	1.15	-0.13	-0.17	-0.29	1.89	2.76	3.79	1.63	3.65	0	0	0.92	zgc:92041oa1	ZF_LNC001163
0	0	0	0	0	0	0	0	0	0	0	0	0	0	0	zgc:92041oa2	ZF_LNC001164
0.98	1.14	0.47	0.49	0.37	0.48	0.17	0.68	0.36	1.4	0.89	0.83	0.51	0.96	0.46	lnc1_sc:d217	ZF_LNC001167
0.98	1.14	0.47	0.49	0.37	0.48	0.17	0.68	0.36	1.4	0.89	0.83	0.51	0.96	0.46	lnc1_sc:d217	ZF_LNC001167
0.87	0.66	-0.11	-0.49	-0.71	0.51	-0.44	-0.25	0.04	0.85	0.15	0	-0.29	-0.43	0	lnc4_sc:d217	ZF_LNC001172
0.87	0.66	-0.11	-0.49	-0.71	0.51	-0.44	-0.25	0.04	0.85	0.15	0	-0.29	-0.43	0	lnc4_sc:d217	ZF_LNC001172
1.72	0	0	0	1.31	0.62	1.6	1.86	2.3	0	0	0	2.68	0	0	mstx74	ZF_LNC001364
1.91	1.9	1.55	1.28	0.39	-0.63	-0.71	-0.27	0.63	2.98	0	0.43	-0.08	1.52	3.23	zgc:172225oa	ZF_LNC001366
1.91	1.9	1.55	1.28	0.39	-0.63	-0.71	-0.27	0.63	2.98	0	0.43	-0.08	1.52	3.23	zgc:172225oa	ZF_LNC001366
0	0	0	0	0	1.48	0	0	0	0	2.56	2.17	0	2.66	0	zgc:154125pa	ZF_LNC001367
1.45	1.02	0.78	0	0.58	2.53	3.19	3.33	2.53	2.68	0.84	1.59	0.85	0.96	0	tnni1aloa	ZF_LNC001407
0.27	0.21	-0.21	-0.25	0	-0.43	-1.3	0	-0.49	0	0	0	0	0	0	mirl27	ZF_LNC002238
0.61	-3.07	1.92	1.8	1.51	0.8	1.09	1.73	0.6	-3.11	1.22	-3.05	2.13	-3.22	0	mstx42	ZF_LNC002223
1.61	0.9	2.34	2.49	2.27	1.79	1.72	0.91	1.24	1.58	0	1.11	2.21	1.55	1.7	lnc5	ZF_LNC002224
0.61	-3.07	1.92	1.8	1.51	0.8	1.09	1.73	0.6	-3.11	1.22	-3.05	2.13	-3.22	0	mstx42	ZF_LNC002223
1.61	0.9	2.34	2.49	2.27	1.79	1.72	0.91	1.24	1.58	0	1.11	2.21	1.55	1.7	lnc5	ZF_LNC002224
2.39	1.35	1.46	2.02	2.41	0.68	1.45	-0.76	0.57	1.82	0	0	0.45	0.97	0	edal75	ZF_LNC002230
